# Supplementary material for: Characterization of Host-Specific Genes from Pine- and Grass-Associated Species of the Fusarium fujikuroi Species Complex
Source: Pathogens. 2022 Jul 29;11(8):858. doi: 10.3390/pathogens11080858 (PMC9415769; doi:10.3390/pathogens11080858)
Supplement: Supplementary file 1 [file pathogens-11-00858-s001.zip › Supplemental Figures/Figure S1.pdf]

Figure S1. Phylogenetic relationship of paralogs with regards to the respective host-range-associated gene. FCIR = *Fusarium circinatum*; FFRAC = *Fusarium fracticaudum*; FPIN = *Fusarium pininemorale*; FSUB = *Fusarium subglutinans.*

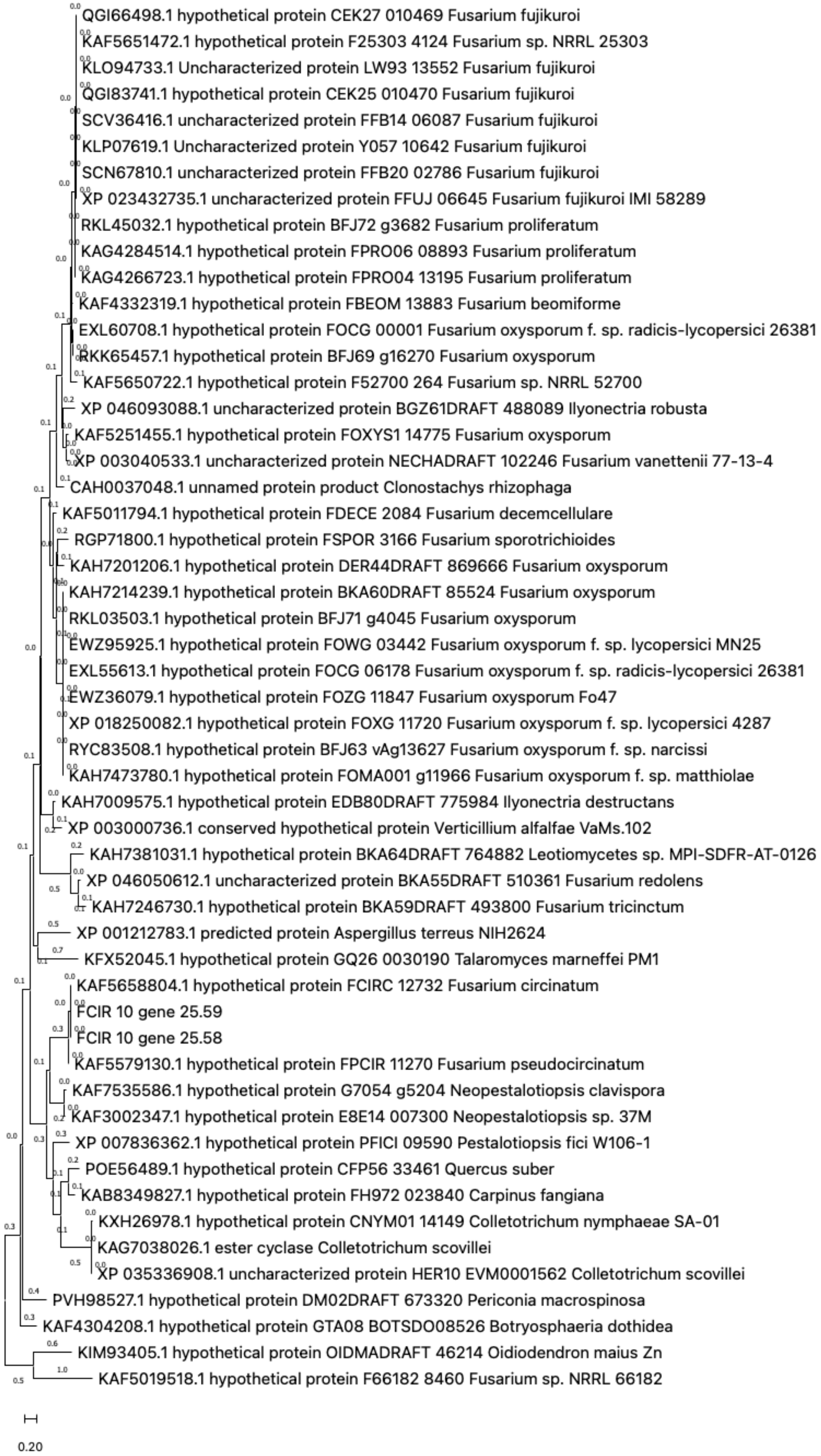
QGI66498.1 hypothetical protein CEK27 010469 Fusarium fujikuroi
KAF5651472.1 hypothetical protein F25303 4124 Fusarium sp. NRRL 25303
KLO94733.1 Uncharacterized protein LW93 13552 Fusarium fujikuroi
QGI83741.1 hypothetical protein CEK25 010470 Fusarium fujikuroi
SCV36416.1 uncharacterized protein FFB14 06087 Fusarium fujikuroi
KLP07619.1 Uncharacterized protein Y057 10642 Fusarium fujikuroi
SCN67810.1 uncharacterized protein FFB20 02786 Fusarium fujikuroi
XP 023432735.1 uncharacterized protein FFUJ 06645 Fusarium fujikuroi IMI 58289
RKL45032.1 hypothetical protein BFJ72 g3682 Fusarium proliferatum
KAG4284514.1 hypothetical protein FPRO06 08893 Fusarium proliferatum
KAG4266723.1 hypothetical protein FPRO04 13195 Fusarium proliferatum
KAF4332319.1 hypothetical protein FBEOM 13883 Fusarium beomiforme
EXL60708.1 hypothetical protein FOCG 00001 Fusarium oxysporum f. sp. radicis-lycopersici 26381
RKK65457.1 hypothetical protein BFJ69 g16270 Fusarium oxysporum
KAF5650722.1 hypothetical protein F52700 264 Fusarium sp. NRRL 52700
XP 046093088.1 uncharacterized protein BGZ61DRAFT 488089 Ilyonectria robusta
KAF5251455.1 hypothetical protein FOXYS1 14775 Fusarium oxysporum
XP 003040533.1 uncharacterized protein NECHADRAFT 102246 Fusarium vanettenii 77-13-4
CAH0037048.1 unnamed protein product Clonostachys rhizophaga
KAF5011794.1 hypothetical protein FDECE 2084 Fusarium decemcellulare
RGP71800.1 hypothetical protein FSPOR 3166 Fusarium sporotrichioides
KAH7201206.1 hypothetical protein DER44DRAFT 869666 Fusarium oxysporum
KAH7214239.1 hypothetical protein BKA60DRAFT 85524 Fusarium oxysporum
RKL03503.1 hypothetical protein BFJ71 g4045 Fusarium oxysporum
EWZ95925.1 hypothetical protein FOWG 03442 Fusarium oxysporum f. sp. lycopersici MN25
EXL55613.1 hypothetical protein FOCG 06178 Fusarium oxysporum f. sp. radicis-lycopersici 26381
EWZ36079.1 hypothetical protein FOZG 11847 Fusarium oxysporum Fo47
XP 018250082.1 hypothetical protein FOXG 11720 Fusarium oxysporum f. sp. lycopersici 4287
RYC83508.1 hypothetical protein BFJ63 vAg13627 Fusarium oxysporum f. sp. narcissi
KAH7473780.1 hypothetical protein FOMA001 g11966 Fusarium oxysporum f. sp. matthiolae
KAH7009575.1 hypothetical protein EDB80DRAFT 775984 Ilyonectria destructans
XP 003000736.1 conserved hypothetical protein Verticillium alfalfae VaMs.102
KAH7381031.1 hypothetical protein BKA64DRAFT 764882 Leotiomycetes sp. MPI-SDFR-AT-0126
XP 046050612.1 uncharacterized protein BKA55DRAFT 510361 Fusarium redolens
KAH7246730.1 hypothetical protein BKA59DRAFT 493800 Fusarium tricinctum
XP 001212783.1 predicted protein Aspergillus terreus NIH2624
KFX52045.1 hypothetical protein GQ26 0030190 Talaromyces marneffei PM1
KAF5658804.1 hypothetical protein FCIRC 12732 Fusarium circinatum
FCIR 10 gene 25.59
FCIR 10 gene 25.58
KAF5579130.1 hypothetical protein FPCIR 11270 Fusarium pseudocircinatum
KAF7535586.1 hypothetical protein G7054 g5204 Neopestalotiopsis clavispora
KAF3002347.1 hypothetical protein E8E14 007300 Neopestalotiopsis sp. 37M
XP 007836362.1 hypothetical protein PFICI 09590 Pestalotiopsis fici W106-1
POE56489.1 hypothetical protein CFP56 33461 Quercus suber
KAB8349827.1 hypothetical protein FH972 023840 Carpinus fangiana
KXH26978.1 hypothetical protein CNYM01 14149 Colletotrichum nymphaeae SA-01
KAG7038026.1 ester cyclase Colletotrichum scovillei
XP 035336908.1 uncharacterized protein HER10 EVM0001562 Colletotrichum scovillei
PVH98527.1 hypothetical protein DM02DRAFT 673320 Periconia macrospinosa
KAF4304208.1 hypothetical protein GTA08 BOTSDO08526 Botryosphaeria dothidea
KIM93405.1 hypothetical protein OIDMADRAFT 46214 Oidiodendron maius Zn
KAF5019518.1 hypothetical protein F66182 8460 Fusarium sp. NRRL 66182
0.20

The evolutionary history of FCIR_10_gene_25.58 and its paralog FCIR_10_gene_25.59 was inferred by using the Maximum Likelihood method and JTT matrix-based model. Initial tree(s) for the heuristic search were obtained automatically by applying Neighbor-Join and BioNJ algorithms to a matrix of pairwise distances estimated using the JTT model, and then selecting the topology with superior log likelihood value. A discrete Gamma distribution was used to model evolutionary rate differences among sites. The tree is drawn to scale, with branch lengths measured in the number of substitutions per site (next to the branches). Evolutionary analyses were conducted in MEGA11.

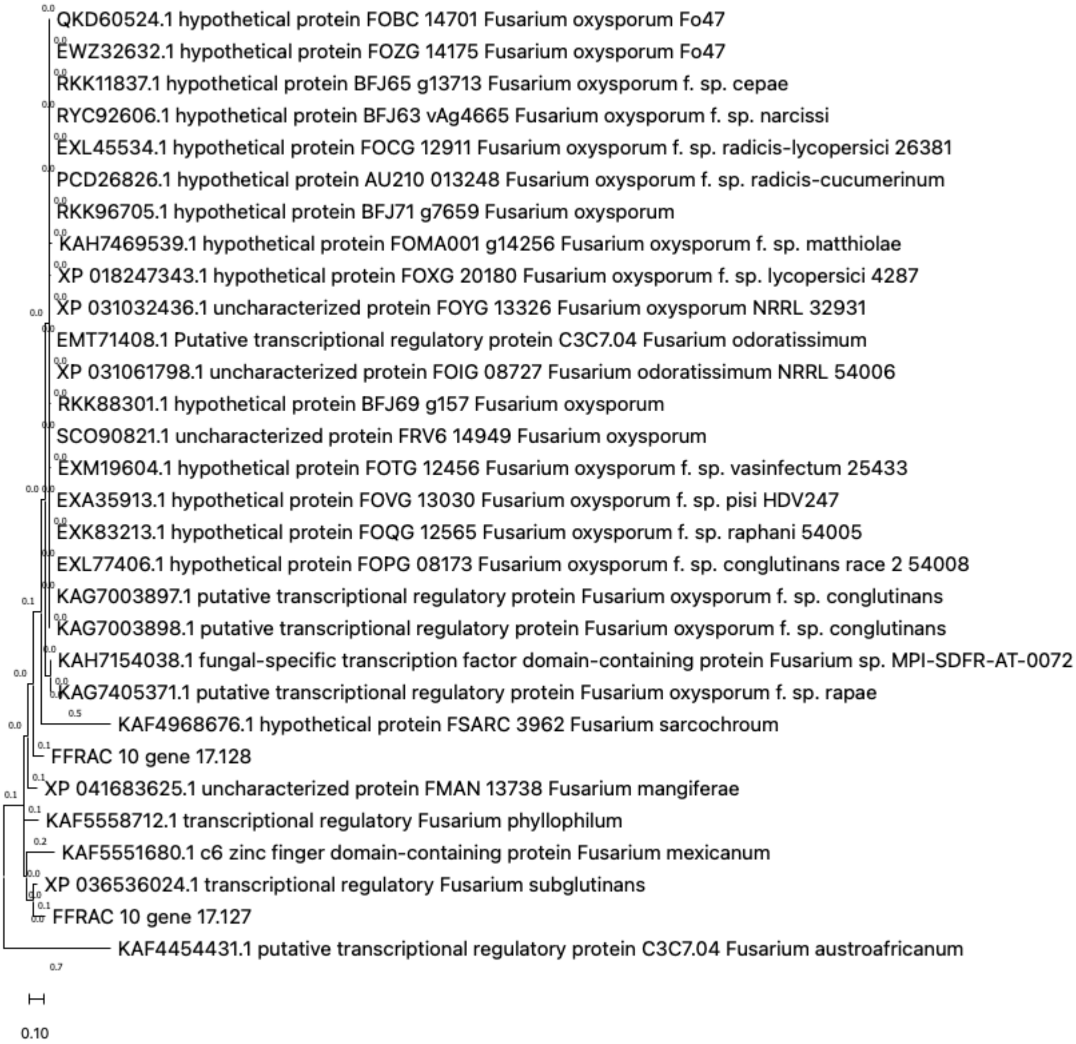
QKD60524.1 hypothetical protein FOBC 14701 Fusarium oxysporum Fo47
EWZ32632.1 hypothetical protein FOZG 14175 Fusarium oxysporum Fo47
RKK11837.1 hypothetical protein BFJ65 g13713 Fusarium oxysporum f. sp. cepae
RYC92606.1 hypothetical protein BFJ63 vAg4665 Fusarium oxysporum f. sp. narcissi
EXL45534.1 hypothetical protein FOCG 12911 Fusarium oxysporum f. sp. radicis-lycopersici 26381
PCD26826.1 hypothetical protein AU210 013248 Fusarium oxysporum f. sp. radicis-cucumerinum
RKK96705.1 hypothetical protein BFJ71 g7659 Fusarium oxysporum
KAH7469539.1 hypothetical protein FOMA001 g14256 Fusarium oxysporum f. sp. matthiolae
XP 018247343.1 hypothetical protein FOXG 20180 Fusarium oxysporum f. sp. lycopersici 4287
XP 031032436.1 uncharacterized protein FOYG 13326 Fusarium oxysporum NRRL 32931
EMT71408.1 Putative transcriptional regulatory protein C3C7.04 Fusarium odoratissimum
XP 031061798.1 uncharacterized protein FOIG 08727 Fusarium odoratissimum NRRL 54006
RKK88301.1 hypothetical protein BFJ69 g157 Fusarium oxysporum
SCO90821.1 uncharacterized protein FRV6 14949 Fusarium oxysporum
EXM19604.1 hypothetical protein FOTG 12456 Fusarium oxysporum f. sp. vasinfectum 25433
EXA35913.1 hypothetical protein FOVG 13030 Fusarium oxysporum f. sp. pisi HDV247
EXK83213.1 hypothetical protein FOQG 12565 Fusarium oxysporum f. sp. raphani 54005
EXL77406.1 hypothetical protein FOPG 08173 Fusarium oxysporum f. sp. conglutinans race 2 54008
KAG7003897.1 putative transcriptional regulatory protein Fusarium oxysporum f. sp. conglutinans
KAG7003898.1 putative transcriptional regulatory protein Fusarium oxysporum f. sp. conglutinans
KAH7154038.1 fungal-specific transcription factor domain-containing protein Fusarium sp. MPI-SDFR-AT-0072
KAG7405371.1 putative transcriptional regulatory protein Fusarium oxysporum f. sp. rapae
KAF4968676.1 hypothetical protein FSARC 3962 Fusarium sarcochroum
FFRAC 10 gene 17.128
XP 041683625.1 uncharacterized protein FMAN 13738 Fusarium mangiferae
KAF5558712.1 transcriptional regulatory Fusarium phyllophilum
KAF5551680.1 c6 zinc finger domain-containing protein Fusarium mexicanum
XP 036536024.1 transcriptional regulatory Fusarium subglutinans
FFRAC 10 gene 17.127
KAF4454431.1 putative transcriptional regulatory protein C3C7.04 Fusarium austroafricanum
0.5
0.7
0.10

The evolutionary history of FFRAC_10_gene_17.127 and its paralog FFRAC_10_gene_17.128 was inferred by using the Maximum Likelihood method and JTT matrix-based model. Initial tree(s) for the heuristic search were obtained automatically by applying Neighbor-Join and BioNJ algorithms to a matrix of pairwise distances estimated using the JTT model, and then selecting the topology with superior log likelihood value. A discrete Gamma distribution was used to model evolutionary rate differences among sites. The tree is drawn to scale, with branch lengths measured in the number of substitutions per site (next to the branches). Evolutionary analyses were conducted in MEGA11.

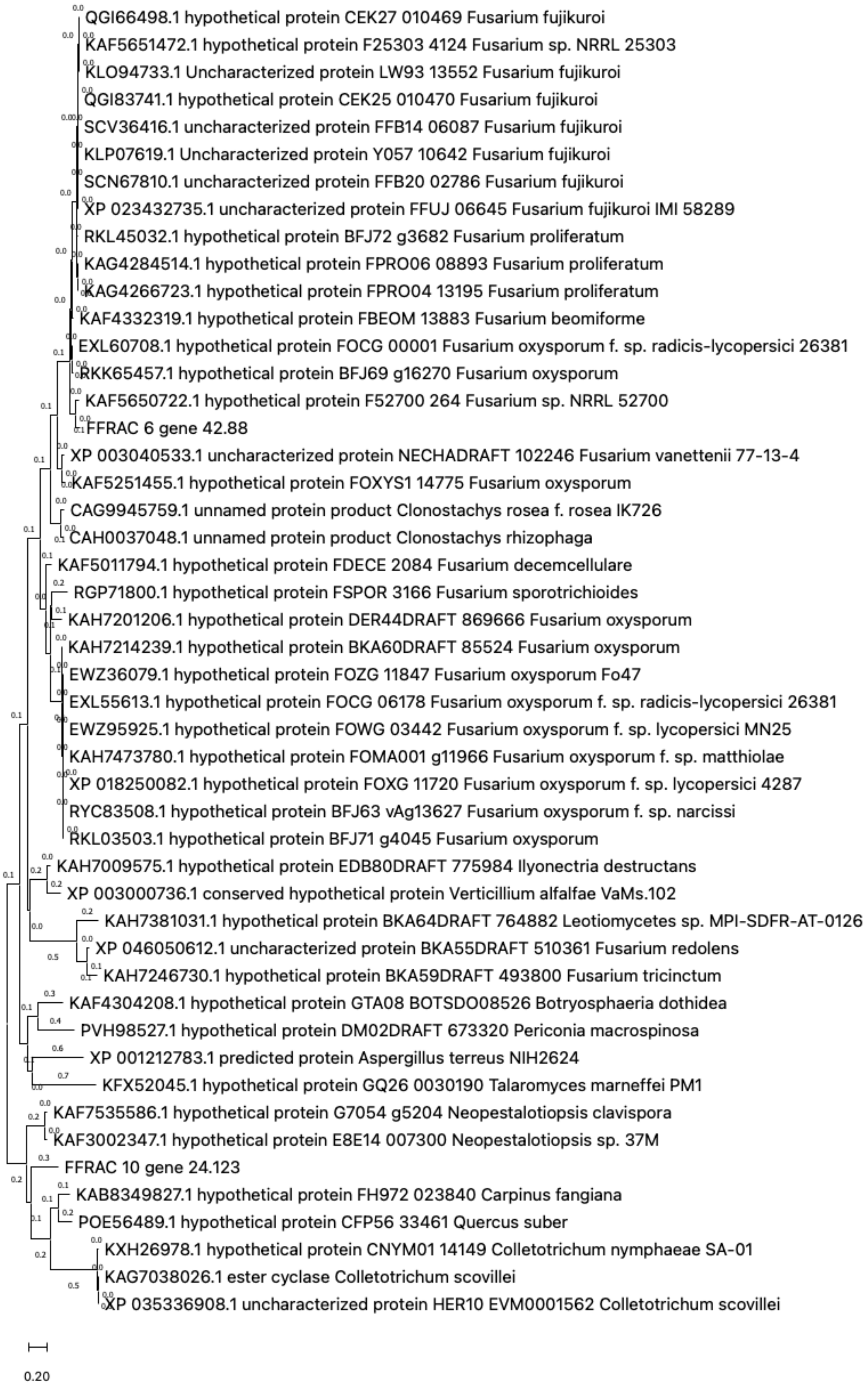

QGI66498.1 hypothetical protein CEK27 010469 Fusarium fujikuroi
KAF5651472.1 hypothetical protein F25303 4124 Fusarium sp. NRRL 25303
KLO94733.1 Uncharacterized protein LW93 13552 Fusarium fujikuroi
QGI83741.1 hypothetical protein CEK25 010470 Fusarium fujikuroi
SCV36416.1 uncharacterized protein FFB14 06087 Fusarium fujikuroi
KLP07619.1 Uncharacterized protein Y057 10642 Fusarium fujikuroi
SCN67810.1 uncharacterized protein FFB20 02786 Fusarium fujikuroi
XP 023432735.1 uncharacterized protein FFUJ 06645 Fusarium fujikuroi IMI 58289
RKL45032.1 hypothetical protein BFJ72 g3682 Fusarium proliferatum
KAG4284514.1 hypothetical protein FPRO06 08893 Fusarium proliferatum
KAG4266723.1 hypothetical protein FPRO04 13195 Fusarium proliferatum
KAF4332319.1 hypothetical protein FBEOM 13883 Fusarium beomiforme
EXL60708.1 hypothetical protein FOCG 00001 Fusarium oxysporum f. sp. radicis-lycopersici 26381
RKK65457.1 hypothetical protein BFJ69 g16270 Fusarium oxysporum
KAF5650722.1 hypothetical protein F52700 264 Fusarium sp. NRRL 52700
FFRAC 6 gene 42.88
XP 003040533.1 uncharacterized protein NECHADRAFT 102246 Fusarium vanettenii 77-13-4
KAF5251455.1 hypothetical protein FOXYS1 14775 Fusarium oxysporum
CAG9945759.1 unnamed protein product Clonostachys rosea f. rosea IK726
CAH0037048.1 unnamed protein product Clonostachys rhizophaga
KAF5011794.1 hypothetical protein FDECE 2084 Fusarium decemcellulare
RGP71800.1 hypothetical protein FSPOR 3166 Fusarium sporotrichioides
KAH7201206.1 hypothetical protein DER44DRAFT 869666 Fusarium oxysporum
KAH7214239.1 hypothetical protein BKA60DRAFT 85524 Fusarium oxysporum
EWZ36079.1 hypothetical protein FOZG 11847 Fusarium oxysporum Fo47
EXL55613.1 hypothetical protein FOCG 06178 Fusarium oxysporum f. sp. radicis-lycopersici 26381
EWZ95925.1 hypothetical protein FOWG 03442 Fusarium oxysporum f. sp. lycopersici MN25
KAH7473780.1 hypothetical protein FOMA001 g11966 Fusarium oxysporum f. sp. matthiolae
XP 018250082.1 hypothetical protein FOXG 11720 Fusarium oxysporum f. sp. lycopersici 4287
RYC83508.1 hypothetical protein BFJ63 vAg13627 Fusarium oxysporum f. sp. narcissi
RKL03503.1 hypothetical protein BFJ71 g4045 Fusarium oxysporum
KAH7009575.1 hypothetical protein EDB80DRAFT 775984 Ilyonectria destructans
XP 003000736.1 conserved hypothetical protein Verticillium alfalfae VaMs.102
KAH7381031.1 hypothetical protein BKA64DRAFT 764882 Leotiomycetes sp. MPI-SDFR-AT-0126
XP 046050612.1 uncharacterized protein BKA55DRAFT 510361 Fusarium redolens
KAH7246730.1 hypothetical protein BKA59DRAFT 493800 Fusarium tricinctum
KAF4304208.1 hypothetical protein GTA08 BOTSDO08526 Botryosphaeria dothidea
PVH98527.1 hypothetical protein DM02DRAFT 673320 Periconia macrospinosa
XP 001212783.1 predicted protein Aspergillus terreus NIH2624
KFX52045.1 hypothetical protein GQ26 0030190 Talaromyces marneffei PM1
KAF7535586.1 hypothetical protein G7054 g5204 Neopestalotiopsis clavispora
KAF3002347.1 hypothetical protein E8E14 007300 Neopestalotiopsis sp. 37M
FFRAC 10 gene 24.123
KAB8349827.1 hypothetical protein FH972 023840 Carpinus fangiana
POE56489.1 hypothetical protein CFP56 33461 Quercus suber
KXH26978.1 hypothetical protein CNYM01 14149 Colletotrichum nymphaeae SA-01
KAG7038026.1 ester cyclase Colletotrichum scovillei
XP 035336908.1 uncharacterized protein HER10 EVM0001562 Colletotrichum scovillei
0.20

The evolutionary history of FFRAC_10_gene_24.123 and its paralog FFRAC_6_gene_42.88 was inferred by using the Maximum Likelihood method and JTT matrix-based model. Initial tree(s) for the heuristic search were obtained automatically by applying Neighbor-Join and BioNJ algorithms to a matrix of pairwise distances estimated using the JTT model, and then selecting the topology with superior log likelihood value. A discrete Gamma distribution was used to model evolutionary rate differences among sites. The tree is drawn to scale, with branch lengths measured in the number of substitutions per site (next to the branches). Evolutionary analyses were conducted in MEGA11.

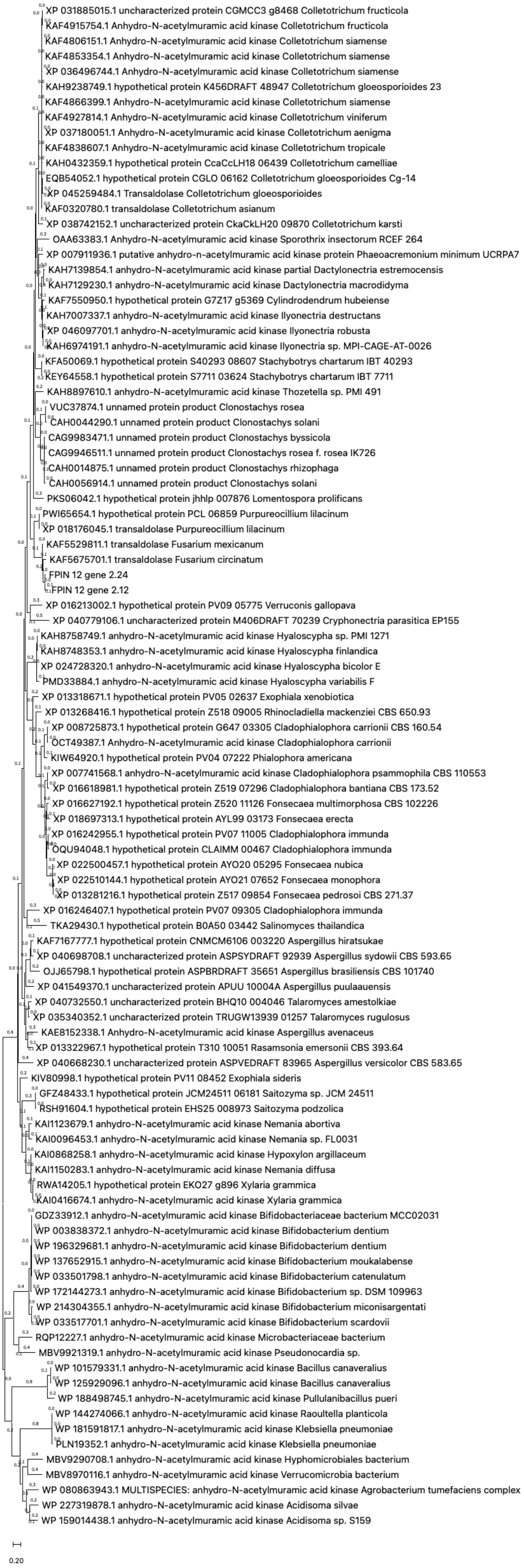
XP 031885015.1 uncharacterized protein CGMCC3 g8468 Colletotrichum fructicola
KAF4915754.1 Anhydro-N-acetylmuramic acid kinase Colletotrichum fructicola
KAF4806151.1 Anhydro-N-acetylmuramic acid kinase Colletotrichum siamense
KAF4853354.1 Anhydro-N-acetylmuramic acid kinase Colletotrichum siamense
XP 036496744.1 Anhydro-N-acetylmuramic acid kinase Colletotrichum siamense
KAH9238749.1 hypothetical protein K456DRAFT 48947 Colletotrichum gloeosporioides 23
KAF4866399.1 Anhydro-N-acetylmuramic acid kinase Colletotrichum siamense
KAF4927814.1 Anhydro-N-acetylmuramic acid kinase Colletotrichum viniferum
XP 037180051.1 Anhydro-N-acetylmuramic acid kinase Colletotrichum aenigma
KAF4838607.1 Anhydro-N-acetylmuramic acid kinase Colletotrichum tropicale
KAH0432359.1 hypothetical protein CcaCcLH18 06439 Colletotrichum camelliae
EQB54052.1 hypothetical protein CGLO 06162 Colletotrichum gloeosporioides Cg-14
XP 045259484.1 Transaldolase Colletotrichum gloeosporioides
KAF0320780.1 transaldolase Colletotrichum asianum
XP 038742152.1 uncharacterized protein CkaCkLH20 09870 Colletotrichum karsti
OAA63383.1 Anhydro-N-acetylmuramic acid kinase Sporothrix insectorum RCEF 264
XP 007911936.1 putative anhydro-n-acetylmuramic acid kinase protein Phaeoacremonium minimum UCRPA7
KAH7139854.1 anhydro-N-acetylmuramic acid kinase partial Dactylonectria estremocensis
KAH7129230.1 anhydro-N-acetylmuramic acid kinase Dactylonectria macrodidyma
KAF7550950.1 hypothetical protein G7Z17 g5369 Cylindrodendrum hubeiense
KAH7007337.1 anhydro-N-acetylmuramic acid kinase Ilyonectria destructans
XP 046097701.1 anhydro-N-acetylmuramic acid kinase Ilyonectria robusta
KAH6974191.1 anhydro-N-acetylmuramic acid kinase Ilyonectria sp. MPI-CAGE-AT-0026
KFA50069.1 hypothetical protein S40293 08607 Stachybotrys chartarum IBT 40293
KEY64558.1 hypothetical protein S7711 03624 Stachybotrys chartarum IBT 7711
KAH8897610.1 anhydro-N-acetylmuramic acid kinase Thozetella sp. PMI 491
VUC37874.1 unnamed protein product Clonostachys rosea
CAH0044290.1 unnamed protein product Clonostachys solani
CAG9983471.1 unnamed protein product Clonostachys byssicola
CAG9946511.1 unnamed protein product Clonostachys rosea f. rosea IK726
CAH0014875.1 unnamed protein product Clonostachys rhizophaga
CAH0056914.1 unnamed protein product Clonostachys solani
PKS06042.1 hypothetical protein jhhlp 007876 Lomentospora prolificans
PWI65654.1 hypothetical protein PCL 06859 Purpureocillium lilacinum
XP 018176045.1 transaldolase Purpureocillium lilacinum
KAF5529811.1 transaldolase Fusarium mexicanum
KAF5675701.1 transaldolase Fusarium circinatum
FPIN 12 gene 2.24
FPIN 12 gene 2.12
XP 016213002.1 hypothetical protein PV09 05775 Verruconis gallopava
XP 040779106.1 uncharacterized protein M406DRAFT 70239 Cryphonectria parasitica EP155
KAH8758749.1 anhydro-N-acetylmuramic acid kinase Hyaloscypha sp. PMI 1271
KAH8748353.1 anhydro-N-acetylmuramic acid kinase Hyaloscypha finlandica
XP 024728320.1 anhydro-N-acetylmuramic acid kinase Hyaloscypha bicolor E
PMD33884.1 anhydro-N-acetylmuramic acid kinase Hyaloscypha variabilis F
XP 013318671.1 hypothetical protein PV05 02637 Exophiala xenobiotica
XP 013268416.1 hypothetical protein Z518 09005 Rhinocladiella mackenziei CBS 650.93
XP 008725873.1 hypothetical protein G647 03305 Cladophialophora carrionii CBS 160.54
OCT49387.1 Anhydro-N-acetylmuramic acid kinase Cladophialophora carrionii
KIW64920.1 hypothetical protein PV04 07222 Phialophora americana
XP 007741568.1 anhydro-N-acetylmuramic acid kinase Cladophialophora psammophila CBS 110553
XP 016618981.1 hypothetical protein Z519 07296 Cladophialophora bantiana CBS 173.52
XP 016627192.1 hypothetical protein Z520 11126 Fonsecaea multimorphosa CBS 102226
XP 018697313.1 hypothetical protein AYL99 03173 Fonsecaea erecta
XP 016242955.1 hypothetical protein PV07 11005 Cladophialophora immunda
OQU94048.1 hypothetical protein CLAIMM 00467 Cladophialophora immunda
XP 022500457.1 hypothetical protein AYO20 05295 Fonsecaea nubica
XP 022510144.1 hypothetical protein AYO21 07652 Fonsecaea monophora
XP 013281216.1 hypothetical protein Z517 09854 Fonsecaea pedrosoi CBS 271.37
XP 016246407.1 hypothetical protein PV07 09305 Cladophialophora immunda
TKA29430.1 hypothetical protein B0A50 03442 Salinomyces thailandica
KAF7167777.1 hypothetical protein CNMCM6106 003220 Aspergillus hiratsukae
XP 040698708.1 uncharacterized protein ASPSYDRAFT 92939 Aspergillus sydowii CBS 593.65
OJJ65798.1 hypothetical protein ASPBRDRAFT 35651 Aspergillus brasiliensis CBS 101740
XP 041549370.1 uncharacterized protein APUU 10004A Aspergillus puulaauensis
XP 040732550.1 uncharacterized protein BHQ10 004046 Talaromyces amestolkiae
XP 035340352.1 uncharacterized protein TRUGW13939 01257 Talaromyces rugulosus
KAE8152338.1 Anhydro-N-acetylmuramic acid kinase Aspergillus avenaceus
XP 013322967.1 hypothetical protein T310 10051 Rasamsonia emersonii CBS 393.64
XP 040668230.1 uncharacterized protein ASPVEDRAFT 83965 Aspergillus versicolor CBS 583.65
KIV80998.1 hypothetical protein PV11 08452 Exophiala sideris
GFZ48433.1 hypothetical protein JCM24511 06181 Saitozyma sp. JCM 24511
RSH91604.1 hypothetical protein EHS25 008973 Saitozyma podzolica
KAI1123679.1 anhydro-N-acetylmuramic acid kinase Nemania abortiva
KAI0096453.1 anhydro-N-acetylmuramic acid kinase Nemania sp. FL0031
KAI0868258.1 anhydro-N-acetylmuramic acid kinase Hypoxylon argillaceum
KAI1150283.1 anhydro-N-acetylmuramic acid kinase Nemania diffusa
RWA14205.1 hypothetical protein EKO27 g896 Xylaria grammica
KAI0416674.1 anhydro-N-acetylmuramic acid kinase Xylaria grammica
GDZ33912.1 anhydro-N-acetylmuramic acid kinase Bifidobacteriaceae bacterium MCC02031
WP 003838372.1 anhydro-N-acetylmuramic acid kinase Bifidobacterium dentium
WP 196329681.1 anhydro-N-acetylmuramic acid kinase Bifidobacterium dentium
WP 137652915.1 anhydro-N-acetylmuramic acid kinase Bifidobacterium moukalabense
WP 033501798.1 anhydro-N-acetylmuramic acid kinase Bifidobacterium catenulatum
WP 172144273.1 anhydro-N-acetylmuramic acid kinase Bifidobacterium sp. DSM 109963
WP 214304355.1 anhydro-N-acetylmuramic acid kinase Bifidobacterium miconisargentati
WP 033517701.1 anhydro-N-acetylmuramic acid kinase Bifidobacterium scardovii
RQP12227.1 anhydro-N-acetylmuramic acid kinase Microbacteriaceae bacterium
MBV9921319.1 anhydro-N-acetylmuramic acid kinase Pseudonocardia sp.
WP 101579331.1 anhydro-N-acetylmuramic acid kinase Bacillus canaveralius
WP 125929096.1 anhydro-N-acetylmuramic acid kinase Bacillus canaveralius
WP 188498745.1 anhydro-N-acetylmuramic acid kinase Pullulanibacillus pueri
WP 144274066.1 anhydro-N-acetylmuramic acid kinase Raoultella planticola
WP 181591817.1 anhydro-N-acetylmuramic acid kinase Klebsiella pneumoniae
PLN19352.1 anhydro-N-acetylmuramic acid kinase Klebsiella pneumoniae
MBV9290708.1 anhydro-N-acetylmuramic acid kinase Hyphomicrobiales bacterium
MBV8970116.1 anhydro-N-acetylmuramic acid kinase Verrucomicrobia bacterium
WP 080863943.1 MULTISPECIES: anhydro-N-acetylmuramic acid kinase Agrobacterium tumefaciens complex
WP 227319878.1 anhydro-N-acetylmuramic acid kinase Acidisoma silvae
WP 159014438.1 anhydro-N-acetylmuramic acid kinase Acidisoma sp. S159
0.20

The evolutionary history of FPIN_12_gene_2.12 and its paralog FPIN_12_gene_2.24 was inferred by using the Maximum Likelihood method and the Le Gascuel model. Initial tree(s) for the heuristic search were obtained automatically by applying Neighbor-Join and BioNJ algorithms to a matrix of pairwise distances estimated using the JTT model, and then selecting the topology with superior log likelihood value. A discrete Gamma distribution was used to model evolutionary rate differences among sites. The tree is drawn to scale, with branch lengths measured in the number of substitutions per site (next to the branches). Evolutionary analyses were conducted in MEGA11.

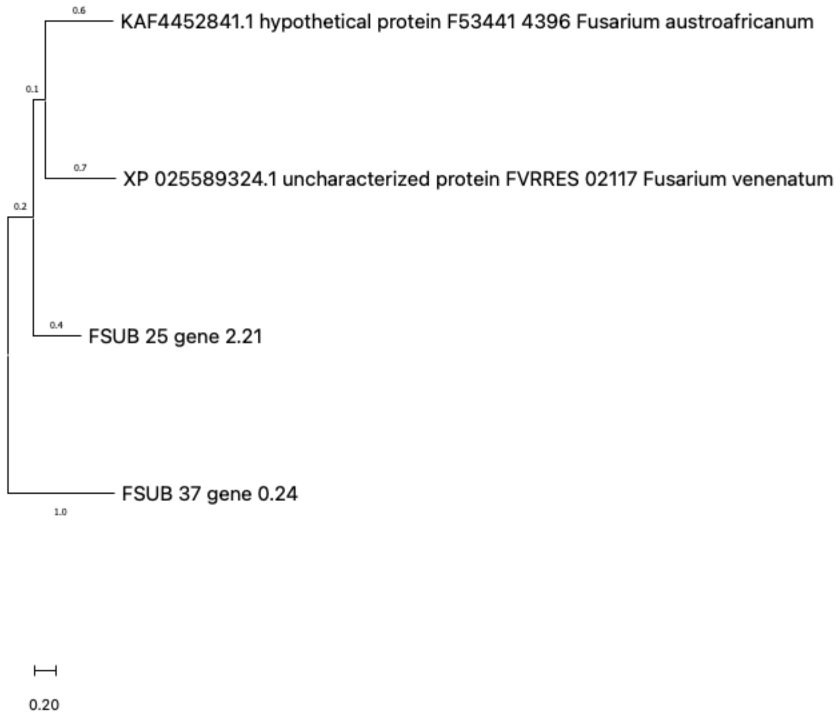
0.6
KAF4452841.1 hypothetical protein F53441 4396 Fusarium austroafricanum
0.1
0.7
XP 025589324.1 uncharacterized protein FVRRES 02117 Fusarium venenatum
0.2
0.4
FSUB 25 gene 2.21
FSUB 37 gene 0.24
1.0
0.20

The evolutionary history of FSUB_25_gene_2.21 and its paralog FSUB_37_gene_0.24 was inferred by using the Maximum Likelihood method and the Le Gascuel model. Initial tree(s) for the heuristic search were obtained automatically by applying Neighbor-Join and BioNJ algorithms to a matrix of pairwise distances estimated using the JTT model, and then selecting the topology with superior log likelihood value. A discrete Gamma distribution was used to model evolutionary rate differences among sites. The tree is drawn to scale, with branch lengths measured in the number of substitutions per site (next to the branches). Evolutionary analyses were conducted in MEGA11.
