# Supplementary material for: Characterization of Host-Specific Genes from Pine- and Grass-Associated Species of the Fusarium fujikuroi Species Complex
Source: Pathogens. 2022 Jul 29;11(8):858. doi: 10.3390/pathogens11080858 (PMC9415769; doi:10.3390/pathogens11080858)
Supplement: Supplementary file 1 [file pathogens-11-00858-s001.zip › Supplemental Figures/Figure S10.pdf]

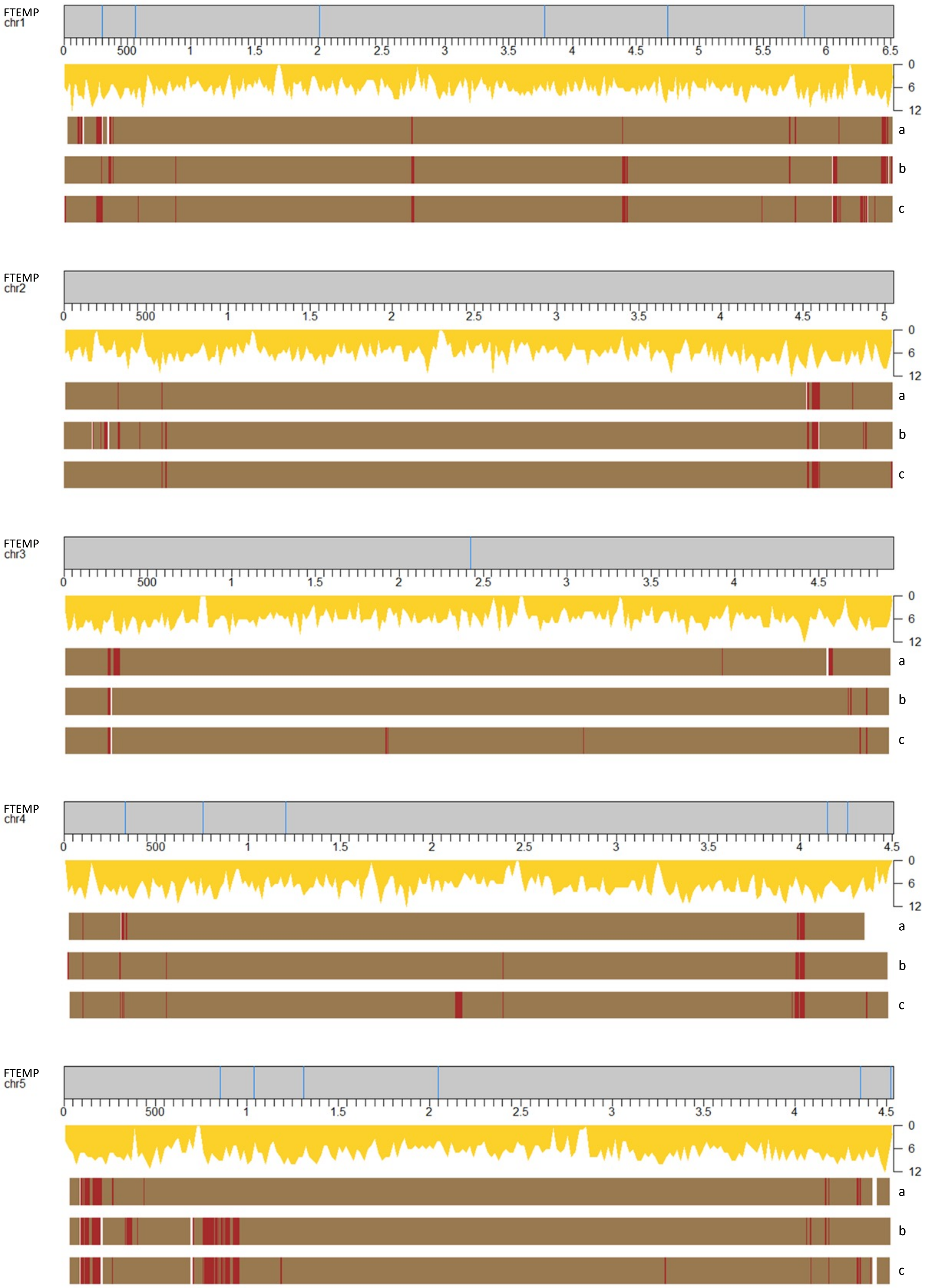
FTEMP
chr1
0 500 1 1.5 2 2.5 3 3.5 4 4.5 5 5.5 6 6.5
0 6 12
a
b
c
FTEMP
chr2
0 500 1 1.5 2 2.5 3 3.5 4 4.5 5
0 6 12
a
b
c
FTEMP
chr3
0 500 1 1.5 2 2.5 3 3.5 4 4.5
0 6 12
a
b
c
FTEMP
chr4
0 500 1 1.5 2 2.5 3 3.5 4 4.5
0 6 12
a
b
c
FTEMP
chr5
0 500 1 1.5 2 2.5 3 3.5 4 4.5
0 6 12
a
b
c

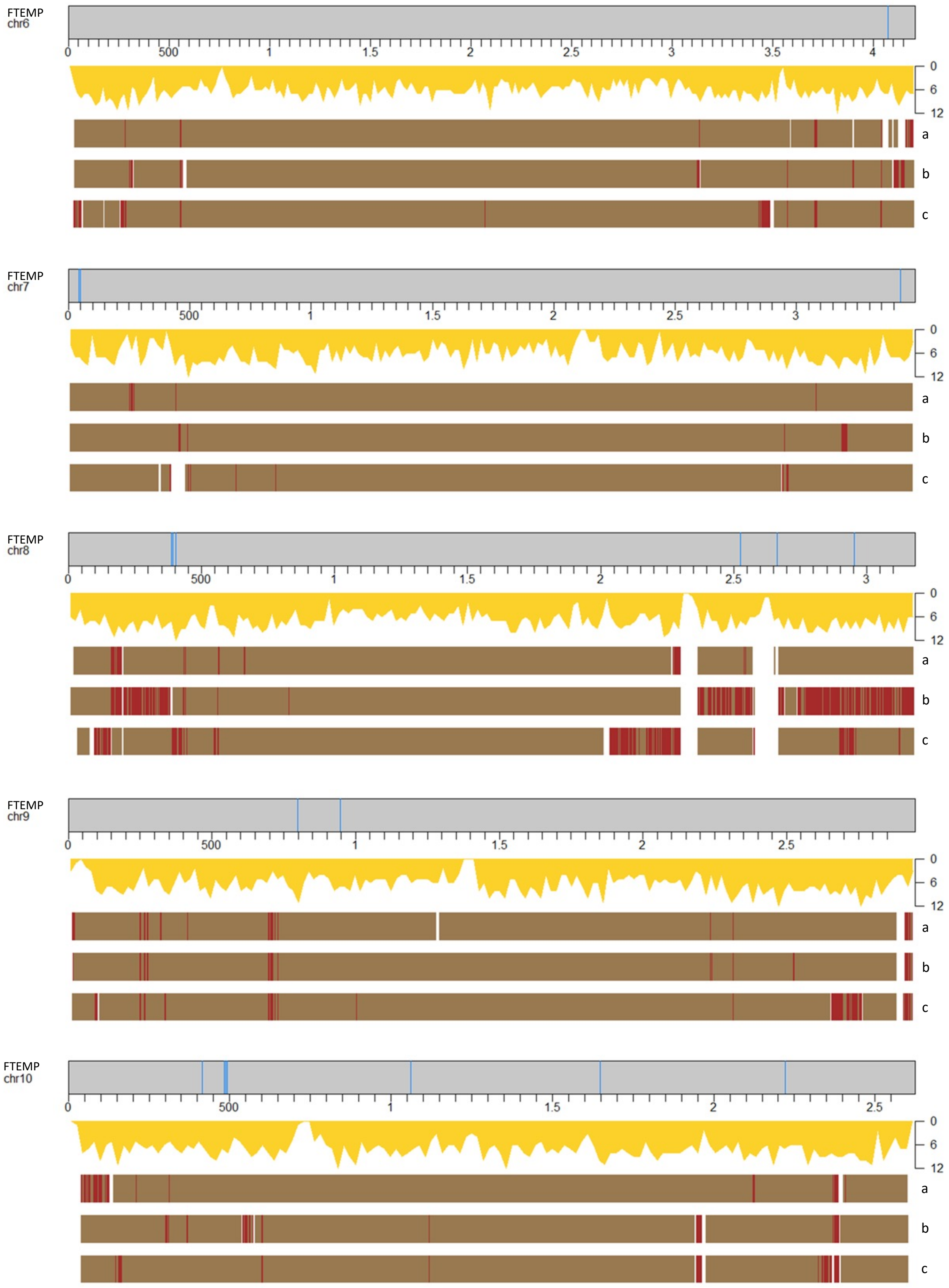
FTEMP
chr6
0
500
1
1.5
2
2.5
3
3.5
4
0
6
12
a
b
c
FTEMP
chr7
0
500
1
1.5
2
2.5
3
0
6
12
a
b
c
FTEMP
chr8
0
500
1
1.5
2
2.5
3
0
6
12
a
b
c
FTEMP
chr9
0
500
1
1.5
2
2.5
0
6
12
a
b
c
FTEMP
chr10
0
500
1
1.5
2
2.5
0
6
12
a
b
c

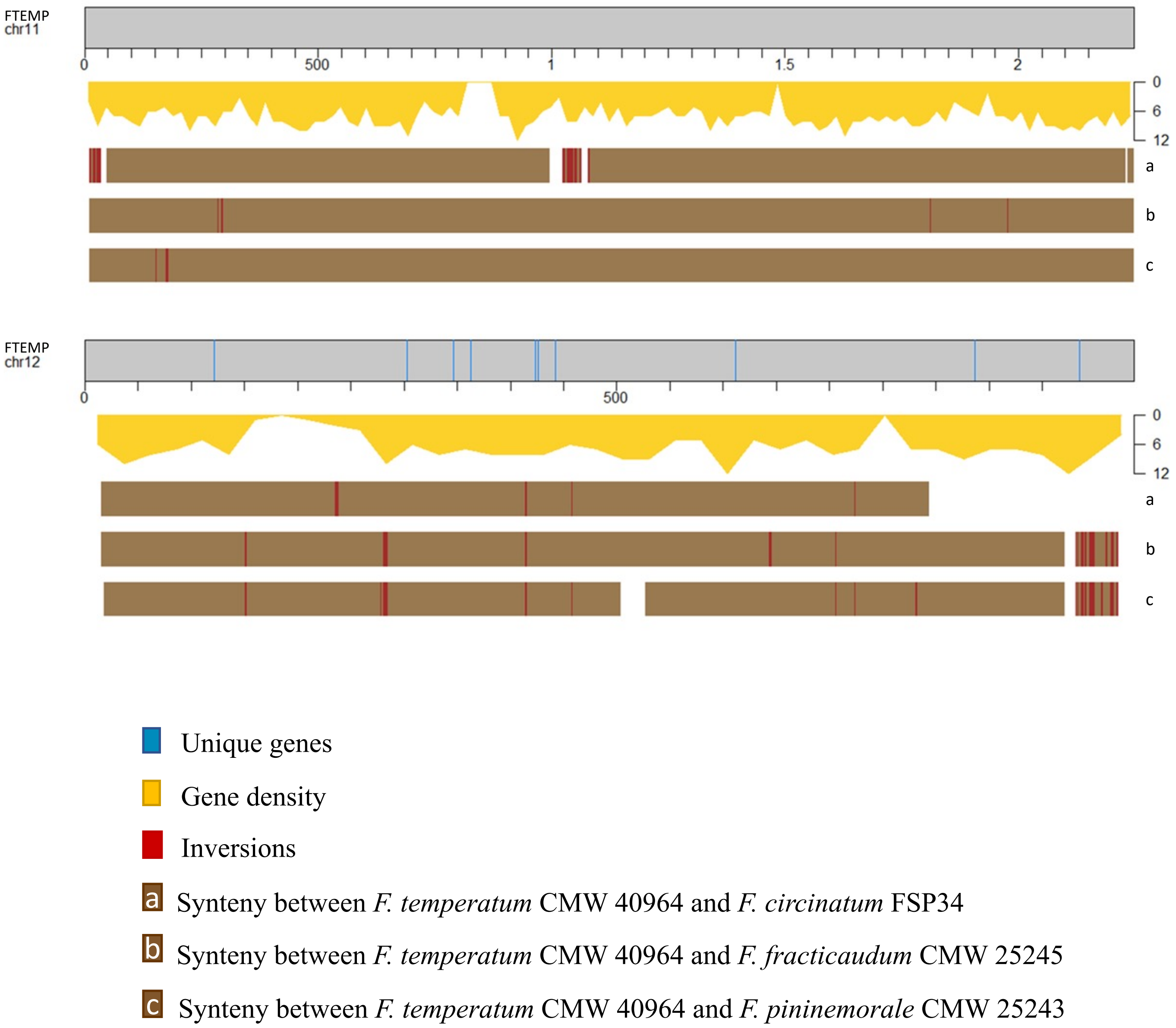

Figure S10. The distribution of Poaceae-host-associated genes and conservation of synteny across and between chromosomes and genomes. Poaceae-host-associated genes distribution across each of the chromosomes as indicated by the blue lines. The conservation of synteny and inversion between the relevant genomes are indicated in the brown blocks and red lines. FTEMP = *F. temperatum*; chromosome size is given in kbp.
