# Supplementary material for: Characterization of Host-Specific Genes from Pine- and Grass-Associated Species of the Fusarium fujikuroi Species Complex
Source: Pathogens. 2022 Jul 29;11(8):858. doi: 10.3390/pathogens11080858 (PMC9415769; doi:10.3390/pathogens11080858)
Supplement: Supplementary file 1 [file pathogens-11-00858-s001.zip › Supplemental Figures/Figure S2.pdf]

Figure S2. Host-range-associated genes with ancestral origins that emerged within the FFSC. Host-range-associated genes investigated are highlighted in yellow; FCIR = *Fusarium circinatum* and FTEMP= *Fusarium temperatum*.

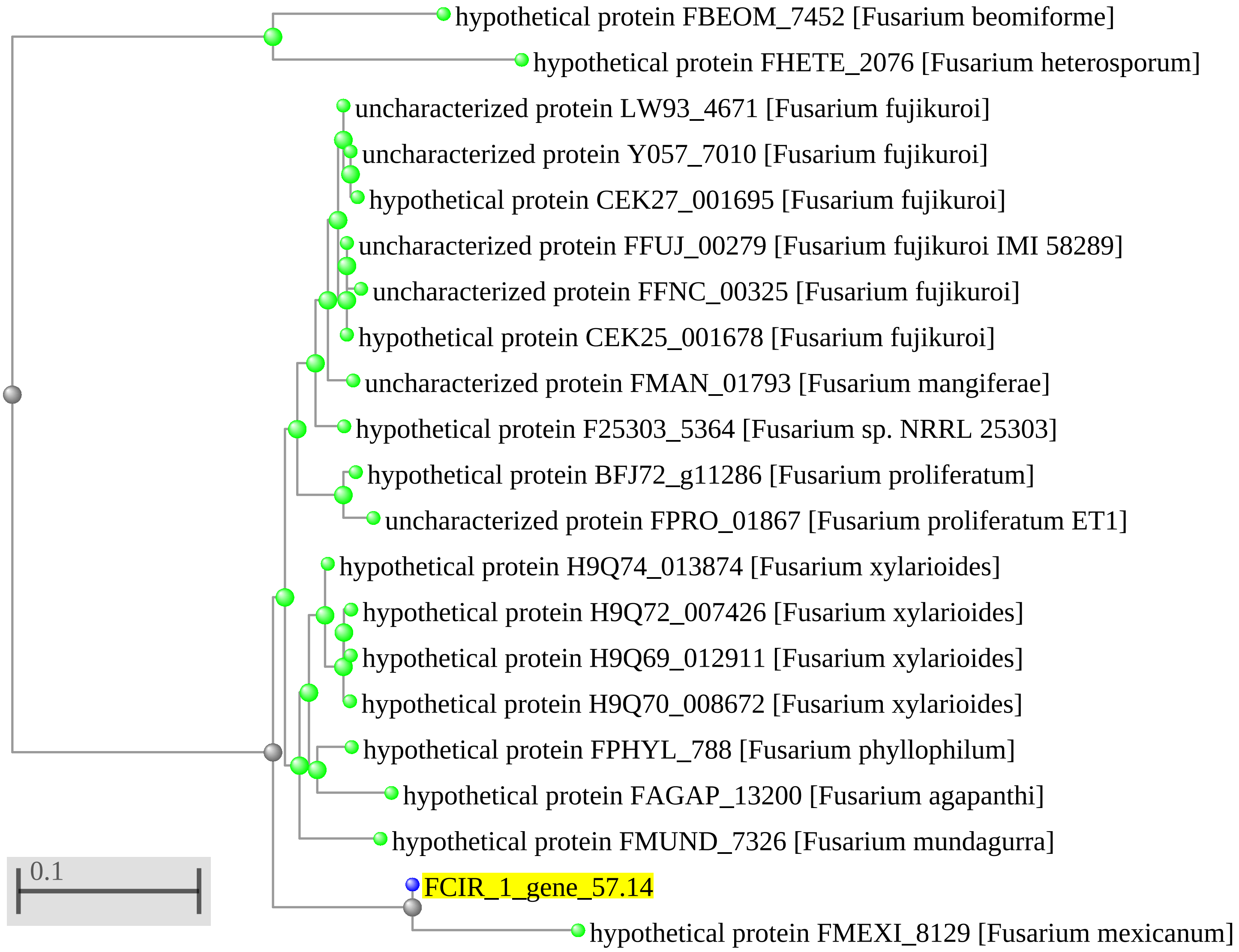
hypothetical protein FBEOM_7452 [Fusarium beomiforme]
hypothetical protein FHETE_2076 [Fusarium heterosporum]
uncharacterized protein LW93_4671 [Fusarium fujikuroi]
uncharacterized protein Y057_7010 [Fusarium fujikuroi]
hypothetical protein CEK27_001695 [Fusarium fujikuroi]
uncharacterized protein FFUJ_00279 [Fusarium fujikuroi IMI 58289]
uncharacterized protein FFNC_00325 [Fusarium fujikuroi]
hypothetical protein CEK25_001678 [Fusarium fujikuroi]
uncharacterized protein FMAN_01793 [Fusarium mangiferae]
hypothetical protein F25303_5364 [Fusarium sp. NRRL 25303]
hypothetical protein BFJ72_g11286 [Fusarium proliferatum]
uncharacterized protein FPRO_01867 [Fusarium proliferatum ET1]
hypothetical protein H9Q74_013874 [Fusarium xylarioides]
hypothetical protein H9Q72_007426 [Fusarium xylarioides]
hypothetical protein H9Q69_012911 [Fusarium xylarioides]
hypothetical protein H9Q70_008672 [Fusarium xylarioides]
hypothetical protein FPHYL_788 [Fusarium phyllophilum]
hypothetical protein FAGAP_13200 [Fusarium agapanthi]
hypothetical protein FMUND_7326 [Fusarium mundagurra]
FCIR_1_gene_57.14
hypothetical protein FMEXI_8129 [Fusarium mexicanum]
0.1

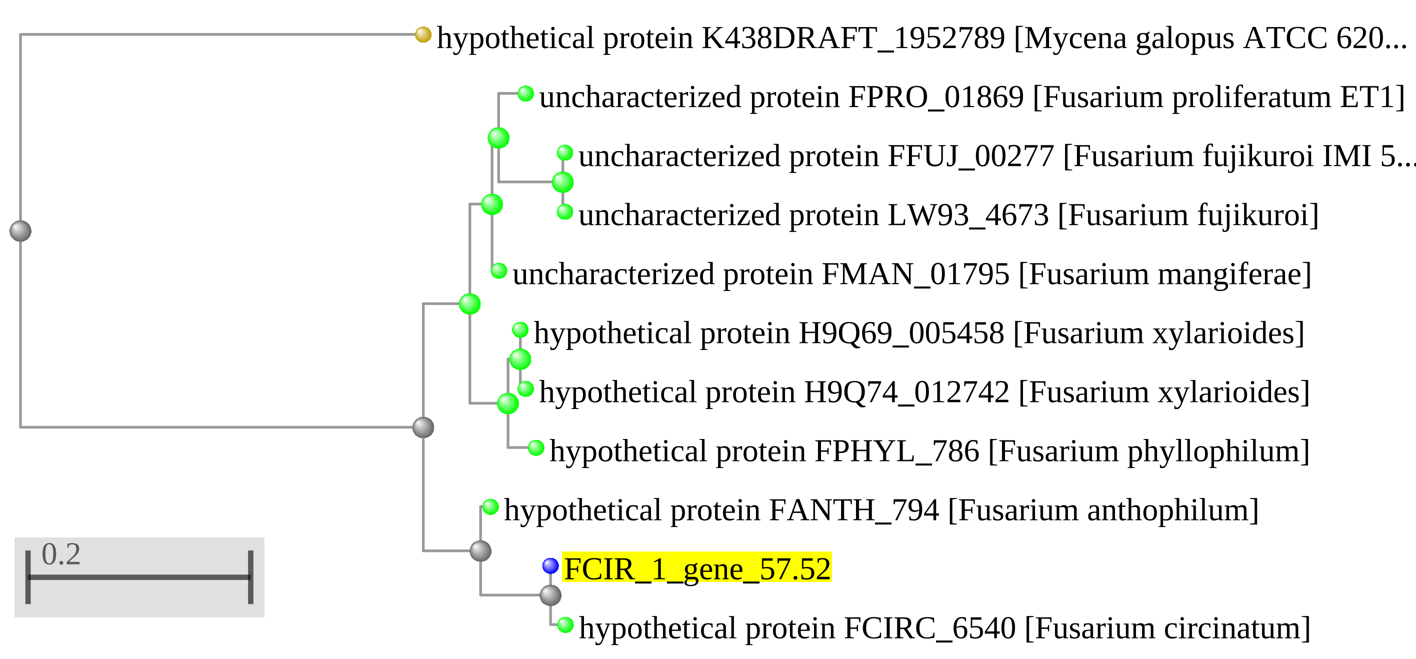
hypothetical protein K438DRAFT_1952789 [Mycena galopus ATCC 620...
uncharacterized protein FPRO_01869 [Fusarium proliferatum ET1]
uncharacterized protein FFUJ_00277 [Fusarium fujikuroi IMI 5...
uncharacterized protein LW93_4673 [Fusarium fujikuroi]
uncharacterized protein FMAN_01795 [Fusarium mangiferae]
hypothetical protein H9Q69_005458 [Fusarium xylarioides]
hypothetical protein H9Q74_012742 [Fusarium xylarioides]
hypothetical protein FPHYL_786 [Fusarium phyllophilum]
hypothetical protein FANTH_794 [Fusarium anthophilum]
FCIR_1_gene_57.52
hypothetical protein FCIRC_6540 [Fusarium circinatum]
0.2

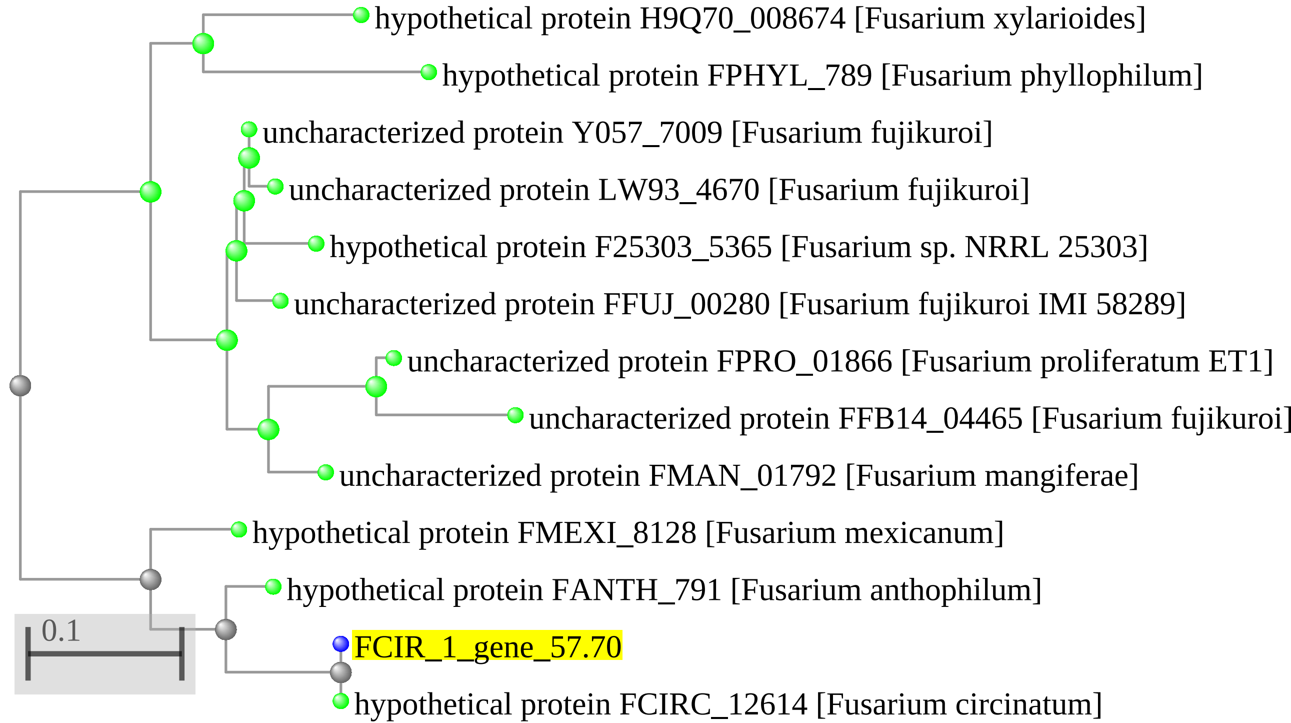
hypothetical protein H9Q70_008674 [Fusarium xylarioides]
hypothetical protein FPHYL_789 [Fusarium phyllophilum]
uncharacterized protein Y057_7009 [Fusarium fujikuroi]
uncharacterized protein LW93_4670 [Fusarium fujikuroi]
hypothetical protein F25303_5365 [Fusarium sp. NRRL 25303]
uncharacterized protein FFUJ_00280 [Fusarium fujikuroi IMI 58289]
uncharacterized protein FPRO_01866 [Fusarium proliferatum ET1]
uncharacterized protein FFB14_04465 [Fusarium fujikuroi]
uncharacterized protein FMAN_01792 [Fusarium mangiferae]
hypothetical protein FMEXI_8128 [Fusarium mexicanum]
hypothetical protein FANTH_791 [Fusarium anthophilum]
FCIR_1_gene_57.70
hypothetical protein FCIRC_12614 [Fusarium circinatum]
0.1

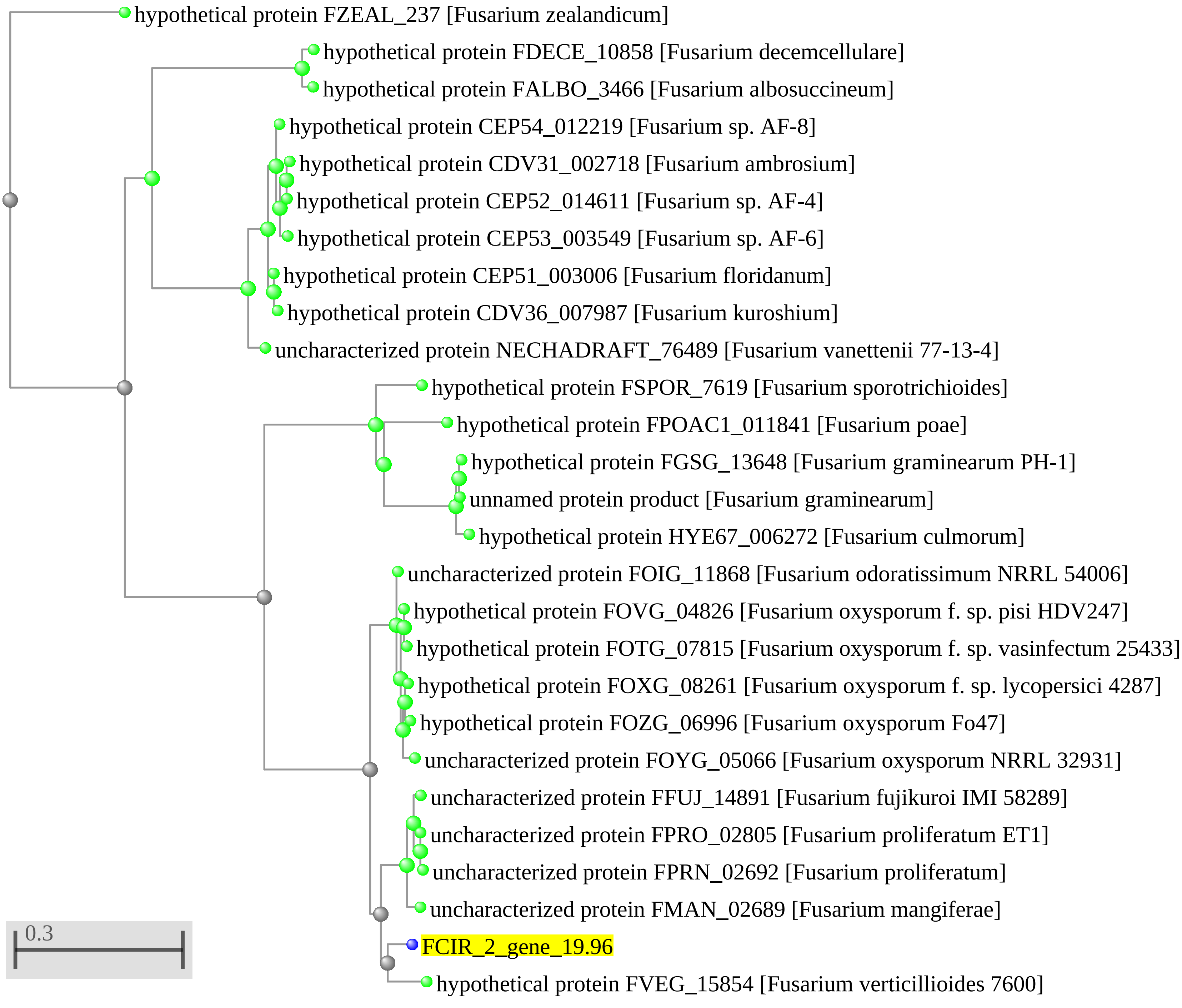
hypothetical protein FZEAL_237 [Fusarium zealandicum]
hypothetical protein FDECE_10858 [Fusarium decemcellulare]
hypothetical protein FALBO_3466 [Fusarium albosuccineum]
hypothetical protein CEP54_012219 [Fusarium sp. AF-8]
hypothetical protein CDV31_002718 [Fusarium ambrosium]
hypothetical protein CEP52_014611 [Fusarium sp. AF-4]
hypothetical protein CEP53_003549 [Fusarium sp. AF-6]
hypothetical protein CEP51_003006 [Fusarium floridanum]
hypothetical protein CDV36_007987 [Fusarium kuroshium]
uncharacterized protein NECHADRAFT_76489 [Fusarium vanettenii 77-13-4]
hypothetical protein FSPOR_7619 [Fusarium sporotrichioides]
hypothetical protein FPOAC1_011841 [Fusarium poae]
hypothetical protein FGSG_13648 [Fusarium graminearum PH-1]
unnamed protein product [Fusarium graminearum]
hypothetical protein HYE67_006272 [Fusarium culmorum]
uncharacterized protein FOIG_11868 [Fusarium odoratissimum NRRL 54006]
hypothetical protein FOVG_04826 [Fusarium oxysporum f. sp. pisi HDV247]
hypothetical protein FOTG_07815 [Fusarium oxysporum f. sp. vasinfectum 25433]
hypothetical protein FOXG_08261 [Fusarium oxysporum f. sp. lycopersici 4287]
hypothetical protein FOZG_06996 [Fusarium oxysporum Fo47]
uncharacterized protein FOYG_05066 [Fusarium oxysporum NRRL 32931]
uncharacterized protein FFUJ_14891 [Fusarium fujikuroi IMI 58289]
uncharacterized protein FPRO_02805 [Fusarium proliferatum ET1]
uncharacterized protein FPRN_02692 [Fusarium proliferatum]
uncharacterized protein FMAN_02689 [Fusarium mangiferae]
FCIR_2_gene_19.96
hypothetical protein FVEG_15854 [Fusarium verticillioides 7600]
0.3

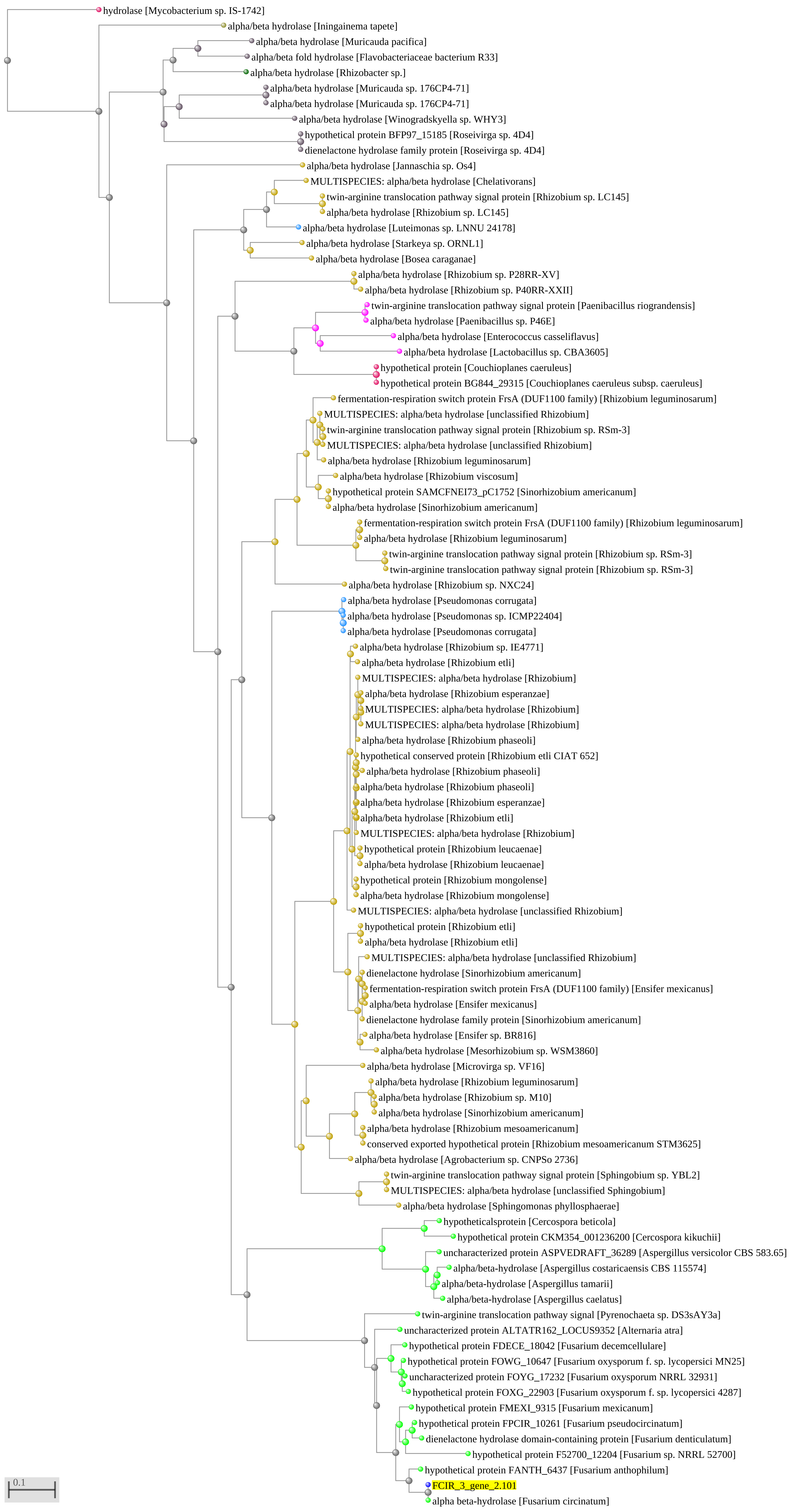
hydrolase [Mycobacterium sp. IS-1742]
alpha/beta hydrolase [Iningainema tapete]
alpha/beta hydrolase [Muricauda pacifica]
alpha/beta fold hydrolase [Flavobacteriaceae bacterium R33]
alpha/beta hydrolase [Rhizobacter sp.]
alpha/beta hydrolase [Muricauda sp. 176CP4-71]
alpha/beta hydrolase [Muricauda sp. 176CP4-71]
alpha/beta hydrolase [Winogradskyella sp. WHY3]
hypothetical protein BFP97_15185 [Roseivirga sp. 4D4]
dienelactone hydrolase family protein [Roseivirga sp. 4D4]
alpha/beta hydrolase [Jannaschia sp. Os4]
MULTISPECIES: alpha/beta hydrolase [Chelativorans]
twin-arginine translocation pathway signal protein [Rhizobium sp. LC145]
alpha/beta hydrolase [Rhizobium sp. LC145]
alpha/beta hydrolase [Luteimonas sp. LNNU 24178]
alpha/beta hydrolase [Starkeya sp. ORNL1]
alpha/beta hydrolase [Bosea caraganae]
alpha/beta hydrolase [Rhizobium sp. P28RR-XV]
alpha/beta hydrolase [Rhizobium sp. P40RR-XXII]
twin-arginine translocation pathway signal protein [Paenibacillus riograndensis]
alpha/beta hydrolase [Paenibacillus sp. P46E]
alpha/beta hydrolase [Enterococcus casseliflavus]
alpha/beta hydrolase [Lactobacillus sp. CBA3605]
hypothetical protein [Couchioplanes caeruleus]
hypothetical protein BG844_29315 [Couchioplanes caeruleus subsp. caeruleus]
fermentation-respiration switch protein FrsA (DUF1100 family) [Rhizobium leguminosarum]
MULTISPECIES: alpha/beta hydrolase [unclassified Rhizobium]
twin-arginine translocation pathway signal protein [Rhizobium sp. RSm-3]
MULTISPECIES: alpha/beta hydrolase [unclassified Rhizobium]
alpha/beta hydrolase [Rhizobium leguminosarum]
alpha/beta hydrolase [Rhizobium viscosum]
hypothetical protein SAMCFNEI73_pC1752 [Sinorhizobium americanum]
alpha/beta hydrolase [Sinorhizobium americanum]
fermentation-respiration switch protein FrsA (DUF1100 family) [Rhizobium leguminosarum]
alpha/beta hydrolase [Rhizobium leguminosarum]
twin-arginine translocation pathway signal protein [Rhizobium sp. RSm-3]
twin-arginine translocation pathway signal protein [Rhizobium sp. RSm-3]
alpha/beta hydrolase [Rhizobium sp. NXC24]
alpha/beta hydrolase [Pseudomonas corrugata]
alpha/beta hydrolase [Pseudomonas sp. ICMP22404]
alpha/beta hydrolase [Pseudomonas corrugata]
alpha/beta hydrolase [Rhizobium sp. IE4771]
alpha/beta hydrolase [Rhizobium etli]
MULTISPECIES: alpha/beta hydrolase [Rhizobium]
alpha/beta hydrolase [Rhizobium esperanzae]
MULTISPECIES: alpha/beta hydrolase [Rhizobium]
MULTISPECIES: alpha/beta hydrolase [Rhizobium]
alpha/beta hydrolase [Rhizobium phaseoli]
hypothetical conserved protein [Rhizobium etli CIAT 652]
alpha/beta hydrolase [Rhizobium phaseoli]
alpha/beta hydrolase [Rhizobium phaseoli]
alpha/beta hydrolase [Rhizobium esperanzae]
alpha/beta hydrolase [Rhizobium etli]
MULTISPECIES: alpha/beta hydrolase [Rhizobium]
hypothetical protein [Rhizobium leucaenae]
alpha/beta hydrolase [Rhizobium leucaenae]
hypothetical protein [Rhizobium mongolense]
alpha/beta hydrolase [Rhizobium mongolense]
MULTISPECIES: alpha/beta hydrolase [unclassified Rhizobium]
hypothetical protein [Rhizobium etli]
alpha/beta hydrolase [Rhizobium etli]
MULTISPECIES: alpha/beta hydrolase [unclassified Rhizobium]
dienelactone hydrolase [Sinorhizobium americanum]
fermentation-respiration switch protein FrsA (DUF1100 family) [Ensifer mexicanus]
alpha/beta hydrolase [Ensifer mexicanus]
dienelactone hydrolase family protein [Sinorhizobium americanum]
alpha/beta hydrolase [Ensifer sp. BR816]
alpha/beta hydrolase [Mesorhizobium sp. WSM3860]
alpha/beta hydrolase [Microvirga sp. VF16]
alpha/beta hydrolase [Rhizobium leguminosarum]
alpha/beta hydrolase [Rhizobium sp. M10]
alpha/beta hydrolase [Sinorhizobium americanum]
alpha/beta hydrolase [Rhizobium mesoamericanum]
conserved exported hypothetical protein [Rhizobium mesoamericanum STM3625]
alpha/beta hydrolase [Agrobacterium sp. CNPSo 2736]
twin-arginine translocation pathway signal protein [Sphingobium sp. YBL2]
MULTISPECIES: alpha/beta hydrolase [unclassified Sphingobium]
alpha/beta hydrolase [Sphingomonas phyllosphaerae]
hypotheticalsprotein [Cercospora beticola]
hypothetical protein CKM354_001236200 [Cercospora kikuchii]
uncharacterized protein ASPVEDRAFT_36289 [Aspergillus versicolor CBS 583.65]
alpha/beta-hydrolase [Aspergillus costaricaensis CBS 115574]
alpha/beta-hydrolase [Aspergillus tamarii]
alpha/beta-hydrolase [Aspergillus caelatus]
twin-arginine translocation pathway signal [Pyrenochaeta sp. DS3sAY3a]
uncharacterized protein ALTATR162_LOCUS9352 [Alternaria atra]
hypothetical protein FDECE_18042 [Fusarium decemcellulare]
hypothetical protein FOWG_10647 [Fusarium oxysporum f. sp. lycopersici MN25]
uncharacterized protein FOYG_17232 [Fusarium oxysporum NRRL 32931]
hypothetical protein FOXG_22903 [Fusarium oxysporum f. sp. lycopersici 4287]
hypothetical protein FMEXI_9315 [Fusarium mexicanum]
hypothetical protein FPCIR_10261 [Fusarium pseudocircinatum]
dienelactone hydrolase domain-containing protein [Fusarium denticulatum]
hypothetical protein F52700_12204 [Fusarium sp. NRRL 52700]
hypothetical protein FANTH_6437 [Fusarium anthophilum]
FCIR_3_gene_2.101
alpha beta-hydrolase [Fusarium circinatum]
0.1

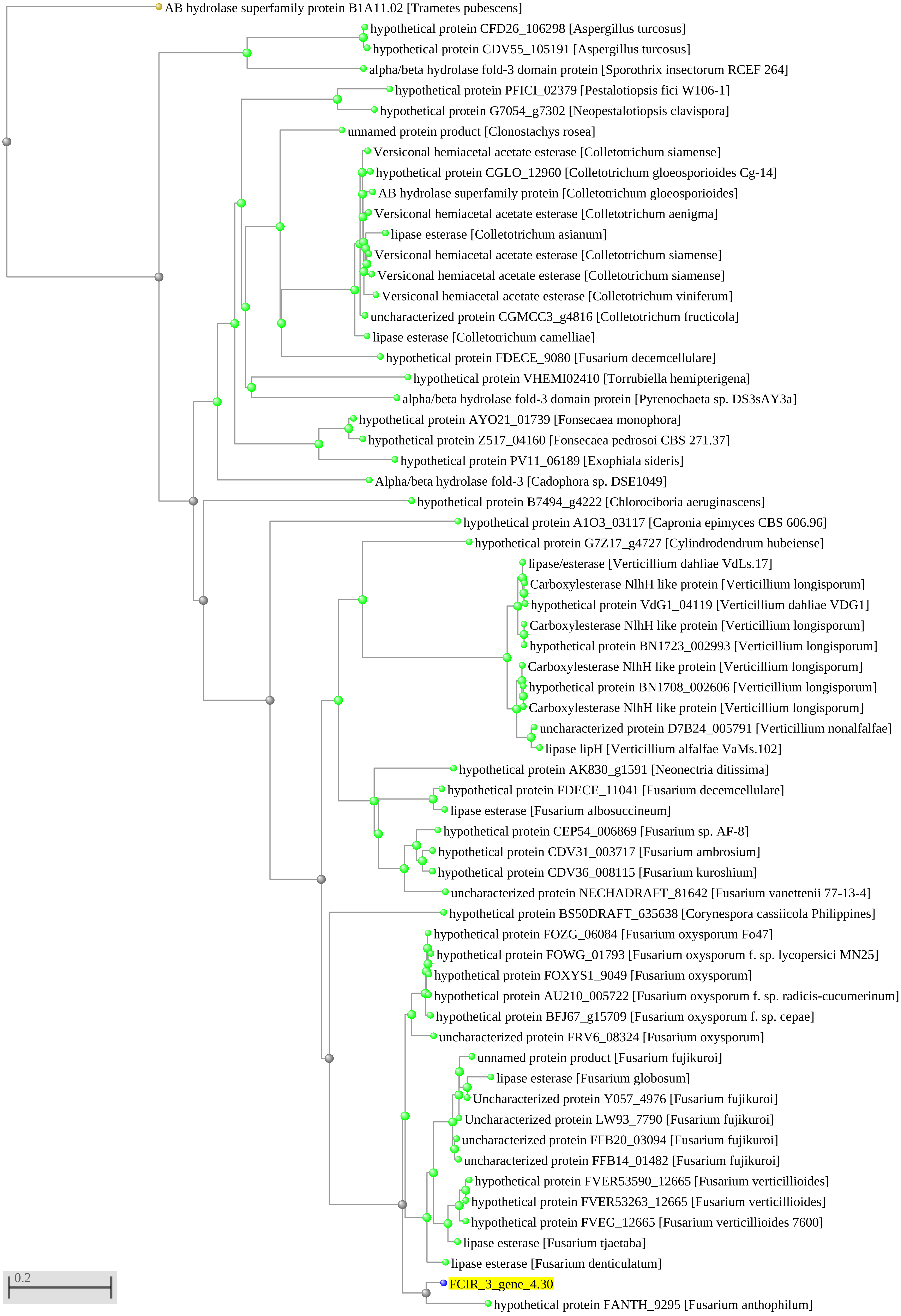

AB hydrolase superfamily protein B1A11.02 [Trametes pubescens]
hypothetical protein CFD26_106298 [Aspergillus turcosus]
hypothetical protein CDV55_105191 [Aspergillus turcosus]
alpha/beta hydrolase fold-3 domain protein [Sporothrix insectorum RCEF 264]
hypothetical protein PFICI_02379 [Pestalotiopsis fici W106-1]
hypothetical protein G7054_g7302 [Neopestalotiopsis clavispora]
unnamed protein product [Clonostachys rosea]
Versiconal hemiacetal acetate esterase [Colletotrichum siamense]
hypothetical protein CGLO_12960 [Colletotrichum gloeosporioides Cg-14]
AB hydrolase superfamily protein [Colletotrichum gloeosporioides]
Versiconal hemiacetal acetate esterase [Colletotrichum aenigma]
lipase esterase [Colletotrichum asianum]
Versiconal hemiacetal acetate esterase [Colletotrichum siamense]
Versiconal hemiacetal acetate esterase [Colletotrichum siamense]
Versiconal hemiacetal acetate esterase [Colletotrichum viniferum]
uncharacterized protein CGMCC3_g4816 [Colletotrichum fructicola]
lipase esterase [Colletotrichum camelliae]
hypothetical protein FDECE_9080 [Fusarium decemcellulare]
hypothetical protein VHEMI02410 [Torrubiella hemipterigena]
alpha/beta hydrolase fold-3 domain protein [Pyrenochaeta sp. DS3sAY3a]
hypothetical protein AYO21_01739 [Fonsecaea monophora]
hypothetical protein Z517_04160 [Fonsecaea pedrosoi CBS 271.37]
hypothetical protein PV11_06189 [Exophiala sideris]
Alpha/beta hydrolase fold-3 [Cadophora sp. DSE1049]
hypothetical protein B7494_g4222 [Chlorociboria aeruginascens]
hypothetical protein A1O3_03117 [Capronia epimyces CBS 606.96]
hypothetical protein G7Z17_g4727 [Cylindrodendrum hubeiense]
lipase/esterase [Verticillium dahliae VdLs.17]
Carboxylesterase NlhH like protein [Verticillium longisporum]
hypothetical protein VdG1_04119 [Verticillium dahliae VDG1]
Carboxylesterase NlhH like protein [Verticillium longisporum]
hypothetical protein BN1723_002993 [Verticillium longisporum]
Carboxylesterase NlhH like protein [Verticillium longisporum]
hypothetical protein BN1708_002606 [Verticillium longisporum]
Carboxylesterase NlhH like protein [Verticillium longisporum]
uncharacterized protein D7B24_005791 [Verticillium nonalfalfae]
lipase lipH [Verticillium alfalfae VaMs.102]
hypothetical protein AK830_g1591 [Neonectria ditissima]
hypothetical protein FDECE_11041 [Fusarium decemcellulare]
lipase esterase [Fusarium albosuccineum]
hypothetical protein CEP54_006869 [Fusarium sp. AF-8]
hypothetical protein CDV31_003717 [Fusarium ambrosium]
hypothetical protein CDV36_008115 [Fusarium kuroshium]
uncharacterized protein NECHADRAFT_81642 [Fusarium vanettenii 77-13-4]
hypothetical protein BS50DRAFT_635638 [Corynespora cassiicola Philippines]
hypothetical protein FOZG_06084 [Fusarium oxysporum Fo47]
hypothetical protein FOWG_01793 [Fusarium oxysporum f. sp. lycopersici MN25]
hypothetical protein FOXYS1_9049 [Fusarium oxysporum]
hypothetical protein AU210_005722 [Fusarium oxysporum f. sp. radicis-cucumerinum]
hypothetical protein BFJ67_g15709 [Fusarium oxysporum f. sp. cepae]
uncharacterized protein FRV6_08324 [Fusarium oxysporum]
unnamed protein product [Fusarium fujikuroi]
lipase esterase [Fusarium globosum]
Uncharacterized protein Y057_4976 [Fusarium fujikuroi]
Uncharacterized protein LW93_7790 [Fusarium fujikuroi]
uncharacterized protein FFB20_03094 [Fusarium fujikuroi]
uncharacterized protein FFB14_01482 [Fusarium fujikuroi]
hypothetical protein FVER53590_12665 [Fusarium verticillioides]
hypothetical protein FVER53263_12665 [Fusarium verticillioides]
hypothetical protein FVEG_12665 [Fusarium verticillioides 7600]
lipase esterase [Fusarium tjaetaba]
lipase esterase [Fusarium denticulatum]
FCIR_3_gene_4.30
hypothetical protein FANTH_9295 [Fusarium anthophilum]
0.2

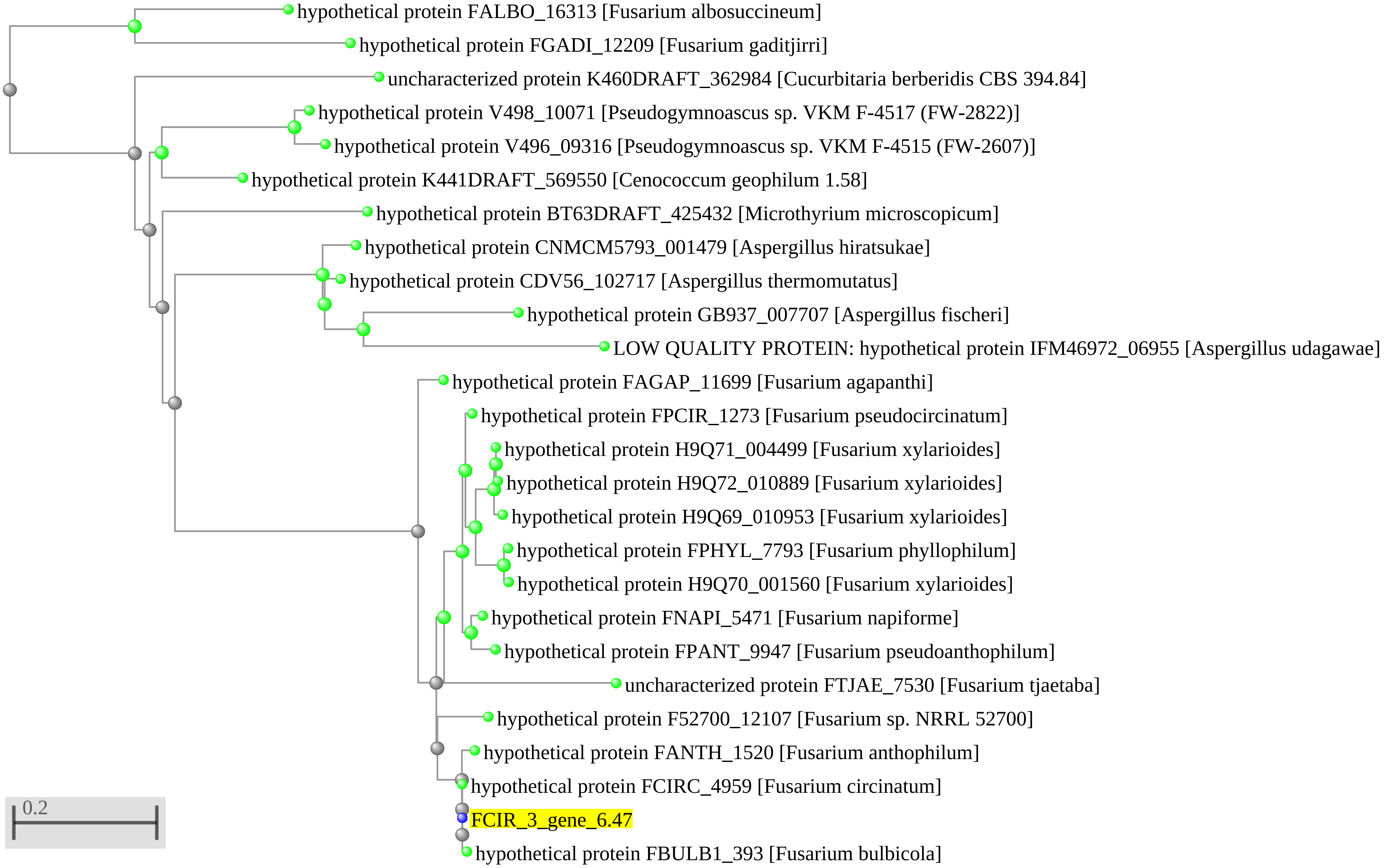
hypothetical protein FALBO_16313 [Fusarium albosuccineum]
hypothetical protein FGADI_12209 [Fusarium gaditjirri]
uncharacterized protein K460DRAFT_362984 [Cucurbitaria berberidis CBS 394.84]
hypothetical protein V498_10071 [Pseudogymnoascus sp. VKM F-4517 (FW-2822)]
hypothetical protein V496_09316 [Pseudogymnoascus sp. VKM F-4515 (FW-2607)]
hypothetical protein K441DRAFT_569550 [Cenococcum geophilum 1.58]
hypothetical protein BT63DRAFT_425432 [Microthyrium microscopicum]
hypothetical protein CNMCM5793_001479 [Aspergillus hiratsukae]
hypothetical protein CDV56_102717 [Aspergillus thermomutatus]
hypothetical protein GB937_007707 [Aspergillus fischeri]
LOW QUALITY PROTEIN: hypothetical protein IFM46972_06955 [Aspergillus udagawae]
hypothetical protein FAGAP_11699 [Fusarium agapanthi]
hypothetical protein FPCIR_1273 [Fusarium pseudocircinatum]
hypothetical protein H9Q71_004499 [Fusarium xylarioides]
hypothetical protein H9Q72_010889 [Fusarium xylarioides]
hypothetical protein H9Q69_010953 [Fusarium xylarioides]
hypothetical protein FPHYL_7793 [Fusarium phyllophilum]
hypothetical protein H9Q70_001560 [Fusarium xylarioides]
hypothetical protein FNAPI_5471 [Fusarium napiforme]
hypothetical protein FPANT_9947 [Fusarium pseudoanthophilum]
uncharacterized protein FTJAE_7530 [Fusarium tjaetaba]
hypothetical protein F52700_12107 [Fusarium sp. NRRL 52700]
hypothetical protein FANTH_1520 [Fusarium anthophilum]
hypothetical protein FCIRC_4959 [Fusarium circinatum]
FCIR_3_gene_6.47
hypothetical protein FBULB1_393 [Fusarium bulbicola]
0.2

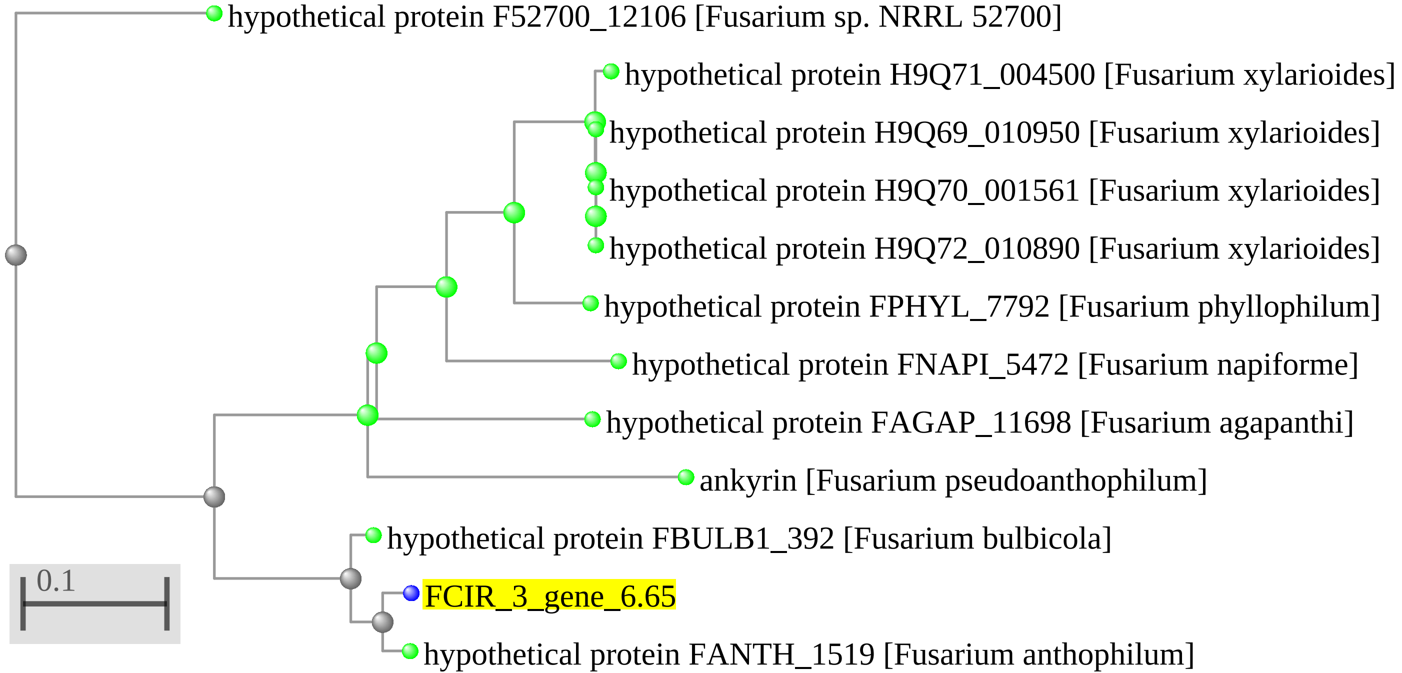
hypothetical protein F52700_12106 [Fusarium sp. NRRL 52700]
hypothetical protein H9Q71_004500 [Fusarium xylarioides]
hypothetical protein H9Q69_010950 [Fusarium xylarioides]
hypothetical protein H9Q70_001561 [Fusarium xylarioides]
hypothetical protein H9Q72_010890 [Fusarium xylarioides]
hypothetical protein FPHYL_7792 [Fusarium phyllophilum]
hypothetical protein FNAPI_5472 [Fusarium napiforme]
hypothetical protein FAGAP_11698 [Fusarium agapanthi]
ankyrin [Fusarium pseudoanthophilum]
hypothetical protein FBULB1_392 [Fusarium bulbicola]
FCIR_3_gene_6.65
hypothetical protein FANTH_1519 [Fusarium anthophilum]
0.1

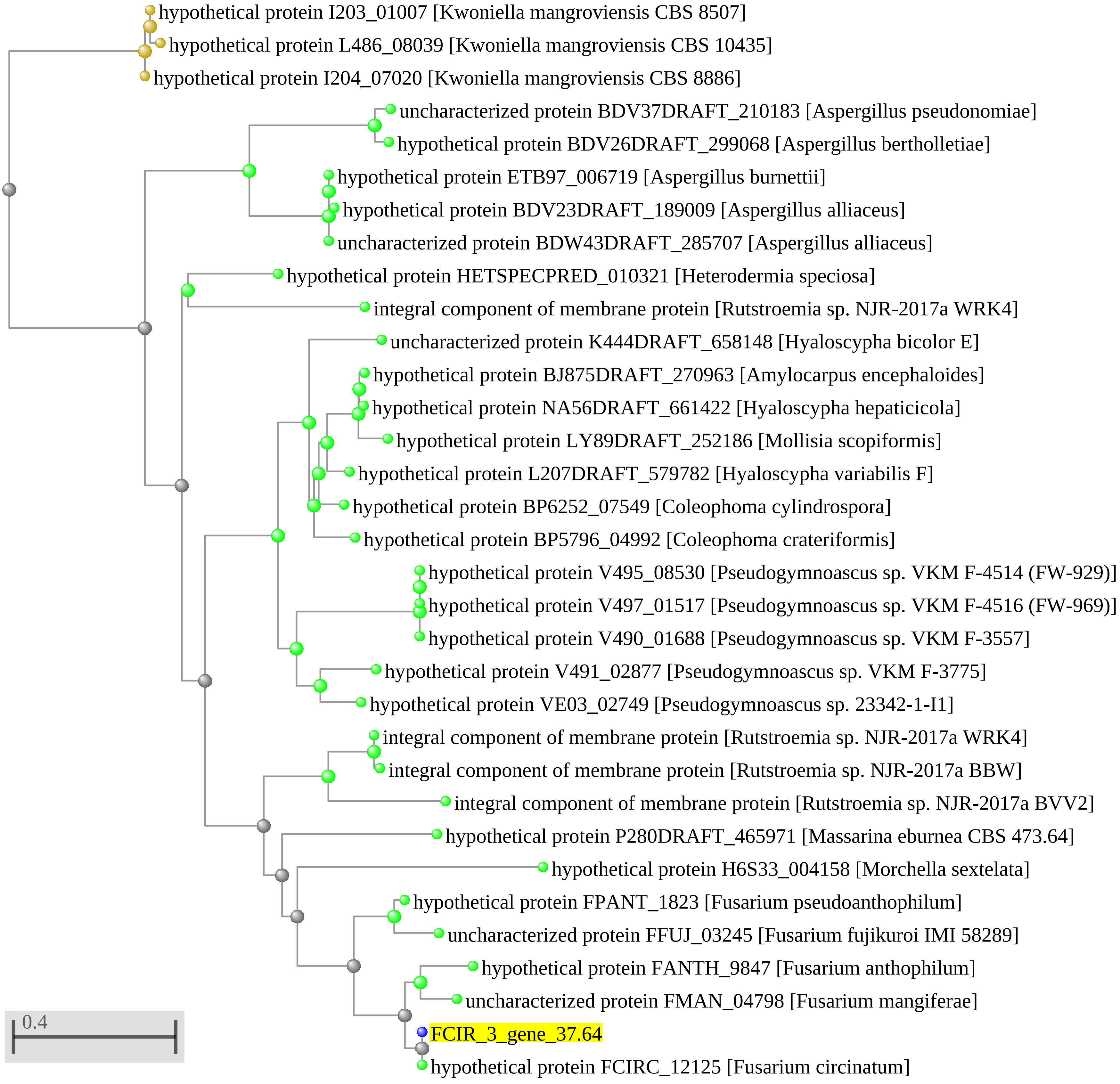
hypothetical protein I203_01007 [Kwoniella mangroviensis CBS 8507]
hypothetical protein L486_08039 [Kwoniella mangroviensis CBS 10435]
hypothetical protein I204_07020 [Kwoniella mangroviensis CBS 8886]
uncharacterized protein BDV37DRAFT_210183 [Aspergillus pseudonomiae]
hypothetical protein BDV26DRAFT_299068 [Aspergillus bertholletiae]
hypothetical protein ETB97_006719 [Aspergillus burnettii]
hypothetical protein BDV23DRAFT_189009 [Aspergillus alliaceus]
uncharacterized protein BDW43DRAFT_285707 [Aspergillus alliaceus]
hypothetical protein HETSPECPRED_010321 [Heterodermia speciosa]
integral component of membrane protein [Rutstroemia sp. NJR-2017a WRK4]
uncharacterized protein K444DRAFT_658148 [Hyaloscypha bicolor E]
hypothetical protein BJ875DRAFT_270963 [Amylocarpus encephaloides]
hypothetical protein NA56DRAFT_661422 [Hyaloscypha hepaticicola]
hypothetical protein LY89DRAFT_252186 [Mollisia scopiformis]
hypothetical protein L207DRAFT_579782 [Hyaloscypha variabilis F]
hypothetical protein BP6252_07549 [Coleophoma cylindrospora]
hypothetical protein BP5796_04992 [Coleophoma crateriformis]
hypothetical protein V495_08530 [Pseudogymnoascus sp. VKM F-4514 (FW-929)]
hypothetical protein V497_01517 [Pseudogymnoascus sp. VKM F-4516 (FW-969)]
hypothetical protein V490_01688 [Pseudogymnoascus sp. VKM F-3557]
hypothetical protein V491_02877 [Pseudogymnoascus sp. VKM F-3775]
hypothetical protein VE03_02749 [Pseudogymnoascus sp. 23342-1-I1]
integral component of membrane protein [Rutstroemia sp. NJR-2017a WRK4]
integral component of membrane protein [Rutstroemia sp. NJR-2017a BBW]
integral component of membrane protein [Rutstroemia sp. NJR-2017a BVV2]
hypothetical protein P280DRAFT_465971 [Massarina eburnea CBS 473.64]
hypothetical protein H6S33_004158 [Morchella sextelata]
hypothetical protein FPANT_1823 [Fusarium pseudoanthophilum]
uncharacterized protein FFUJ_03245 [Fusarium fujikuroi IMI 58289]
hypothetical protein FANTH_9847 [Fusarium anthophilum]
uncharacterized protein FMAN_04798 [Fusarium mangiferae]
FCIR_3_gene_37.64
hypothetical protein FCIRC_12125 [Fusarium circinatum]
0.4

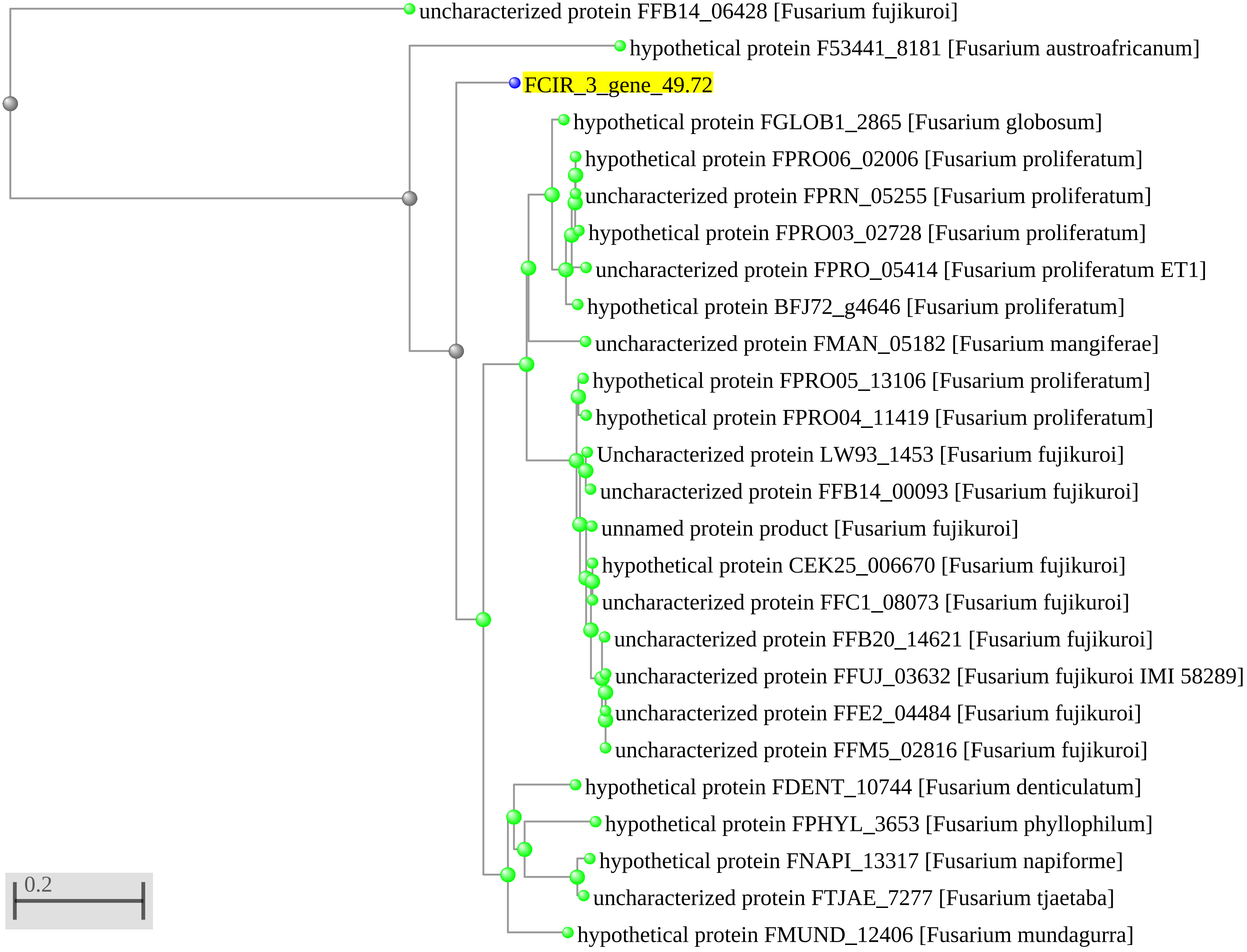
uncharacterized protein FFB14_06428 [Fusarium fujikuroi]
hypothetical protein F53441_8181 [Fusarium austroafricanum]
FCIR_3_gene_49.72
hypothetical protein FGLOB1_2865 [Fusarium globosum]
hypothetical protein FPRO06_02006 [Fusarium proliferatum]
uncharacterized protein FPRN_05255 [Fusarium proliferatum]
hypothetical protein FPRO03_02728 [Fusarium proliferatum]
uncharacterized protein FPRO_05414 [Fusarium proliferatum ET1]
hypothetical protein BFJ72_g4646 [Fusarium proliferatum]
uncharacterized protein FMAN_05182 [Fusarium mangiferae]
hypothetical protein FPRO05_13106 [Fusarium proliferatum]
hypothetical protein FPRO04_11419 [Fusarium proliferatum]
Uncharacterized protein LW93_1453 [Fusarium fujikuroi]
uncharacterized protein FFB14_00093 [Fusarium fujikuroi]
unnamed protein product [Fusarium fujikuroi]
hypothetical protein CEK25_006670 [Fusarium fujikuroi]
uncharacterized protein FFC1_08073 [Fusarium fujikuroi]
uncharacterized protein FFB20_14621 [Fusarium fujikuroi]
uncharacterized protein FFUJ_03632 [Fusarium fujikuroi IMI 58289]
uncharacterized protein FFE2_04484 [Fusarium fujikuroi]
uncharacterized protein FFM5_02816 [Fusarium fujikuroi]
hypothetical protein FDENT_10744 [Fusarium denticulatum]
hypothetical protein FPHYL_3653 [Fusarium phyllophilum]
hypothetical protein FNAPI_13317 [Fusarium napiforme]
uncharacterized protein FTJAE_7277 [Fusarium tjaetaba]
hypothetical protein FMUND_12406 [Fusarium mundagurra]
0.2

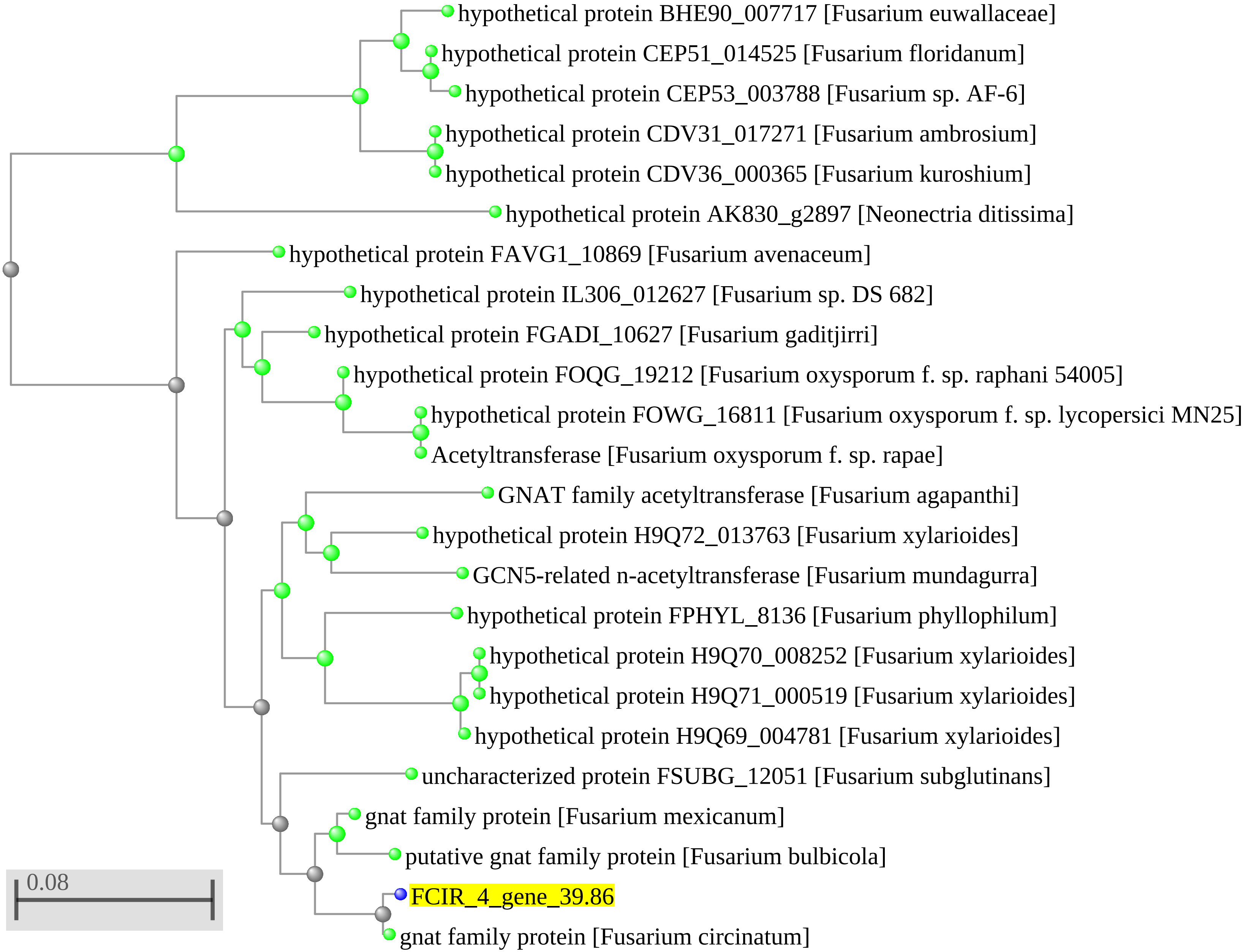

hypothetical protein BHE90_007717 [Fusarium euwallaceae]
hypothetical protein CEP51_014525 [Fusarium floridanum]
hypothetical protein CEP53_003788 [Fusarium sp. AF-6]
hypothetical protein CDV31_017271 [Fusarium ambrosium]
hypothetical protein CDV36_000365 [Fusarium kuroshium]
hypothetical protein AK830_g2897 [Neonectria ditissima]
hypothetical protein FAVG1_10869 [Fusarium avenaceum]
hypothetical protein IL306_012627 [Fusarium sp. DS 682]
hypothetical protein FGADI_10627 [Fusarium gaditjirri]
hypothetical protein FOQG_19212 [Fusarium oxysporum f. sp. raphani 54005]
hypothetical protein FOWG_16811 [Fusarium oxysporum f. sp. lycopersici MN25]
Acetyltransferase [Fusarium oxysporum f. sp. rapae]
GNAT family acetyltransferase [Fusarium agapanthi]
hypothetical protein H9Q72_013763 [Fusarium xylarioides]
GCN5-related n-acetyltransferase [Fusarium mundagurra]
hypothetical protein FPHYL_8136 [Fusarium phyllophilum]
hypothetical protein H9Q70_008252 [Fusarium xylarioides]
hypothetical protein H9Q71_000519 [Fusarium xylarioides]
hypothetical protein H9Q69_004781 [Fusarium xylarioides]
uncharacterized protein FSUBG_12051 [Fusarium subglutinans]
gnat family protein [Fusarium mexicanum]
putative gnat family protein [Fusarium bulbicola]
FCIR_4_gene_39.86
gnat family protein [Fusarium circinatum]
0.08

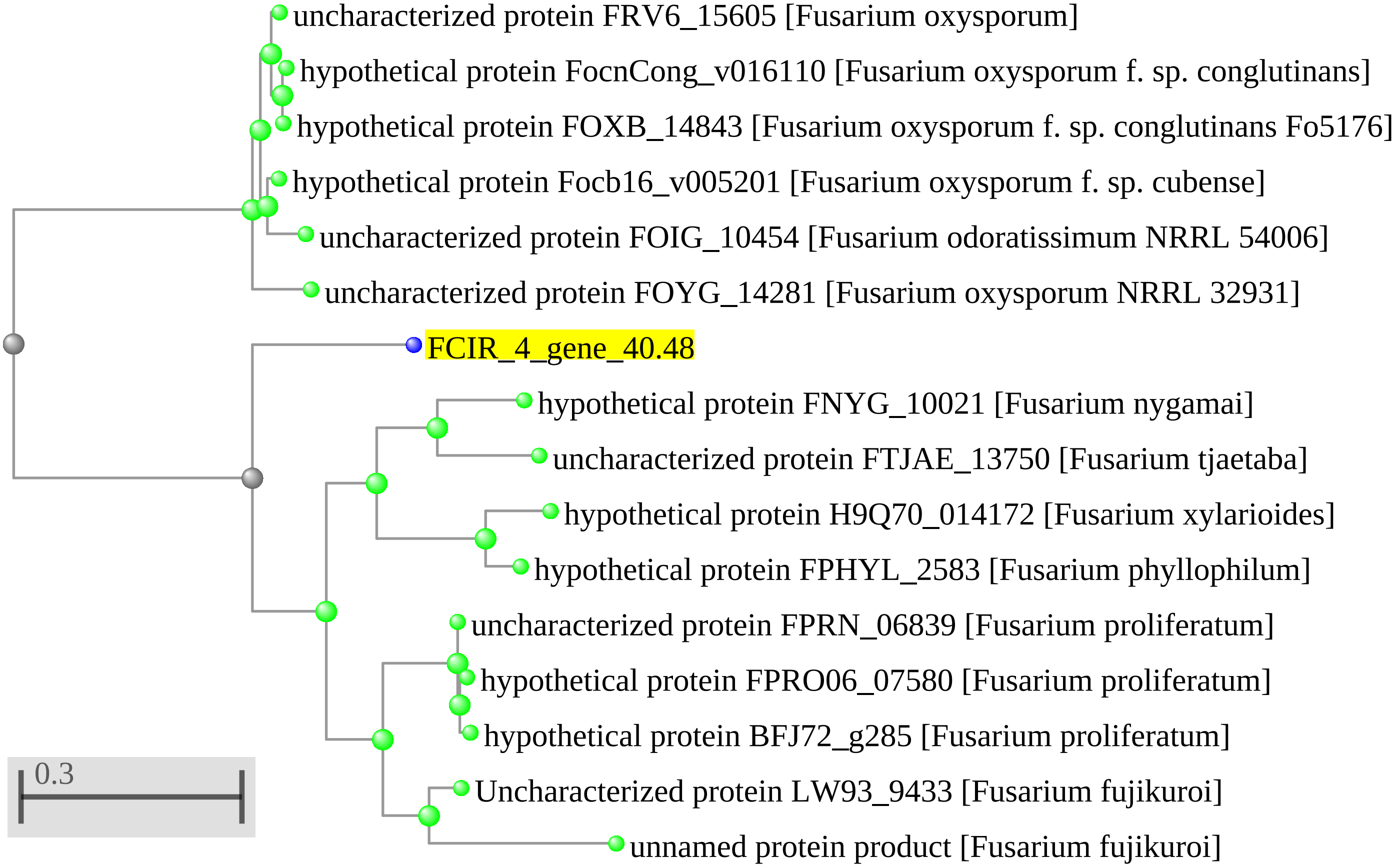
uncharacterized protein FRV6_15605 [Fusarium oxysporum]
hypothetical protein FocnCong_v016110 [Fusarium oxysporum f. sp. conglutinans]
hypothetical protein FOXB_14843 [Fusarium oxysporum f. sp. conglutinans Fo5176]
hypothetical protein Focb16_v005201 [Fusarium oxysporum f. sp. cubense]
uncharacterized protein FOIG_10454 [Fusarium odoratissimum NRRL 54006]
uncharacterized protein FOYG_14281 [Fusarium oxysporum NRRL 32931]
FCIR_4_gene_40.48
hypothetical protein FNYG_10021 [Fusarium nygamai]
uncharacterized protein FTJAE_13750 [Fusarium tjaetaba]
hypothetical protein H9Q70_014172 [Fusarium xylarioides]
hypothetical protein FPHYL_2583 [Fusarium phyllophilum]
uncharacterized protein FPRN_06839 [Fusarium proliferatum]
hypothetical protein FPRO06_07580 [Fusarium proliferatum]
hypothetical protein BFJ72_g285 [Fusarium proliferatum]
Uncharacterized protein LW93_9433 [Fusarium fujikuroi]
unnamed protein product [Fusarium fujikuroi]
0.3

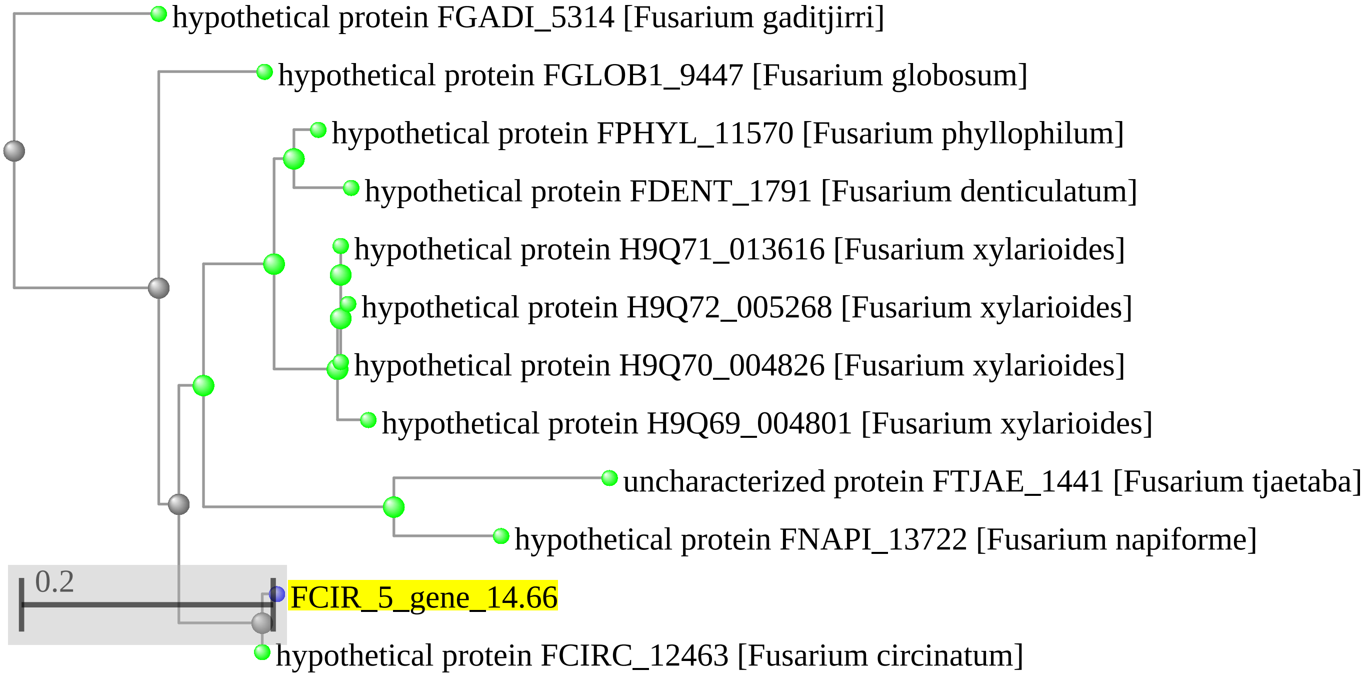
hypothetical protein FGADI_5314 [Fusarium gaditjirri]
hypothetical protein FGLOB1_9447 [Fusarium globosum]
hypothetical protein FPHYL_11570 [Fusarium phyllophilum]
hypothetical protein FDENT_1791 [Fusarium denticulatum]
hypothetical protein H9Q71_013616 [Fusarium xylarioides]
hypothetical protein H9Q72_005268 [Fusarium xylarioides]
hypothetical protein H9Q70_004826 [Fusarium xylarioides]
hypothetical protein H9Q69_004801 [Fusarium xylarioides]
uncharacterized protein FTJAE_1441 [Fusarium tjaetaba]
hypothetical protein FNAPI_13722 [Fusarium napiforme]
0.2
FCIR_5_gene_14.66
hypothetical protein FCIRC_12463 [Fusarium circinatum]

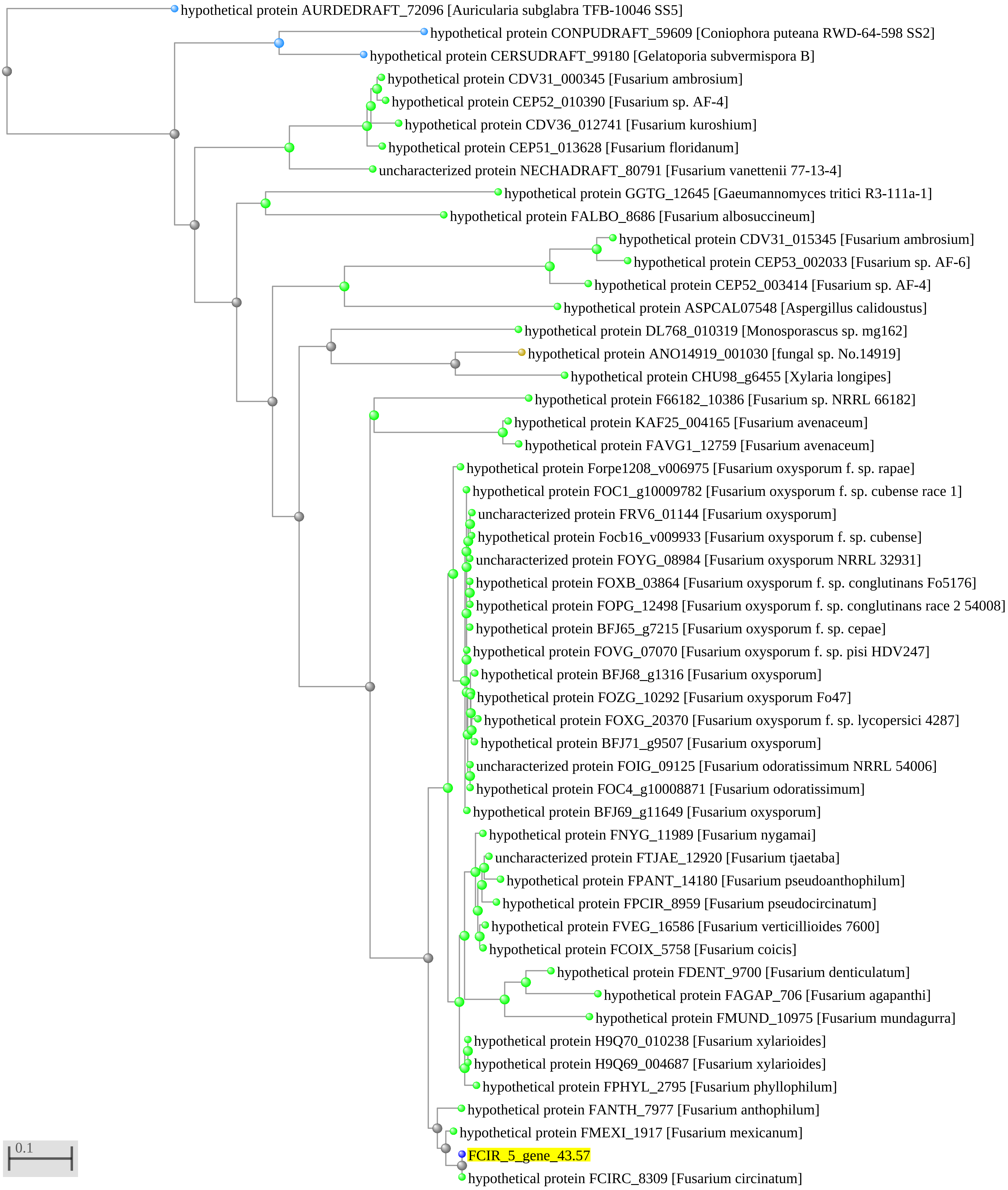
hypothetical protein AURDEDRAFT_72096 [Auricularia subglabra TFB-10046 SS5]
hypothetical protein CONPUDRAFT_59609 [Coniophora puteana RWD-64-598 SS2]
hypothetical protein CERSUDRAFT_99180 [Gelatoporia subvermispora B]
hypothetical protein CDV31_000345 [Fusarium ambrosium]
hypothetical protein CEP52_010390 [Fusarium sp. AF-4]
hypothetical protein CDV36_012741 [Fusarium kuroshium]
hypothetical protein CEP51_013628 [Fusarium floridanum]
uncharacterized protein NECHADRAFT_80791 [Fusarium vanettenii 77-13-4]
hypothetical protein GGTG_12645 [Gaeumannomyces tritici R3-111a-1]
hypothetical protein FALBO_8686 [Fusarium albosuccineum]
hypothetical protein CDV31_015345 [Fusarium ambrosium]
hypothetical protein CEP53_002033 [Fusarium sp. AF-6]
hypothetical protein CEP52_003414 [Fusarium sp. AF-4]
hypothetical protein ASPCAL07548 [Aspergillus calidoustus]
hypothetical protein DL768_010319 [Monosporascus sp. mg162]
hypothetical protein ANO14919_001030 [fungal sp. No.14919]
hypothetical protein CHU98_g6455 [Xylaria longipes]
hypothetical protein F66182_10386 [Fusarium sp. NRRL 66182]
hypothetical protein KAF25_004165 [Fusarium avenaceum]
hypothetical protein FAVG1_12759 [Fusarium avenaceum]
hypothetical protein Forpe1208_v006975 [Fusarium oxysporum f. sp. rapae]
hypothetical protein FOC1_g10009782 [Fusarium oxysporum f. sp. cubense race 1]
uncharacterized protein FRV6_01144 [Fusarium oxysporum]
hypothetical protein Focb16_v009933 [Fusarium oxysporum f. sp. cubense]
uncharacterized protein FOYG_08984 [Fusarium oxysporum NRRL 32931]
hypothetical protein FOXB_03864 [Fusarium oxysporum f. sp. conglutinans Fo5176]
hypothetical protein FOPG_12498 [Fusarium oxysporum f. sp. conglutinans race 2 54008]
hypothetical protein BFJ65_g7215 [Fusarium oxysporum f. sp. cepae]
hypothetical protein FOVG_07070 [Fusarium oxysporum f. sp. pisi HDV247]
hypothetical protein BFJ68_g1316 [Fusarium oxysporum]
hypothetical protein FOZG_10292 [Fusarium oxysporum Fo47]
hypothetical protein FOXG_20370 [Fusarium oxysporum f. sp. lycopersici 4287]
hypothetical protein BFJ71_g9507 [Fusarium oxysporum]
uncharacterized protein FOIG_09125 [Fusarium odoratissimum NRRL 54006]
hypothetical protein FOC4_g10008871 [Fusarium odoratissimum]
hypothetical protein BFJ69_g11649 [Fusarium oxysporum]
hypothetical protein FNYG_11989 [Fusarium nygamai]
uncharacterized protein FTJAE_12920 [Fusarium tjaetaba]
hypothetical protein FPANT_14180 [Fusarium pseudoanthophilum]
hypothetical protein FPCIR_8959 [Fusarium pseudocircinatum]
hypothetical protein FVEG_16586 [Fusarium verticillioides 7600]
hypothetical protein FCOIX_5758 [Fusarium coicis]
hypothetical protein FDENT_9700 [Fusarium denticulatum]
hypothetical protein FAGAP_706 [Fusarium agapanthi]
hypothetical protein FMUND_10975 [Fusarium mundagurra]
hypothetical protein H9Q70_010238 [Fusarium xylarioides]
hypothetical protein H9Q69_004687 [Fusarium xylarioides]
hypothetical protein FPHYL_2795 [Fusarium phyllophilum]
hypothetical protein FANTH_7977 [Fusarium anthophilum]
hypothetical protein FMEXI_1917 [Fusarium mexicanum]
FCIR_5_gene_43.57
hypothetical protein FCIRC_8309 [Fusarium circinatum]
0.1

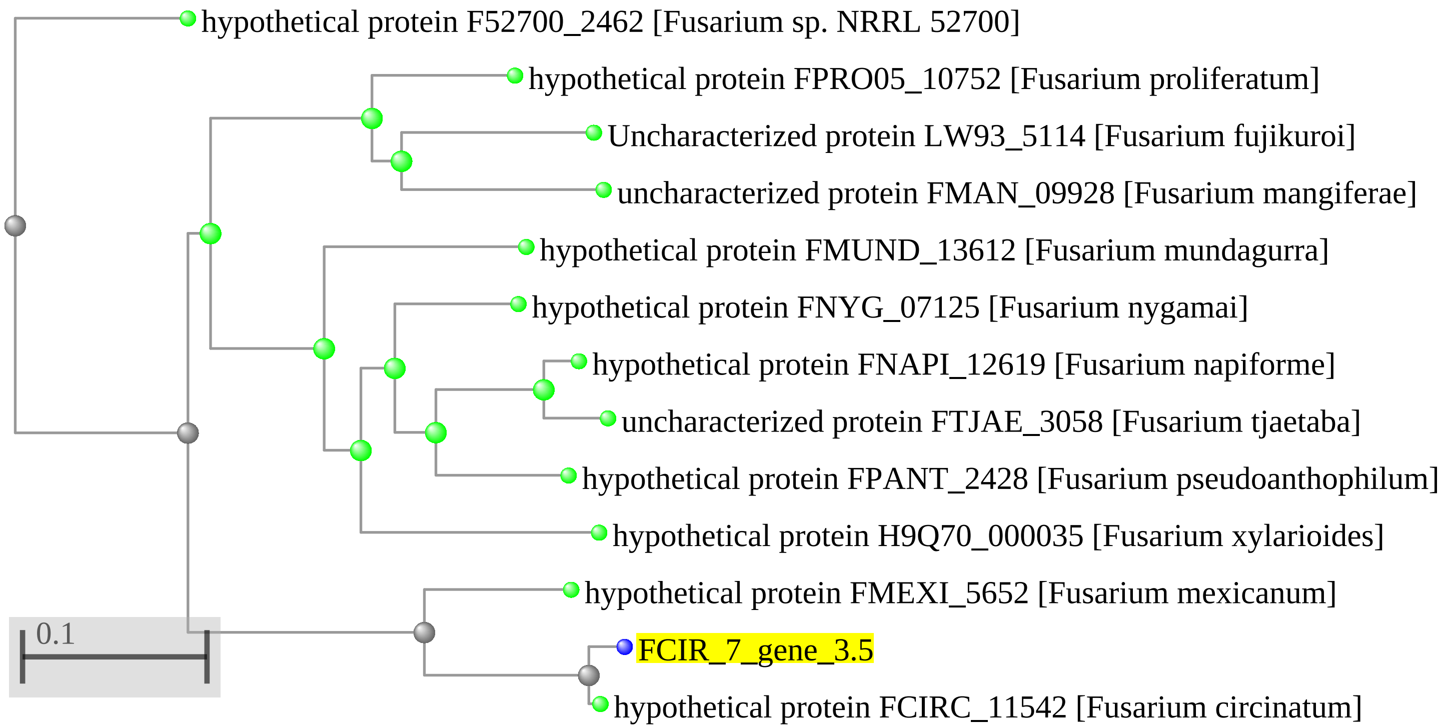
hypothetical protein F52700_2462 [Fusarium sp. NRRL 52700]
hypothetical protein FPRO05_10752 [Fusarium proliferatum]
Uncharacterized protein LW93_5114 [Fusarium fujikuroi]
uncharacterized protein FMAN_09928 [Fusarium mangiferae]
hypothetical protein FMUND_13612 [Fusarium mundagurra]
hypothetical protein FNYG_07125 [Fusarium nygamai]
hypothetical protein FNAPI_12619 [Fusarium napiforme]
uncharacterized protein FTJAE_3058 [Fusarium tjaetaba]
hypothetical protein FPANT_2428 [Fusarium pseudoanthophilum]
hypothetical protein H9Q70_000035 [Fusarium xylarioides]
hypothetical protein FMEXI_5652 [Fusarium mexicanum]
0.1
FCIR_7_gene_3.5
hypothetical protein FCIRC_11542 [Fusarium circinatum]

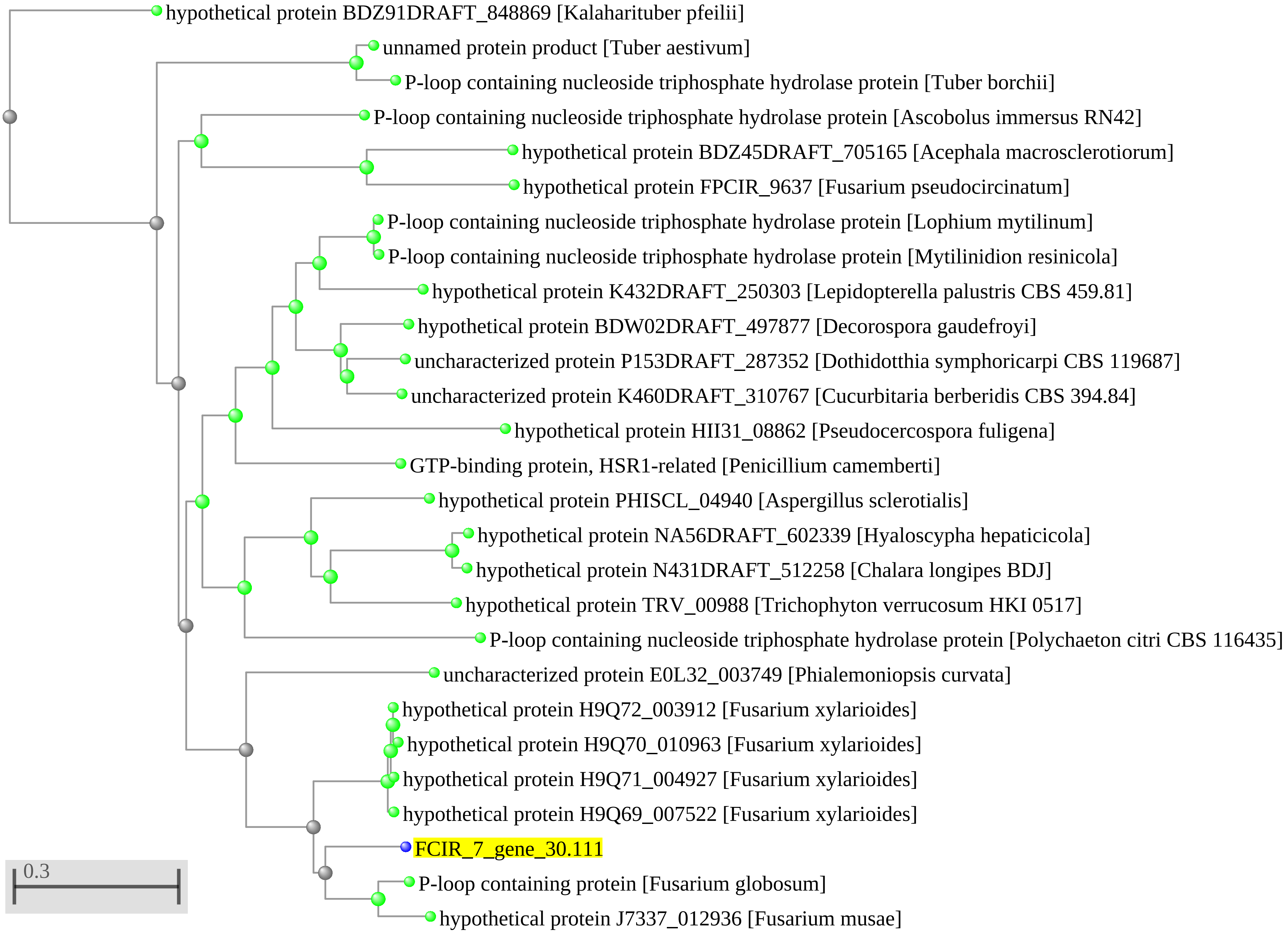
hypothetical protein BDZ91DRAFT_848869 [Kalaharituber pfeilii]
unnamed protein product [Tuber aestivum]
P-loop containing nucleoside triphosphate hydrolase protein [Tuber borchii]
P-loop containing nucleoside triphosphate hydrolase protein [Ascobolus immersus RN42]
hypothetical protein BDZ45DRAFT_705165 [Acephala macrosclerotiorum]
hypothetical protein FPCIR_9637 [Fusarium pseudocircinatum]
P-loop containing nucleoside triphosphate hydrolase protein [Lophium mytilinum]
P-loop containing nucleoside triphosphate hydrolase protein [Mytilinidion resinicola]
hypothetical protein K432DRAFT_250303 [Lepidopterella palustris CBS 459.81]
hypothetical protein BDW02DRAFT_497877 [Decorospora gaudefroyi]
uncharacterized protein P153DRAFT_287352 [Dothidotthia symphoricarpi CBS 119687]
uncharacterized protein K460DRAFT_310767 [Cucurbitaria berberidis CBS 394.84]
hypothetical protein HII31_08862 [Pseudocercospora fuligena]
GTP-binding protein, HSR1-related [Penicillium camemberti]
hypothetical protein PHISCL_04940 [Aspergillus sclerotialis]
hypothetical protein NA56DRAFT_602339 [Hyaloscypha hepaticicola]
hypothetical protein N431DRAFT_512258 [Chalara longipes BDJ]
hypothetical protein TRV_00988 [Trichophyton verrucosum HKI 0517]
P-loop containing nucleoside triphosphate hydrolase protein [Polychaeton citri CBS 116435]
uncharacterized protein E0L32_003749 [Phialemoniopsis curvata]
hypothetical protein H9Q72_003912 [Fusarium xylarioides]
hypothetical protein H9Q70_010963 [Fusarium xylarioides]
hypothetical protein H9Q71_004927 [Fusarium xylarioides]
hypothetical protein H9Q69_007522 [Fusarium xylarioides]
FCIR_7_gene_30.111
P-loop containing protein [Fusarium globosum]
hypothetical protein J7337_012936 [Fusarium musae]
0.3

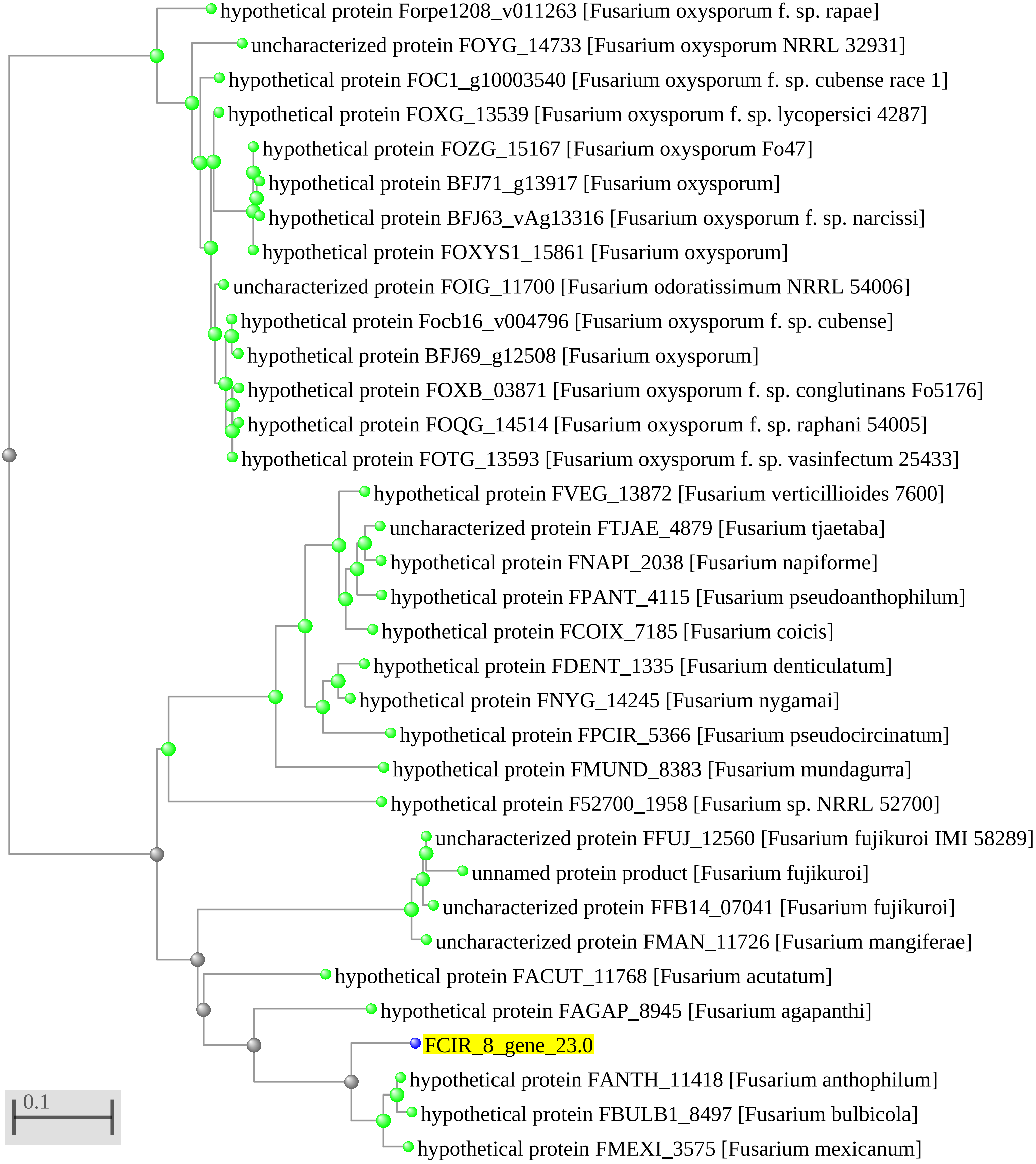
hypothetical protein Forpe1208_v011263 [Fusarium oxysporum f. sp. rapae]
uncharacterized protein FOYG_14733 [Fusarium oxysporum NRRL 32931]
hypothetical protein FOC1_g10003540 [Fusarium oxysporum f. sp. cubense race 1]
hypothetical protein FOXG_13539 [Fusarium oxysporum f. sp. lycopersici 4287]
hypothetical protein FOZG_15167 [Fusarium oxysporum Fo47]
hypothetical protein BFJ71_g13917 [Fusarium oxysporum]
hypothetical protein BFJ63_vAg13316 [Fusarium oxysporum f. sp. narcissi]
hypothetical protein FOXYS1_15861 [Fusarium oxysporum]
uncharacterized protein FOIG_11700 [Fusarium odoratissimum NRRL 54006]
hypothetical protein Focb16_v004796 [Fusarium oxysporum f. sp. cubense]
hypothetical protein BFJ69_g12508 [Fusarium oxysporum]
hypothetical protein FOXB_03871 [Fusarium oxysporum f. sp. conglutinans Fo5176]
hypothetical protein FOQG_14514 [Fusarium oxysporum f. sp. raphani 54005]
hypothetical protein FOTG_13593 [Fusarium oxysporum f. sp. vasinfectum 25433]
hypothetical protein FVEG_13872 [Fusarium verticillioides 7600]
uncharacterized protein FTJAE_4879 [Fusarium tjaetaba]
hypothetical protein FNAPI_2038 [Fusarium napiforme]
hypothetical protein FPANT_4115 [Fusarium pseudoanthophilum]
hypothetical protein FCOIX_7185 [Fusarium coicis]
hypothetical protein FDENT_1335 [Fusarium denticulatum]
hypothetical protein FNYG_14245 [Fusarium nygamai]
hypothetical protein FPCIR_5366 [Fusarium pseudocircinatum]
hypothetical protein FMUND_8383 [Fusarium mundagurra]
hypothetical protein F52700_1958 [Fusarium sp. NRRL 52700]
uncharacterized protein FFUJ_12560 [Fusarium fujikuroi IMI 58289]
unnamed protein product [Fusarium fujikuroi]
uncharacterized protein FFB14_07041 [Fusarium fujikuroi]
uncharacterized protein FMAN_11726 [Fusarium mangiferae]
hypothetical protein FACUT_11768 [Fusarium acutatum]
hypothetical protein FAGAP_8945 [Fusarium agapanthi]
FCIR_8_gene_23.0
hypothetical protein FANTH_11418 [Fusarium anthophilum]
hypothetical protein FBULB1_8497 [Fusarium bulbicola]
hypothetical protein FMEXI_3575 [Fusarium mexicanum]
0.1

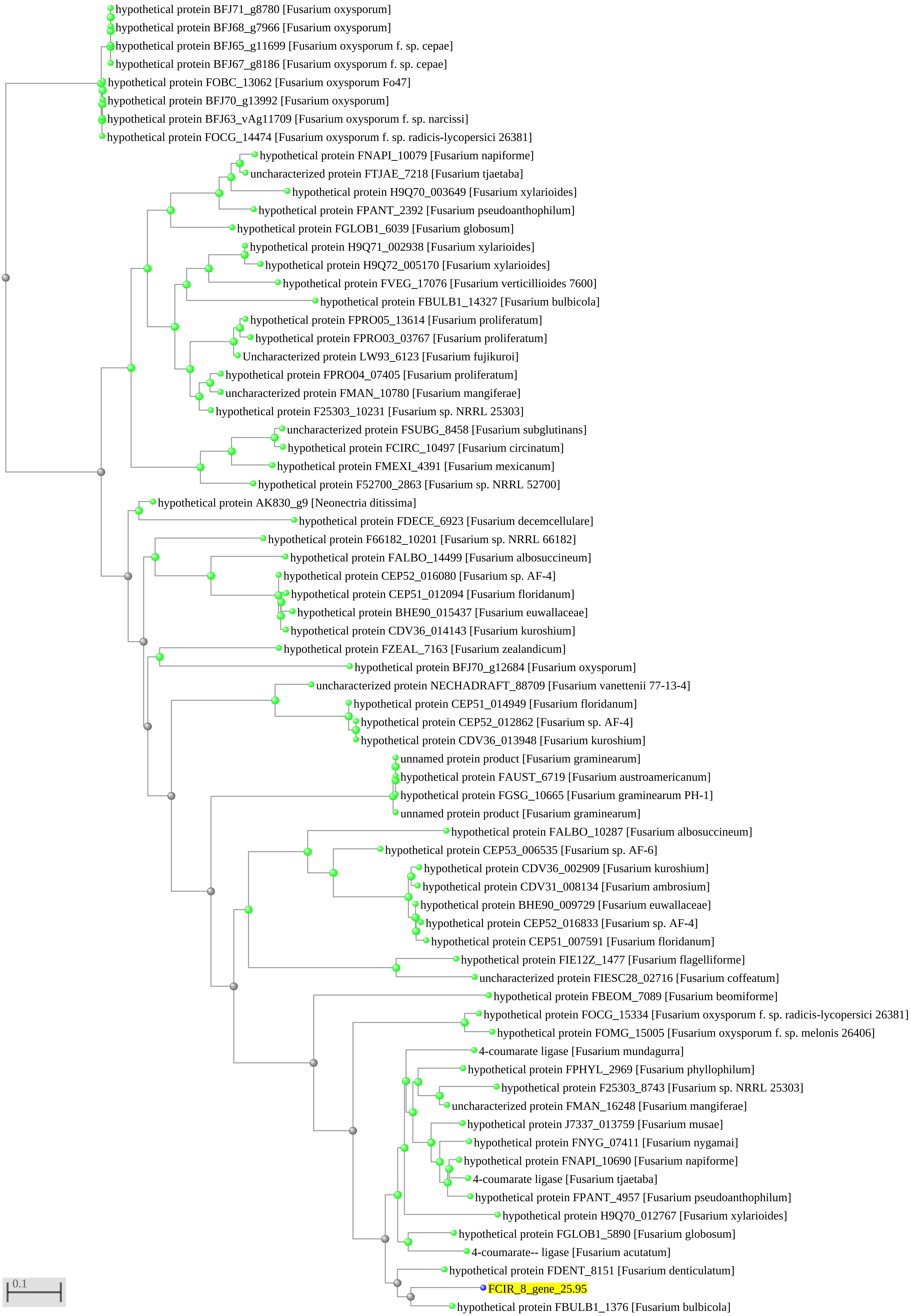
hypothetical protein BFJ71_g8780 [Fusarium oxysporum]
hypothetical protein BFJ68_g7966 [Fusarium oxysporum]
hypothetical protein BFJ65_g11699 [Fusarium oxysporum f. sp. cepae]
hypothetical protein BFJ67_g8186 [Fusarium oxysporum f. sp. cepae]
hypothetical protein FOBC_13062 [Fusarium oxysporum Fo47]
hypothetical protein BFJ70_g13992 [Fusarium oxysporum]
hypothetical protein BFJ63_vAg11709 [Fusarium oxysporum f. sp. narcissi]
hypothetical protein FOCG_14474 [Fusarium oxysporum f. sp. radicis-lycopersici 26381]
hypothetical protein FNAPI_10079 [Fusarium napiforme]
uncharacterized protein FTJAE_7218 [Fusarium tjaetaba]
hypothetical protein H9Q70_003649 [Fusarium xylarioides]
hypothetical protein FPANT_2392 [Fusarium pseudoanthophilum]
hypothetical protein FGLOB1_6039 [Fusarium globosum]
hypothetical protein H9Q71_002938 [Fusarium xylarioides]
hypothetical protein H9Q72_005170 [Fusarium xylarioides]
hypothetical protein FVEG_17076 [Fusarium verticillioides 7600]
hypothetical protein FBULB1_14327 [Fusarium bulbicola]
hypothetical protein FPRO05_13614 [Fusarium proliferatum]
hypothetical protein FPRO03_03767 [Fusarium proliferatum]
Uncharacterized protein LW93_6123 [Fusarium fujikuroi]
hypothetical protein FPRO04_07405 [Fusarium proliferatum]
uncharacterized protein FMAN_10780 [Fusarium mangiferae]
hypothetical protein F25303_10231 [Fusarium sp. NRRL 25303]
uncharacterized protein FSUBG_8458 [Fusarium subglutinans]
hypothetical protein FCIRC_10497 [Fusarium circinatum]
hypothetical protein FMEXI_4391 [Fusarium mexicanum]
hypothetical protein F52700_2863 [Fusarium sp. NRRL 52700]
hypothetical protein AK830_g9 [Neonectria ditissima]
hypothetical protein FDECE_6923 [Fusarium decemcellulare]
hypothetical protein F66182_10201 [Fusarium sp. NRRL 66182]
hypothetical protein FALBO_14499 [Fusarium albosuccineum]
hypothetical protein CEP52_016080 [Fusarium sp. AF-4]
hypothetical protein CEP51_012094 [Fusarium floridanum]
hypothetical protein BHE90_015437 [Fusarium euwallaceae]
hypothetical protein CDV36_014143 [Fusarium kuroshium]
hypothetical protein FZEAL_7163 [Fusarium zealandicum]
hypothetical protein BFJ70_g12684 [Fusarium oxysporum]
uncharacterized protein NECHADRAFT_88709 [Fusarium vanettenii 77-13-4]
hypothetical protein CEP51_014949 [Fusarium floridanum]
hypothetical protein CEP52_012862 [Fusarium sp. AF-4]
hypothetical protein CDV36_013948 [Fusarium kuroshium]
unnamed protein product [Fusarium graminearum]
hypothetical protein FAUST_6719 [Fusarium austroamericanum]
hypothetical protein FGSG_10665 [Fusarium graminearum PH-1]
unnamed protein product [Fusarium graminearum]
hypothetical protein FALBO_10287 [Fusarium albosuccineum]
hypothetical protein CEP53_006535 [Fusarium sp. AF-6]
hypothetical protein CDV36_002909 [Fusarium kuroshium]
hypothetical protein CDV31_008134 [Fusarium ambrosium]
hypothetical protein BHE90_009729 [Fusarium euwallaceae]
hypothetical protein CEP52_016833 [Fusarium sp. AF-4]
hypothetical protein CEP51_007591 [Fusarium floridanum]
hypothetical protein FIE12Z_1477 [Fusarium flagelliforme]
uncharacterized protein FIESC28_02716 [Fusarium coffeatum]
hypothetical protein FBEOM_7089 [Fusarium beomiforme]
hypothetical protein FOCG_15334 [Fusarium oxysporum f. sp. radicis-lycopersici 26381]
hypothetical protein FOMG_15005 [Fusarium oxysporum f. sp. melonis 26406]
4-coumarate ligase [Fusarium mundagurra]
hypothetical protein FPHYL_2969 [Fusarium phyllophilum]
hypothetical protein F25303_8743 [Fusarium sp. NRRL 25303]
uncharacterized protein FMAN_16248 [Fusarium mangiferae]
hypothetical protein J7337_013759 [Fusarium musae]
hypothetical protein FNYG_07411 [Fusarium nygamai]
hypothetical protein FNAPI_10690 [Fusarium napiforme]
4-coumarate ligase [Fusarium tjaetaba]
hypothetical protein FPANT_4957 [Fusarium pseudoanthophilum]
hypothetical protein H9Q70_012767 [Fusarium xylarioides]
hypothetical protein FGLOB1_5890 [Fusarium globosum]
4-coumarate-- ligase [Fusarium acutatum]
hypothetical protein FDENT_8151 [Fusarium denticulatum]
FCIR_8_gene_25.95
hypothetical protein FBULB1_1376 [Fusarium bulbicola]
0.1

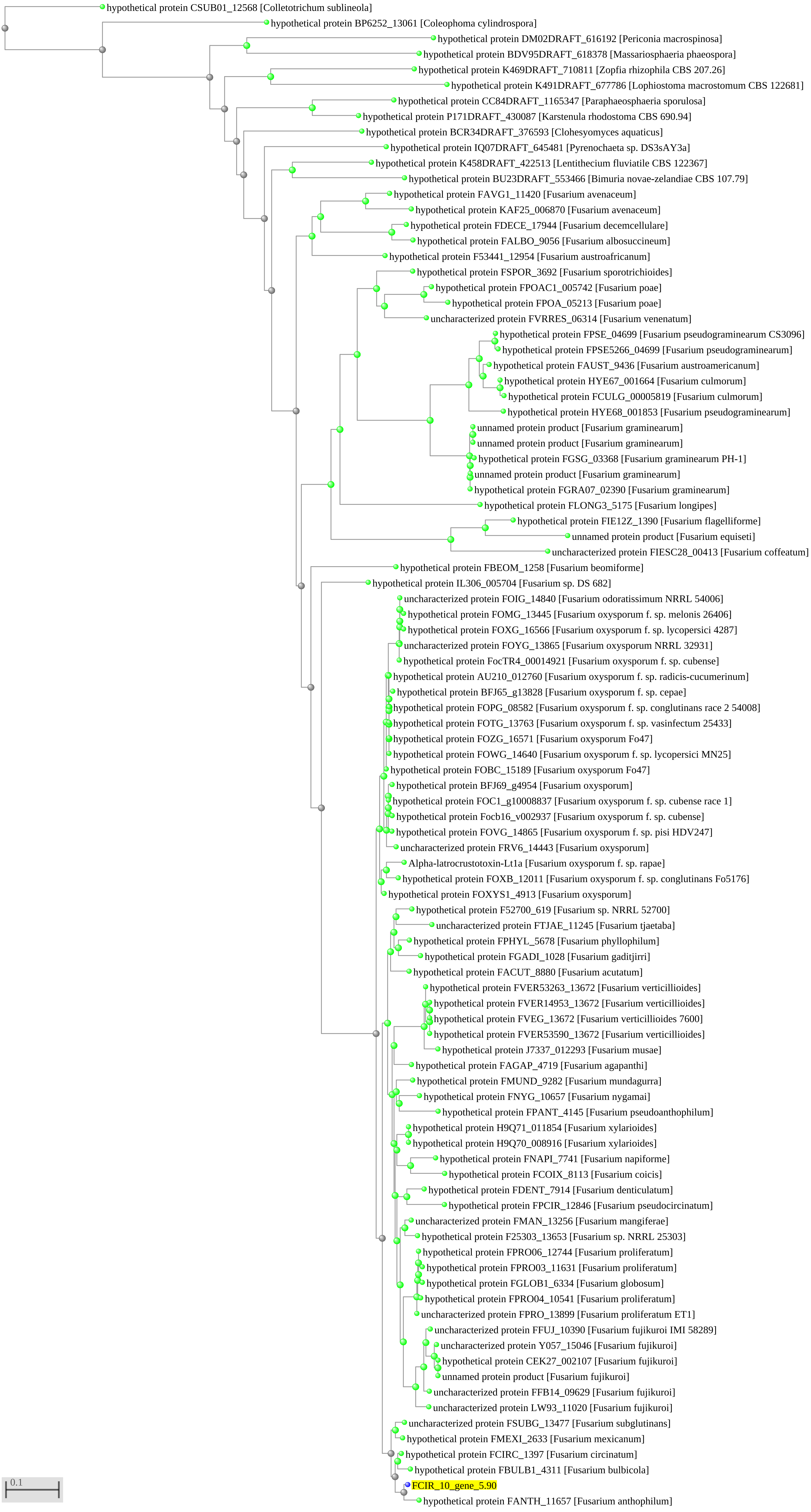
hypothetical protein CSUB01_12568 [Colletotrichum sublineola]
hypothetical protein BP6252_13061 [Coleophoma cylindrospora]
hypothetical protein DM02DRAFT_616192 [Periconia macrospinosa]
hypothetical protein BDV95DRAFT_618378 [Massariosphaeria phaeospora]
hypothetical protein K469DRAFT_710811 [Zopfia rhizophila CBS 207.26]
hypothetical protein K491DRAFT_677786 [Lophiostoma macrostomum CBS 122681]
hypothetical protein CC84DRAFT_1165347 [Paraphaeosphaeria sporulosa]
hypothetical protein P171DRAFT_430087 [Karstenula rhodostoma CBS 690.94]
hypothetical protein BCR34DRAFT_376593 [Clohesyomyces aquaticus]
hypothetical protein IQ07DRAFT_645481 [Pyrenochaeta sp. DS3sAY3a]
hypothetical protein K458DRAFT_422513 [Lentithecium fluviatile CBS 122367]
hypothetical protein BU23DRAFT_553466 [Bimuria novae-zelandiae CBS 107.79]
hypothetical protein FAVG1_11420 [Fusarium avenaceum]
hypothetical protein KAF25_006870 [Fusarium avenaceum]
hypothetical protein FDECE_17944 [Fusarium decemcellulare]
hypothetical protein FALBO_9056 [Fusarium albosuccineum]
hypothetical protein F53441_12954 [Fusarium austroafricanum]
hypothetical protein FSPOR_3692 [Fusarium sporotrichioides]
hypothetical protein FPOAC1_005742 [Fusarium poae]
hypothetical protein FPOA_05213 [Fusarium poae]
uncharacterized protein FVRRES_06314 [Fusarium venenatum]
hypothetical protein FPSE_04699 [Fusarium pseudograminearum CS3096]
hypothetical protein FPSE5266_04699 [Fusarium pseudograminearum]
hypothetical protein FAUST_9436 [Fusarium austroamericanum]
hypothetical protein HYE67_001664 [Fusarium culmorum]
hypothetical protein FCULG_00005819 [Fusarium culmorum]
hypothetical protein HYE68_001853 [Fusarium pseudograminearum]
unnamed protein product [Fusarium graminearum]
unnamed protein product [Fusarium graminearum]
hypothetical protein FGSG_03368 [Fusarium graminearum PH-1]
unnamed protein product [Fusarium graminearum]
hypothetical protein FGRA07_02390 [Fusarium graminearum]
hypothetical protein FLONG3_5175 [Fusarium longipes]
hypothetical protein FIE12Z_1390 [Fusarium flagelliforme]
unnamed protein product [Fusarium equiseti]
uncharacterized protein FIESC28_00413 [Fusarium coffeatum]
hypothetical protein FBEOM_1258 [Fusarium beomiforme]
hypothetical protein IL306_005704 [Fusarium sp. DS 682]
uncharacterized protein FOIG_14840 [Fusarium odoratissimum NRRL 54006]
hypothetical protein FOMG_13445 [Fusarium oxysporum f. sp. melonis 26406]
hypothetical protein FOXG_16566 [Fusarium oxysporum f. sp. lycopersici 4287]
uncharacterized protein FOYG_13865 [Fusarium oxysporum NRRL 32931]
hypothetical protein FocTR4_00014921 [Fusarium oxysporum f. sp. cubense]
hypothetical protein AU210_012760 [Fusarium oxysporum f. sp. radicis-cucumerinum]
hypothetical protein BFJ65_g13828 [Fusarium oxysporum f. sp. cepae]
hypothetical protein FOPG_08582 [Fusarium oxysporum f. sp. conglutinans race 2 54008]
hypothetical protein FOTG_13763 [Fusarium oxysporum f. sp. vasinfectum 25433]
hypothetical protein FOZG_16571 [Fusarium oxysporum Fo47]
hypothetical protein FOWG_14640 [Fusarium oxysporum f. sp. lycopersici MN25]
hypothetical protein FOBC_15189 [Fusarium oxysporum Fo47]
hypothetical protein BFJ69_g4954 [Fusarium oxysporum]
hypothetical protein FOC1_g10008837 [Fusarium oxysporum f. sp. cubense race 1]
hypothetical protein Focb16_v002937 [Fusarium oxysporum f. sp. cubense]
hypothetical protein FOVG_14865 [Fusarium oxysporum f. sp. pisi HDV247]
uncharacterized protein FRV6_14443 [Fusarium oxysporum]
Alpha-latrocrustotoxin-Lt1a [Fusarium oxysporum f. sp. rapae]
hypothetical protein FOXB_12011 [Fusarium oxysporum f. sp. conglutinans Fo5176]
hypothetical protein FOXYS1_4913 [Fusarium oxysporum]
hypothetical protein F52700_619 [Fusarium sp. NRRL 52700]
uncharacterized protein FTJAE_11245 [Fusarium tjaetaba]
hypothetical protein FPHYL_5678 [Fusarium phyllophilum]
hypothetical protein FGADI_1028 [Fusarium gaditjirri]
hypothetical protein FACUT_8880 [Fusarium acutatum]
hypothetical protein FVER53263_13672 [Fusarium verticillioides]
hypothetical protein FVER14953_13672 [Fusarium verticillioides]
hypothetical protein FVEG_13672 [Fusarium verticillioides 7600]
hypothetical protein FVER53590_13672 [Fusarium verticillioides]
hypothetical protein J7337_012293 [Fusarium musae]
hypothetical protein FAGAP_4719 [Fusarium agapanthi]
hypothetical protein FMUND_9282 [Fusarium mundagurra]
hypothetical protein FNYG_10657 [Fusarium nygamai]
hypothetical protein FPANT_4145 [Fusarium pseudoanthophilum]
hypothetical protein H9Q71_011854 [Fusarium xylarioides]
hypothetical protein H9Q70_008916 [Fusarium xylarioides]
hypothetical protein FNAPI_7741 [Fusarium napiforme]
hypothetical protein FCOIX_8113 [Fusarium coicis]
hypothetical protein FDENT_7914 [Fusarium denticulatum]
hypothetical protein FPCIR_12846 [Fusarium pseudocircinatum]
uncharacterized protein FMAN_13256 [Fusarium mangiferae]
hypothetical protein F25303_13653 [Fusarium sp. NRRL 25303]
hypothetical protein FPRO06_12744 [Fusarium proliferatum]
hypothetical protein FPRO03_11631 [Fusarium proliferatum]
hypothetical protein FGLOB1_6334 [Fusarium globosum]
hypothetical protein FPRO04_10541 [Fusarium proliferatum]
uncharacterized protein FPRO_13899 [Fusarium proliferatum ET1]
uncharacterized protein FFUJ_10390 [Fusarium fujikuroi IMI 58289]
uncharacterized protein Y057_15046 [Fusarium fujikuroi]
hypothetical protein CEK27_002107 [Fusarium fujikuroi]
unnamed protein product [Fusarium fujikuroi]
uncharacterized protein FFB14_09629 [Fusarium fujikuroi]
uncharacterized protein LW93_11020 [Fusarium fujikuroi]
uncharacterized protein FSUBG_13477 [Fusarium subglutinans]
hypothetical protein FMEXI_2633 [Fusarium mexicanum]
hypothetical protein FCIRC_1397 [Fusarium circinatum]
hypothetical protein FBULB1_4311 [Fusarium bulbicola]
FCIR_10_gene_5.90
hypothetical protein FANTH_11657 [Fusarium anthophilum]
0.1

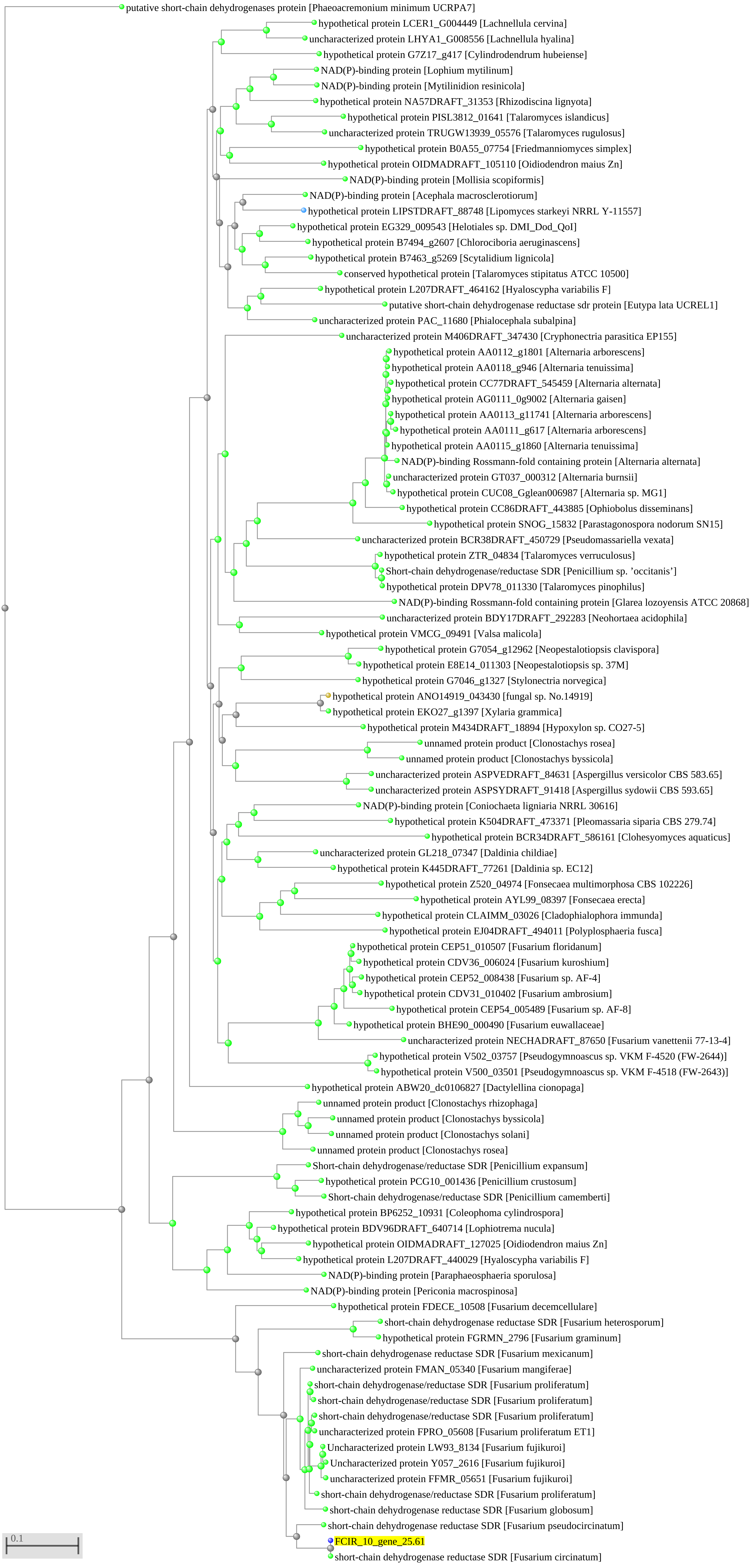
putative short-chain dehydrogenases protein [Phaeoacremonium minimum UCRPA7]
hypothetical protein LCER1_G004449 [Lachnellula cervina]
uncharacterized protein LHYA1_G008556 [Lachnellula hyalina]
hypothetical protein G7Z17_g417 [Cylindrodendrum hubeiense]
NAD(P)-binding protein [Lophium mytilinum]
NAD(P)-binding protein [Mytilinidion resinicola]
hypothetical protein NA57DRAFT_31353 [Rhizodiscina lignyota]
hypothetical protein PISL3812_01641 [Talaromyces islandicus]
uncharacterized protein TRUGW13939_05576 [Talaromyces rugulosus]
hypothetical protein B0A55_07754 [Friedmanniomyces simplex]
hypothetical protein OIDMADRAFT_105110 [Oidiodendron maius Zn]
NAD(P)-binding protein [Mollisia scopiformis]
NAD(P)-binding protein [Acephala macrosclerotiorum]
hypothetical protein LIPSTDRAFT_88748 [Lipomyces starkeyi NRRL Y-11557]
hypothetical protein EG329_009543 [Helotiales sp. DMI_Dod_QoI]
hypothetical protein B7494_g2607 [Chlorociboria aeruginascens]
hypothetical protein B7463_g5269 [Scytalidium lignicola]
conserved hypothetical protein [Talaromyces stipitatus ATCC 10500]
hypothetical protein L207DRAFT_464162 [Hyaloscypha variabilis F]
putative short-chain dehydrogenase reductase sdr protein [Eutypa lata UCREL1]
uncharacterized protein PAC_11680 [Phialocephala subalpina]
uncharacterized protein M406DRAFT_347430 [Cryphonectria parasitica EP155]
hypothetical protein AA0112_g1801 [Alternaria arborescens]
hypothetical protein AA0118_g946 [Alternaria tenuissima]
hypothetical protein CC77DRAFT_545459 [Alternaria alternata]
hypothetical protein AG0111_0g9002 [Alternaria gaisen]
hypothetical protein AA0113_g11741 [Alternaria arborescens]
hypothetical protein AA0111_g617 [Alternaria arborescens]
hypothetical protein AA0115_g1860 [Alternaria tenuissima]
NAD(P)-binding Rossmann-fold containing protein [Alternaria alternata]
uncharacterized protein GT037_000312 [Alternaria burnsii]
hypothetical protein CUC08_Gglean006987 [Alternaria sp. MG1]
hypothetical protein CC86DRAFT_443885 [Ophiobolus disseminans]
hypothetical protein SNOG_15832 [Parastagonospora nodorum SN15]
uncharacterized protein BCR38DRAFT_450729 [Pseudomassariella vexata]
hypothetical protein ZTR_04834 [Talaromyces verruculosus]
Short-chain dehydrogenase/reductase SDR [Penicillium sp. 'occitanis']
hypothetical protein DPV78_011330 [Talaromyces pinophilus]
NAD(P)-binding Rossmann-fold containing protein [Glarea lozoyensis ATCC 20868]
uncharacterized protein BDY17DRAFT_292283 [Neohortaea acidophila]
hypothetical protein VMCG_09491 [Valsa malicola]
hypothetical protein G7054_g12962 [Neopestalotiopsis clavispora]
hypothetical protein E8E14_011303 [Neopestalotiopsis sp. 37M]
hypothetical protein G7046_g1327 [Stylonectria norvegica]
hypothetical protein ANO14919_043430 [fungal sp. No.14919]
hypothetical protein EKO27_g1397 [Xylaria grammica]
hypothetical protein M434DRAFT_18894 [Hypoxylon sp. CO27-5]
unnamed protein product [Clonostachys rosea]
unnamed protein product [Clonostachys byssicola]
uncharacterized protein ASPVEDRAFT_84631 [Aspergillus versicolor CBS 583.65]
uncharacterized protein ASPSYDRAFT_91418 [Aspergillus sydowii CBS 593.65]
NAD(P)-binding protein [Coniochaeta ligniaria NRRL 30616]
hypothetical protein K504DRAFT_473371 [Pleomassaria siparia CBS 279.74]
hypothetical protein BCR34DRAFT_586161 [Clohesyomyces aquaticus]
uncharacterized protein GL218_07347 [Daldinia childiae]
hypothetical protein K445DRAFT_77261 [Daldinia sp. EC12]
hypothetical protein Z520_04974 [Fonsecaea multimorphosa CBS 102226]
hypothetical protein AYL99_08397 [Fonsecaea erecta]
hypothetical protein CLAIMM_03026 [Cladophialophora immunda]
hypothetical protein EJ04DRAFT_494011 [Polyplosphaeria fusca]
hypothetical protein CEP51_010507 [Fusarium floridanum]
hypothetical protein CDV36_006024 [Fusarium kuroshium]
hypothetical protein CEP52_008438 [Fusarium sp. AF-4]
hypothetical protein CDV31_010402 [Fusarium ambrosium]
hypothetical protein CEP54_005489 [Fusarium sp. AF-8]
hypothetical protein BHE90_000490 [Fusarium euwallaceae]
uncharacterized protein NECHADRAFT_87650 [Fusarium vanettenii 77-13-4]
hypothetical protein V502_03757 [Pseudogymnoascus sp. VKM F-4520 (FW-2644)]
hypothetical protein V500_03501 [Pseudogymnoascus sp. VKM F-4518 (FW-2643)]
hypothetical protein ABW20_dc0106827 [Dactylellina cionopaga]
unnamed protein product [Clonostachys rhizophaga]
unnamed protein product [Clonostachys byssicola]
unnamed protein product [Clonostachys solani]
unnamed protein product [Clonostachys rosea]
Short-chain dehydrogenase/reductase SDR [Penicillium expansum]
hypothetical protein PCG10_001436 [Penicillium crustosum]
Short-chain dehydrogenase/reductase SDR [Penicillium camemberti]
hypothetical protein BP6252_10931 [Coleophoma cylindrospora]
hypothetical protein BDV96DRAFT_640714 [Lophiotrema nucula]
hypothetical protein OIDMADRAFT_127025 [Oidiodendron maius Zn]
hypothetical protein L207DRAFT_440029 [Hyaloscypha variabilis F]
NAD(P)-binding protein [Paraphaeosphaeria sporulosa]
NAD(P)-binding protein [Periconia macrospinosa]
hypothetical protein FDECE_10508 [Fusarium decemcellulare]
short-chain dehydrogenase reductase SDR [Fusarium heterosporum]
hypothetical protein FGRMN_2796 [Fusarium graminum]
short-chain dehydrogenase reductase SDR [Fusarium mexicanum]
uncharacterized protein FMAN_05340 [Fusarium mangiferae]
short-chain dehydrogenase/reductase SDR [Fusarium proliferatum]
short-chain dehydrogenase/reductase SDR [Fusarium proliferatum]
short-chain dehydrogenase/reductase SDR [Fusarium proliferatum]
uncharacterized protein FPRO_05608 [Fusarium proliferatum ET1]
Uncharacterized protein LW93_8134 [Fusarium fujikuroi]
Uncharacterized protein Y057_2616 [Fusarium fujikuroi]
uncharacterized protein FFMR_05651 [Fusarium fujikuroi]
short-chain dehydrogenase/reductase SDR [Fusarium proliferatum]
short-chain dehydrogenase reductase SDR [Fusarium globosum]
short-chain dehydrogenase reductase SDR [Fusarium pseudocircinatum]
FCIR_10_gene_25.61
short-chain dehydrogenase reductase SDR [Fusarium circinatum]
0.1

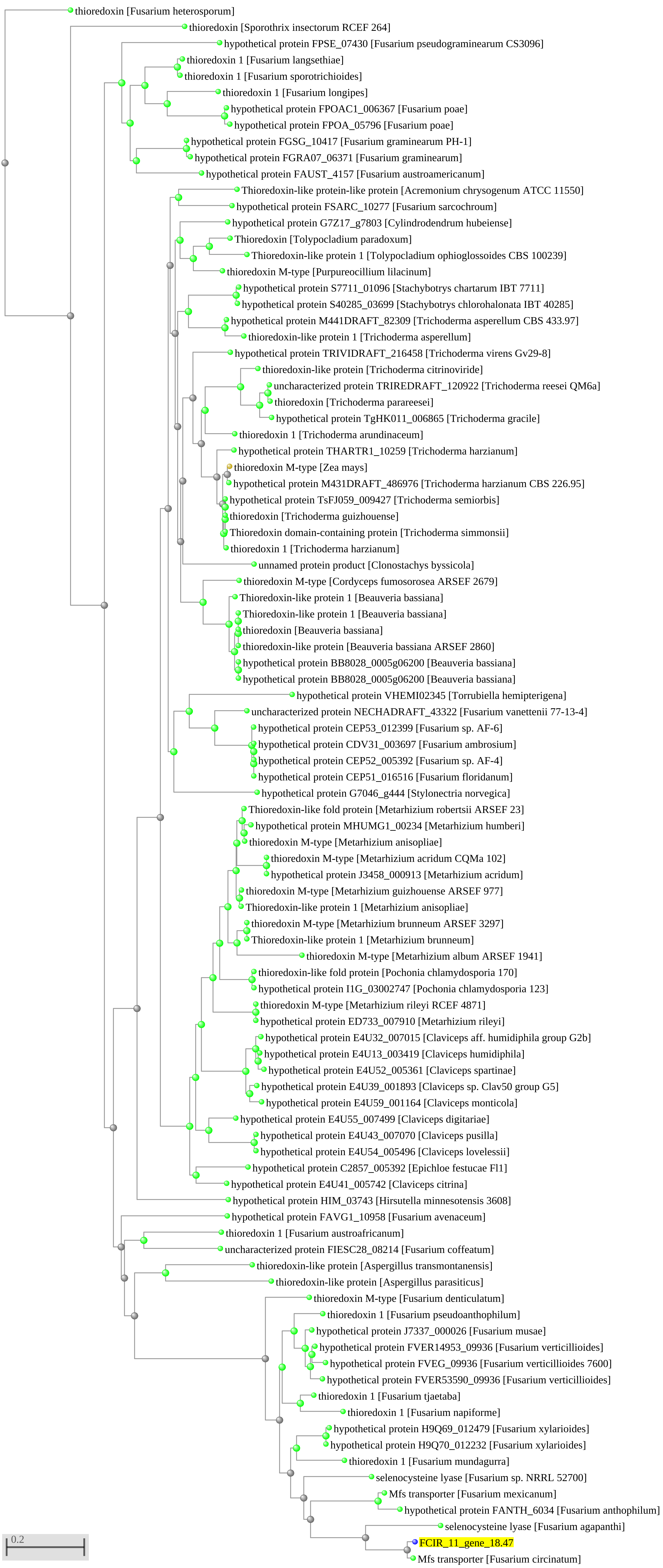
thioredoxin [Fusarium heterosporum]
thioredoxin [Sporothrix insectorum RCEF 264]
hypothetical protein FPSE_07430 [Fusarium pseudograminearum CS3096]
thioredoxin 1 [Fusarium langsethiae]
thioredoxin 1 [Fusarium sporotrichioides]
thioredoxin 1 [Fusarium longipes]
hypothetical protein FPOAC1_006367 [Fusarium poae]
hypothetical protein FPOA_05796 [Fusarium poae]
hypothetical protein FGSG_10417 [Fusarium graminearum PH-1]
hypothetical protein FGRA07_06371 [Fusarium graminearum]
hypothetical protein FAUST_4157 [Fusarium austroamericanum]
Thioredoxin-like protein-like protein [Acremonium chrysogenum ATCC 11550]
hypothetical protein FSARC_10277 [Fusarium sarcochroum]
hypothetical protein G7Z17_g7803 [Cylindrodendrum hubeiense]
Thioredoxin [Tolypocladium paradoxum]
Thioredoxin-like protein 1 [Tolypocladium ophioglossoides CBS 100239]
thioredoxin M-type [Purpureocillium lilacinum]
hypothetical protein S7711_01096 [Stachybotrys chartarum IBT 7711]
hypothetical protein S40285_03699 [Stachybotrys chlorohalonata IBT 40285]
hypothetical protein M441DRAFT_82309 [Trichoderma asperellum CBS 433.97]
thioredoxin-like protein 1 [Trichoderma asperellum]
hypothetical protein TRIVIDRAFT_216458 [Trichoderma virens Gv29-8]
thioredoxin-like protein [Trichoderma citrinoviride]
uncharacterized protein TRIREDRAFT_120922 [Trichoderma reesei QM6a]
thioredoxin [Trichoderma parareesei]
hypothetical protein TgHK011_006865 [Trichoderma gracile]
thioredoxin 1 [Trichoderma arundinaceum]
hypothetical protein THARTR1_10259 [Trichoderma harzianum]
thioredoxin M-type [Zea mays]
hypothetical protein M431DRAFT_486976 [Trichoderma harzianum CBS 226.95]
hypothetical protein TsFJ059_009427 [Trichoderma semiorbis]
thioredoxin [Trichoderma guizhouense]
Thioredoxin domain-containing protein [Trichoderma simmonsii]
thioredoxin 1 [Trichoderma harzianum]
unnamed protein product [Clonostachys byssicola]
thioredoxin M-type [Cordyceps fumosorosea ARSEF 2679]
Thioredoxin-like protein 1 [Beauveria bassiana]
Thioredoxin-like protein 1 [Beauveria bassiana]
thioredoxin [Beauveria bassiana]
thioredoxin-like protein [Beauveria bassiana ARSEF 2860]
hypothetical protein BB8028_0005g06200 [Beauveria bassiana]
hypothetical protein BB8028_0005g06200 [Beauveria bassiana]
hypothetical protein VHEMI02345 [Torrubiella hemipterigena]
uncharacterized protein NECHADRAFT_43322 [Fusarium vanettenii 77-13-4]
hypothetical protein CEP53_012399 [Fusarium sp. AF-6]
hypothetical protein CDV31_003697 [Fusarium ambrosium]
hypothetical protein CEP52_005392 [Fusarium sp. AF-4]
hypothetical protein CEP51_016516 [Fusarium floridanum]
hypothetical protein G7046_g444 [Stylonectria norvegica]
Thioredoxin-like fold protein [Metarhizium robertsii ARSEF 23]
hypothetical protein MHUMG1_00234 [Metarhizium humberi]
thioredoxin M-type [Metarhizium anisopliae]
thioredoxin M-type [Metarhizium acridum CQMa 102]
hypothetical protein J3458_000913 [Metarhizium acridum]
thioredoxin M-type [Metarhizium guizhouense ARSEF 977]
Thioredoxin-like protein 1 [Metarhizium anisopliae]
thioredoxin M-type [Metarhizium brunneum ARSEF 3297]
Thioredoxin-like protein 1 [Metarhizium brunneum]
thioredoxin M-type [Metarhizium album ARSEF 1941]
thioredoxin-like fold protein [Pochonia chlamydosporia 170]
hypothetical protein I1G_03002747 [Pochonia chlamydosporia 123]
thioredoxin M-type [Metarhizium rileyi RCEF 4871]
hypothetical protein ED733_007910 [Metarhizium rileyi]
hypothetical protein E4U32_007015 [Claviceps aff. humidiphila group G2b]
hypothetical protein E4U13_003419 [Claviceps humidiphila]
hypothetical protein E4U52_005361 [Claviceps spartinae]
hypothetical protein E4U39_001893 [Claviceps sp. Clav50 group G5]
hypothetical protein E4U59_001164 [Claviceps monticola]
hypothetical protein E4U55_007499 [Claviceps digitariae]
hypothetical protein E4U43_007070 [Claviceps pusilla]
hypothetical protein E4U54_005496 [Claviceps lovelessii]
hypothetical protein C2857_005392 [Epichloe festucae Fl1]
hypothetical protein E4U41_005742 [Claviceps citrina]
hypothetical protein HIM_03743 [Hirsutella minnesotensis 3608]
hypothetical protein FAVG1_10958 [Fusarium avenaceum]
thioredoxin 1 [Fusarium austroafricanum]
uncharacterized protein FIESC28_08214 [Fusarium coffeatum]
thioredoxin-like protein [Aspergillus transmontanensis]
thioredoxin-like protein [Aspergillus parasiticus]
thioredoxin M-type [Fusarium denticulatum]
thioredoxin 1 [Fusarium pseudoanthophilum]
hypothetical protein J7337_000026 [Fusarium musae]
hypothetical protein FVER14953_09936 [Fusarium verticillioides]
hypothetical protein FVEG_09936 [Fusarium verticillioides 7600]
hypothetical protein FVER53590_09936 [Fusarium verticillioides]
thioredoxin 1 [Fusarium tjaetaba]
thioredoxin 1 [Fusarium napiforme]
hypothetical protein H9Q69_012479 [Fusarium xylarioides]
hypothetical protein H9Q70_012232 [Fusarium xylarioides]
thioredoxin 1 [Fusarium mundagurra]
selenocysteine lyase [Fusarium sp. NRRL 52700]
Mfs transporter [Fusarium mexicanum]
hypothetical protein FANTH_6034 [Fusarium anthophilum]
selenocysteine lyase [Fusarium agapanthi]
FCIR_11_gene_18.47
Mfs transporter [Fusarium circinatum]
0.2

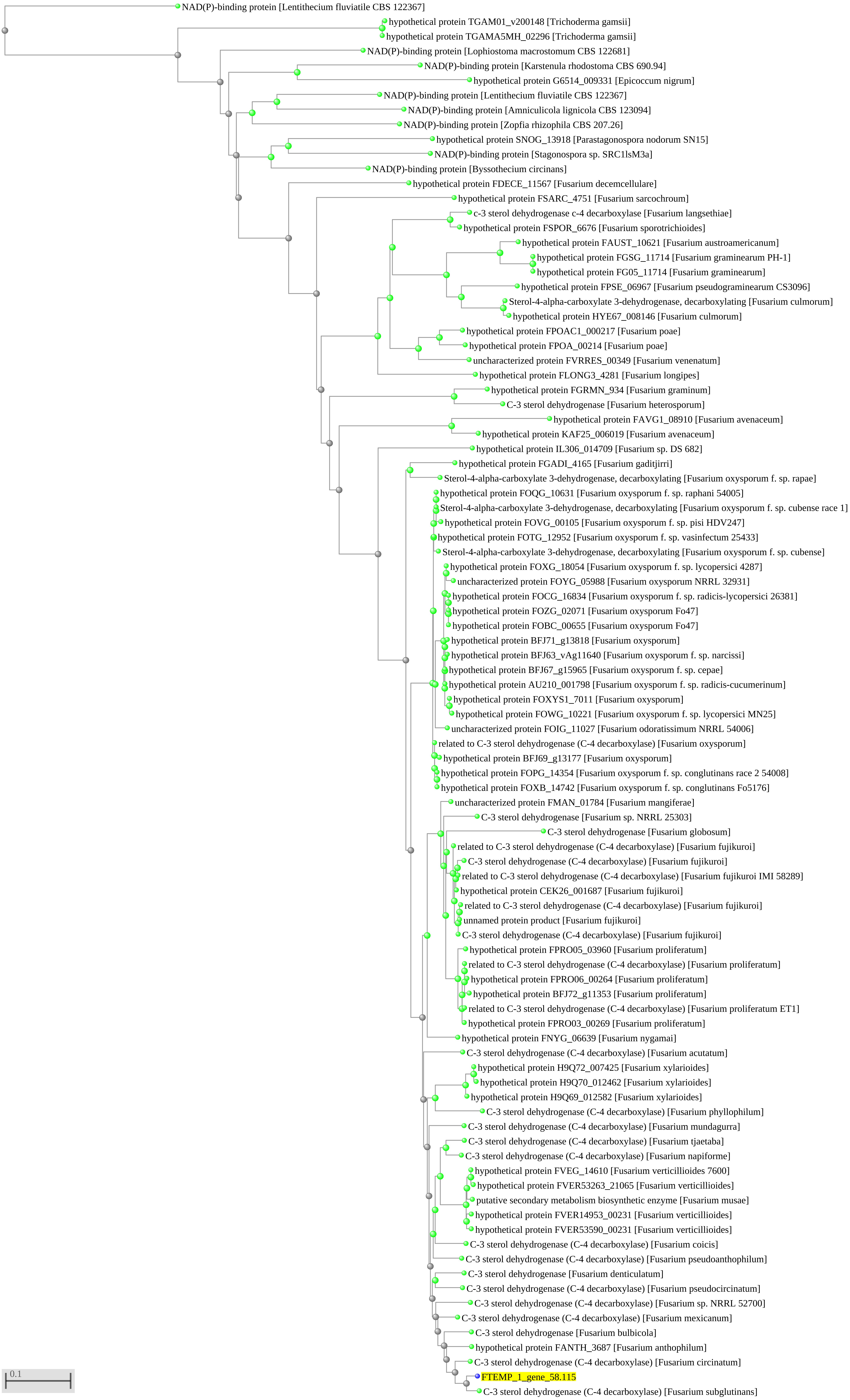
NAD(P)-binding protein [Lentithecium fluviatile CBS 122367]
hypothetical protein TGAM01_v200148 [Trichoderma gamsii]
hypothetical protein TGAMA5MH_02296 [Trichoderma gamsii]
NAD(P)-binding protein [Lophiostoma macrostomum CBS 122681]
NAD(P)-binding protein [Karstenula rhodostoma CBS 690.94]
hypothetical protein G6514_009331 [Epicoccum nigrum]
NAD(P)-binding protein [Lentithecium fluviatile CBS 122367]
NAD(P)-binding protein [Amniculicola lignicola CBS 123094]
NAD(P)-binding protein [Zopfia rhizophila CBS 207.26]
hypothetical protein SNOG_13918 [Parastagonospora nodorum SN15]
NAD(P)-binding protein [Stagonospora sp. SRC1lsM3a]
NAD(P)-binding protein [Byssothecium circinans]
hypothetical protein FDECE_11567 [Fusarium decemcellulare]
hypothetical protein FSARC_4751 [Fusarium sarcochroum]
c-3 sterol dehydrogenase c-4 decarboxylase [Fusarium langsethiae]
hypothetical protein FSPOR_6676 [Fusarium sporotrichioides]
hypothetical protein FAUST_10621 [Fusarium austroamericanum]
hypothetical protein FGSG_11714 [Fusarium graminearum PH-1]
hypothetical protein FG05_11714 [Fusarium graminearum]
hypothetical protein FPSE_06967 [Fusarium pseudograminearum CS3096]
Sterol-4-alpha-carboxylate 3-dehydrogenase, decarboxylating [Fusarium culmorum]
hypothetical protein HYE67_008146 [Fusarium culmorum]
hypothetical protein FPOAC1_000217 [Fusarium poae]
hypothetical protein FPOA_00214 [Fusarium poae]
uncharacterized protein FVRRES_00349 [Fusarium venenatum]
hypothetical protein FLONG3_4281 [Fusarium longipes]
hypothetical protein FGRMN_934 [Fusarium graminum]
C-3 sterol dehydrogenase [Fusarium heterosporum]
hypothetical protein FAVG1_08910 [Fusarium avenaceum]
hypothetical protein KAF25_006019 [Fusarium avenaceum]
hypothetical protein IL306_014709 [Fusarium sp. DS 682]
hypothetical protein FGADI_4165 [Fusarium gaditjirri]
Sterol-4-alpha-carboxylate 3-dehydrogenase, decarboxylating [Fusarium oxysporum f. sp. rapae]
hypothetical protein FOQG_10631 [Fusarium oxysporum f. sp. raphani 54005]
Sterol-4-alpha-carboxylate 3-dehydrogenase, decarboxylating [Fusarium oxysporum f. sp. cubense race 1]
hypothetical protein FOVG_00105 [Fusarium oxysporum f. sp. pisi HDV247]
hypothetical protein FOTG_12952 [Fusarium oxysporum f. sp. vasinfectum 25433]
Sterol-4-alpha-carboxylate 3-dehydrogenase, decarboxylating [Fusarium oxysporum f. sp. cubense]
hypothetical protein FOXG_18054 [Fusarium oxysporum f. sp. lycopersici 4287]
uncharacterized protein FOYG_05988 [Fusarium oxysporum NRRL 32931]
hypothetical protein FOCG_16834 [Fusarium oxysporum f. sp. radicis-lycopersici 26381]
hypothetical protein FOZG_02071 [Fusarium oxysporum Fo47]
hypothetical protein FOBC_00655 [Fusarium oxysporum Fo47]
hypothetical protein BFJ71_g13818 [Fusarium oxysporum]
hypothetical protein BFJ63_vAg11640 [Fusarium oxysporum f. sp. narcissi]
hypothetical protein BFJ67_g15965 [Fusarium oxysporum f. sp. cepae]
hypothetical protein AU210_001798 [Fusarium oxysporum f. sp. radicis-cucumerinum]
hypothetical protein FOXYS1_7011 [Fusarium oxysporum]
hypothetical protein FOWG_10221 [Fusarium oxysporum f. sp. lycopersici MN25]
uncharacterized protein FOIG_11027 [Fusarium odoratissimum NRRL 54006]
related to C-3 sterol dehydrogenase (C-4 decarboxylase) [Fusarium oxysporum]
hypothetical protein BFJ69_g13177 [Fusarium oxysporum]
hypothetical protein FOPG_14354 [Fusarium oxysporum f. sp. conglutinans race 2 54008]
hypothetical protein FOXB_14742 [Fusarium oxysporum f. sp. conglutinans Fo5176]
uncharacterized protein FMAN_01784 [Fusarium mangiferae]
C-3 sterol dehydrogenase [Fusarium sp. NRRL 25303]
C-3 sterol dehydrogenase [Fusarium globosum]
related to C-3 sterol dehydrogenase (C-4 decarboxylase) [Fusarium fujikuroi]
C-3 sterol dehydrogenase (C-4 decarboxylase) [Fusarium fujikuroi]
related to C-3 sterol dehydrogenase (C-4 decarboxylase) [Fusarium fujikuroi IMI 58289]
hypothetical protein CEK26_001687 [Fusarium fujikuroi]
related to C-3 sterol dehydrogenase (C-4 decarboxylase) [Fusarium fujikuroi]
unnamed protein product [Fusarium fujikuroi]
C-3 sterol dehydrogenase (C-4 decarboxylase) [Fusarium fujikuroi]
hypothetical protein FPRO05_03960 [Fusarium proliferatum]
related to C-3 sterol dehydrogenase (C-4 decarboxylase) [Fusarium proliferatum]
hypothetical protein FPRO06_00264 [Fusarium proliferatum]
hypothetical protein BFJ72_g11353 [Fusarium proliferatum]
related to C-3 sterol dehydrogenase (C-4 decarboxylase) [Fusarium proliferatum ET1]
hypothetical protein FPRO03_00269 [Fusarium proliferatum]
hypothetical protein FNYG_06639 [Fusarium nygamai]
C-3 sterol dehydrogenase (C-4 decarboxylase) [Fusarium acutatum]
hypothetical protein H9Q72_007425 [Fusarium xylarioides]
hypothetical protein H9Q70_012462 [Fusarium xylarioides]
hypothetical protein H9Q69_012582 [Fusarium xylarioides]
C-3 sterol dehydrogenase (C-4 decarboxylase) [Fusarium phyllophilum]
C-3 sterol dehydrogenase (C-4 decarboxylase) [Fusarium mundagurra]
C-3 sterol dehydrogenase (C-4 decarboxylase) [Fusarium tjaetaba]
C-3 sterol dehydrogenase (C-4 decarboxylase) [Fusarium napiforme]
hypothetical protein FVEG_14610 [Fusarium verticillioides 7600]
hypothetical protein FVER53263_21065 [Fusarium verticillioides]
putative secondary metabolism biosynthetic enzyme [Fusarium musae]
hypothetical protein FVER14953_00231 [Fusarium verticillioides]
hypothetical protein FVER53590_00231 [Fusarium verticillioides]
C-3 sterol dehydrogenase (C-4 decarboxylase) [Fusarium coicis]
C-3 sterol dehydrogenase (C-4 decarboxylase) [Fusarium pseudoanthophilum]
C-3 sterol dehydrogenase [Fusarium denticulatum]
C-3 sterol dehydrogenase (C-4 decarboxylase) [Fusarium pseudocircinatum]
C-3 sterol dehydrogenase (C-4 decarboxylase) [Fusarium sp. NRRL 52700]
C-3 sterol dehydrogenase (C-4 decarboxylase) [Fusarium mexicanum]
C-3 sterol dehydrogenase [Fusarium bulbicola]
hypothetical protein FANTH_3687 [Fusarium anthophilum]
C-3 sterol dehydrogenase (C-4 decarboxylase) [Fusarium circinatum]
FTEMP_1_gene_58.115
C-3 sterol dehydrogenase (C-4 decarboxylase) [Fusarium subglutinans]
0.1

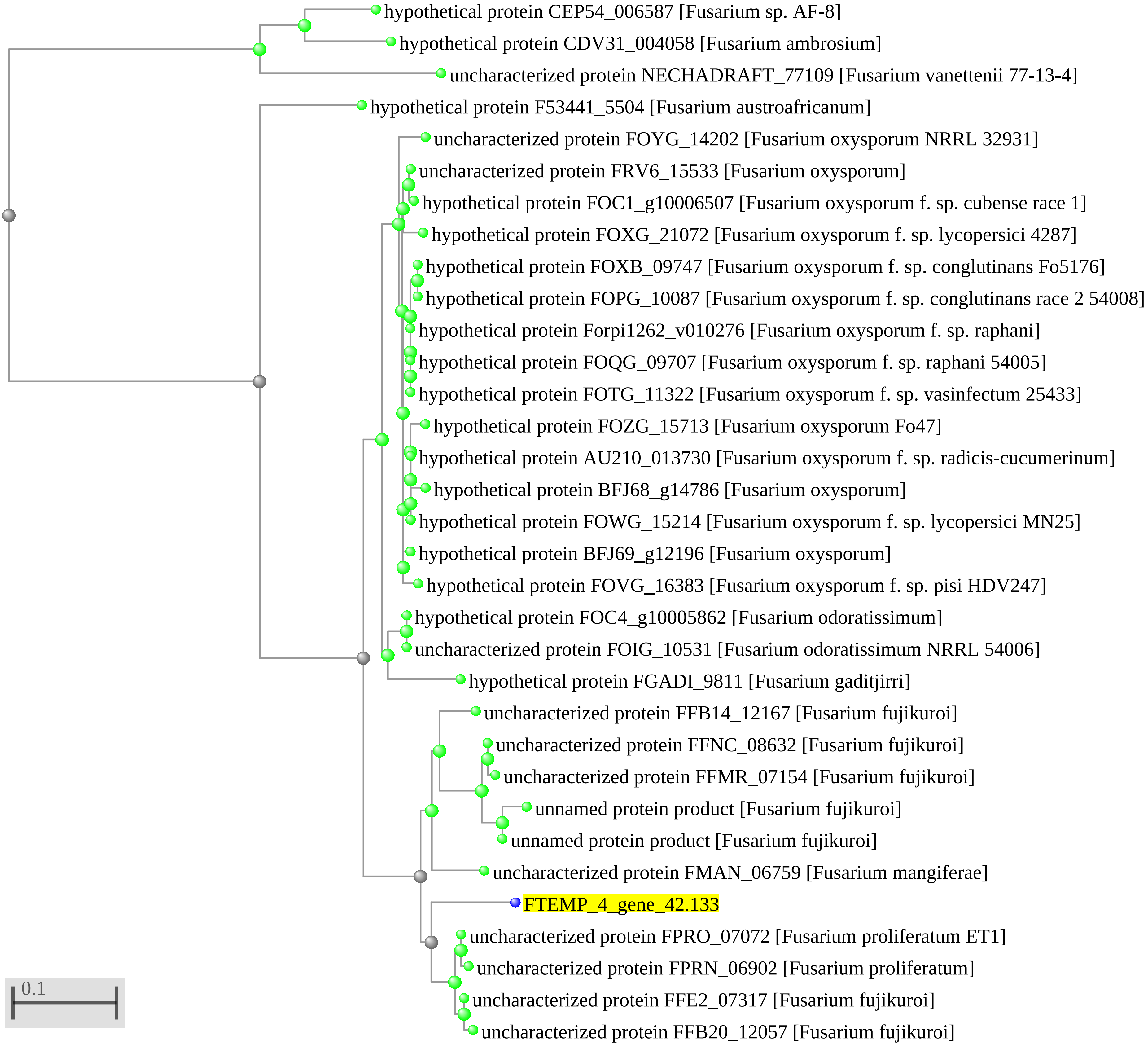
hypothetical protein CEP54_006587 [Fusarium sp. AF-8]
hypothetical protein CDV31_004058 [Fusarium ambrosium]
uncharacterized protein NECHADRAFT_77109 [Fusarium vanettenii 77-13-4]
hypothetical protein F53441_5504 [Fusarium austroafricanum]
uncharacterized protein FOYG_14202 [Fusarium oxysporum NRRL 32931]
uncharacterized protein FRV6_15533 [Fusarium oxysporum]
hypothetical protein FOC1_g10006507 [Fusarium oxysporum f. sp. cubense race 1]
hypothetical protein FOXG_21072 [Fusarium oxysporum f. sp. lycopersici 4287]
hypothetical protein FOXB_09747 [Fusarium oxysporum f. sp. conglutinans Fo5176]
hypothetical protein FOPG_10087 [Fusarium oxysporum f. sp. conglutinans race 2 54008]
hypothetical protein Forpi1262_v010276 [Fusarium oxysporum f. sp. raphani]
hypothetical protein FOQG_09707 [Fusarium oxysporum f. sp. raphani 54005]
hypothetical protein FOTG_11322 [Fusarium oxysporum f. sp. vasinfectum 25433]
hypothetical protein FOZG_15713 [Fusarium oxysporum Fo47]
hypothetical protein AU210_013730 [Fusarium oxysporum f. sp. radicis-cucumerinum]
hypothetical protein BFJ68_g14786 [Fusarium oxysporum]
hypothetical protein FOWG_15214 [Fusarium oxysporum f. sp. lycopersici MN25]
hypothetical protein BFJ69_g12196 [Fusarium oxysporum]
hypothetical protein FOVG_16383 [Fusarium oxysporum f. sp. pisi HDV247]
hypothetical protein FOC4_g10005862 [Fusarium odoratissimum]
uncharacterized protein FOIG_10531 [Fusarium odoratissimum NRRL 54006]
hypothetical protein FGADI_9811 [Fusarium gaditjirri]
uncharacterized protein FFB14_12167 [Fusarium fujikuroi]
uncharacterized protein FFNC_08632 [Fusarium fujikuroi]
uncharacterized protein FFMR_07154 [Fusarium fujikuroi]
unnamed protein product [Fusarium fujikuroi]
unnamed protein product [Fusarium fujikuroi]
uncharacterized protein FMAN_06759 [Fusarium mangiferae]
FTEMP_4_gene_42.133
uncharacterized protein FPRO_07072 [Fusarium proliferatum ET1]
uncharacterized protein FPRN_06902 [Fusarium proliferatum]
uncharacterized protein FFE2_07317 [Fusarium fujikuroi]
uncharacterized protein FFB20_12057 [Fusarium fujikuroi]
0.1

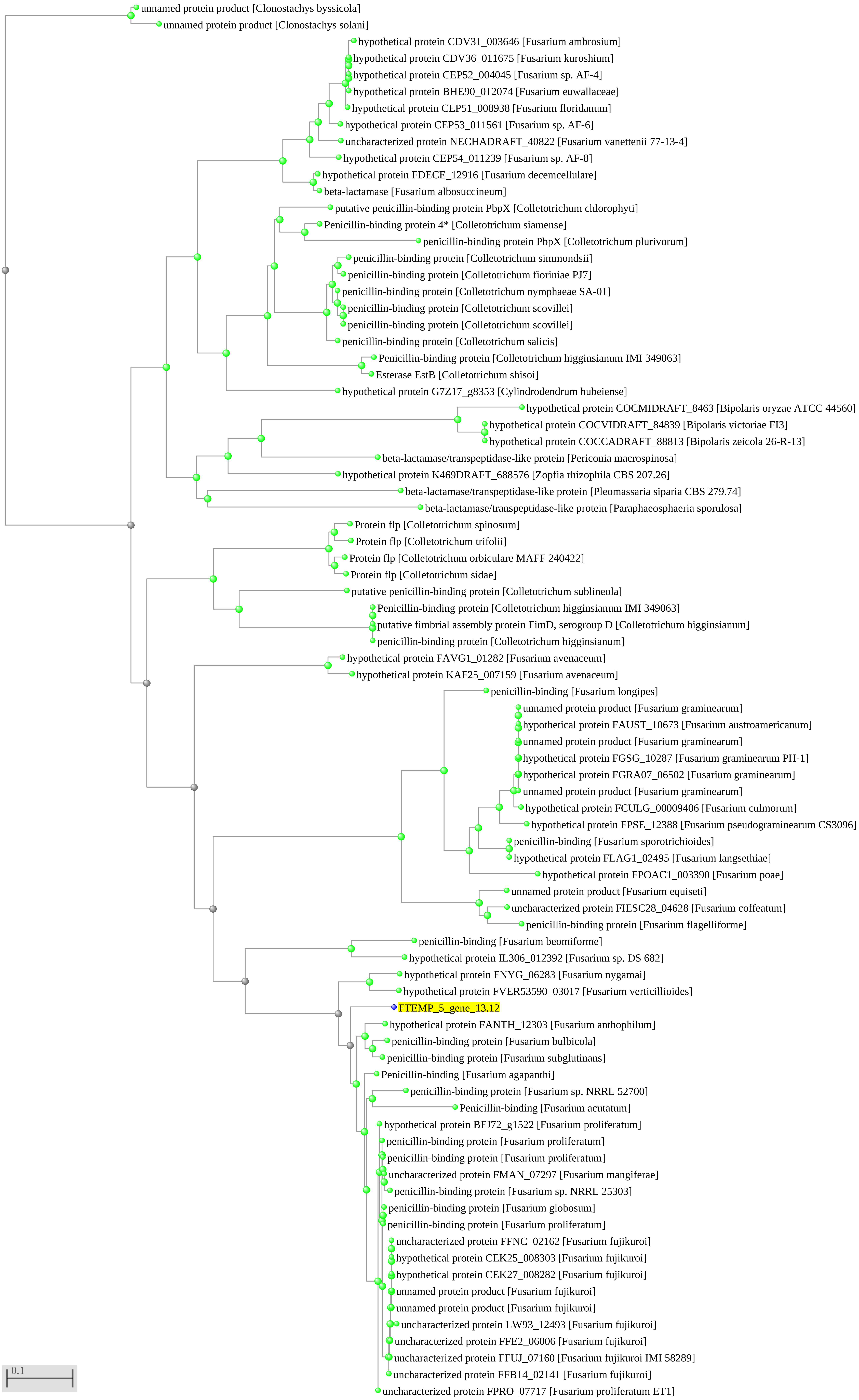
unnamed protein product [Clonostachys byssicola]
unnamed protein product [Clonostachys solani]
hypothetical protein CDV31_003646 [Fusarium ambrosium]
hypothetical protein CDV36_011675 [Fusarium kuroshium]
hypothetical protein CEP52_004045 [Fusarium sp. AF-4]
hypothetical protein BHE90_012074 [Fusarium euwallaceae]
hypothetical protein CEP51_008938 [Fusarium floridanum]
hypothetical protein CEP53_011561 [Fusarium sp. AF-6]
uncharacterized protein NECHADRAFT_40822 [Fusarium vanettenii 77-13-4]
hypothetical protein CEP54_011239 [Fusarium sp. AF-8]
hypothetical protein FDECE_12916 [Fusarium decemcellulare]
beta-lactamase [Fusarium albosuccineum]
putative penicillin-binding protein PbpX [Colletotrichum chlorophyti]
Penicillin-binding protein 4* [Colletotrichum siamense]
penicillin-binding protein PbpX [Colletotrichum plurivorum]
penicillin-binding protein [Colletotrichum simmondsii]
penicillin-binding protein [Colletotrichum fioriniae PJ7]
penicillin-binding protein [Colletotrichum nymphaeae SA-01]
penicillin-binding protein [Colletotrichum scovillei]
penicillin-binding protein [Colletotrichum scovillei]
penicillin-binding protein [Colletotrichum salicis]
Penicillin-binding protein [Colletotrichum higginsianum IMI 349063]
Esterase EstB [Colletotrichum shisoi]
hypothetical protein G7Z17_g8353 [Cylindrodendrum hubeiense]
hypothetical protein COCMIDRAFT_8463 [Bipolaris oryzae ATCC 44560]
hypothetical protein COCVIDRAFT_84839 [Bipolaris victoriae FI3]
hypothetical protein COCCADRAFT_88813 [Bipolaris zeicola 26-R-13]
beta-lactamase/transpeptidase-like protein [Periconia macrospinosa]
hypothetical protein K469DRAFT_688576 [Zopfia rhizophila CBS 207.26]
beta-lactamase/transpeptidase-like protein [Pleomassaria siparia CBS 279.74]
beta-lactamase/transpeptidase-like protein [Paraphaeosphaeria sporulosa]
Protein flp [Colletotrichum spinosum]
Protein flp [Colletotrichum trifolii]
Protein flp [Colletotrichum orbiculare MAFF 240422]
Protein flp [Colletotrichum sidae]
putative penicillin-binding protein [Colletotrichum sublineola]
Penicillin-binding protein [Colletotrichum higginsianum IMI 349063]
putative fimbrial assembly protein FimD, serogroup D [Colletotrichum higginsianum]
penicillin-binding protein [Colletotrichum higginsianum]
hypothetical protein FAVG1_01282 [Fusarium avenaceum]
hypothetical protein KAF25_007159 [Fusarium avenaceum]
penicillin-binding [Fusarium longipes]
unnamed protein product [Fusarium graminearum]
hypothetical protein FAUST_10673 [Fusarium austroamericanum]
unnamed protein product [Fusarium graminearum]
hypothetical protein FGSG_10287 [Fusarium graminearum PH-1]
hypothetical protein FGRA07_06502 [Fusarium graminearum]
unnamed protein product [Fusarium graminearum]
hypothetical protein FCULG_00009406 [Fusarium culmorum]
hypothetical protein FPSE_12388 [Fusarium pseudograminearum CS3096]
penicillin-binding [Fusarium sporotrichioides]
hypothetical protein FLAG1_02495 [Fusarium langsethiae]
hypothetical protein FPOAC1_003390 [Fusarium poae]
unnamed protein product [Fusarium equiseti]
uncharacterized protein FIESC28_04628 [Fusarium coffeatum]
penicillin-binding protein [Fusarium flagelliforme]
penicillin-binding [Fusarium beomiforme]
hypothetical protein IL306_012392 [Fusarium sp. DS 682]
hypothetical protein FNYG_06283 [Fusarium nygamai]
hypothetical protein FVER53590_03017 [Fusarium verticillioides]
FTEMP_5_gene_13.12
hypothetical protein FANTH_12303 [Fusarium anthophilum]
penicillin-binding protein [Fusarium bulbicola]
penicillin-binding protein [Fusarium subglutinans]
Penicillin-binding [Fusarium agapanthi]
penicillin-binding protein [Fusarium sp. NRRL 52700]
Penicillin-binding [Fusarium acutatum]
hypothetical protein BFJ72_g1522 [Fusarium proliferatum]
penicillin-binding protein [Fusarium proliferatum]
penicillin-binding protein [Fusarium proliferatum]
uncharacterized protein FMAN_07297 [Fusarium mangiferae]
penicillin-binding protein [Fusarium sp. NRRL 25303]
penicillin-binding protein [Fusarium globosum]
penicillin-binding protein [Fusarium proliferatum]
uncharacterized protein FFNC_02162 [Fusarium fujikuroi]
hypothetical protein CEK25_008303 [Fusarium fujikuroi]
hypothetical protein CEK27_008282 [Fusarium fujikuroi]
unnamed protein product [Fusarium fujikuroi]
unnamed protein product [Fusarium fujikuroi]
uncharacterized protein LW93_12493 [Fusarium fujikuroi]
uncharacterized protein FFE2_06006 [Fusarium fujikuroi]
uncharacterized protein FFUJ_07160 [Fusarium fujikuroi IMI 58289]
uncharacterized protein FFB14_02141 [Fusarium fujikuroi]
uncharacterized protein FPRO_07717 [Fusarium proliferatum ET1]
0.1

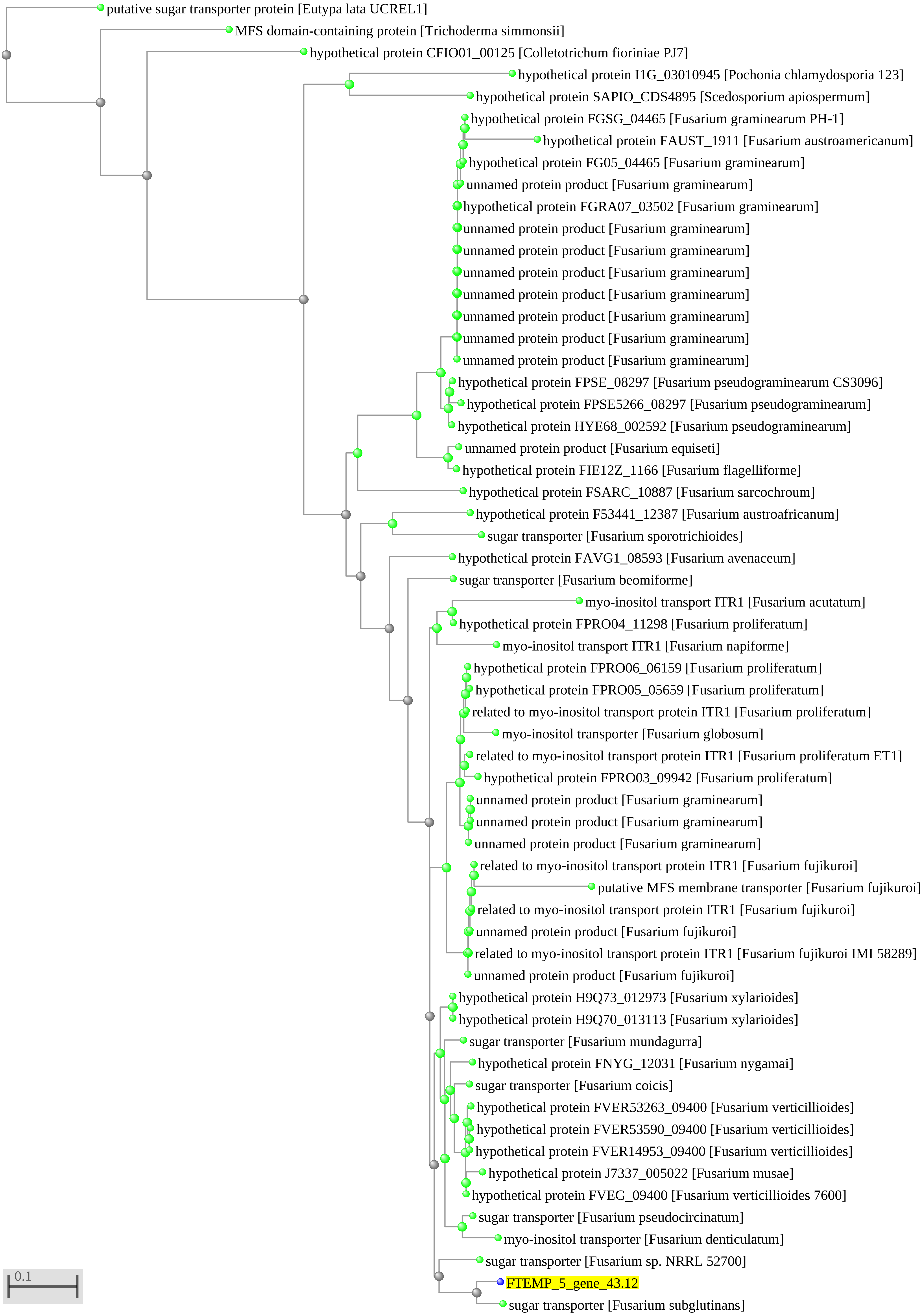
putative sugar transporter protein [Eutypa lata UCREL1]
MFS domain-containing protein [Trichoderma simmonsii]
hypothetical protein CFIO01_00125 [Colletotrichum fioriniae PJ7]
hypothetical protein I1G_03010945 [Pochonia chlamydosporia 123]
hypothetical protein SAPIO_CDS4895 [Scedosporium apiospermum]
hypothetical protein FGSG_04465 [Fusarium graminearum PH-1]
hypothetical protein FAUST_1911 [Fusarium austroamericanum]
hypothetical protein FG05_04465 [Fusarium graminearum]
unnamed protein product [Fusarium graminearum]
hypothetical protein FGRA07_03502 [Fusarium graminearum]
unnamed protein product [Fusarium graminearum]
unnamed protein product [Fusarium graminearum]
unnamed protein product [Fusarium graminearum]
unnamed protein product [Fusarium graminearum]
unnamed protein product [Fusarium graminearum]
unnamed protein product [Fusarium graminearum]
unnamed protein product [Fusarium graminearum]
hypothetical protein FPSE_08297 [Fusarium pseudograminearum CS3096]
hypothetical protein FPSE5266_08297 [Fusarium pseudograminearum]
hypothetical protein HYE68_002592 [Fusarium pseudograminearum]
unnamed protein product [Fusarium equiseti]
hypothetical protein FIE12Z_1166 [Fusarium flagelliforme]
hypothetical protein FSARC_10887 [Fusarium sarcochroum]
hypothetical protein F53441_12387 [Fusarium austroafricanum]
sugar transporter [Fusarium sporotrichioides]
hypothetical protein FAVG1_08593 [Fusarium avenaceum]
sugar transporter [Fusarium beomiforme]
myo-inositol transport ITR1 [Fusarium acutatum]
hypothetical protein FPRO04_11298 [Fusarium proliferatum]
myo-inositol transport ITR1 [Fusarium napiforme]
hypothetical protein FPRO06_06159 [Fusarium proliferatum]
hypothetical protein FPRO05_05659 [Fusarium proliferatum]
related to myo-inositol transport protein ITR1 [Fusarium proliferatum]
myo-inositol transporter [Fusarium globosum]
related to myo-inositol transport protein ITR1 [Fusarium proliferatum ET1]
hypothetical protein FPRO03_09942 [Fusarium proliferatum]
unnamed protein product [Fusarium graminearum]
unnamed protein product [Fusarium graminearum]
unnamed protein product [Fusarium graminearum]
related to myo-inositol transport protein ITR1 [Fusarium fujikuroi]
putative MFS membrane transporter [Fusarium fujikuroi]
related to myo-inositol transport protein ITR1 [Fusarium fujikuroi]
unnamed protein product [Fusarium fujikuroi]
related to myo-inositol transport protein ITR1 [Fusarium fujikuroi IMI 58289]
unnamed protein product [Fusarium fujikuroi]
hypothetical protein H9Q73_012973 [Fusarium xylarioides]
hypothetical protein H9Q70_013113 [Fusarium xylarioides]
sugar transporter [Fusarium mundagurra]
hypothetical protein FNYG_12031 [Fusarium nygamai]
sugar transporter [Fusarium coicis]
hypothetical protein FVER53263_09400 [Fusarium verticillioides]
hypothetical protein FVER53590_09400 [Fusarium verticillioides]
hypothetical protein FVER14953_09400 [Fusarium verticillioides]
hypothetical protein J7337_005022 [Fusarium musae]
hypothetical protein FVEG_09400 [Fusarium verticillioides 7600]
sugar transporter [Fusarium pseudocircinatum]
myo-inositol transporter [Fusarium denticulatum]
sugar transporter [Fusarium sp. NRRL 52700]
FTEMP_5_gene_43.12
sugar transporter [Fusarium subglutinans]
0.1

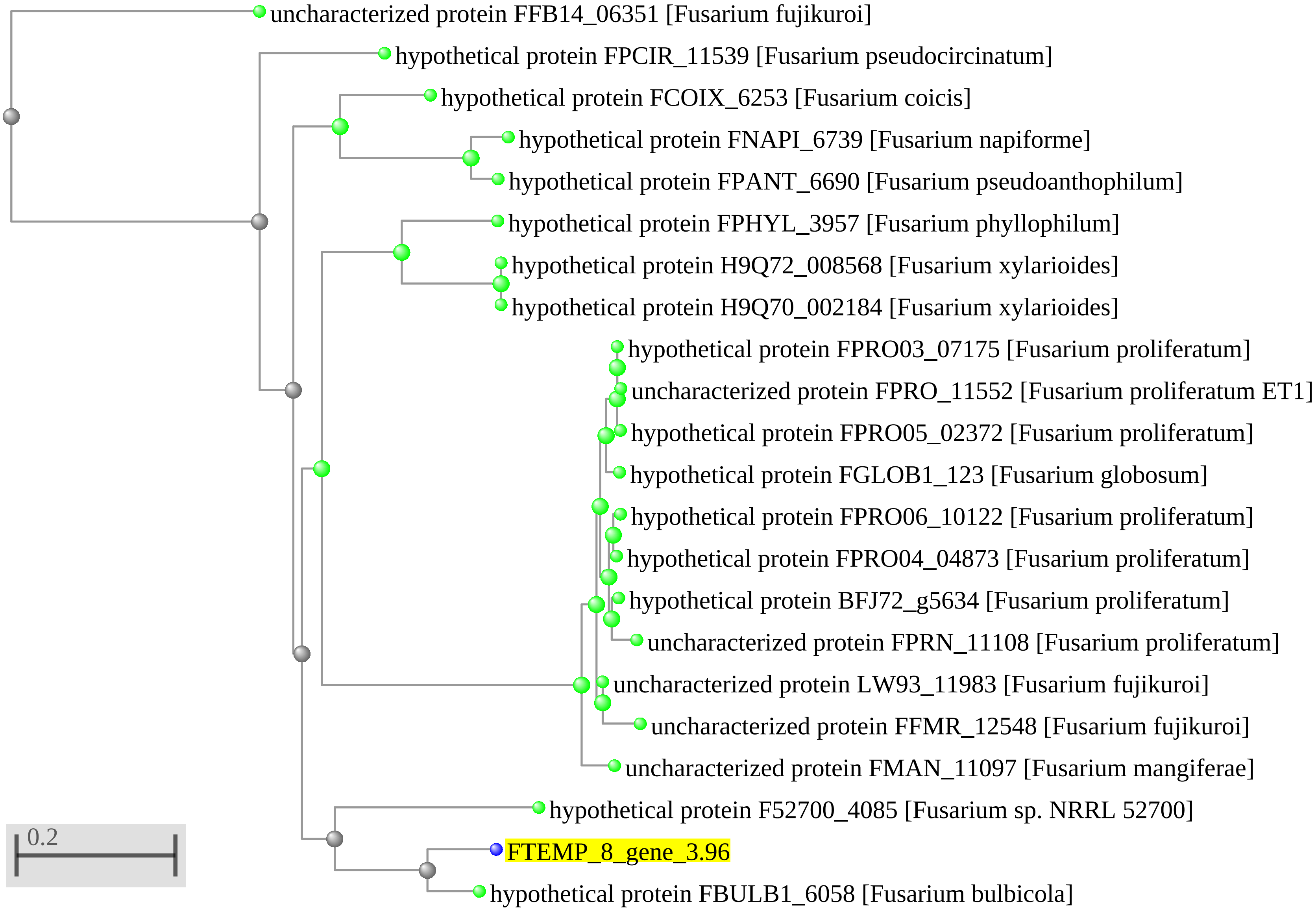
uncharacterized protein FFB14_06351 [Fusarium fujikuroi]
hypothetical protein FPCIR_11539 [Fusarium pseudocircinatum]
hypothetical protein FCOIX_6253 [Fusarium coicis]
hypothetical protein FNAPI_6739 [Fusarium napiforme]
hypothetical protein FPANT_6690 [Fusarium pseudoanthophilum]
hypothetical protein FPHYL_3957 [Fusarium phyllophilum]
hypothetical protein H9Q72_008568 [Fusarium xylarioides]
hypothetical protein H9Q70_002184 [Fusarium xylarioides]
hypothetical protein FPRO03_07175 [Fusarium proliferatum]
uncharacterized protein FPRO_11552 [Fusarium proliferatum ET1]
hypothetical protein FPRO05_02372 [Fusarium proliferatum]
hypothetical protein FGLOB1_123 [Fusarium globosum]
hypothetical protein FPRO06_10122 [Fusarium proliferatum]
hypothetical protein FPRO04_04873 [Fusarium proliferatum]
hypothetical protein BFJ72_g5634 [Fusarium proliferatum]
uncharacterized protein FPRN_11108 [Fusarium proliferatum]
uncharacterized protein LW93_11983 [Fusarium fujikuroi]
uncharacterized protein FFMR_12548 [Fusarium fujikuroi]
uncharacterized protein FMAN_11097 [Fusarium mangiferae]
hypothetical protein F52700_4085 [Fusarium sp. NRRL 52700]
FTEMP_8_gene_3.96
hypothetical protein FBULB1_6058 [Fusarium bulbicola]
0.2

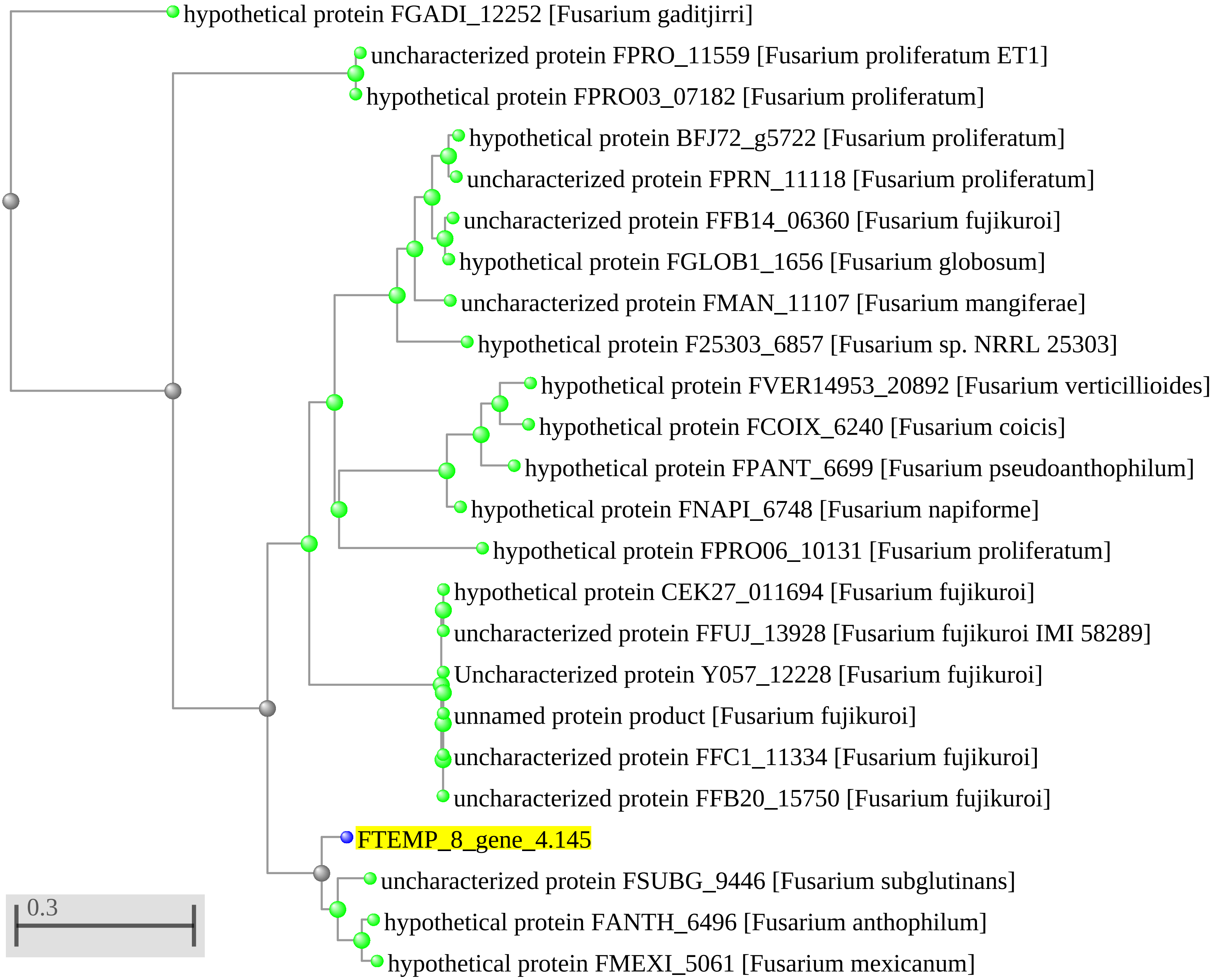
hypothetical protein FGADI_12252 [Fusarium gaditjirri]
uncharacterized protein FPRO_11559 [Fusarium proliferatum ET1]
hypothetical protein FPRO03_07182 [Fusarium proliferatum]
hypothetical protein BFJ72_g5722 [Fusarium proliferatum]
uncharacterized protein FPRN_11118 [Fusarium proliferatum]
uncharacterized protein FFB14_06360 [Fusarium fujikuroi]
hypothetical protein FGLOB1_1656 [Fusarium globosum]
uncharacterized protein FMAN_11107 [Fusarium mangiferae]
hypothetical protein F25303_6857 [Fusarium sp. NRRL 25303]
hypothetical protein FVER14953_20892 [Fusarium verticillioides]
hypothetical protein FCOIX_6240 [Fusarium coicis]
hypothetical protein FPANT_6699 [Fusarium pseudoanthophilum]
hypothetical protein FNAPI_6748 [Fusarium napiforme]
hypothetical protein FPRO06_10131 [Fusarium proliferatum]
hypothetical protein CEK27_011694 [Fusarium fujikuroi]
uncharacterized protein FFUJ_13928 [Fusarium fujikuroi IMI 58289]
Uncharacterized protein Y057_12228 [Fusarium fujikuroi]
unnamed protein product [Fusarium fujikuroi]
uncharacterized protein FFC1_11334 [Fusarium fujikuroi]
uncharacterized protein FFB20_15750 [Fusarium fujikuroi]
FTEMP_8_gene_4.145
uncharacterized protein FSUBG_9446 [Fusarium subglutinans]
hypothetical protein FANTH_6496 [Fusarium anthophilum]
hypothetical protein FMEXI_5061 [Fusarium mexicanum]
0.3

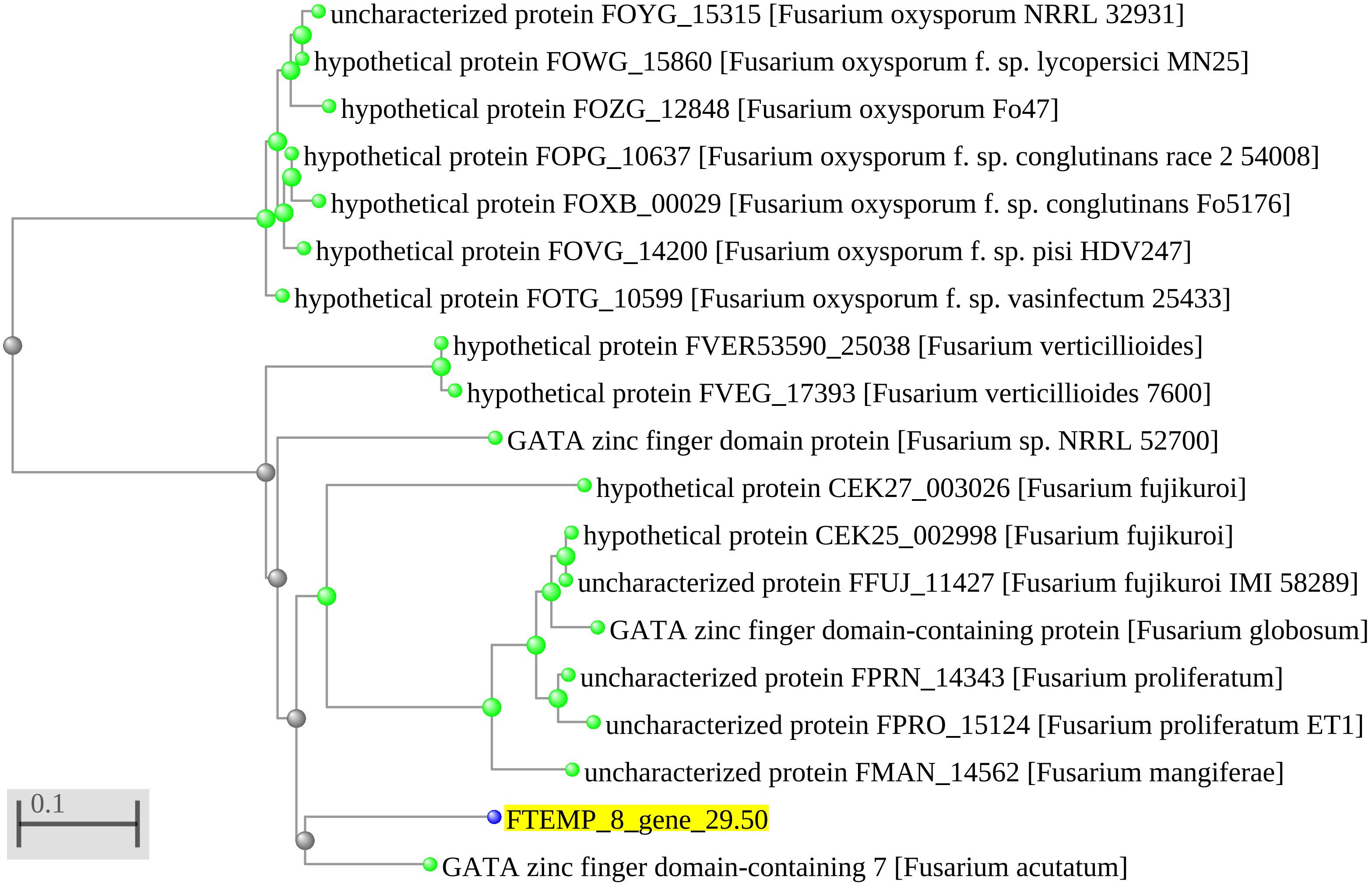
uncharacterized protein FOYG_15315 [Fusarium oxysporum NRRL 32931]
hypothetical protein FOWG_15860 [Fusarium oxysporum f. sp. lycopersici MN25]
hypothetical protein FOZG_12848 [Fusarium oxysporum Fo47]
hypothetical protein FOPG_10637 [Fusarium oxysporum f. sp. conglutinans race 2 54008]
hypothetical protein FOXB_00029 [Fusarium oxysporum f. sp. conglutinans Fo5176]
hypothetical protein FOVG_14200 [Fusarium oxysporum f. sp. pisi HDV247]
hypothetical protein FOTG_10599 [Fusarium oxysporum f. sp. vasinfectum 25433]
hypothetical protein FVER53590_25038 [Fusarium verticillioides]
hypothetical protein FVEG_17393 [Fusarium verticillioides 7600]
GATA zinc finger domain protein [Fusarium sp. NRRL 52700]
hypothetical protein CEK27_003026 [Fusarium fujikuroi]
hypothetical protein CEK25_002998 [Fusarium fujikuroi]
uncharacterized protein FFUJ_11427 [Fusarium fujikuroi IMI 58289]
GATA zinc finger domain-containing protein [Fusarium globosum]
uncharacterized protein FPRN_14343 [Fusarium proliferatum]
uncharacterized protein FPRO_15124 [Fusarium proliferatum ET1]
uncharacterized protein FMAN_14562 [Fusarium mangiferae]
0.1
FTEMP_8_gene_29.50
GATA zinc finger domain-containing 7 [Fusarium acutatum]

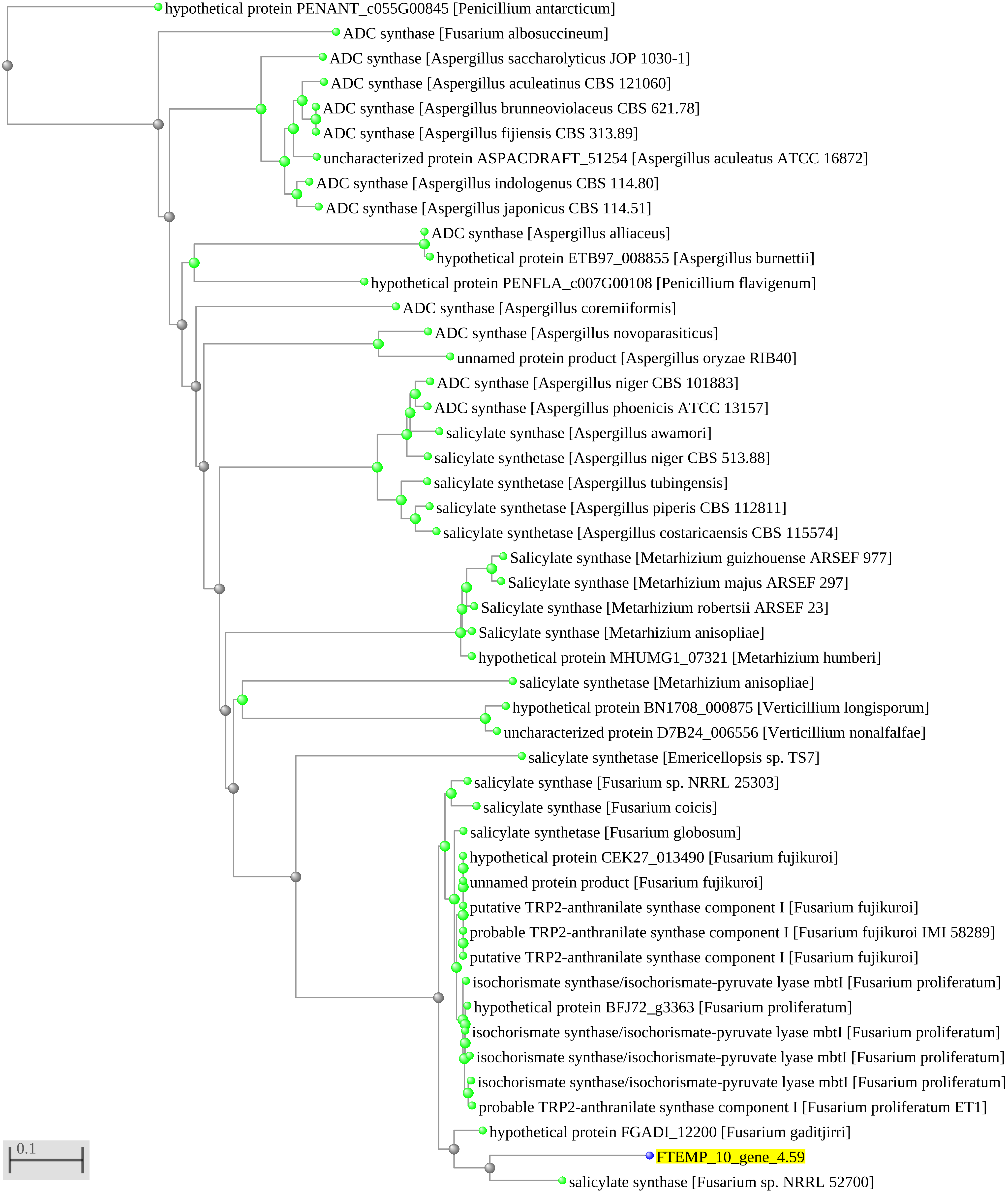
hypothetical protein PENANT_c055G00845 [Penicillium antarcticum]
ADC synthase [Fusarium albosuccineum]
ADC synthase [Aspergillus saccharolyticus JOP 1030-1]
ADC synthase [Aspergillus aculeatinus CBS 121060]
ADC synthase [Aspergillus brunneoviolaceus CBS 621.78]
ADC synthase [Aspergillus fijiensis CBS 313.89]
uncharacterized protein ASPACDRAFT_51254 [Aspergillus aculeatus ATCC 16872]
ADC synthase [Aspergillus indologenus CBS 114.80]
ADC synthase [Aspergillus japonicus CBS 114.51]
ADC synthase [Aspergillus alliaceus]
hypothetical protein ETB97_008855 [Aspergillus burnettii]
hypothetical protein PENFLA_c007G00108 [Penicillium flavigenum]
ADC synthase [Aspergillus coremiiformis]
ADC synthase [Aspergillus novoparasiticus]
unnamed protein product [Aspergillus oryzae RIB40]
ADC synthase [Aspergillus niger CBS 101883]
ADC synthase [Aspergillus phoenicis ATCC 13157]
salicylate synthase [Aspergillus awamori]
salicylate synthetase [Aspergillus niger CBS 513.88]
salicylate synthetase [Aspergillus tubingensis]
salicylate synthetase [Aspergillus piperis CBS 112811]
salicylate synthetase [Aspergillus costaricaensis CBS 115574]
Salicylate synthase [Metarhizium guizhouense ARSEF 977]
Salicylate synthase [Metarhizium majus ARSEF 297]
Salicylate synthase [Metarhizium robertsii ARSEF 23]
Salicylate synthase [Metarhizium anisopliae]
hypothetical protein MHUMG1_07321 [Metarhizium humberi]
salicylate synthetase [Metarhizium anisopliae]
hypothetical protein BN1708_000875 [Verticillium longisporum]
uncharacterized protein D7B24_006556 [Verticillium nonalfalfae]
salicylate synthetase [Emericellopsis sp. TS7]
salicylate synthase [Fusarium sp. NRRL 25303]
salicylate synthase [Fusarium coicis]
salicylate synthetase [Fusarium globosum]
hypothetical protein CEK27_013490 [Fusarium fujikuroi]
unnamed protein product [Fusarium fujikuroi]
putative TRP2-anthranilate synthase component I [Fusarium fujikuroi]
probable TRP2-anthranilate synthase component I [Fusarium fujikuroi IMI 58289]
putative TRP2-anthranilate synthase component I [Fusarium fujikuroi]
isochorismate synthase/isochorismate-pyruvate lyase mbtI [Fusarium proliferatum]
hypothetical protein BFJ72_g3363 [Fusarium proliferatum]
isochorismate synthase/isochorismate-pyruvate lyase mbtI [Fusarium proliferatum]
isochorismate synthase/isochorismate-pyruvate lyase mbtI [Fusarium proliferatum]
isochorismate synthase/isochorismate-pyruvate lyase mbtI [Fusarium proliferatum]
probable TRP2-anthranilate synthase component I [Fusarium proliferatum ET1]
hypothetical protein FGADI_12200 [Fusarium gaditjirri]
FTEMP_10_gene_4.59
salicylate synthase [Fusarium sp. NRRL 52700]
0.1

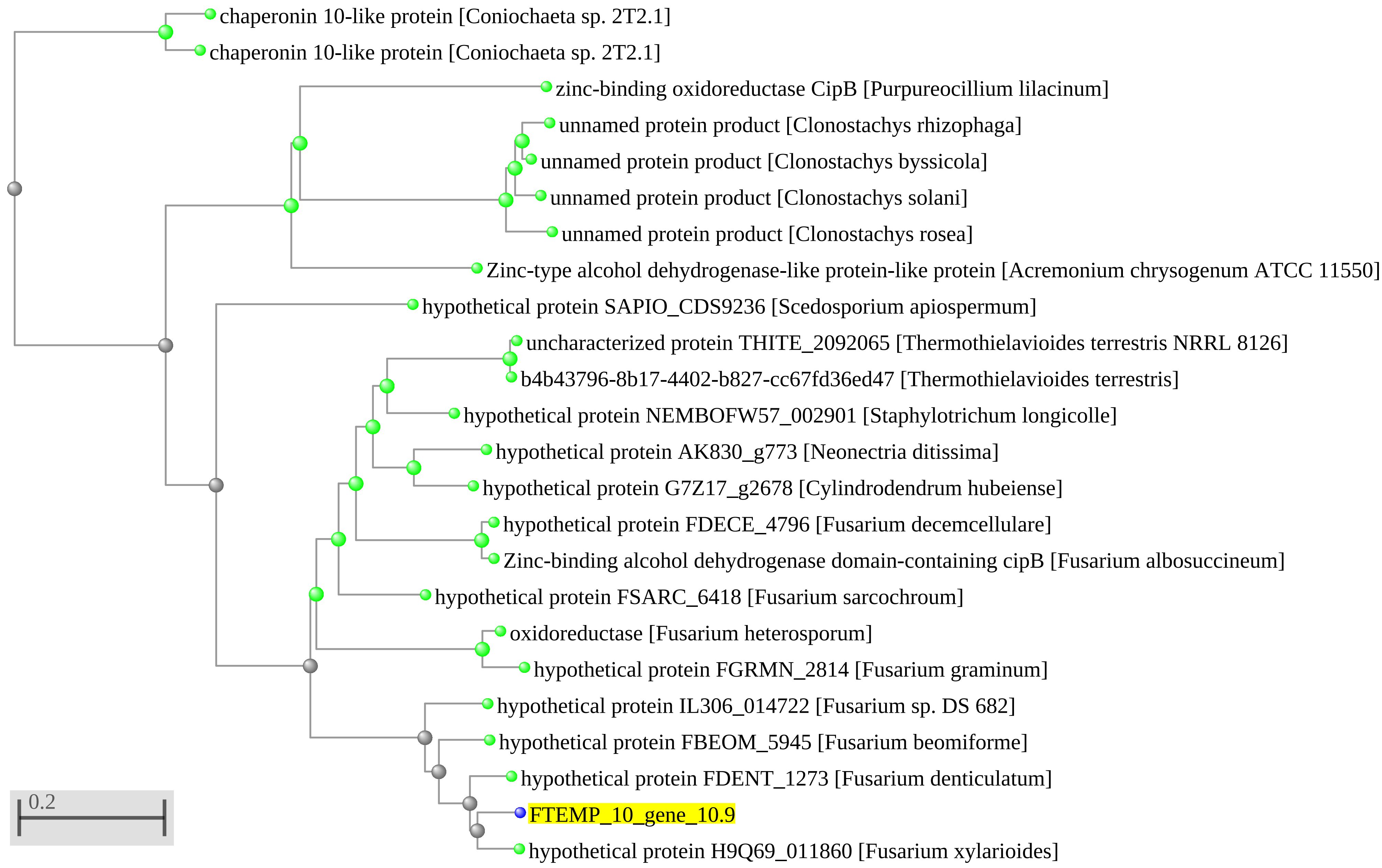

chaperonin 10-like protein [Coniochaeta sp. 2T2.1]
chaperonin 10-like protein [Coniochaeta sp. 2T2.1]
zinc-binding oxidoreductase CipB [Purpureocillium lilacinum]
unnamed protein product [Clonostachys rhizophaga]
unnamed protein product [Clonostachys byssicola]
unnamed protein product [Clonostachys solani]
unnamed protein product [Clonostachys rosea]
Zinc-type alcohol dehydrogenase-like protein-like protein [Acremonium chrysogenum ATCC 11550]
hypothetical protein SAPIO_CDS9236 [Scedosporium apiospermum]
uncharacterized protein THITE_2092065 [Thermothielavioides terrestris NRRL 8126]
b4b43796-8b17-4402-b827-cc67fd36ed47 [Thermothielavioides terrestris]
hypothetical protein NEMBOFW57_002901 [Staphylotrichum longicolle]
hypothetical protein AK830_g773 [Neonectria ditissima]
hypothetical protein G7Z17_g2678 [Cylindrodendrum hubeiense]
hypothetical protein FDECE_4796 [Fusarium decemcellulare]
Zinc-binding alcohol dehydrogenase domain-containing cipB [Fusarium albosuccineum]
hypothetical protein FSARC_6418 [Fusarium sarcochroum]
oxidoreductase [Fusarium heterosporum]
hypothetical protein FGRMN_2814 [Fusarium graminum]
hypothetical protein IL306_014722 [Fusarium sp. DS 682]
hypothetical protein FBEOM_5945 [Fusarium beomiforme]
hypothetical protein FDENT_1273 [Fusarium denticulatum]
FTEMP_10_gene_10.9
hypothetical protein H9Q69_011860 [Fusarium xylarioides]
0.2

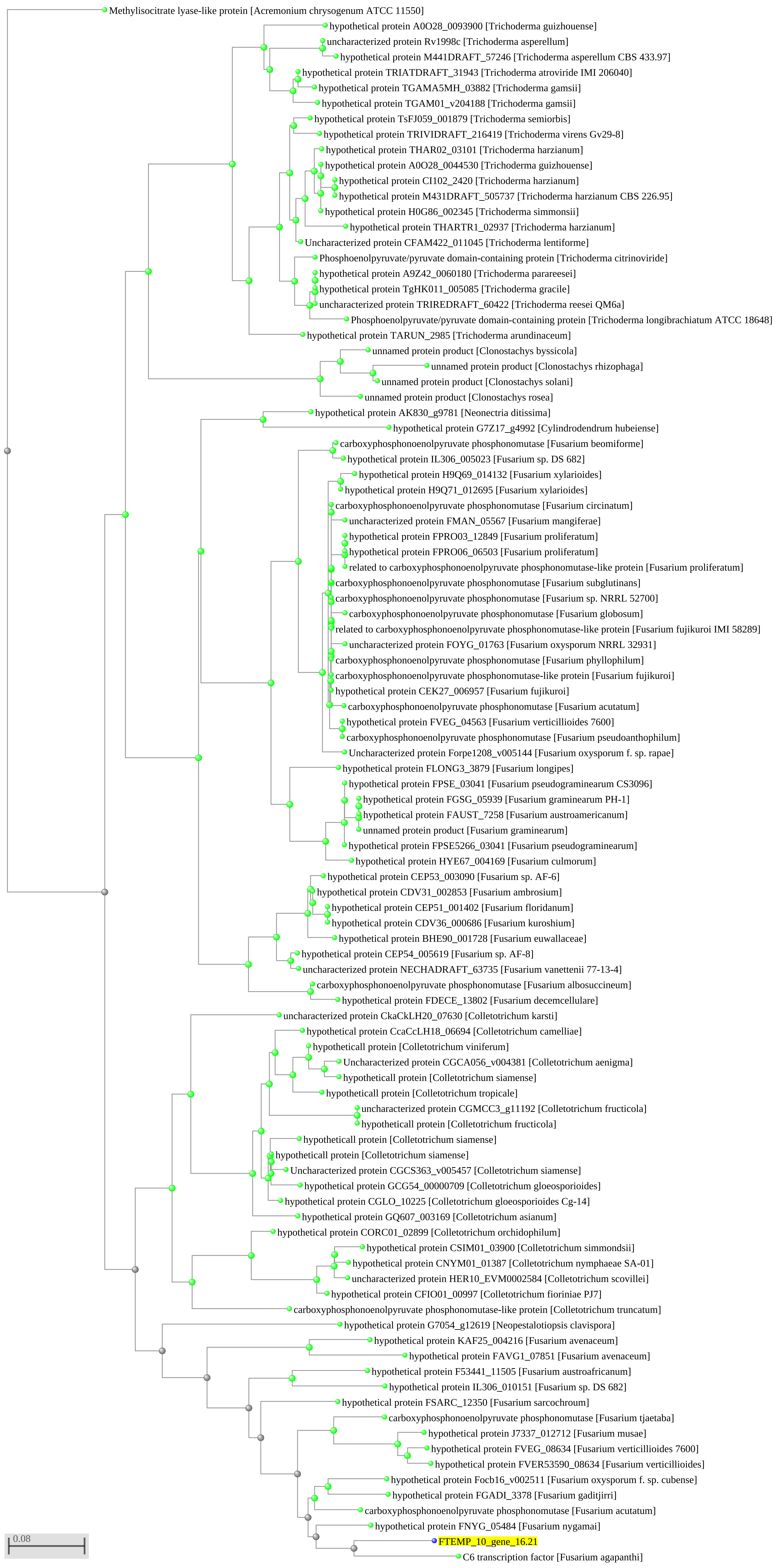
Methylisocitrate lyase-like protein [Acremonium chrysogenum ATCC 11550]
hypothetical protein A0O28_0093900 [Trichoderma guizhouense]
uncharacterized protein Rv1998c [Trichoderma asperellum]
hypothetical protein M441DRAFT_57246 [Trichoderma asperellum CBS 433.97]
hypothetical protein TRIATDRAFT_31943 [Trichoderma atroviride IMI 206040]
hypothetical protein TGAMA5MH_03882 [Trichoderma gamsii]
hypothetical protein TGAM01_v204188 [Trichoderma gamsii]
hypothetical protein TsFJ059_001879 [Trichoderma semiorbis]
hypothetical protein TRIVIDRAFT_216419 [Trichoderma virens Gv29-8]
hypothetical protein THAR02_03101 [Trichoderma harzianum]
hypothetical protein A0O28_0044530 [Trichoderma guizhouense]
hypothetical protein CI102_2420 [Trichoderma harzianum]
hypothetical protein M431DRAFT_505737 [Trichoderma harzianum CBS 226.95]
hypothetical protein H0G86_002345 [Trichoderma simmonsii]
hypothetical protein THARTR1_02937 [Trichoderma harzianum]
Uncharacterized protein CFAM422_011045 [Trichoderma lentiforme]
Phosphoenolpyruvate/pyruvate domain-containing protein [Trichoderma citrinoviride]
hypothetical protein A9Z42_0060180 [Trichoderma parareesei]
hypothetical protein TgHK011_005085 [Trichoderma gracile]
uncharacterized protein TRIREDRAFT_60422 [Trichoderma reesei QM6a]
Phosphoenolpyruvate/pyruvate domain-containing protein [Trichoderma longibrachiatum ATCC 18648]
hypothetical protein TARUN_2985 [Trichoderma arundinaceum]
unnamed protein product [Clonostachys byssicola]
unnamed protein product [Clonostachys rhizophaga]
unnamed protein product [Clonostachys solani]
unnamed protein product [Clonostachys rosea]
hypothetical protein AK830_g9781 [Neonectria ditissima]
hypothetical protein G7Z17_g4992 [Cylindrodendrum hubeiense]
carboxyphosphonoenolpyruvate phosphonomutase [Fusarium beomiforme]
hypothetical protein IL306_005023 [Fusarium sp. DS 682]
hypothetical protein H9Q69_014132 [Fusarium xylarioides]
hypothetical protein H9Q71_012695 [Fusarium xylarioides]
carboxyphosphonoenolpyruvate phosphonomutase [Fusarium circinatum]
uncharacterized protein FMAN_05567 [Fusarium mangiferae]
hypothetical protein FPRO03_12849 [Fusarium proliferatum]
hypothetical protein FPRO06_06503 [Fusarium proliferatum]
related to carboxyphosphonoenolpyruvate phosphonomutase-like protein [Fusarium proliferatum]
carboxyphosphonoenolpyruvate phosphonomutase [Fusarium subglutinans]
carboxyphosphonoenolpyruvate phosphonomutase [Fusarium sp. NRRL 52700]
carboxyphosphonoenolpyruvate phosphonomutase [Fusarium globosum]
related to carboxyphosphonoenolpyruvate phosphonomutase-like protein [Fusarium fujikuroi IMI 58289]
uncharacterized protein FOYG_01763 [Fusarium oxysporum NRRL 32931]
carboxyphosphonoenolpyruvate phosphonomutase [Fusarium phyllophilum]
carboxyphosphonoenolpyruvate phosphonomutase-like protein [Fusarium fujikuroi]
hypothetical protein CEK27_006957 [Fusarium fujikuroi]
carboxyphosphonoenolpyruvate phosphonomutase [Fusarium acutatum]
hypothetical protein FVEG_04563 [Fusarium verticillioides 7600]
carboxyphosphonoenolpyruvate phosphonomutase [Fusarium pseudoanthophilum]
Uncharacterized protein Forpe1208_v005144 [Fusarium oxysporum f. sp. rapae]
hypothetical protein FLONG3_3879 [Fusarium longipes]
hypothetical protein FPSE_03041 [Fusarium pseudograminearum CS3096]
hypothetical protein FGSG_05939 [Fusarium graminearum PH-1]
hypothetical protein FAUST_7258 [Fusarium austroamericanum]
unnamed protein product [Fusarium graminearum]
hypothetical protein FPSE5266_03041 [Fusarium pseudograminearum]
hypothetical protein HYE67_004169 [Fusarium culmorum]
hypothetical protein CEP53_003090 [Fusarium sp. AF-6]
hypothetical protein CDV31_002853 [Fusarium ambrosium]
hypothetical protein CEP51_001402 [Fusarium floridanum]
hypothetical protein CDV36_000686 [Fusarium kuroshium]
hypothetical protein BHE90_001728 [Fusarium euwallaceae]
hypothetical protein CEP54_005619 [Fusarium sp. AF-8]
uncharacterized protein NECHADRAFT_63735 [Fusarium vanettenii 77-13-4]
carboxyphosphonoenolpyruvate phosphonomutase [Fusarium albosuccineum]
hypothetical protein FDECE_13802 [Fusarium decemcellulare]
uncharacterized protein CkaCkLH20_07630 [Colletotrichum karsti]
hypothetical protein CcaCcLH18_06694 [Colletotrichum camelliae]
hypotheticall protein [Colletotrichum viniferum]
Uncharacterized protein CGCA056_v004381 [Colletotrichum aenigma]
hypotheticall protein [Colletotrichum siamense]
hypotheticall protein [Colletotrichum tropicale]
uncharacterized protein CGMCC3_g11192 [Colletotrichum fructicola]
hypotheticall protein [Colletotrichum fructicola]
hypotheticall protein [Colletotrichum siamense]
hypotheticall protein [Colletotrichum siamense]
Uncharacterized protein CGCS363_v005457 [Colletotrichum siamense]
hypothetical protein GCG54_00000709 [Colletotrichum gloeosporioides]
hypothetical protein CGLO_10225 [Colletotrichum gloeosporioides Cg-14]
hypothetical protein GQ607_003169 [Colletotrichum asianum]
hypothetical protein CORC01_02899 [Colletotrichum orchidophilum]
hypothetical protein CSIM01_03900 [Colletotrichum simmondsii]
hypothetical protein CNYM01_01387 [Colletotrichum nymphaeae SA-01]
uncharacterized protein HER10_EVM0002584 [Colletotrichum scovillei]
hypothetical protein CFIO01_00997 [Colletotrichum fioriniae PJ7]
carboxyphosphonoenolpyruvate phosphonomutase-like protein [Colletotrichum truncatum]
hypothetical protein G7054_g12619 [Neopestalotiopsis clavispora]
hypothetical protein KAF25_004216 [Fusarium avenaceum]
hypothetical protein FAVG1_07851 [Fusarium avenaceum]
hypothetical protein F53441_11505 [Fusarium austroafricanum]
hypothetical protein IL306_010151 [Fusarium sp. DS 682]
hypothetical protein FSARC_12350 [Fusarium sarcochroum]
carboxyphosphonoenolpyruvate phosphonomutase [Fusarium tjaetaba]
hypothetical protein J7337_012712 [Fusarium musae]
hypothetical protein FVEG_08634 [Fusarium verticillioides 7600]
hypothetical protein FVER53590_08634 [Fusarium verticillioides]
hypothetical protein Focb16_v002511 [Fusarium oxysporum f. sp. cubense]
hypothetical protein FGADI_3378 [Fusarium gaditjirri]
carboxyphosphonoenolpyruvate phosphonomutase [Fusarium acutatum]
hypothetical protein FNYG_05484 [Fusarium nygamai]
FTEMP_10_gene_16.21
C6 transcription factor [Fusarium agapanthi]
0.08

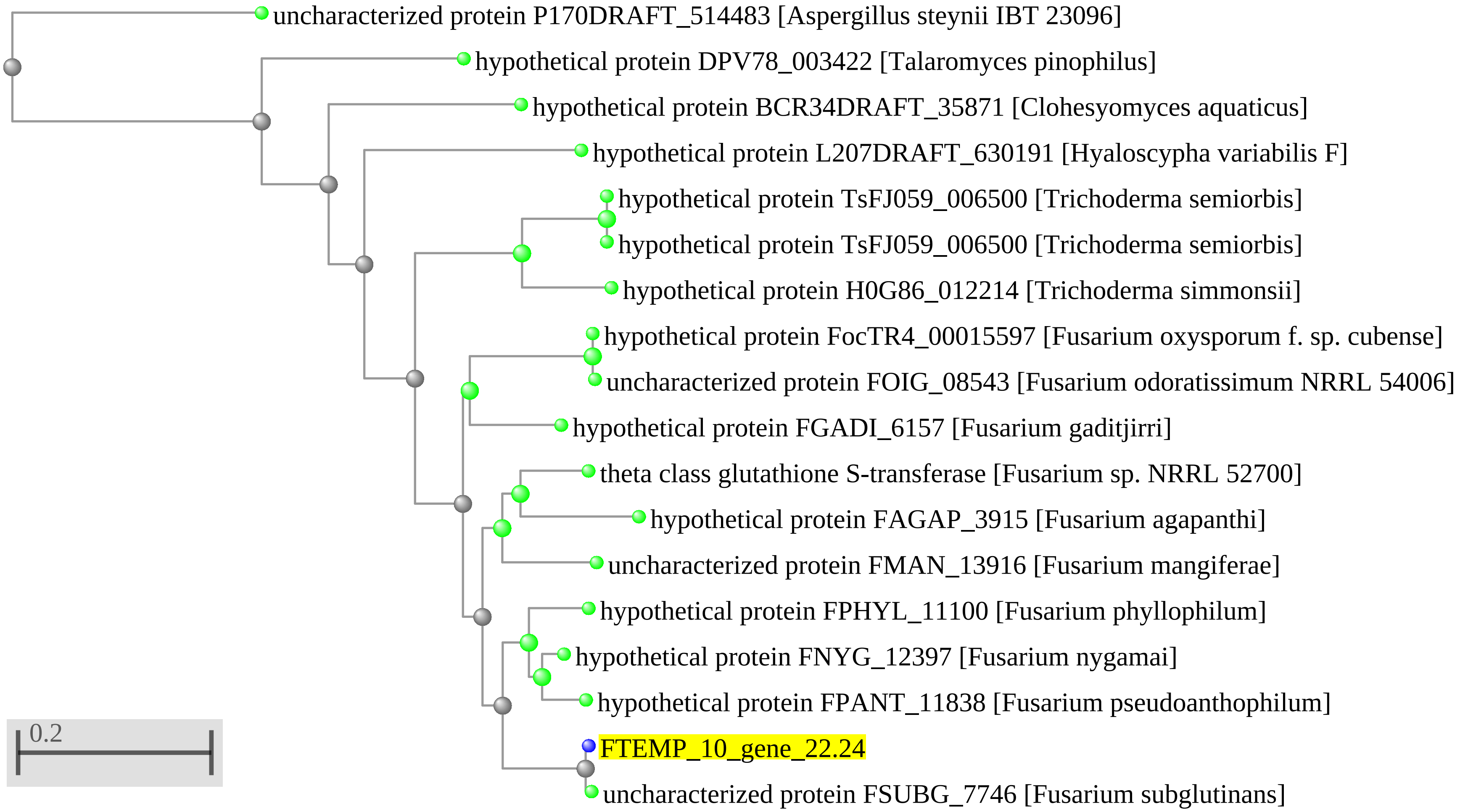

uncharacterized protein P170DRAFT_514483 [Aspergillus steynii IBT 23096]
hypothetical protein DPV78_003422 [Talaromyces pinophilus]
hypothetical protein BCR34DRAFT_35871 [Clohesyomyces aquaticus]
hypothetical protein L207DRAFT_630191 [Hyaloscypha variabilis F]
hypothetical protein TsFJ059_006500 [Trichoderma semiorbis]
hypothetical protein TsFJ059_006500 [Trichoderma semiorbis]
hypothetical protein H0G86_012214 [Trichoderma simmonsii]
hypothetical protein FocTR4_00015597 [Fusarium oxysporum f. sp. cubense]
uncharacterized protein FOIG_08543 [Fusarium odoratissimum NRRL 54006]
hypothetical protein FGADI_6157 [Fusarium gaditjirri]
theta class glutathione S-transferase [Fusarium sp. NRRL 52700]
hypothetical protein FAGAP_3915 [Fusarium agapanthi]
uncharacterized protein FMAN_13916 [Fusarium mangiferae]
hypothetical protein FPHYL_11100 [Fusarium phyllophilum]
hypothetical protein FNYG_12397 [Fusarium nygamai]
hypothetical protein FPANT_11838 [Fusarium pseudoanthophilum]
FTEMP_10_gene_22.24
uncharacterized protein FSUBG_7746 [Fusarium subglutinans]
0.2

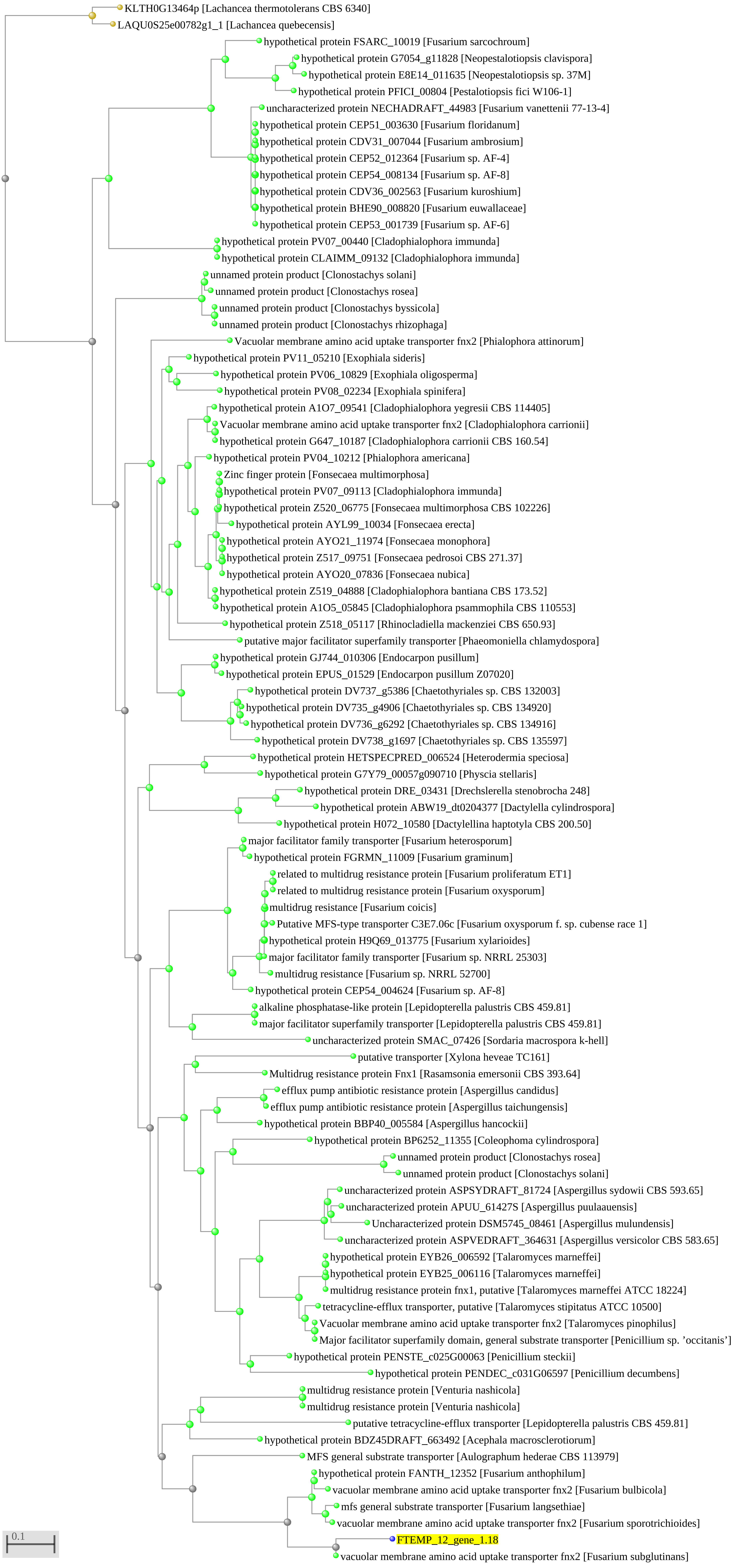

KLTH0G13464p [Lachancea thermotolerans CBS 6340]
LAQU0S25e00782g1_1 [Lachancea quebecensis]
hypothetical protein FSARC_10019 [Fusarium sarcochroum]
hypothetical protein G7054_g11828 [Neopestalotiopsis clavispora]
hypothetical protein E8E14_011635 [Neopestalotiopsis sp. 37M]
hypothetical protein PFICI_00804 [Pestalotiopsis fici W106-1]
uncharacterized protein NECHADRAFT_44983 [Fusarium vanettenii 77-13-4]
hypothetical protein CEP51_003630 [Fusarium floridanum]
hypothetical protein CDV31_007044 [Fusarium ambrosium]
hypothetical protein CEP52_012364 [Fusarium sp. AF-4]
hypothetical protein CEP54_008134 [Fusarium sp. AF-8]
hypothetical protein CDV36_002563 [Fusarium kuroshium]
hypothetical protein BHE90_008820 [Fusarium euwallaceae]
hypothetical protein CEP53_001739 [Fusarium sp. AF-6]
hypothetical protein PV07_00440 [Cladophialophora immunda]
hypothetical protein CLAIMM_09132 [Cladophialophora immunda]
unnamed protein product [Clonostachys solani]
unnamed protein product [Clonostachys rosea]
unnamed protein product [Clonostachys byssicola]
unnamed protein product [Clonostachys rhizophaga]
Vacuolar membrane amino acid uptake transporter fnx2 [Phialophora attinorum]
hypothetical protein PV11_05210 [Exophiala sideris]
hypothetical protein PV06_10829 [Exophiala oligosperma]
hypothetical protein PV08_02234 [Exophiala spinifera]
hypothetical protein A1O7_09541 [Cladophialophora yegresii CBS 114405]
Vacuolar membrane amino acid uptake transporter fnx2 [Cladophialophora carrionii]
hypothetical protein G647_10187 [Cladophialophora carrionii CBS 160.54]
hypothetical protein PV04_10212 [Phialophora americana]
Zinc finger protein [Fonsecaea multimorphosa]
hypothetical protein PV07_09113 [Cladophialophora immunda]
hypothetical protein Z520_06775 [Fonsecaea multimorphosa CBS 102226]
hypothetical protein AYL99_10034 [Fonsecaea erecta]
hypothetical protein AYO21_11974 [Fonsecaea monophora]
hypothetical protein Z517_09751 [Fonsecaea pedrosoi CBS 271.37]
hypothetical protein AYO20_07836 [Fonsecaea nubica]
hypothetical protein Z519_04888 [Cladophialophora bantiana CBS 173.52]
hypothetical protein A1O5_05845 [Cladophialophora psammophila CBS 110553]
hypothetical protein Z518_05117 [Rhinocladiella mackenziei CBS 650.93]
putative major facilitator superfamily transporter [Phaeomoniella chlamydospora]
hypothetical protein GJ744_010306 [Endocarpon pusillum]
hypothetical protein EPUS_01529 [Endocarpon pusillum Z07020]
hypothetical protein DV737_g5386 [Chaetothyriales sp. CBS 132003]
hypothetical protein DV735_g4906 [Chaetothyriales sp. CBS 134920]
hypothetical protein DV736_g6292 [Chaetothyriales sp. CBS 134916]
hypothetical protein DV738_g1697 [Chaetothyriales sp. CBS 135597]
hypothetical protein HETSPECPRED_006524 [Heterodermia speciosa]
hypothetical protein G7Y79_00057g090710 [Physcia stellaris]
hypothetical protein DRE_03431 [Drechslerella stenobrocha 248]
hypothetical protein ABW19_dt0204377 [Dactylella cylindrospora]
hypothetical protein H072_10580 [Dactylellina haptotyla CBS 200.50]
major facilitator family transporter [Fusarium heterosporum]
hypothetical protein FGRMN_11009 [Fusarium graminum]
related to multidrug resistance protein [Fusarium proliferatum ET1]
related to multidrug resistance protein [Fusarium oxysporum]
multidrug resistance [Fusarium coicis]
Putative MFS-type transporter C3E7.06c [Fusarium oxysporum f. sp. cubense race 1]
hypothetical protein H9Q69_013775 [Fusarium xylarioides]
major facilitator family transporter [Fusarium sp. NRRL 25303]
multidrug resistance [Fusarium sp. NRRL 52700]
hypothetical protein CEP54_004624 [Fusarium sp. AF-8]
alkaline phosphatase-like protein [Lepidopterella palustris CBS 459.81]
major facilitator superfamily transporter [Lepidopterella palustris CBS 459.81]
uncharacterized protein SMAC_07426 [Sordaria macrospora k-hell]
putative transporter [Xylona heveae TC161]
Multidrug resistance protein Fnx1 [Rasamsonia emersonii CBS 393.64]
efflux pump antibiotic resistance protein [Aspergillus candidus]
efflux pump antibiotic resistance protein [Aspergillus taichungensis]
hypothetical protein BBP40_005584 [Aspergillus hancockii]
hypothetical protein BP6252_11355 [Coleophoma cylindrospora]
unnamed protein product [Clonostachys rosea]
unnamed protein product [Clonostachys solani]
uncharacterized protein ASPSYDRAFT_81724 [Aspergillus sydowii CBS 593.65]
uncharacterized protein APUU_61427S [Aspergillus puulaauensis]
Uncharacterized protein DSM5745_08461 [Aspergillus mulundensis]
uncharacterized protein ASPVEDRAFT_364631 [Aspergillus versicolor CBS 583.65]
hypothetical protein EYB26_006592 [Talaromyces marneffei]
hypothetical protein EYB25_006116 [Talaromyces marneffei]
multidrug resistance protein fnx1, putative [Talaromyces marneffei ATCC 18224]
tetracycline-efflux transporter, putative [Talaromyces stipitatus ATCC 10500]
Vacuolar membrane amino acid uptake transporter fnx2 [Talaromyces pinophilus]
Major facilitator superfamily domain, general substrate transporter [Penicillium sp. ’occitanis’]
hypothetical protein PENSTE_c025G00063 [Penicillium steckii]
hypothetical protein PENDEC_c031G06597 [Penicillium decumbens]
multidrug resistance protein [Venturia nashicola]
multidrug resistance protein [Venturia nashicola]
putative tetracycline-efflux transporter [Lepidopterella palustris CBS 459.81]
hypothetical protein BDZ45DRAFT_663492 [Acephala macrosclerotiorum]
MFS general substrate transporter [Aulographum hederae CBS 113979]
hypothetical protein FANTH_12352 [Fusarium anthophilum]
vacuolar membrane amino acid uptake transporter fnx2 [Fusarium bulbicola]
mfs general substrate transporter [Fusarium langsethiae]
vacuolar membrane amino acid uptake transporter fnx2 [Fusarium sporotrichioides]
FTEMP_12_gene_1.18
vacuolar membrane amino acid uptake transporter fnx2 [Fusarium subglutinans]
0.1

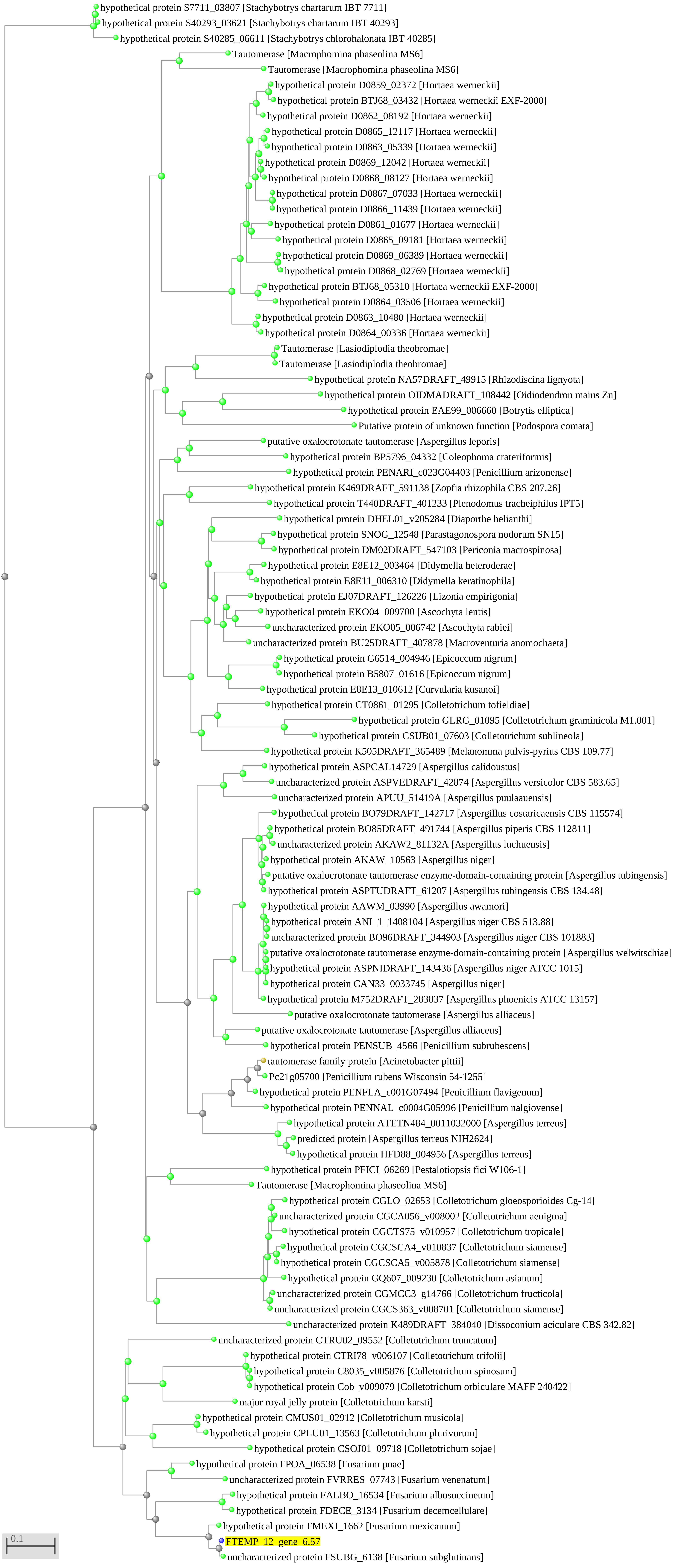
hypothetical protein S7711_03807 [Stachybotrys chartarum IBT 7711]
hypothetical protein S40293_03621 [Stachybotrys chartarum IBT 40293]
hypothetical protein S40285_06611 [Stachybotrys chlorohalonata IBT 40285]
Tautomerase [Macrophomina phaseolina MS6]
Tautomerase [Macrophomina phaseolina MS6]
hypothetical protein D0859_02372 [Hortaea werneckii]
hypothetical protein BTJ68_03432 [Hortaea werneckii EXF-2000]
hypothetical protein D0862_08192 [Hortaea werneckii]
hypothetical protein D0865_12117 [Hortaea werneckii]
hypothetical protein D0863_05339 [Hortaea werneckii]
hypothetical protein D0869_12042 [Hortaea werneckii]
hypothetical protein D0868_08127 [Hortaea werneckii]
hypothetical protein D0867_07033 [Hortaea werneckii]
hypothetical protein D0866_11439 [Hortaea werneckii]
hypothetical protein D0861_01677 [Hortaea werneckii]
hypothetical protein D0865_09181 [Hortaea werneckii]
hypothetical protein D0869_06389 [Hortaea werneckii]
hypothetical protein D0868_02769 [Hortaea werneckii]
hypothetical protein BTJ68_05310 [Hortaea werneckii EXF-2000]
hypothetical protein D0864_03506 [Hortaea werneckii]
hypothetical protein D0863_10480 [Hortaea werneckii]
hypothetical protein D0864_00336 [Hortaea werneckii]
Tautomerase [Lasiodiplodia theobromae]
Tautomerase [Lasiodiplodia theobromae]
hypothetical protein NA57DRAFT_49915 [Rhizodiscina lignyota]
hypothetical protein OIDMADRAFT_108442 [Oidiodendron maius Zn]
hypothetical protein EAE99_006660 [Botrytis elliptica]
Putative protein of unknown function [Podospora comata]
putative oxalocrotonate tautomerase [Aspergillus leporis]
hypothetical protein BP5796_04332 [Coleophoma crateriformis]
hypothetical protein PENARI_c023G04403 [Penicillium arizonense]
hypothetical protein K469DRAFT_591138 [Zopfia rhizophila CBS 207.26]
hypothetical protein T440DRAFT_401233 [Plenodomus tracheiphilus IPT5]
hypothetical protein DHEL01_v205284 [Diaporthe helianthi]
hypothetical protein SNOG_12548 [Parastagonospora nodorum SN15]
hypothetical protein DM02DRAFT_547103 [Periconia macrospinosa]
hypothetical protein E8E12_003464 [Didymella heteroderae]
hypothetical protein E8E11_006310 [Didymella keratinophila]
hypothetical protein EJ07DRAFT_126226 [Lizonia empirigonia]
hypothetical protein EKO04_009700 [Ascochyta lentis]
uncharacterized protein EKO05_006742 [Ascochyta rabiei]
uncharacterized protein BU25DRAFT_407878 [Macroventuria anomochaeta]
hypothetical protein G6514_004946 [Epicoccum nigrum]
hypothetical protein B5807_01616 [Epicoccum nigrum]
hypothetical protein E8E13_010612 [Curvularia kusanoi]
hypothetical protein CT0861_01295 [Colletotrichum tofieldiae]
hypothetical protein GLRG_01095 [Colletotrichum graminicola M1.001]
hypothetical protein CSUB01_07603 [Colletotrichum sublineola]
hypothetical protein K505DRAFT_365489 [Melanomma pulvis-pyrius CBS 109.77]
hypothetical protein ASPCAL14729 [Aspergillus calidoustus]
uncharacterized protein ASPVEDRAFT_42874 [Aspergillus versicolor CBS 583.65]
uncharacterized protein APUU_51419A [Aspergillus puulaauensis]
hypothetical protein BO79DRAFT_142717 [Aspergillus costaricaensis CBS 115574]
hypothetical protein BO85DRAFT_491744 [Aspergillus piperis CBS 112811]
uncharacterized protein AKAW2_81132A [Aspergillus luchuensis]
hypothetical protein AKAW_10563 [Aspergillus niger]
putative oxalocrotonate tautomerase enzyme-domain-containing protein [Aspergillus tubingensis]
hypothetical protein ASPTUDRAFT_61207 [Aspergillus tubingensis CBS 134.48]
hypothetical protein AAWM_03990 [Aspergillus awamori]
hypothetical protein ANI_1_1408104 [Aspergillus niger CBS 513.88]
uncharacterized protein BO96DRAFT_344903 [Aspergillus niger CBS 101883]
putative oxalocrotonate tautomerase enzyme-domain-containing protein [Aspergillus welwitschiae]
hypothetical protein ASPNIDRAFT_143436 [Aspergillus niger ATCC 1015]
hypothetical protein CAN33_0033745 [Aspergillus niger]
hypothetical protein M752DRAFT_283837 [Aspergillus phoenicis ATCC 13157]
putative oxalocrotonate tautomerase [Aspergillus alliaceus]
putative oxalocrotonate tautomerase [Aspergillus alliaceus]
hypothetical protein PENSUB_4566 [Penicillium subrubescens]
tautomerase family protein [Acinetobacter pittii]
Pc21g05700 [Penicillium rubens Wisconsin 54-1255]
hypothetical protein PENFLA_c001G07494 [Penicillium flavigenum]
hypothetical protein PENNAL_c0004G05996 [Penicillium nalgiovense]
hypothetical protein ATETN484_0011032000 [Aspergillus terreus]
predicted protein [Aspergillus terreus NIH2624]
hypothetical protein HFD88_004956 [Aspergillus terreus]
hypothetical protein PFICI_06269 [Pestalotiopsis fici W106-1]
Tautomerase [Macrophomina phaseolina MS6]
hypothetical protein CGLO_02653 [Colletotrichum gloeosporioides Cg-14]
uncharacterized protein CGCA056_v008002 [Colletotrichum aenigma]
hypothetical protein CGCTS75_v010957 [Colletotrichum tropicale]
hypothetical protein CGCSCA4_v010837 [Colletotrichum siamense]
hypothetical protein CGCSCA5_v005878 [Colletotrichum siamense]
hypothetical protein GQ607_009230 [Colletotrichum asianum]
uncharacterized protein CGMCC3_g14766 [Colletotrichum fructicola]
uncharacterized protein CGCS363_v008701 [Colletotrichum siamense]
uncharacterized protein K489DRAFT_384040 [Dissoconium aciculare CBS 342.82]
uncharacterized protein CTRU02_09552 [Colletotrichum truncatum]
hypothetical protein CTRI78_v006107 [Colletotrichum trifolii]
hypothetical protein C8035_v005876 [Colletotrichum spinosum]
hypothetical protein Cob_v009079 [Colletotrichum orbiculare MAFF 240422]
major royal jelly protein [Colletotrichum karsti]
hypothetical protein CMUS01_02912 [Colletotrichum musicola]
hypothetical protein CPLU01_13563 [Colletotrichum plurivorum]
hypothetical protein CSOJ01_09718 [Colletotrichum sojae]
hypothetical protein FPOA_06538 [Fusarium poae]
uncharacterized protein FVRRES_07743 [Fusarium venenatum]
hypothetical protein FALBO_16534 [Fusarium albosuccineum]
hypothetical protein FDECE_3134 [Fusarium decemcellulare]
hypothetical protein FMEXI_1662 [Fusarium mexicanum]
FTEMP_12_gene_6.57
uncharacterized protein FSUBG_6138 [Fusarium subglutinans]
0.1
