# Supplementary material for: Characterization of Host-Specific Genes from Pine- and Grass-Associated Species of the Fusarium fujikuroi Species Complex
Source: Pathogens. 2022 Jul 29;11(8):858. doi: 10.3390/pathogens11080858 (PMC9415769; doi:10.3390/pathogens11080858)
Supplement: Supplementary file 1 [file pathogens-11-00858-s001.zip › Supplemental Figures/Figure S3.pdf]

Figure S3. Host-range-associated genes with ancestral origins that emerged within the FFSC and FOSC. Host-range-associated genes investigated are highlighted in yellow; FCIR = *Fusarium circinatum* and FTEMP = *Fusarium temperatum.*

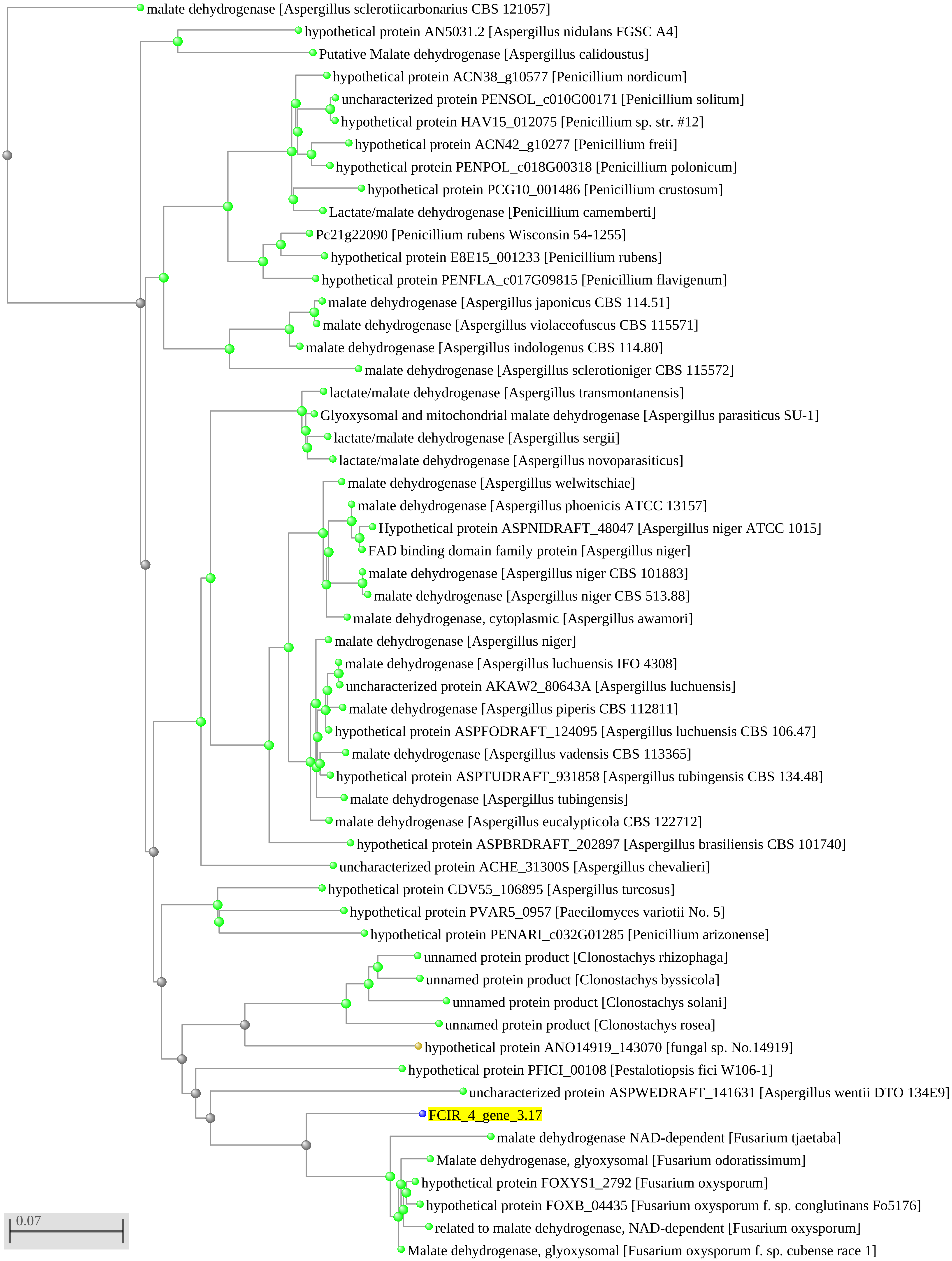

malate dehydrogenase [Aspergillus sclerotiicarbonarius CBS 121057]
hypothetical protein AN5031.2 [Aspergillus nidulans FGSC A4]
Putative Malate dehydrogenase [Aspergillus calidoustus]
hypothetical protein ACN38_g10577 [Penicillium nordicum]
uncharacterized protein PENSOL_c010G00171 [Penicillium solitum]
hypothetical protein HAV15_012075 [Penicillium sp. str. #12]
hypothetical protein ACN42_g10277 [Penicillium freii]
hypothetical protein PENPOL_c018G00318 [Penicillium polonicum]
hypothetical protein PCG10_001486 [Penicillium crustosum]
Lactate/malate dehydrogenase [Penicillium camemberti]
Pc21g22090 [Penicillium rubens Wisconsin 54-1255]
hypothetical protein E8E15_001233 [Penicillium rubens]
hypothetical protein PENFLA_c017G09815 [Penicillium flavigenum]
malate dehydrogenase [Aspergillus japonicus CBS 114.51]
malate dehydrogenase [Aspergillus violaceofuscus CBS 115571]
malate dehydrogenase [Aspergillus indologenus CBS 114.80]
malate dehydrogenase [Aspergillus sclerotioniger CBS 115572]
lactate/malate dehydrogenase [Aspergillus transmontanensis]
Glyoxysomal and mitochondrial malate dehydrogenase [Aspergillus parasiticus SU-1]
lactate/malate dehydrogenase [Aspergillus sergii]
lactate/malate dehydrogenase [Aspergillus novoparasiticus]
malate dehydrogenase [Aspergillus welwitschiae]
malate dehydrogenase [Aspergillus phoenicis ATCC 13157]
Hypothetical protein ASPNIDRAFT_48047 [Aspergillus niger ATCC 1015]
FAD binding domain family protein [Aspergillus niger]
malate dehydrogenase [Aspergillus niger CBS 101883]
malate dehydrogenase [Aspergillus niger CBS 513.88]
malate dehydrogenase, cytoplasmic [Aspergillus awamori]
malate dehydrogenase [Aspergillus niger]
malate dehydrogenase [Aspergillus luchuensis IFO 4308]
uncharacterized protein AKAW2_80643A [Aspergillus luchuensis]
malate dehydrogenase [Aspergillus piperis CBS 112811]
hypothetical protein ASPFODRAFT_124095 [Aspergillus luchuensis CBS 106.47]
malate dehydrogenase [Aspergillus vadensis CBS 113365]
hypothetical protein ASPTUDRAFT_931858 [Aspergillus tubingensis CBS 134.48]
malate dehydrogenase [Aspergillus tubingensis]
malate dehydrogenase [Aspergillus eucalypticola CBS 122712]
hypothetical protein ASPBRDRAFT_202897 [Aspergillus brasiliensis CBS 101740]
uncharacterized protein ACHE_31300S [Aspergillus chevalieri]
hypothetical protein CDV55_106895 [Aspergillus turcosus]
hypothetical protein PVAR5_0957 [Paecilomyces variotii No. 5]
hypothetical protein PENARI_c032G01285 [Penicillium arizonense]
unnamed protein product [Clonostachys rhizophaga]
unnamed protein product [Clonostachys byssicola]
unnamed protein product [Clonostachys solani]
unnamed protein product [Clonostachys rosea]
hypothetical protein ANO14919_143070 [fungal sp. No.14919]
hypothetical protein PFICI_00108 [Pestalotiopsis fici W106-1]
uncharacterized protein ASPWEDRAFT_141631 [Aspergillus wentii DTO 134E9]
FCIR_4_gene_3.17
malate dehydrogenase NAD-dependent [Fusarium tjaetaba]
Malate dehydrogenase, glyoxysomal [Fusarium odoratissimum]
hypothetical protein FOXYS1_2792 [Fusarium oxysporum]
hypothetical protein FOXB_04435 [Fusarium oxysporum f. sp. conglutinans Fo5176]
related to malate dehydrogenase, NAD-dependent [Fusarium oxysporum]
Malate dehydrogenase, glyoxysomal [Fusarium oxysporum f. sp. cubense race 1]
0.07

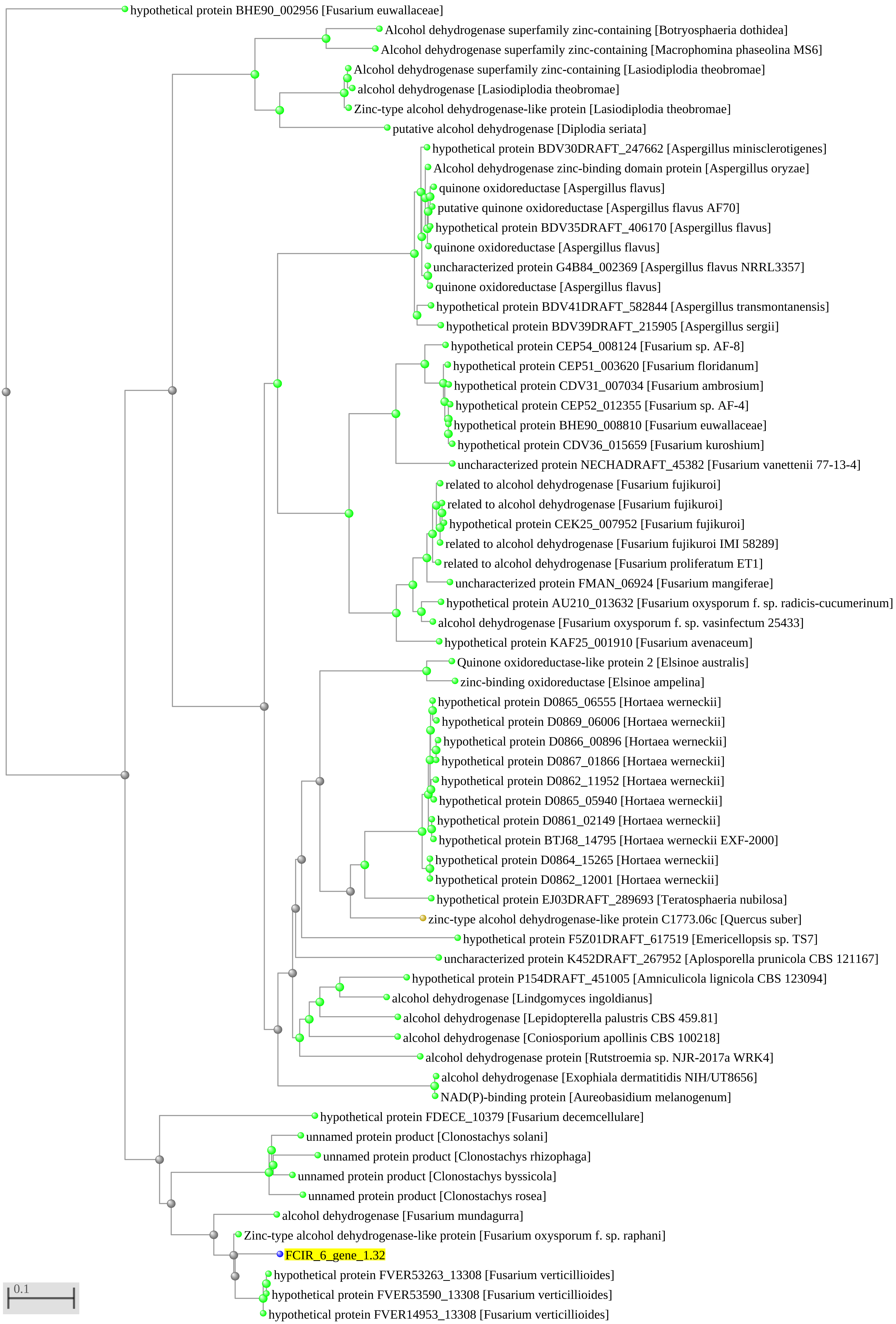
hypothetical protein BHE90_002956 [Fusarium euwallaceae]
Alcohol dehydrogenase superfamily zinc-containing [Botryosphaeria dothidea]
Alcohol dehydrogenase superfamily zinc-containing [Macrophomina phaseolina MS6]
Alcohol dehydrogenase superfamily zinc-containing [Lasiodiplodia theobromae]
alcohol dehydrogenase [Lasiodiplodia theobromae]
Zinc-type alcohol dehydrogenase-like protein [Lasiodiplodia theobromae]
putative alcohol dehydrogenase [Diplodia seriata]
hypothetical protein BDV30DRAFT_247662 [Aspergillus minisclerotigenes]
Alcohol dehydrogenase zinc-binding domain protein [Aspergillus oryzae]
quinone oxidoreductase [Aspergillus flavus]
putative quinone oxidoreductase [Aspergillus flavus AF70]
hypothetical protein BDV35DRAFT_406170 [Aspergillus flavus]
quinone oxidoreductase [Aspergillus flavus]
uncharacterized protein G4B84_002369 [Aspergillus flavus NRRL3357]
quinone oxidoreductase [Aspergillus flavus]
hypothetical protein BDV41DRAFT_582844 [Aspergillus transmontanensis]
hypothetical protein BDV39DRAFT_215905 [Aspergillus sergii]
hypothetical protein CEP54_008124 [Fusarium sp. AF-8]
hypothetical protein CEP51_003620 [Fusarium floridanum]
hypothetical protein CDV31_007034 [Fusarium ambrosium]
hypothetical protein CEP52_012355 [Fusarium sp. AF-4]
hypothetical protein BHE90_008810 [Fusarium euwallaceae]
hypothetical protein CDV36_015659 [Fusarium kuroshium]
uncharacterized protein NECHADRAFT_45382 [Fusarium vanettenii 77-13-4]
related to alcohol dehydrogenase [Fusarium fujikuroi]
related to alcohol dehydrogenase [Fusarium fujikuroi]
hypothetical protein CEK25_007952 [Fusarium fujikuroi]
related to alcohol dehydrogenase [Fusarium fujikuroi IMI 58289]
related to alcohol dehydrogenase [Fusarium proliferatum ET1]
uncharacterized protein FMAN_06924 [Fusarium mangiferae]
hypothetical protein AU210_013632 [Fusarium oxysporum f. sp. radicis-cucumerinum]
alcohol dehydrogenase [Fusarium oxysporum f. sp. vasinfectum 25433]
hypothetical protein KAF25_001910 [Fusarium avenaceum]
Quinone oxidoreductase-like protein 2 [Elsinoe australis]
zinc-binding oxidoreductase [Elsinoe ampelina]
hypothetical protein D0865_06555 [Hortaea werneckii]
hypothetical protein D0869_06006 [Hortaea werneckii]
hypothetical protein D0866_00896 [Hortaea werneckii]
hypothetical protein D0867_01866 [Hortaea werneckii]
hypothetical protein D0862_11952 [Hortaea werneckii]
hypothetical protein D0865_05940 [Hortaea werneckii]
hypothetical protein D0861_02149 [Hortaea werneckii]
hypothetical protein BTJ68_14795 [Hortaea werneckii EXF-2000]
hypothetical protein D0864_15265 [Hortaea werneckii]
hypothetical protein D0862_12001 [Hortaea werneckii]
hypothetical protein EJ03DRAFT_289693 [Teratosphaeria nubilosa]
zinc-type alcohol dehydrogenase-like protein C1773.06c [Quercus suber]
hypothetical protein F5Z01DRAFT_617519 [Emericellopsis sp. TS7]
uncharacterized protein K452DRAFT_267952 [Aplosporella prunicola CBS 121167]
hypothetical protein P154DRAFT_451005 [Amniculicola lignicola CBS 123094]
alcohol dehydrogenase [Lindgomyces ingoldianus]
alcohol dehydrogenase [Lepidopterella palustris CBS 459.81]
alcohol dehydrogenase [Coniosporium apollinis CBS 100218]
alcohol dehydrogenase protein [Rutstroemia sp. NJR-2017a WRK4]
alcohol dehydrogenase [Exophiala dermatitidis NIH/UT8656]
NAD(P)-binding protein [Aureobasidium melanogenum]
hypothetical protein FDECE_10379 [Fusarium decemcellulare]
unnamed protein product [Clonostachys solani]
unnamed protein product [Clonostachys rhizophaga]
unnamed protein product [Clonostachys byssicola]
unnamed protein product [Clonostachys rosea]
alcohol dehydrogenase [Fusarium mundagurra]
Zinc-type alcohol dehydrogenase-like protein [Fusarium oxysporum f. sp. raphani]
FCIR_6_gene_1.32
hypothetical protein FVER53263_13308 [Fusarium verticillioides]
hypothetical protein FVER53590_13308 [Fusarium verticillioides]
hypothetical protein FVER14953_13308 [Fusarium verticillioides]
0.1

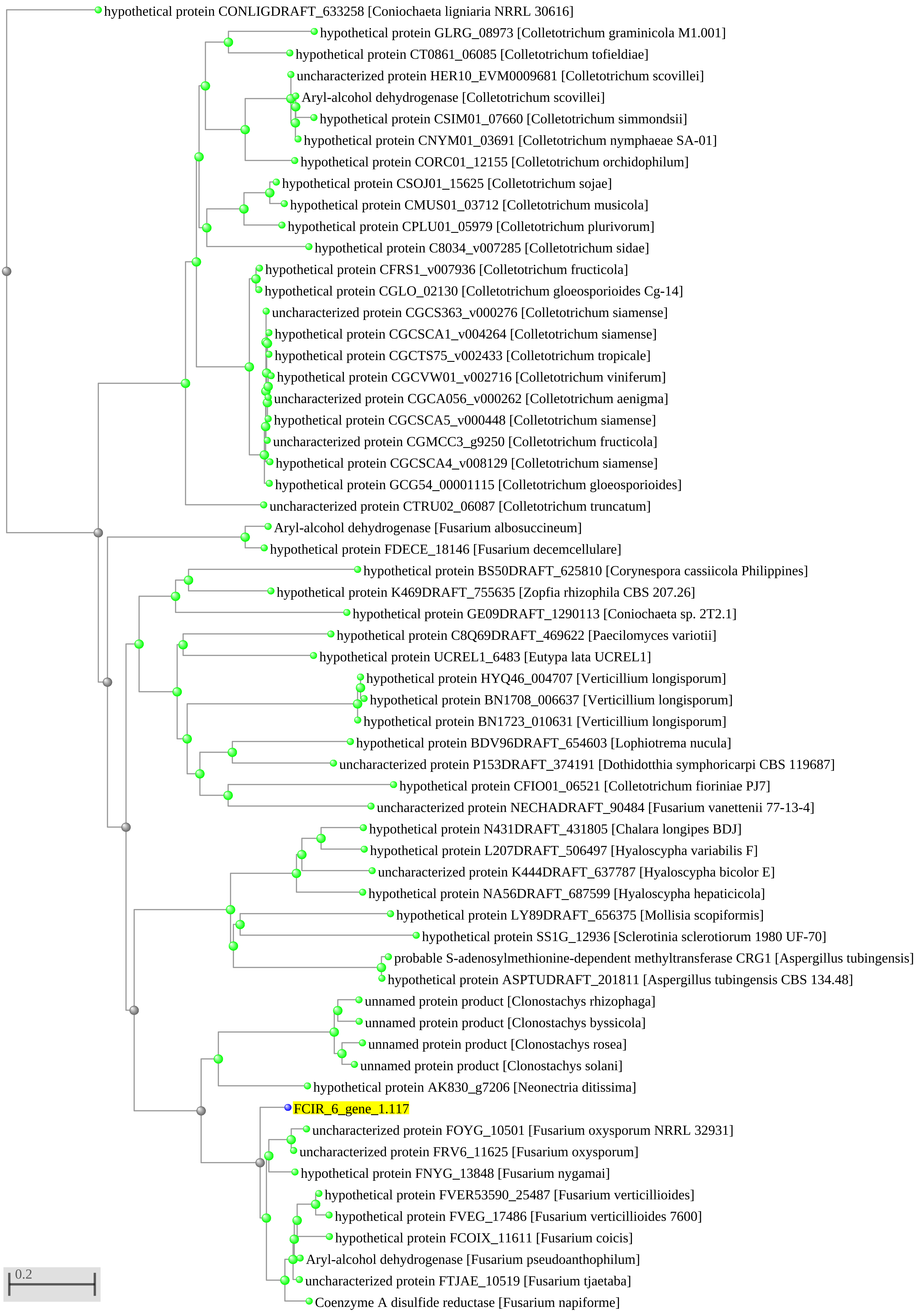
hypothetical protein CONLIGDRAFT_633258 [Coniochaeta ligniaria NRRL 30616]
hypothetical protein GLRG_08973 [Colletotrichum graminicola M1.001]
hypothetical protein CT0861_06085 [Colletotrichum tofieldiae]
uncharacterized protein HER10_EVM0009681 [Colletotrichum scovillei]
Aryl-alcohol dehydrogenase [Colletotrichum scovillei]
hypothetical protein CSIM01_07660 [Colletotrichum simmondsii]
hypothetical protein CNYM01_03691 [Colletotrichum nymphaeae SA-01]
hypothetical protein CORC01_12155 [Colletotrichum orchidophilum]
hypothetical protein CSOJ01_15625 [Colletotrichum sojae]
hypothetical protein CMUS01_03712 [Colletotrichum musicola]
hypothetical protein CPLU01_05979 [Colletotrichum plurivorum]
hypothetical protein C8034_v007285 [Colletotrichum sidae]
hypothetical protein CFRS1_v007936 [Colletotrichum fructicola]
hypothetical protein CGLO_02130 [Colletotrichum gloeosporioides Cg-14]
uncharacterized protein CGCS363_v000276 [Colletotrichum siamense]
hypothetical protein CGCSCA1_v004264 [Colletotrichum siamense]
hypothetical protein CGCTS75_v002433 [Colletotrichum tropicale]
hypothetical protein CGCVW01_v002716 [Colletotrichum viniferum]
uncharacterized protein CGCA056_v000262 [Colletotrichum aenigma]
hypothetical protein CGCSCA5_v000448 [Colletotrichum siamense]
uncharacterized protein CGMCC3_g9250 [Colletotrichum fructicola]
hypothetical protein CGCSCA4_v008129 [Colletotrichum siamense]
hypothetical protein GCG54_00001115 [Colletotrichum gloeosporioides]
uncharacterized protein CTRU02_06087 [Colletotrichum truncatum]
Aryl-alcohol dehydrogenase [Fusarium albosuccineum]
hypothetical protein FDECE_18146 [Fusarium decemcellulare]
hypothetical protein BS50DRAFT_625810 [Corynespora cassiicola Philippines]
hypothetical protein K469DRAFT_755635 [Zopfia rhizophila CBS 207.26]
hypothetical protein GE09DRAFT_1290113 [Coniochaeta sp. 2T2.1]
hypothetical protein C8Q69DRAFT_469622 [Paecilomyces variotii]
hypothetical protein UCREL1_6483 [Eutypa lata UCREL1]
hypothetical protein HYQ46_004707 [Verticillium longisporum]
hypothetical protein BN1708_006637 [Verticillium longisporum]
hypothetical protein BN1723_010631 [Verticillium longisporum]
hypothetical protein BDV96DRAFT_654603 [Lophiotrema nucula]
uncharacterized protein P153DRAFT_374191 [Dothidotthia symphoricarpi CBS 119687]
hypothetical protein CFIO01_06521 [Colletotrichum fioriniae PJ7]
uncharacterized protein NECHADRAFT_90484 [Fusarium vanettenii 77-13-4]
hypothetical protein N431DRAFT_431805 [Chalara longipes BDJ]
hypothetical protein L207DRAFT_506497 [Hyaloscypha variabilis F]
uncharacterized protein K444DRAFT_637787 [Hyaloscypha bicolor E]
hypothetical protein NA56DRAFT_687599 [Hyaloscypha hepaticicola]
hypothetical protein LY89DRAFT_656375 [Mollisia scopiformis]
hypothetical protein SS1G_12936 [Sclerotinia sclerotiorum 1980 UF-70]
probable S-adenosylmethionine-dependent methyltransferase CRG1 [Aspergillus tubingensis]
hypothetical protein ASPTUDRAFT_201811 [Aspergillus tubingensis CBS 134.48]
unnamed protein product [Clonostachys rhizophaga]
unnamed protein product [Clonostachys byssicola]
unnamed protein product [Clonostachys rosea]
unnamed protein product [Clonostachys solani]
hypothetical protein AK830_g7206 [Neonectria ditissima]
FCIR_6_gene_1.117
uncharacterized protein FOYG_10501 [Fusarium oxysporum NRRL 32931]
uncharacterized protein FRV6_11625 [Fusarium oxysporum]
hypothetical protein FNYG_13848 [Fusarium nygamai]
hypothetical protein FVER53590_25487 [Fusarium verticillioides]
hypothetical protein FVEG_17486 [Fusarium verticillioides 7600]
hypothetical protein FCOIX_11611 [Fusarium coicis]
Aryl-alcohol dehydrogenase [Fusarium pseudoanthophilum]
uncharacterized protein FTJAE_10519 [Fusarium tjaetaba]
Coenzyme A disulfide reductase [Fusarium napiforme]
0.2

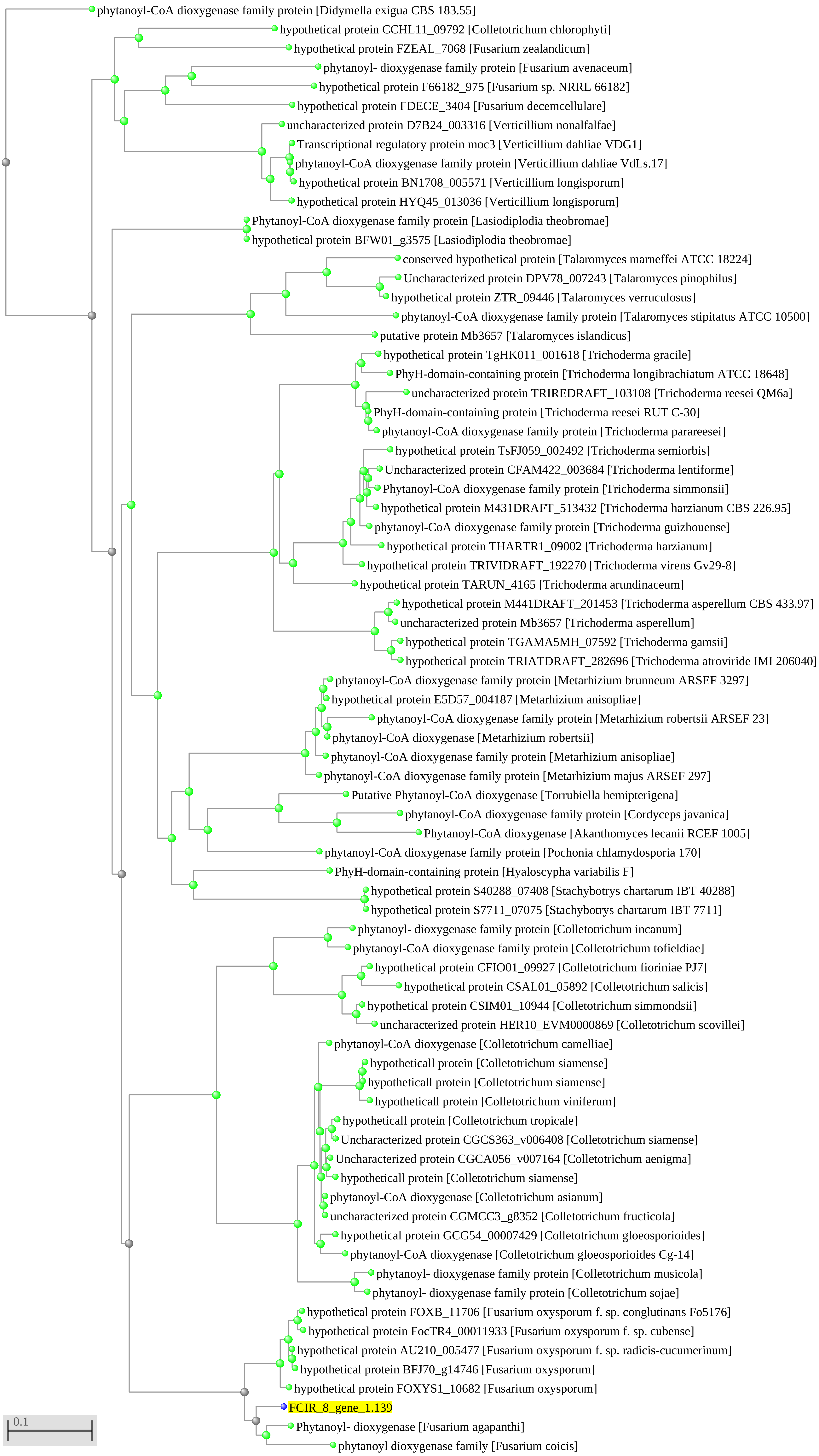
phytanoyl-CoA dioxygenase family protein [Didymella exigua CBS 183.55]
hypothetical protein CCHL11_09792 [Colletotrichum chlorophyti]
hypothetical protein FZEAL_7068 [Fusarium zealandicum]
phytanoyl- dioxygenase family protein [Fusarium avenaceum]
hypothetical protein F66182_975 [Fusarium sp. NRRL 66182]
hypothetical protein FDECE_3404 [Fusarium decemcellulare]
uncharacterized protein D7B24_003316 [Verticillium nonalfalfae]
Transcriptional regulatory protein moc3 [Verticillium dahliae VDG1]
phytanoyl-CoA dioxygenase family protein [Verticillium dahliae VdLs.17]
hypothetical protein BN1708_005571 [Verticillium longisporum]
hypothetical protein HYQ45_013036 [Verticillium longisporum]
Phytanoyl-CoA dioxygenase family protein [Lasiodiplodia theobromae]
hypothetical protein BFW01_g3575 [Lasiodiplodia theobromae]
conserved hypothetical protein [Talaromyces marneffei ATCC 18224]
Uncharacterized protein DPV78_007243 [Talaromyces pinophilus]
hypothetical protein ZTR_09446 [Talaromyces verruculosus]
phytanoyl-CoA dioxygenase family protein [Talaromyces stipitatus ATCC 10500]
putative protein Mb3657 [Talaromyces islandicus]
hypothetical protein TgHK011_001618 [Trichoderma gracile]
PhyH-domain-containing protein [Trichoderma longibrachiatum ATCC 18648]
uncharacterized protein TRIREDRAFT_103108 [Trichoderma reesei QM6a]
PhyH-domain-containing protein [Trichoderma reesei RUT C-30]
phytanoyl-CoA dioxygenase family protein [Trichoderma parareesei]
hypothetical protein TsFJ059_002492 [Trichoderma semiorbis]
Uncharacterized protein CFAM422_003684 [Trichoderma lentiforme]
Phytanoyl-CoA dioxygenase family protein [Trichoderma simmonsii]
hypothetical protein M431DRAFT_513432 [Trichoderma harzianum CBS 226.95]
phytanoyl-CoA dioxygenase family protein [Trichoderma guizhouense]
hypothetical protein THARTR1_09002 [Trichoderma harzianum]
hypothetical protein TRIVIDRAFT_192270 [Trichoderma virens Gv29-8]
hypothetical protein TARUN_4165 [Trichoderma arundinaceum]
hypothetical protein M441DRAFT_201453 [Trichoderma asperellum CBS 433.97]
uncharacterized protein Mb3657 [Trichoderma asperellum]
hypothetical protein TGAMA5MH_07592 [Trichoderma gamsii]
hypothetical protein TRIATDRAFT_282696 [Trichoderma atroviride IMI 206040]
phytanoyl-CoA dioxygenase family protein [Metarhizium brunneum ARSEF 3297]
hypothetical protein E5D57_004187 [Metarhizium anisopliae]
phytanoyl-CoA dioxygenase family protein [Metarhizium robertsii ARSEF 23]
phytanoyl-CoA dioxygenase [Metarhizium robertsii]
phytanoyl-CoA dioxygenase family protein [Metarhizium anisopliae]
phytanoyl-CoA dioxygenase family protein [Metarhizium majus ARSEF 297]
Putative Phytanoyl-CoA dioxygenase [Torrubiella hemipterigena]
phytanoyl-CoA dioxygenase family protein [Cordyceps javanica]
Phytanoyl-CoA dioxygenase [Akanthomyces lecanii RCEF 1005]
phytanoyl-CoA dioxygenase family protein [Pochonia chlamydosporia 170]
PhyH-domain-containing protein [Hyaloscypha variabilis F]
hypothetical protein S40288_07408 [Stachybotrys chartarum IBT 40288]
hypothetical protein S7711_07075 [Stachybotrys chartarum IBT 7711]
phytanoyl- dioxygenase family protein [Colletotrichum incanum]
phytanoyl-CoA dioxygenase family protein [Colletotrichum tofieldiae]
hypothetical protein CFIO01_09927 [Colletotrichum fioriniae PJ7]
hypothetical protein CSAL01_05892 [Colletotrichum salicis]
hypothetical protein CSIM01_10944 [Colletotrichum simmondsii]
uncharacterized protein HER10_EVM0000869 [Colletotrichum scovillei]
phytanoyl-CoA dioxygenase [Colletotrichum camelliae]
hypotheticall protein [Colletotrichum siamense]
hypotheticall protein [Colletotrichum siamense]
hypotheticall protein [Colletotrichum viniferum]
hypotheticall protein [Colletotrichum tropicale]
Uncharacterized protein CGCS363_v006408 [Colletotrichum siamense]
Uncharacterized protein CGCA056_v007164 [Colletotrichum aenigma]
hypotheticall protein [Colletotrichum siamense]
phytanoyl-CoA dioxygenase [Colletotrichum asianum]
uncharacterized protein CGMCC3_g8352 [Colletotrichum fructicola]
hypothetical protein GCG54_00007429 [Colletotrichum gloeosporioides]
phytanoyl-CoA dioxygenase [Colletotrichum gloeosporioides Cg-14]
phytanoyl- dioxygenase family protein [Colletotrichum musicola]
phytanoyl- dioxygenase family protein [Colletotrichum sojae]
hypothetical protein FOXB_11706 [Fusarium oxysporum f. sp. conglutinans Fo5176]
hypothetical protein FocTR4_00011933 [Fusarium oxysporum f. sp. cubense]
hypothetical protein AU210_005477 [Fusarium oxysporum f. sp. radicis-cucumerinum]
hypothetical protein BFJ70_g14746 [Fusarium oxysporum]
hypothetical protein FOXYS1_10682 [Fusarium oxysporum]
FCIR_8_gene_1.139
Phytanoyl- dioxygenase [Fusarium agapanthi]
phytanoyl dioxygenase family [Fusarium coicis]
0.1

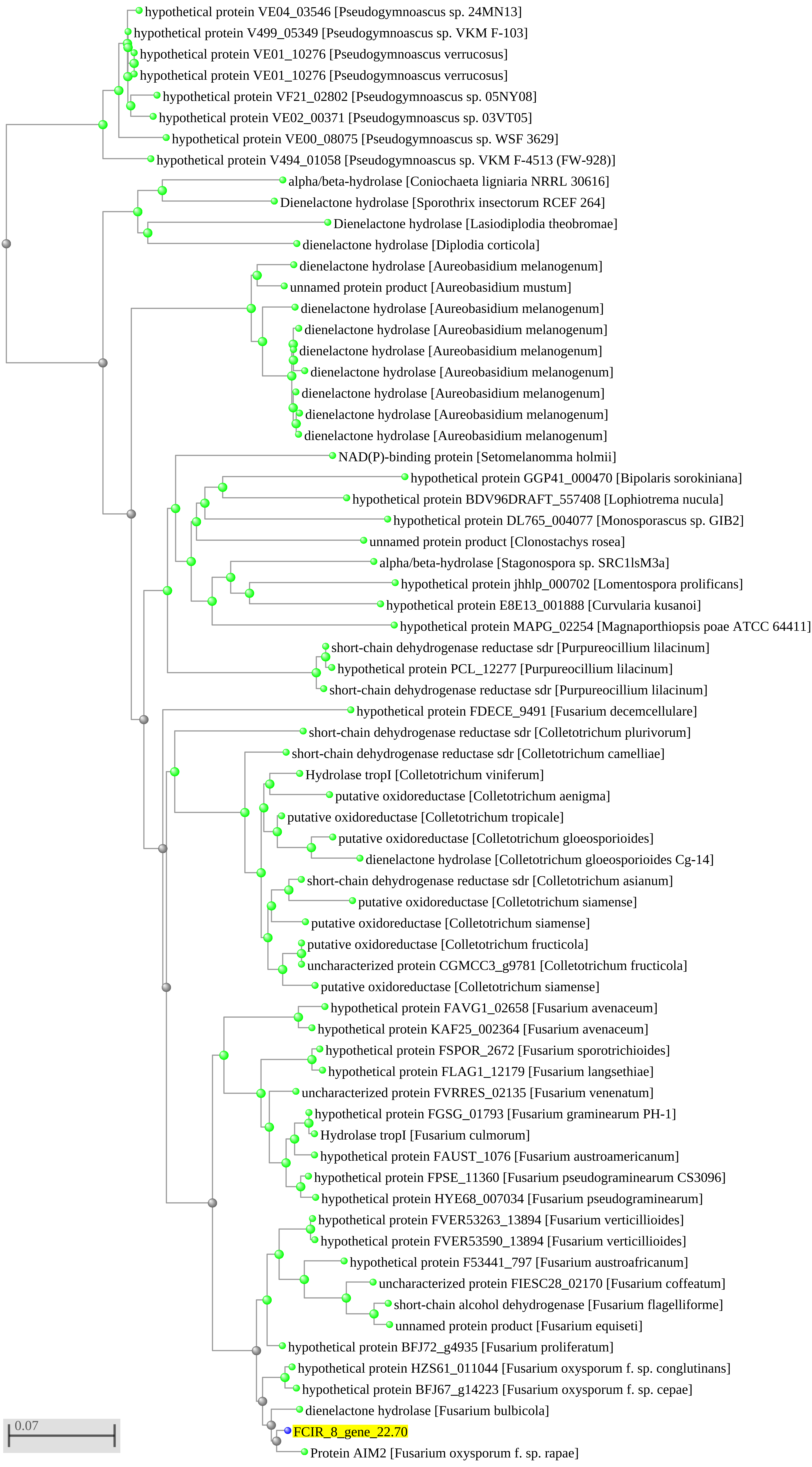
hypothetical protein VE04_03546 [Pseudogymnoascus sp. 24MN13]
hypothetical protein V499_05349 [Pseudogymnoascus sp. VKM F-103]
hypothetical protein VE01_10276 [Pseudogymnoascus verrucosus]
hypothetical protein VE01_10276 [Pseudogymnoascus verrucosus]
hypothetical protein VF21_02802 [Pseudogymnoascus sp. 05NY08]
hypothetical protein VE02_00371 [Pseudogymnoascus sp. 03VT05]
hypothetical protein VE00_08075 [Pseudogymnoascus sp. WSF 3629]
hypothetical protein V494_01058 [Pseudogymnoascus sp. VKM F-4513 (FW-928)]
alpha/beta-hydrolase [Coniochaeta ligniaria NRRL 30616]
Dienelactone hydrolase [Sporothrix insectorum RCEF 264]
Dienelactone hydrolase [Lasiodiplodia theobromae]
dienelactone hydrolase [Diplodia corticola]
dienelactone hydrolase [Aureobasidium melanogenum]
unnamed protein product [Aureobasidium mustum]
dienelactone hydrolase [Aureobasidium melanogenum]
dienelactone hydrolase [Aureobasidium melanogenum]
dienelactone hydrolase [Aureobasidium melanogenum]
dienelactone hydrolase [Aureobasidium melanogenum]
dienelactone hydrolase [Aureobasidium melanogenum]
dienelactone hydrolase [Aureobasidium melanogenum]
dienelactone hydrolase [Aureobasidium melanogenum]
NAD(P)-binding protein [Setomelanomma holmii]
hypothetical protein GGP41_000470 [Bipolaris sorokiniana]
hypothetical protein BDV96DRAFT_557408 [Lophiotrema nucula]
hypothetical protein DL765_004077 [Monosporascus sp. GIB2]
unnamed protein product [Clonostachys rosea]
alpha/beta-hydrolase [Stagonospora sp. SRC1lsM3a]
hypothetical protein jhhlp_000702 [Lomentospora prolificans]
hypothetical protein E8E13_001888 [Curvularia kusanoi]
hypothetical protein MAPG_02254 [Magnaporthiopsis poae ATCC 64411]
short-chain dehydrogenase reductase sdr [Purpureocillium lilacinum]
hypothetical protein PCL_12277 [Purpureocillium lilacinum]
short-chain dehydrogenase reductase sdr [Purpureocillium lilacinum]
hypothetical protein FDECE_9491 [Fusarium decemcellulare]
short-chain dehydrogenase reductase sdr [Colletotrichum plurivorum]
short-chain dehydrogenase reductase sdr [Colletotrichum camelliae]
Hydrolase tropI [Colletotrichum viniferum]
putative oxidoreductase [Colletotrichum aenigma]
putative oxidoreductase [Colletotrichum tropicale]
putative oxidoreductase [Colletotrichum gloeosporioides]
dienelactone hydrolase [Colletotrichum gloeosporioides Cg-14]
short-chain dehydrogenase reductase sdr [Colletotrichum asianum]
putative oxidoreductase [Colletotrichum siamense]
putative oxidoreductase [Colletotrichum siamense]
putative oxidoreductase [Colletotrichum fructicola]
uncharacterized protein CGMCC3_g9781 [Colletotrichum fructicola]
putative oxidoreductase [Colletotrichum siamense]
hypothetical protein FAVG1_02658 [Fusarium avenaceum]
hypothetical protein KAF25_002364 [Fusarium avenaceum]
hypothetical protein FSPOR_2672 [Fusarium sporotrichioides]
hypothetical protein FLAG1_12179 [Fusarium langsethiae]
uncharacterized protein FVRRES_02135 [Fusarium venenatum]
hypothetical protein FGSG_01793 [Fusarium graminearum PH-1]
Hydrolase tropI [Fusarium culmorum]
hypothetical protein FAUST_1076 [Fusarium austroamericanum]
hypothetical protein FPSE_11360 [Fusarium pseudograminearum CS3096]
hypothetical protein HYE68_007034 [Fusarium pseudograminearum]
hypothetical protein FVER53263_13894 [Fusarium verticillioides]
hypothetical protein FVER53590_13894 [Fusarium verticillioides]
hypothetical protein F53441_797 [Fusarium austroafricanum]
uncharacterized protein FIESC28_02170 [Fusarium coffeatum]
short-chain alcohol dehydrogenase [Fusarium flagelliforme]
unnamed protein product [Fusarium equiseti]
hypothetical protein BFJ72_g4935 [Fusarium proliferatum]
hypothetical protein HZS61_011044 [Fusarium oxysporum f. sp. conglutinans]
hypothetical protein BFJ67_g14223 [Fusarium oxysporum f. sp. cepae]
dienelactone hydrolase [Fusarium bulbicola]
FCIR_8_gene_22.70
Protein AIM2 [Fusarium oxysporum f. sp. rapae]
0.07

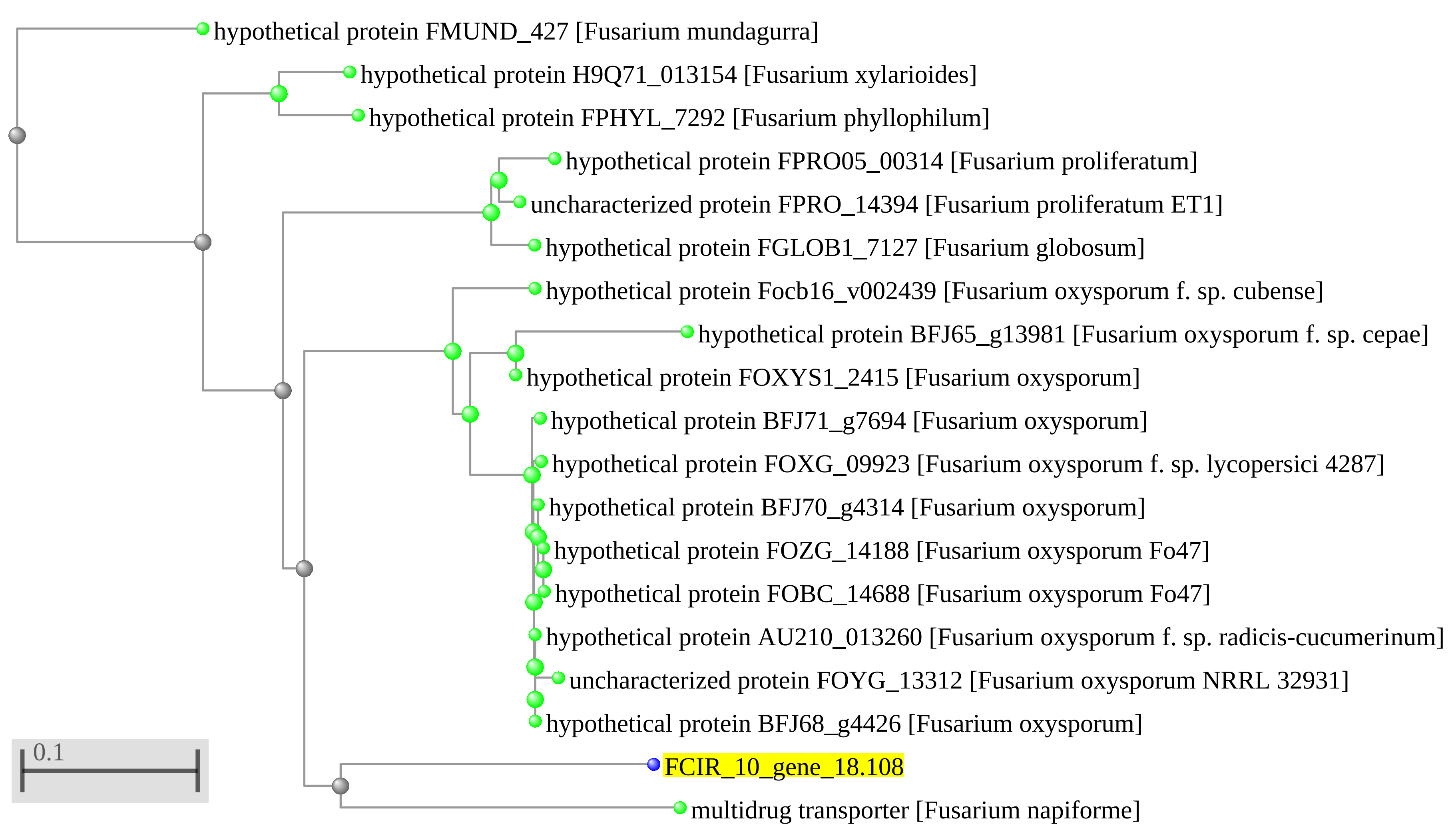
hypothetical protein FMUND_427 [Fusarium mundagurra]
hypothetical protein H9Q71_013154 [Fusarium xylarioides]
hypothetical protein FPHYL_7292 [Fusarium phyllophilum]
hypothetical protein FPRO05_00314 [Fusarium proliferatum]
uncharacterized protein FPRO_14394 [Fusarium proliferatum ET1]
hypothetical protein FGLOB1_7127 [Fusarium globosum]
hypothetical protein Focb16_v002439 [Fusarium oxysporum f. sp. cubense]
hypothetical protein BFJ65_g13981 [Fusarium oxysporum f. sp. cepae]
hypothetical protein FOXYS1_2415 [Fusarium oxysporum]
hypothetical protein BFJ71_g7694 [Fusarium oxysporum]
hypothetical protein FOXG_09923 [Fusarium oxysporum f. sp. lycopersici 4287]
hypothetical protein BFJ70_g4314 [Fusarium oxysporum]
hypothetical protein FOZG_14188 [Fusarium oxysporum Fo47]
hypothetical protein FOBC_14688 [Fusarium oxysporum Fo47]
hypothetical protein AU210_013260 [Fusarium oxysporum f. sp. radicis-cucumerinum]
uncharacterized protein FOYG_13312 [Fusarium oxysporum NRRL 32931]
hypothetical protein BFJ68_g4426 [Fusarium oxysporum]
FCIR_10_gene_18.108
multidrug transporter [Fusarium napiforme]
0.1

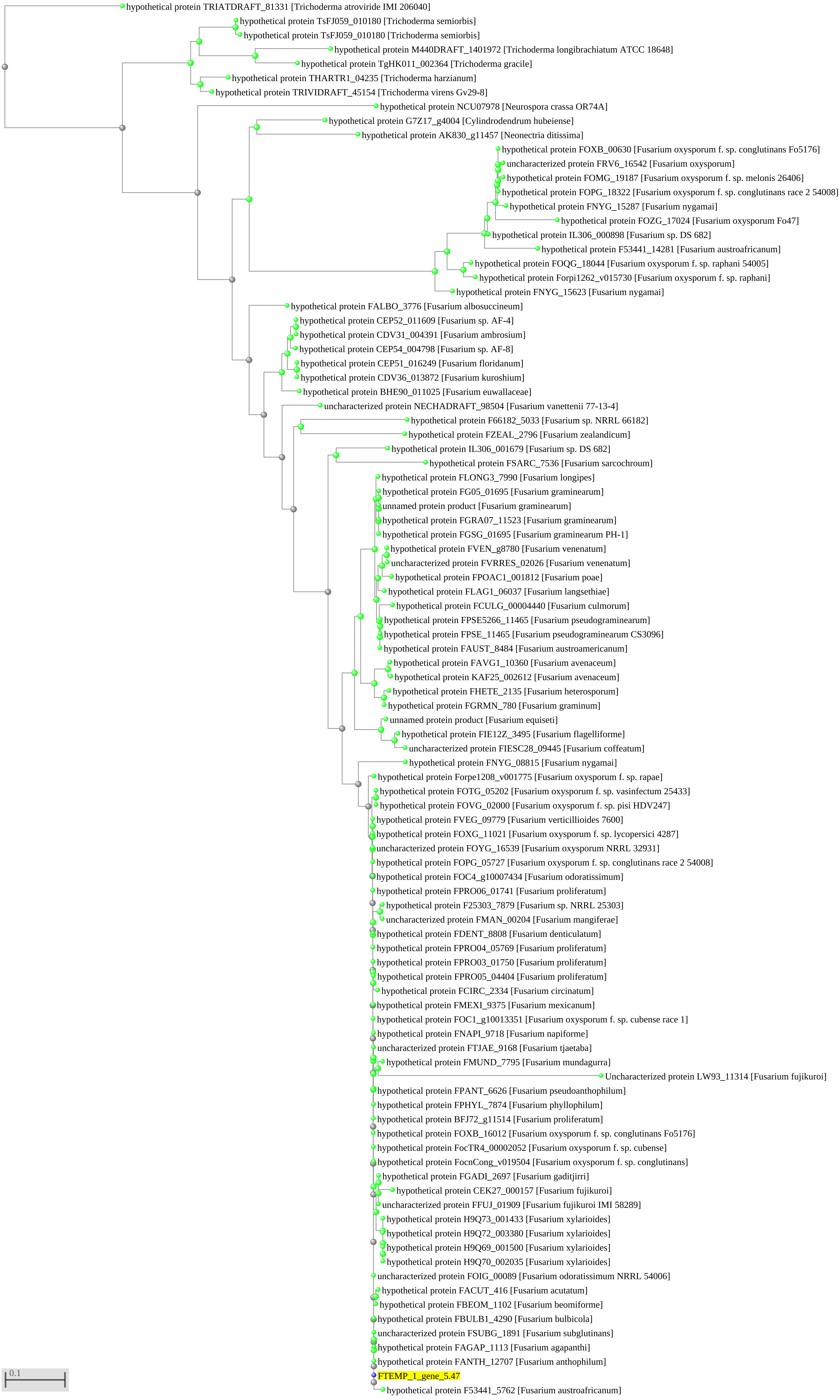

hypothetical protein TRIATDRAFT_81331 [Trichoderma atroviride IMI 206040]
hypothetical protein TsFJ059_010180 [Trichoderma semiorbis]
hypothetical protein TsFJ059_010180 [Trichoderma semiorbis]
hypothetical protein M440DRAFT_1401972 [Trichoderma longibrachiatum ATCC 18648]
hypothetical protein TgHK011_002364 [Trichoderma gracile]
hypothetical protein THARTR1_04235 [Trichoderma harzianum]
hypothetical protein TRIVIDRAFT_45154 [Trichoderma virens Gv29-8]
hypothetical protein NCU07978 [Neurospora crassa OR74A]
hypothetical protein G7Z17_g4004 [Cylindrodendrum hubeiense]
hypothetical protein AK830_g11457 [Neonectria ditissima]
hypothetical protein FOXB_00630 [Fusarium oxysporum f. sp. conglutinans Fo5176]
uncharacterized protein FRV6_16542 [Fusarium oxysporum]
hypothetical protein FOMG_19187 [Fusarium oxysporum f. sp. melonis 26406]
hypothetical protein FOPG_18322 [Fusarium oxysporum f. sp. conglutinans race 2 54008]
hypothetical protein FNYG_15287 [Fusarium nygamai]
hypothetical protein FOZG_17024 [Fusarium oxysporum Fo47]
hypothetical protein IL306_000898 [Fusarium sp. DS 682]
hypothetical protein F53441_14281 [Fusarium austroafricanum]
hypothetical protein FOQG_18044 [Fusarium oxysporum f. sp. raphani 54005]
hypothetical protein Forpi1262_v015730 [Fusarium oxysporum f. sp. raphani]
hypothetical protein FNYG_15623 [Fusarium nygamai]
hypothetical protein FALBO_3776 [Fusarium albosuccineum]
hypothetical protein CEP52_011609 [Fusarium sp. AF-4]
hypothetical protein CDV31_004391 [Fusarium ambrosium]
hypothetical protein CEP54_004798 [Fusarium sp. AF-8]
hypothetical protein CEP51_016249 [Fusarium floridanum]
hypothetical protein CDV36_013872 [Fusarium kuroshium]
hypothetical protein BHE90_011025 [Fusarium euwallaceae]
uncharacterized protein NECHADRAFT_98504 [Fusarium vanettenii 77-13-4]
hypothetical protein F66182_5033 [Fusarium sp. NRRL 66182]
hypothetical protein FZEAL_2796 [Fusarium zealandicum]
hypothetical protein IL306_001679 [Fusarium sp. DS 682]
hypothetical protein FSARC_7536 [Fusarium sarcochroum]
hypothetical protein FLONG3_7990 [Fusarium longipes]
hypothetical protein FG05_01695 [Fusarium graminearum]
unnamed protein product [Fusarium graminearum]
hypothetical protein FGRA07_11523 [Fusarium graminearum]
hypothetical protein FGSG_01695 [Fusarium graminearum PH-1]
hypothetical protein FVEN_g8780 [Fusarium venenatum]
uncharacterized protein FVRRES_02026 [Fusarium venenatum]
hypothetical protein FPOAC1_001812 [Fusarium poae]
hypothetical protein FLAG1_06037 [Fusarium langsethiae]
hypothetical protein FCULG_00004440 [Fusarium culmorum]
hypothetical protein FPSE5266_11465 [Fusarium pseudograminearum]
hypothetical protein FPSE_11465 [Fusarium pseudograminearum CS3096]
hypothetical protein FAUST_8484 [Fusarium austroamericanum]
hypothetical protein FAVG1_10360 [Fusarium avenaceum]
hypothetical protein KAF25_002612 [Fusarium avenaceum]
hypothetical protein FHETE_2135 [Fusarium heterosporum]
hypothetical protein FGRMN_780 [Fusarium graminum]
unnamed protein product [Fusarium equiseti]
hypothetical protein FIE12Z_3495 [Fusarium flagelliforme]
uncharacterized protein FIESC28_09445 [Fusarium coffeatum]
hypothetical protein FNYG_08815 [Fusarium nygamai]
hypothetical protein Forpe1208_v001775 [Fusarium oxysporum f. sp. rapae]
hypothetical protein FOTG_05202 [Fusarium oxysporum f. sp. vasinfectum 25433]
hypothetical protein FOVG_02000 [Fusarium oxysporum f. sp. pisi HDV247]
hypothetical protein FVEG_09779 [Fusarium verticillioides 7600]
hypothetical protein FOXG_11021 [Fusarium oxysporum f. sp. lycopersici 4287]
uncharacterized protein FOYG_16539 [Fusarium oxysporum NRRL 32931]
hypothetical protein FOPG_05727 [Fusarium oxysporum f. sp. conglutinans race 2 54008]
hypothetical protein FOC4_g10007434 [Fusarium odoratissimum]
hypothetical protein FPRO06_01741 [Fusarium proliferatum]
hypothetical protein F25303_7879 [Fusarium sp. NRRL 25303]
uncharacterized protein FMAN_00204 [Fusarium mangiferae]
hypothetical protein FDENT_8808 [Fusarium denticulatum]
hypothetical protein FPRO04_05769 [Fusarium proliferatum]
hypothetical protein FPRO03_01750 [Fusarium proliferatum]
hypothetical protein FPRO05_04404 [Fusarium proliferatum]
hypothetical protein FCIRC_2334 [Fusarium circinatum]
hypothetical protein FMEXI_9375 [Fusarium mexicanum]
hypothetical protein FOC1_g10013351 [Fusarium oxysporum f. sp. cubense race 1]
hypothetical protein FNAPI_9718 [Fusarium napiforme]
uncharacterized protein FTJAE_9168 [Fusarium tjaetaba]
hypothetical protein FMUND_7795 [Fusarium mundagurra]
Uncharacterized protein LW93_11314 [Fusarium fujikuroi]
hypothetical protein FPANT_6626 [Fusarium pseudoanthophilum]
hypothetical protein FPHYL_7874 [Fusarium phyllophilum]
hypothetical protein BFJ72_g11514 [Fusarium proliferatum]
hypothetical protein FOXB_16012 [Fusarium oxysporum f. sp. conglutinans Fo5176]
hypothetical protein FocTR4_00002052 [Fusarium oxysporum f. sp. cubense]
hypothetical protein FocnCong_v019504 [Fusarium oxysporum f. sp. conglutinans]
hypothetical protein FGADI_2697 [Fusarium gaditjirri]
hypothetical protein CEK27_000157 [Fusarium fujikuroi]
uncharacterized protein FFUJ_01909 [Fusarium fujikuroi IMI 58289]
hypothetical protein H9Q73_001433 [Fusarium xylarioides]
hypothetical protein H9Q72_003380 [Fusarium xylarioides]
hypothetical protein H9Q69_001500 [Fusarium xylarioides]
hypothetical protein H9Q70_002035 [Fusarium xylarioides]
uncharacterized protein FOIG_00089 [Fusarium odoratissimum NRRL 54006]
hypothetical protein FACUT_416 [Fusarium acutatum]
hypothetical protein FBEOM_1102 [Fusarium beomiforme]
hypothetical protein FBULB1_4290 [Fusarium bulbicola]
uncharacterized protein FSUBG_1891 [Fusarium subglutinans]
hypothetical protein FAGAP_1113 [Fusarium agapanthi]
hypothetical protein FANTH_12707 [Fusarium anthophilum]
FTEMP_1_gene_5.47
hypothetical protein F53441_5762 [Fusarium austroafricanum]
0.1

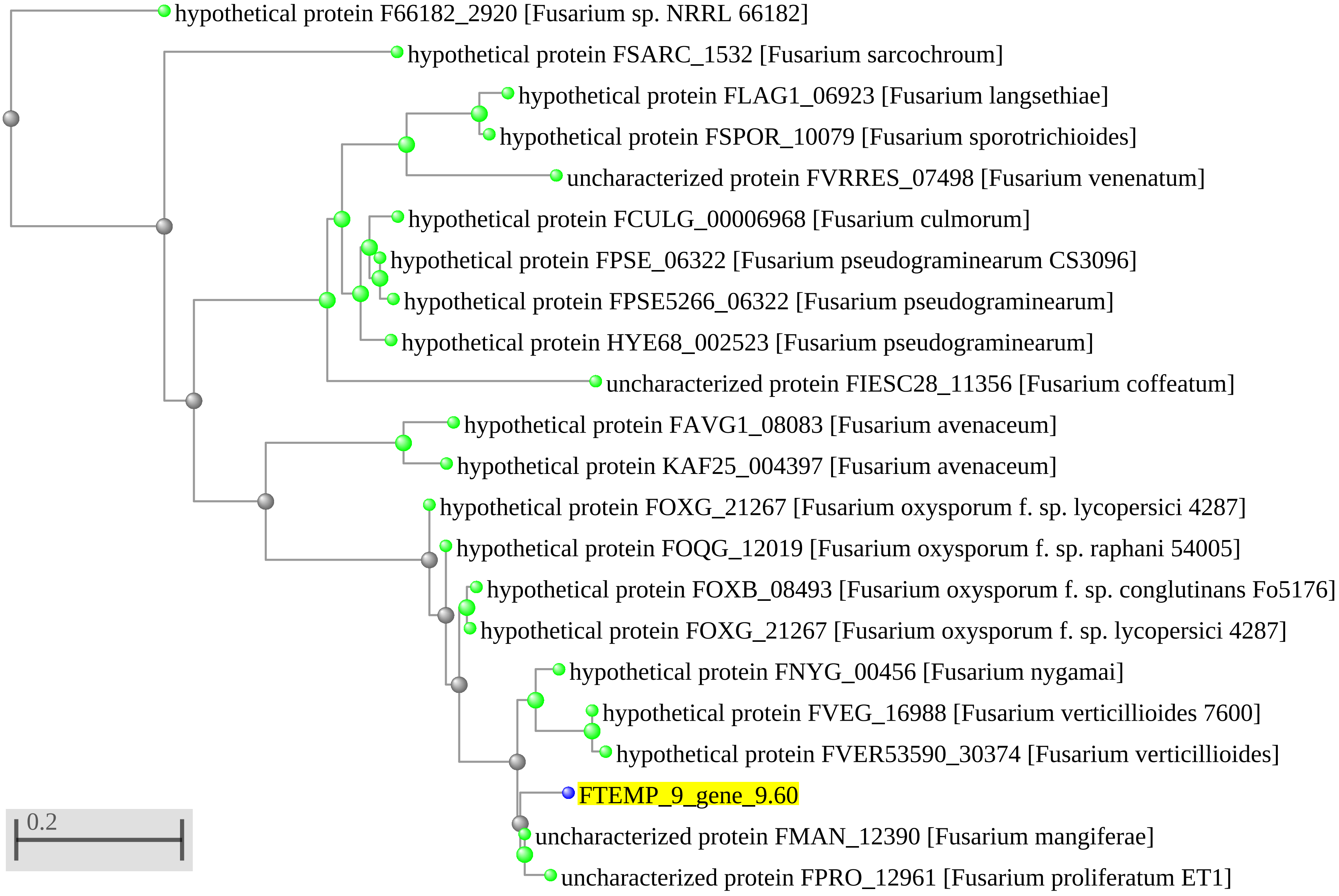
hypothetical protein F66182_2920 [Fusarium sp. NRRL 66182]
hypothetical protein FSARC_1532 [Fusarium sarcochroum]
hypothetical protein FLAG1_06923 [Fusarium langsethiae]
hypothetical protein FSPOR_10079 [Fusarium sporotrichioides]
uncharacterized protein FVRRES_07498 [Fusarium venenatum]
hypothetical protein FCULG_00006968 [Fusarium culmorum]
hypothetical protein FPSE_06322 [Fusarium pseudograminearum CS3096]
hypothetical protein FPSE5266_06322 [Fusarium pseudograminearum]
hypothetical protein HYE68_002523 [Fusarium pseudograminearum]
uncharacterized protein FIESC28_11356 [Fusarium coffeatum]
hypothetical protein FAVG1_08083 [Fusarium avenaceum]
hypothetical protein KAF25_004397 [Fusarium avenaceum]
hypothetical protein FOXG_21267 [Fusarium oxysporum f. sp. lycopersici 4287]
hypothetical protein FOQG_12019 [Fusarium oxysporum f. sp. raphani 54005]
hypothetical protein FOXB_08493 [Fusarium oxysporum f. sp. conglutinans Fo5176]
hypothetical protein FOXG_21267 [Fusarium oxysporum f. sp. lycopersici 4287]
hypothetical protein FNYG_00456 [Fusarium nygamai]
hypothetical protein FVEG_16988 [Fusarium verticillioides 7600]
hypothetical protein FVER53590_30374 [Fusarium verticillioides]
FTEMP_9_gene_9.60
uncharacterized protein FMAN_12390 [Fusarium mangiferae]
uncharacterized protein FPRO_12961 [Fusarium proliferatum ET1]
0.2

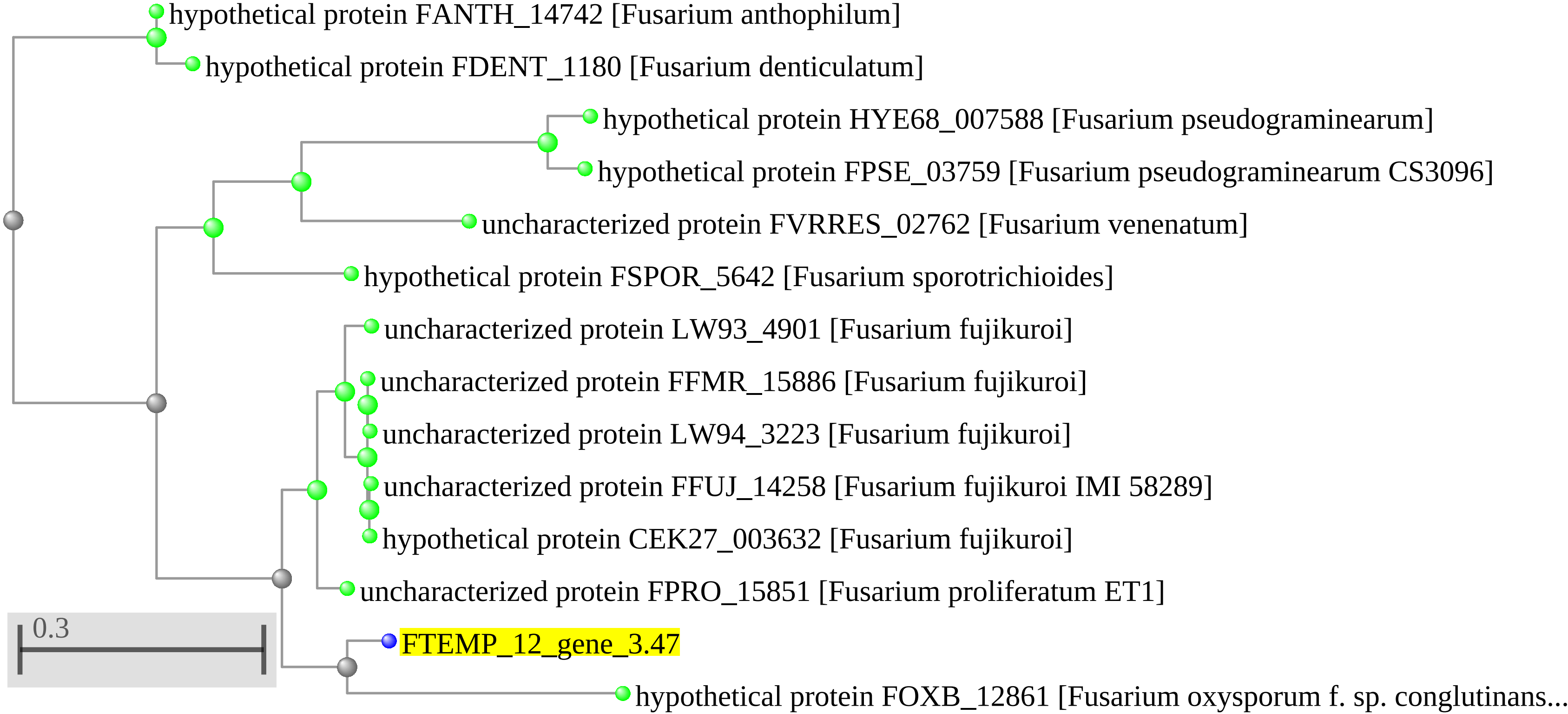
hypothetical protein FANTH_14742 [Fusarium anthophilum]
hypothetical protein FDENT_1180 [Fusarium denticulatum]
hypothetical protein HYE68_007588 [Fusarium pseudograminearum]
hypothetical protein FPSE_03759 [Fusarium pseudograminearum CS3096]
uncharacterized protein FVRRES_02762 [Fusarium venenatum]
hypothetical protein FSPOR_5642 [Fusarium sporotrichioides]
uncharacterized protein LW93_4901 [Fusarium fujikuroi]
uncharacterized protein FFMR_15886 [Fusarium fujikuroi]
uncharacterized protein LW94_3223 [Fusarium fujikuroi]
uncharacterized protein FFUJ_14258 [Fusarium fujikuroi IMI 58289]
hypothetical protein CEK27_003632 [Fusarium fujikuroi]
uncharacterized protein FPRO_15851 [Fusarium proliferatum ET1]
0.3
FTEMP_12_gene_3.47
hypothetical protein FOXB_12861 [Fusarium oxysporum f. sp. conglutinans...
