# Supplementary material for: Characterization of Host-Specific Genes from Pine- and Grass-Associated Species of the Fusarium fujikuroi Species Complex
Source: Pathogens. 2022 Jul 29;11(8):858. doi: 10.3390/pathogens11080858 (PMC9415769; doi:10.3390/pathogens11080858)
Supplement: Supplementary file 1 [file pathogens-11-00858-s001.zip › Supplemental Figures/Figure S4.pdf]

Figure S4. Host-range-associated genes with ancestral origins that emerged within the broader *Fusarium* outside the FFSC and FOSC. Host-range-associated genes investigated are highlighted in yellow; FCIR = *Fusarium circinatum* and FTEMP = *Fusarium temperatum.*

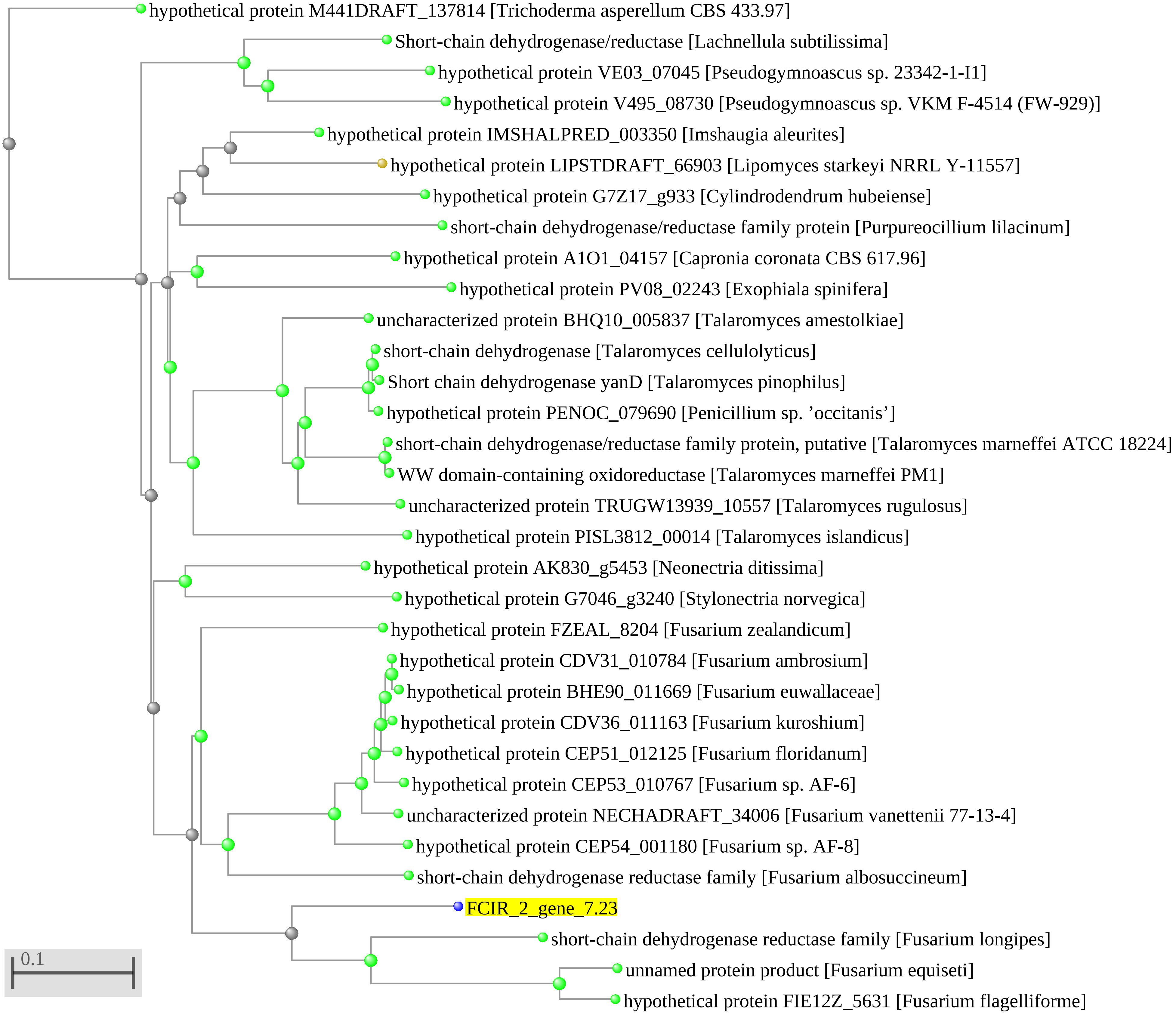
hypothetical protein M441DRAFT_137814 [Trichoderma asperellum CBS 433.97]
Short-chain dehydrogenase/reductase [Lachnellula subtilissima]
hypothetical protein VE03_07045 [Pseudogymnoascus sp. 23342-1-I1]
hypothetical protein V495_08730 [Pseudogymnoascus sp. VKM F-4514 (FW-929)]
hypothetical protein IMSHALPRED_003350 [Imshaugia aleurites]
hypothetical protein LIPSTDRAFT_66903 [Lipomyces starkeyi NRRL Y-11557]
hypothetical protein G7Z17_g933 [Cylindrodendrum hubeiense]
short-chain dehydrogenase/reductase family protein [Purpureocillium lilacinum]
hypothetical protein A1O1_04157 [Capronia coronata CBS 617.96]
hypothetical protein PV08_02243 [Exophiala spinifera]
uncharacterized protein BHQ10_005837 [Talaromyces amestolkiae]
short-chain dehydrogenase [Talaromyces cellulolyticus]
Short chain dehydrogenase yanD [Talaromyces pinophilus]
hypothetical protein PENOC_079690 [Penicillium sp. 'occitanis']
short-chain dehydrogenase/reductase family protein, putative [Talaromyces marneffei ATCC 18224]
WW domain-containing oxidoreductase [Talaromyces marneffei PM1]
uncharacterized protein TRUGW13939_10557 [Talaromyces rugulosus]
hypothetical protein PISL3812_00014 [Talaromyces islandicus]
hypothetical protein AK830_g5453 [Neonectria ditissima]
hypothetical protein G7046_g3240 [Stylonectria norvegica]
hypothetical protein FZEAL_8204 [Fusarium zealandicum]
hypothetical protein CDV31_010784 [Fusarium ambrosium]
hypothetical protein BHE90_011669 [Fusarium euwallaceae]
hypothetical protein CDV36_011163 [Fusarium kuroshium]
hypothetical protein CEP51_012125 [Fusarium floridanum]
hypothetical protein CEP53_010767 [Fusarium sp. AF-6]
uncharacterized protein NECHADRAFT_34006 [Fusarium vanettenii 77-13-4]
hypothetical protein CEP54_001180 [Fusarium sp. AF-8]
short-chain dehydrogenase reductase family [Fusarium albosuccineum]
FCIR_2_gene_7.23
short-chain dehydrogenase reductase family [Fusarium longipes]
unnamed protein product [Fusarium equiseti]
hypothetical protein FIE12Z_5631 [Fusarium flagelliforme]
0.1

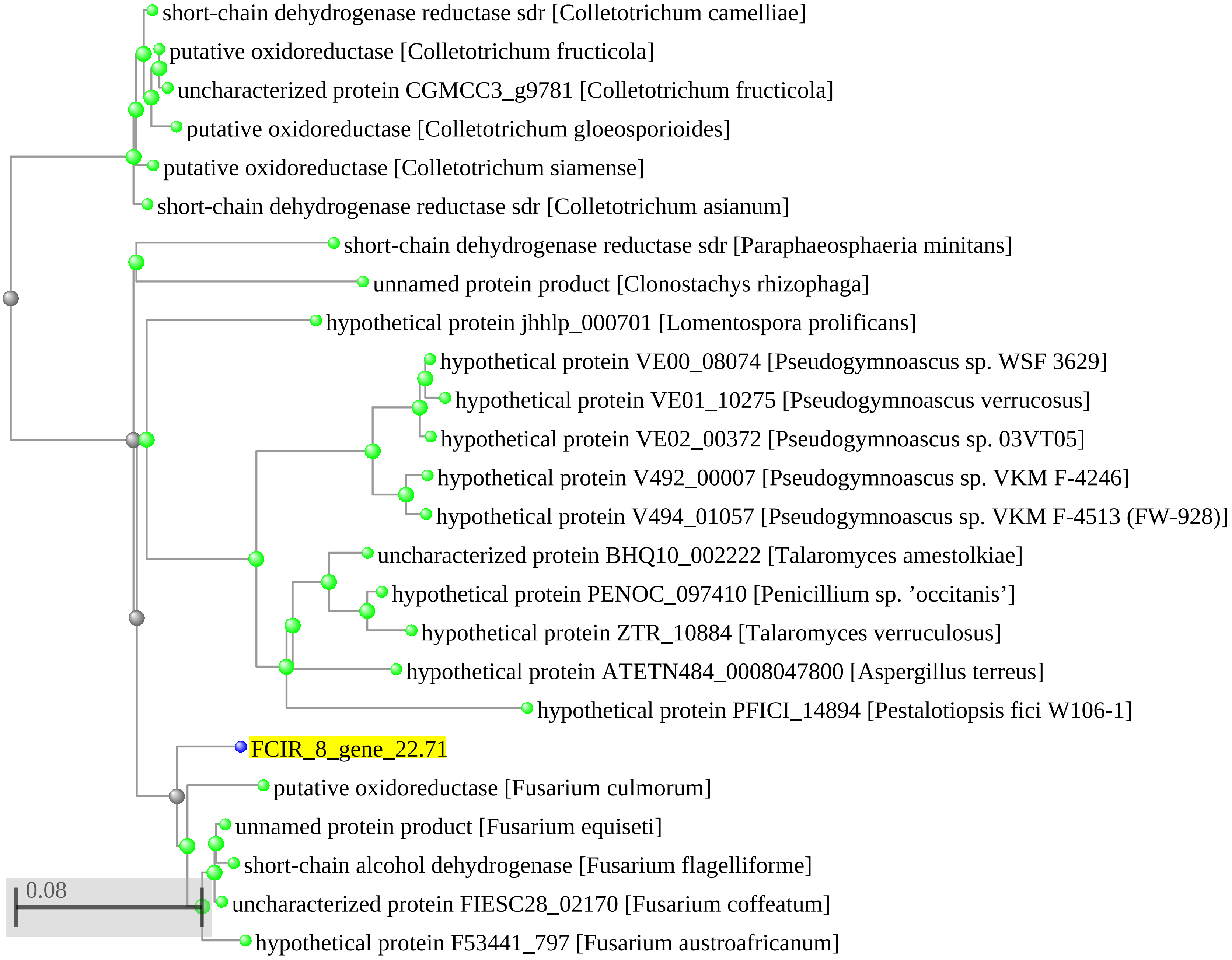
short-chain dehydrogenase reductase sdr [Colletotrichum camelliae]
putative oxidoreductase [Colletotrichum fructicola]
uncharacterized protein CGMCC3_g9781 [Colletotrichum fructicola]
putative oxidoreductase [Colletotrichum gloeosporioides]
putative oxidoreductase [Colletotrichum siamense]
short-chain dehydrogenase reductase sdr [Colletotrichum asianum]
short-chain dehydrogenase reductase sdr [Paraphaeosphaeria minitans]
unnamed protein product [Clonostachys rhizophaga]
hypothetical protein jhhlp_000701 [Lomentospora prolificans]
hypothetical protein VE00_08074 [Pseudogymnoascus sp. WSF 3629]
hypothetical protein VE01_10275 [Pseudogymnoascus verrucosus]
hypothetical protein VE02_00372 [Pseudogymnoascus sp. 03VT05]
hypothetical protein V492_00007 [Pseudogymnoascus sp. VKM F-4246]
hypothetical protein V494_01057 [Pseudogymnoascus sp. VKM F-4513 (FW-928)]
uncharacterized protein BHQ10_002222 [Talaromyces amestolkiae]
hypothetical protein PENOC_097410 [Penicillium sp. ’occitanis’]
hypothetical protein ZTR_10884 [Talaromyces verruculosus]
hypothetical protein ATETN484_0008047800 [Aspergillus terreus]
hypothetical protein PFICI_14894 [Pestalotiopsis fici W106-1]
FCIR_8_gene_22.71
putative oxidoreductase [Fusarium culmorum]
unnamed protein product [Fusarium equiseti]
short-chain alcohol dehydrogenase [Fusarium flagelliforme]
uncharacterized protein FIESC28_02170 [Fusarium coffeatum]
hypothetical protein F53441_797 [Fusarium austroafricanum]
0.08

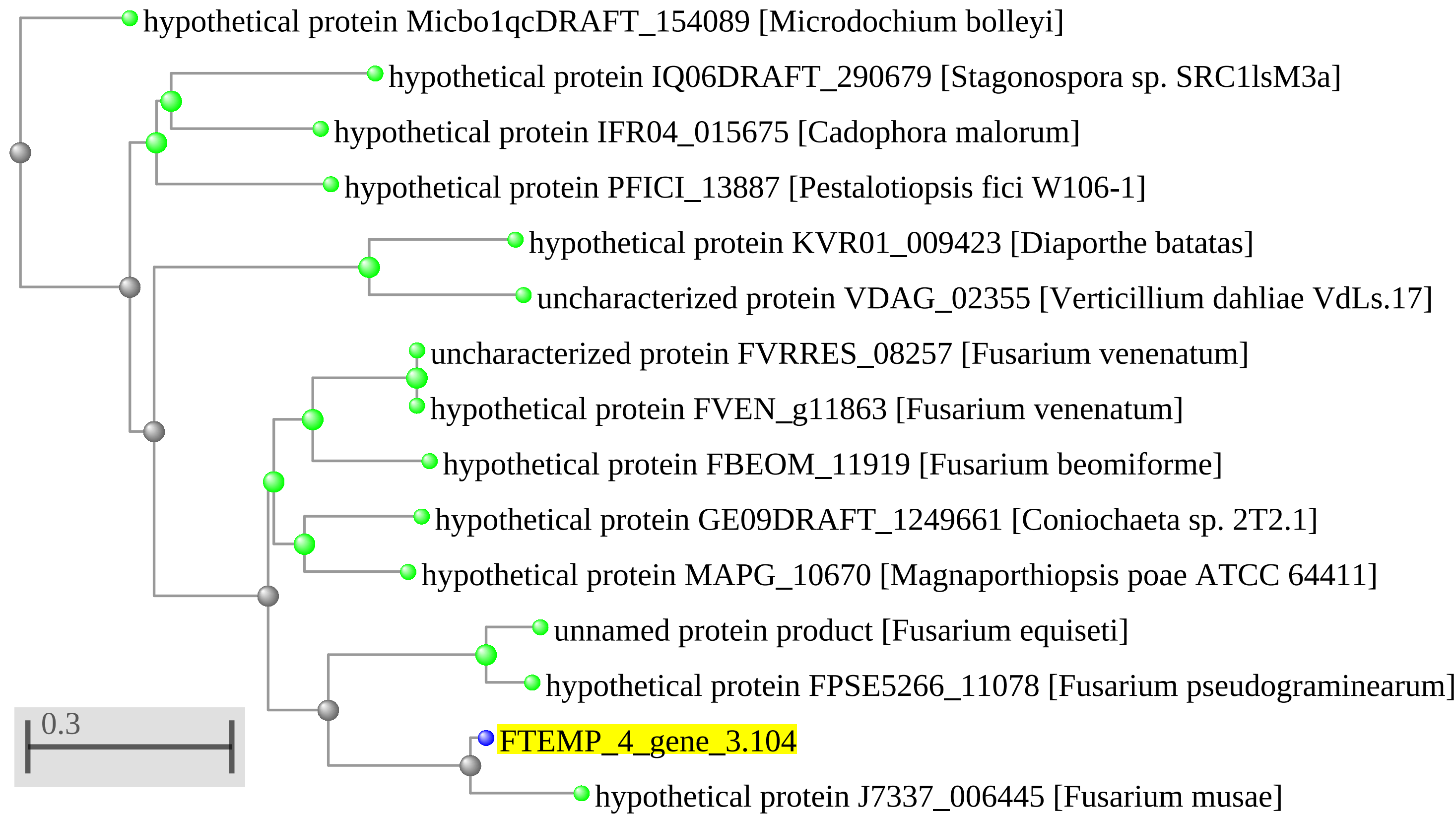
hypothetical protein Micbo1qcDRAFT_154089 [Microdochium bolleyi]
hypothetical protein IQ06DRAFT_290679 [Stagonospora sp. SRC1lsM3a]
hypothetical protein IFR04_015675 [Cadophora malorum]
hypothetical protein PFICI_13887 [Pestalotiopsis fici W106-1]
hypothetical protein KVR01_009423 [Diaporthe batatas]
uncharacterized protein VDAG_02355 [Verticillium dahliae VdLs.17]
uncharacterized protein FVRRES_08257 [Fusarium venenatum]
hypothetical protein FVEN_g11863 [Fusarium venenatum]
hypothetical protein FBEOM_11919 [Fusarium beomiforme]
hypothetical protein GE09DRAFT_1249661 [Coniochaeta sp. 2T2.1]
hypothetical protein MAPG_10670 [Magnaporthiopsis poae ATCC 64411]
unnamed protein product [Fusarium equiseti]
hypothetical protein FPSE5266_11078 [Fusarium pseudograminearum]
0.3
FTEMP_4_gene_3.104
hypothetical protein J7337_006445 [Fusarium musae]

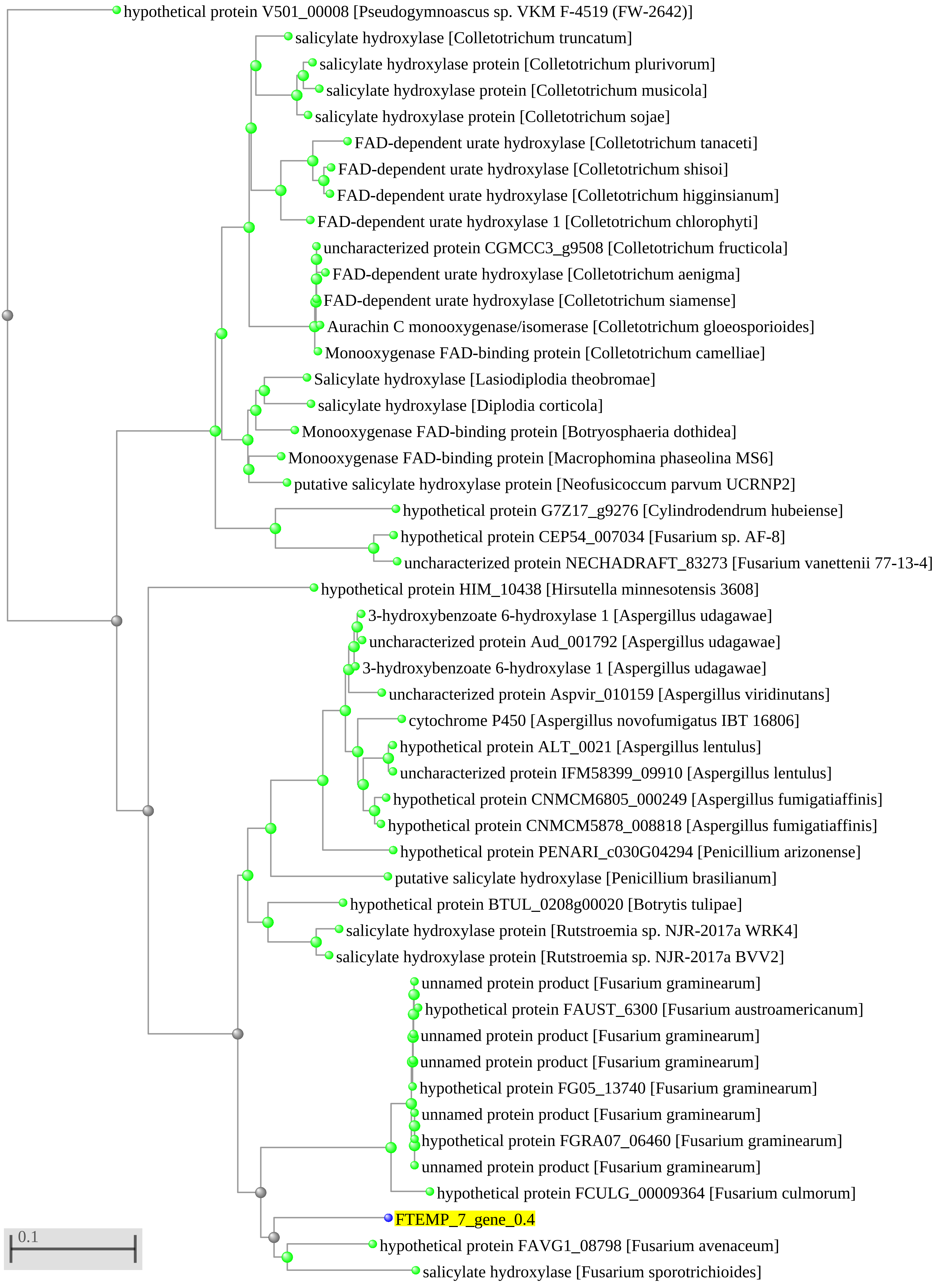
hypothetical protein V501_00008 [Pseudogymnoascus sp. VKM F-4519 (FW-2642)]
salicylate hydroxylase [Colletotrichum truncatum]
salicylate hydroxylase protein [Colletotrichum plurivorum]
salicylate hydroxylase protein [Colletotrichum musicola]
salicylate hydroxylase protein [Colletotrichum sojae]
FAD-dependent urate hydroxylase [Colletotrichum tanaceti]
FAD-dependent urate hydroxylase [Colletotrichum shisoi]
FAD-dependent urate hydroxylase [Colletotrichum higginsianum]
FAD-dependent urate hydroxylase 1 [Colletotrichum chlorophyti]
uncharacterized protein CGMCC3_g9508 [Colletotrichum fructicola]
FAD-dependent urate hydroxylase [Colletotrichum aenigma]
FAD-dependent urate hydroxylase [Colletotrichum siamense]
Aurachin C monooxygenase/isomerase [Colletotrichum gloeosporioides]
Monooxygenase FAD-binding protein [Colletotrichum camelliae]
Salicylate hydroxylase [Lasiodiplodia theobromae]
salicylate hydroxylase [Diplodia corticola]
Monooxygenase FAD-binding protein [Botryosphaeria dothidea]
Monooxygenase FAD-binding protein [Macrophomina phaseolina MS6]
putative salicylate hydroxylase protein [Neofusicoccum parvum UCRNP2]
hypothetical protein G7Z17_g9276 [Cylindrodendrum hubeiense]
hypothetical protein CEP54_007034 [Fusarium sp. AF-8]
uncharacterized protein NECHADRAFT_83273 [Fusarium vanettenii 77-13-4]
hypothetical protein HIM_10438 [Hirsutella minnesotensis 3608]
3-hydroxybenzoate 6-hydroxylase 1 [Aspergillus udagawae]
uncharacterized protein Aud_001792 [Aspergillus udagawae]
3-hydroxybenzoate 6-hydroxylase 1 [Aspergillus udagawae]
uncharacterized protein Aspvir_010159 [Aspergillus viridinutans]
cytochrome P450 [Aspergillus novofumigatus IBT 16806]
hypothetical protein ALT_0021 [Aspergillus lentulus]
uncharacterized protein IFM58399_09910 [Aspergillus lentulus]
hypothetical protein CNMCM6805_000249 [Aspergillus fumigatiaffinis]
hypothetical protein CNMCM5878_008818 [Aspergillus fumigatiaffinis]
hypothetical protein PENARI_c030G04294 [Penicillium arizonense]
putative salicylate hydroxylase [Penicillium brasilianum]
hypothetical protein BTUL_0208g00020 [Botrytis tulipae]
salicylate hydroxylase protein [Rutstroemia sp. NJR-2017a WRK4]
salicylate hydroxylase protein [Rutstroemia sp. NJR-2017a BVV2]
unnamed protein product [Fusarium graminearum]
hypothetical protein FAUST_6300 [Fusarium austroamericanum]
unnamed protein product [Fusarium graminearum]
unnamed protein product [Fusarium graminearum]
hypothetical protein FG05_13740 [Fusarium graminearum]
unnamed protein product [Fusarium graminearum]
hypothetical protein FGRA07_06460 [Fusarium graminearum]
unnamed protein product [Fusarium graminearum]
hypothetical protein FCULG_00009364 [Fusarium culmorum]
FTEMP_7_gene_0.4
hypothetical protein FAVG1_08798 [Fusarium avenaceum]
salicylate hydroxylase [Fusarium sporotrichioides]
0.1
