# Supplementary material for: Characterization of Host-Specific Genes from Pine- and Grass-Associated Species of the Fusarium fujikuroi Species Complex
Source: Pathogens. 2022 Jul 29;11(8):858. doi: 10.3390/pathogens11080858 (PMC9415769; doi:10.3390/pathogens11080858)
Supplement: Supplementary file 1 [file pathogens-11-00858-s001.zip › Supplemental Figures/Figure S5.pdf]

Figure S5. Host-range-associated genes with less than 10 ancestral origin hits and mostly *Fusarium*. Host-range-associated genes investigated are highlighted in yellow; FCIR = *Fusarium circinatum* and FTEMP = *Fusarium temperatum.*

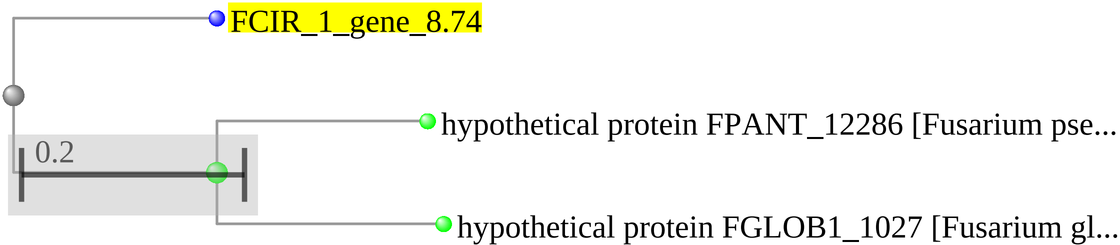
FCIR_1_gene_8.74
0.2
hypothetical protein FPANT_12286 [Fusarium pse...
hypothetical protein FGLOB1_1027 [Fusarium gl...

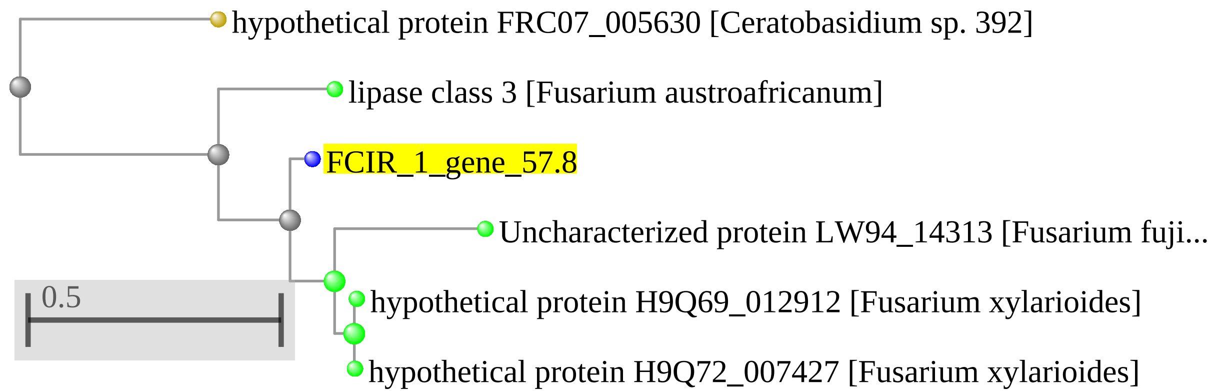
hypothetical protein FRC07_005630 [Ceratobasidium sp. 392]
lipase class 3 [Fusarium austroafricanum]
FCIR_1_gene_57.8
Uncharacterized protein LW94_14313 [Fusarium fuji...
hypothetical protein H9Q69_012912 [Fusarium xylarioides]
hypothetical protein H9Q72_007427 [Fusarium xylarioides]
0.5

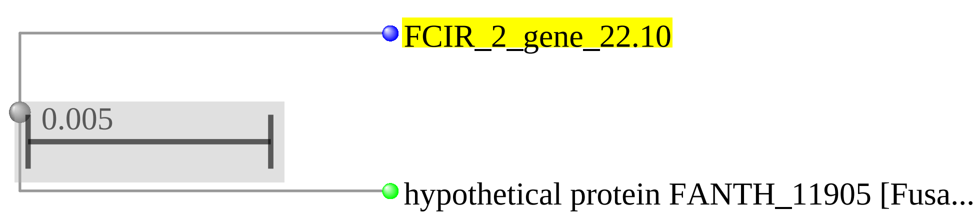
FCIR_2_gene_22.10
0.005
hypothetical protein FANTH_11905 [Fusa...

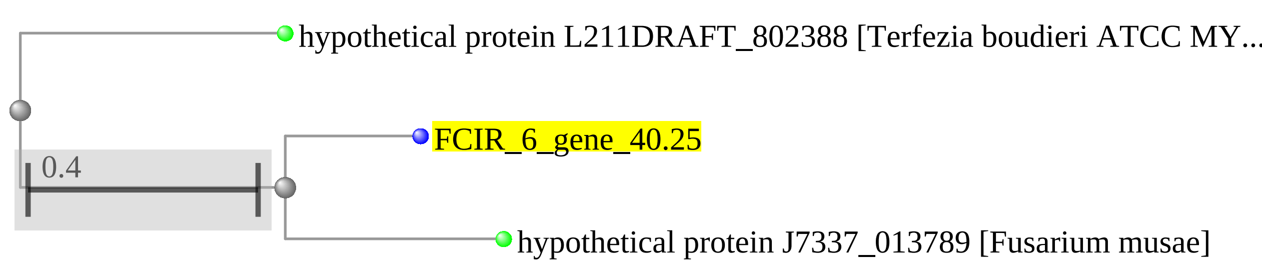
hypothetical protein L211DRAFT_802388 [Terfezia boudieri ATCC MY...
FCIR_6_gene_40.25
0.4
hypothetical protein J7337_013789 [Fusarium musae]

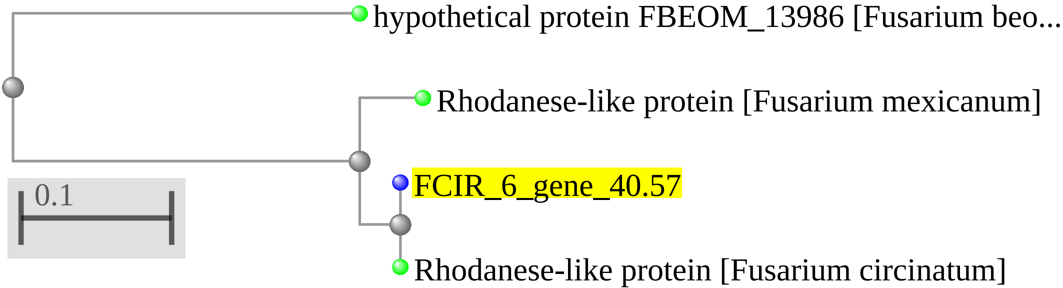
hypothetical protein FBEOM_13986 [Fusarium beo...
Rhodanese-like protein [Fusarium mexicanum]
FCIR_6_gene_40.57
Rhodanese-like protein [Fusarium circinatum]
0.1

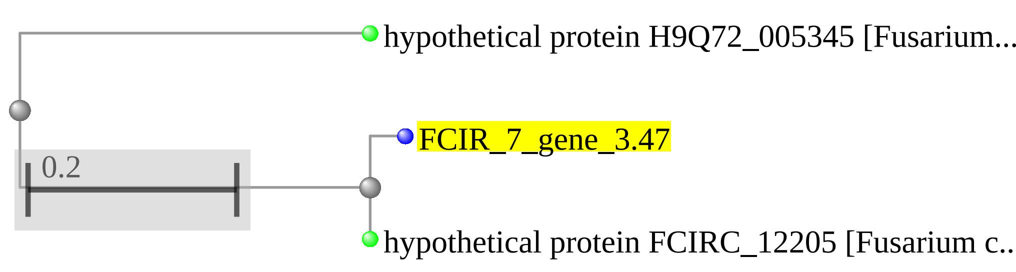
hypothetical protein H9Q72_005345 [Fusarium...
FCIR_7_gene_3.47
0.2
hypothetical protein FCIRC_12205 [Fusarium c..

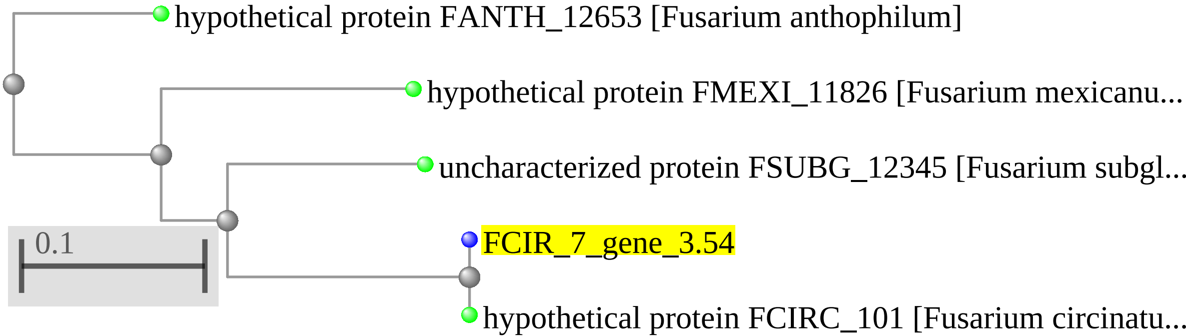
hypothetical protein FANTH_12653 [Fusarium anthophilum]
hypothetical protein FMEXI_11826 [Fusarium mexicanu...
uncharacterized protein FSUBG_12345 [Fusarium subgl...
FCIR_7_gene_3.54
hypothetical protein FCIRC_101 [Fusarium circinatu...
0.1

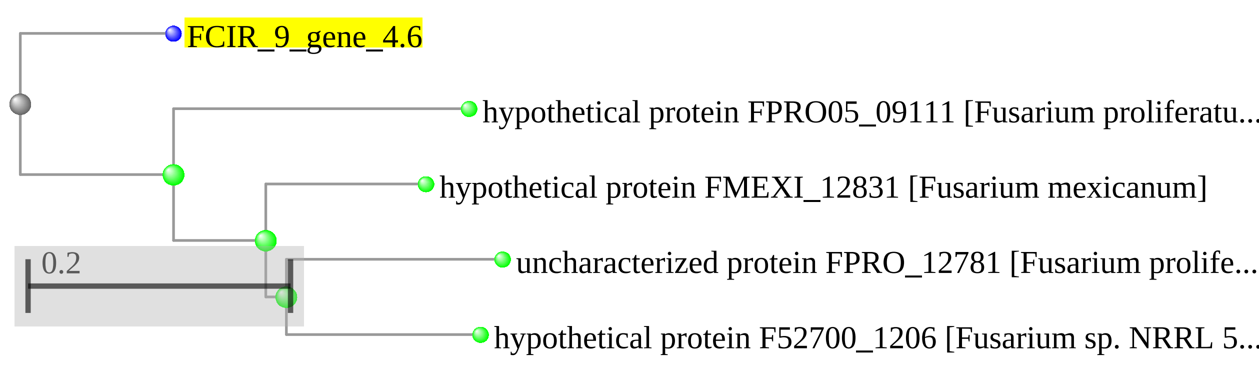
FCIR_9_gene_4.6
hypothetical protein FPRO05_09111 [Fusarium proliferatu...
hypothetical protein FMEXI_12831 [Fusarium mexicanum]
uncharacterized protein FPRO_12781 [Fusarium prolife...
hypothetical protein F52700_1206 [Fusarium sp. NRRL 5...
0.2

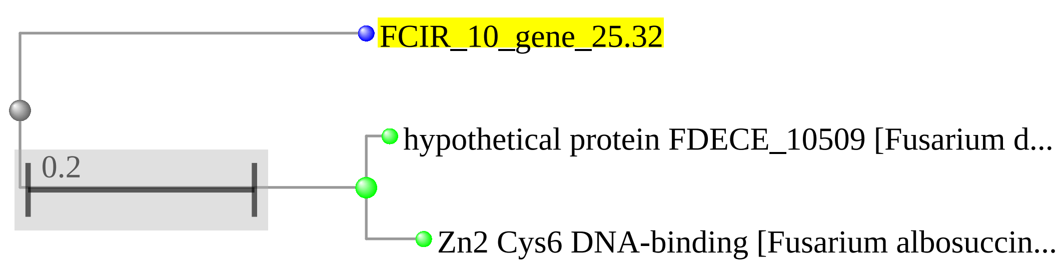
FCIR_10_gene_25.32
hypothetical protein FDECE_10509 [Fusarium d...
Zn2 Cys6 DNA-binding [Fusarium albosuccin...
0.2

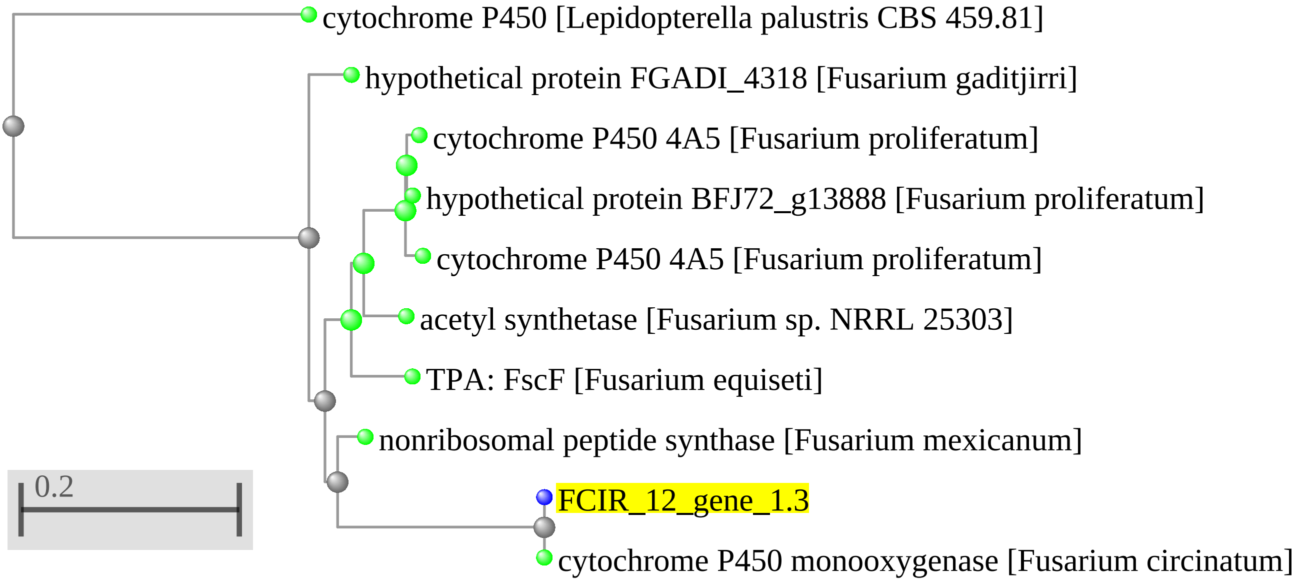
cytochrome P450 [Lepidopterella palustris CBS 459.81]
hypothetical protein FGADI_4318 [Fusarium gaditjirri]
cytochrome P450 4A5 [Fusarium proliferatum]
hypothetical protein BFJ72_g13888 [Fusarium proliferatum]
cytochrome P450 4A5 [Fusarium proliferatum]
acetyl synthetase [Fusarium sp. NRRL 25303]
TPA: FscF [Fusarium equiseti]
nonribosomal peptide synthase [Fusarium mexicanum]
0.2
FCIR_12_gene_1.3
cytochrome P450 monooxygenase [Fusarium circinatum]

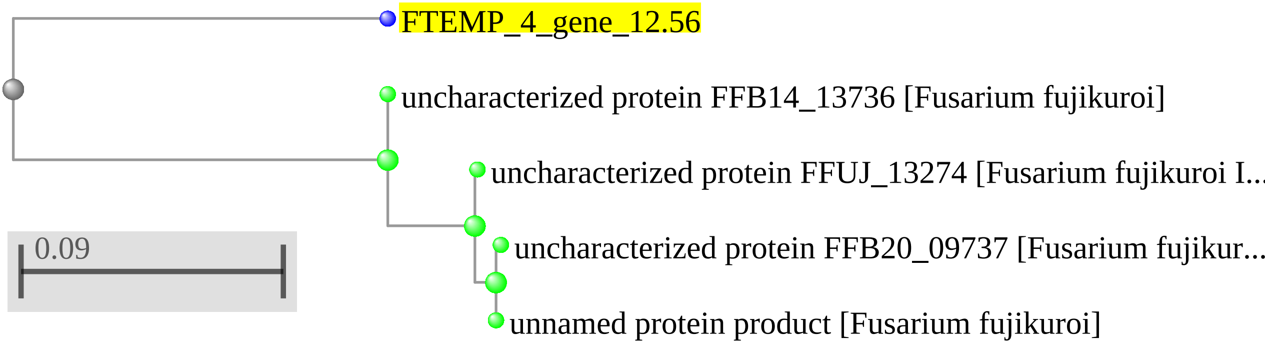
FTEMP_4_gene_12.56
uncharacterized protein FFB14_13736 [Fusarium fujikuroi]
uncharacterized protein FFUJ_13274 [Fusarium fujikuroi I...
uncharacterized protein FFB20_09737 [Fusarium fujikur...
unnamed protein product [Fusarium fujikuroi]
0.09

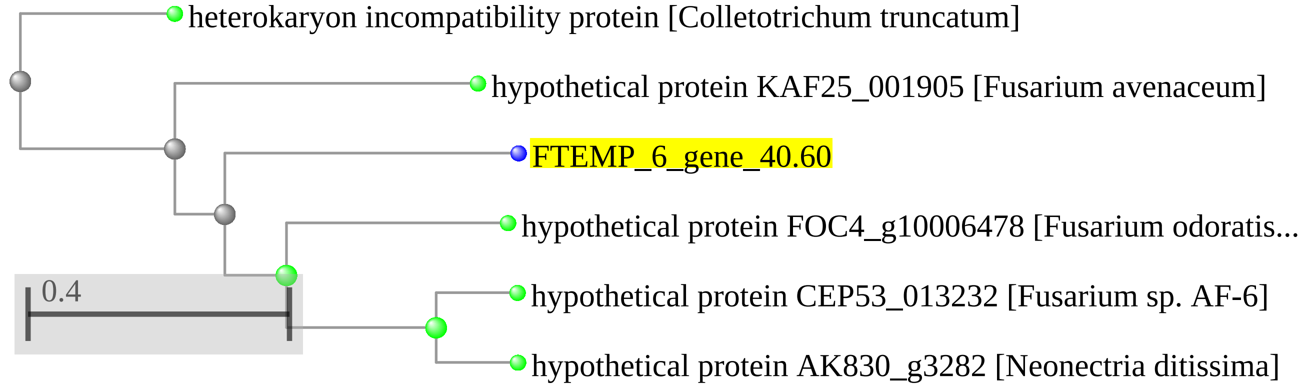
heterokaryon incompatibility protein [Colletotrichum truncatum]
hypothetical protein KAF25_001905 [Fusarium avenaceum]
FTEMP_6_gene_40.60
hypothetical protein FOC4_g10006478 [Fusarium odoratis...
0.4
hypothetical protein CEP53_013232 [Fusarium sp. AF-6]
hypothetical protein AK830_g3282 [Neonectria ditissima]

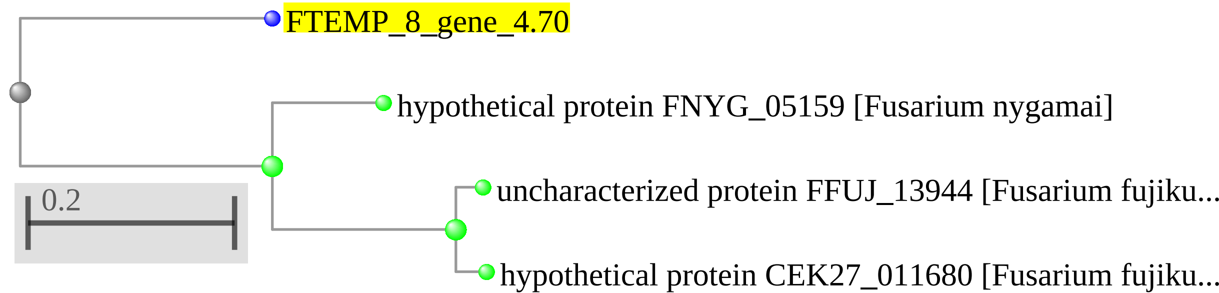
FTEMP_8_gene_4.70
hypothetical protein FNYG_05159 [Fusarium nygamai]
uncharacterized protein FFUJ_13944 [Fusarium fujiku...
hypothetical protein CEK27_011680 [Fusarium fujiku...
0.2

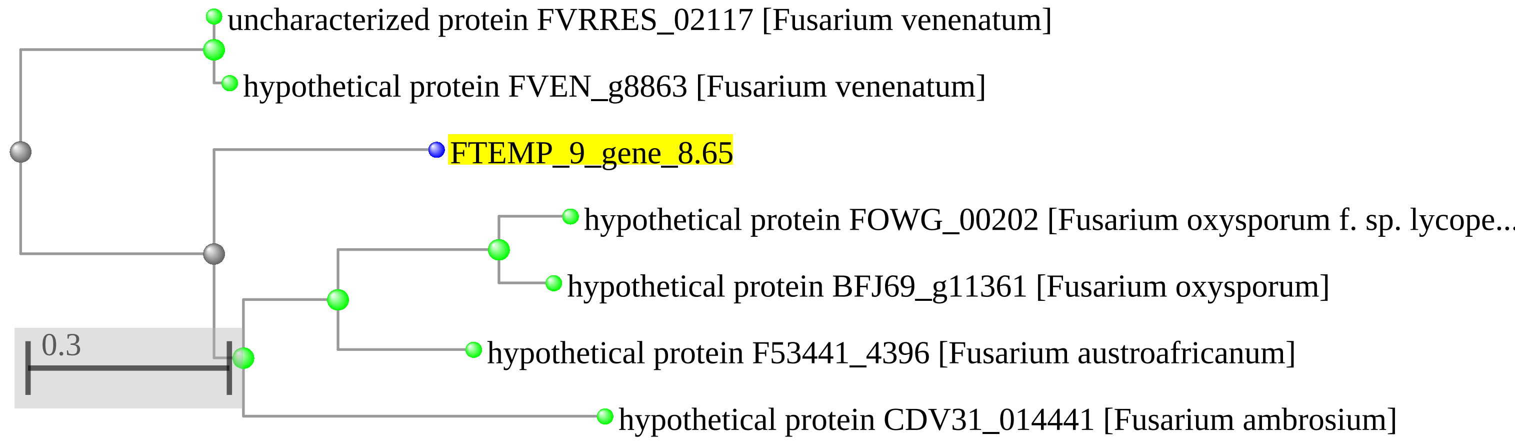
uncharacterized protein FVRRES_02117 [Fusarium venenatum]
hypothetical protein FVEN_g8863 [Fusarium venenatum]
FTEMP_9_gene_8.65
hypothetical protein FOWG_00202 [Fusarium oxysporum f. sp. lycope...
hypothetical protein BFJ69_g11361 [Fusarium oxysporum]
hypothetical protein F53441_4396 [Fusarium austroafricanum]
hypothetical protein CDV31_014441 [Fusarium ambrosium]
0.3

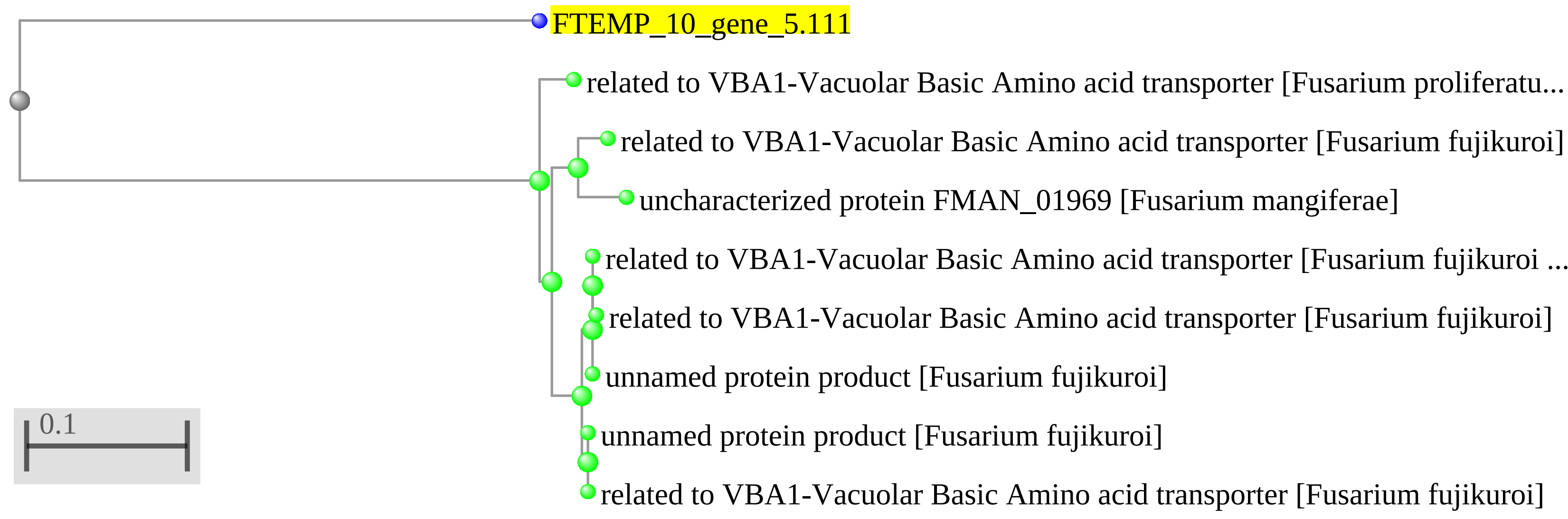
FTEMP_10_gene_5.111
related to VBA1-Vacuolar Basic Amino acid transporter [Fusarium proliferatu...
related to VBA1-Vacuolar Basic Amino acid transporter [Fusarium fujikuroi]
uncharacterized protein FMAN_01969 [Fusarium mangiferae]
related to VBA1-Vacuolar Basic Amino acid transporter [Fusarium fujikuroi ...
related to VBA1-Vacuolar Basic Amino acid transporter [Fusarium fujikuroi]
unnamed protein product [Fusarium fujikuroi]
unnamed protein product [Fusarium fujikuroi]
related to VBA1-Vacuolar Basic Amino acid transporter [Fusarium fujikuroi]
0.1

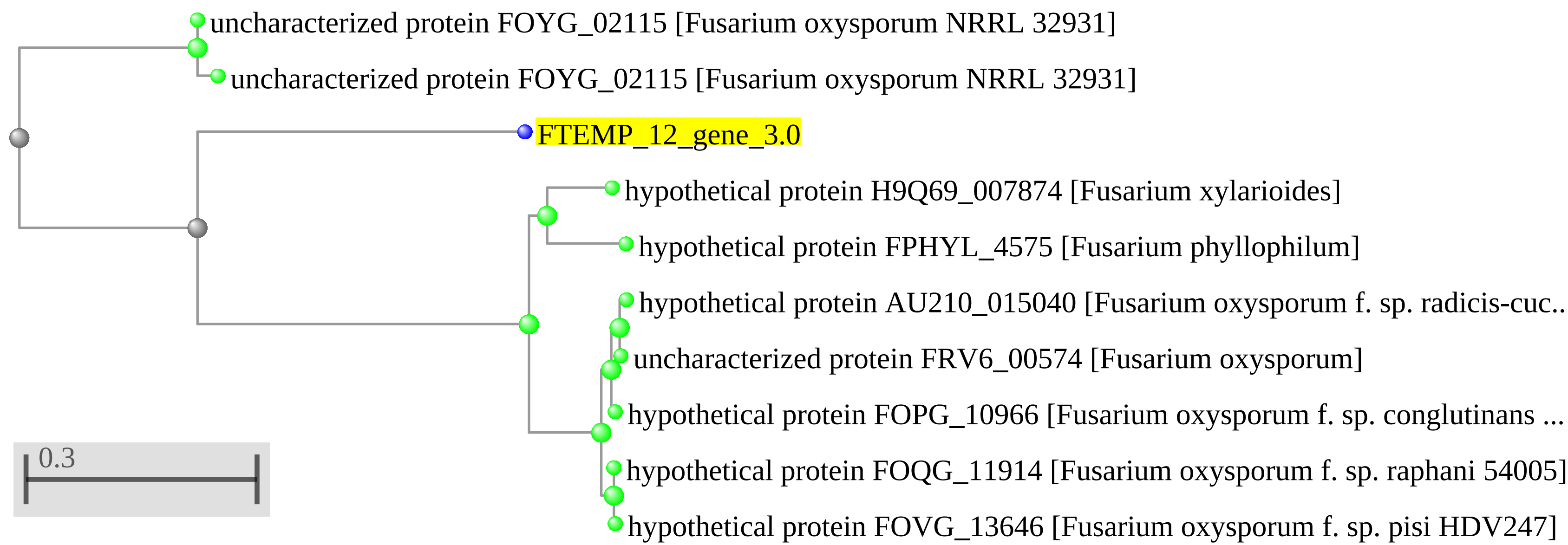

uncharacterized protein FOYG_02115 [Fusarium oxysporum NRRL 32931]
uncharacterized protein FOYG_02115 [Fusarium oxysporum NRRL 32931]
FTEMP_12_gene_3.0
hypothetical protein H9Q69_007874 [Fusarium xylarioides]
hypothetical protein FPHYL_4575 [Fusarium phyllophilum]
hypothetical protein AU210_015040 [Fusarium oxysporum f. sp. radicis-cuc...
uncharacterized protein FRV6_00574 [Fusarium oxysporum]
hypothetical protein FOPG_10966 [Fusarium oxysporum f. sp. conglutinans ...
hypothetical protein FOQG_11914 [Fusarium oxysporum f. sp. raphani 54005]
hypothetical protein FOVG_13646 [Fusarium oxysporum f. sp. pisi HDV247]
0.3

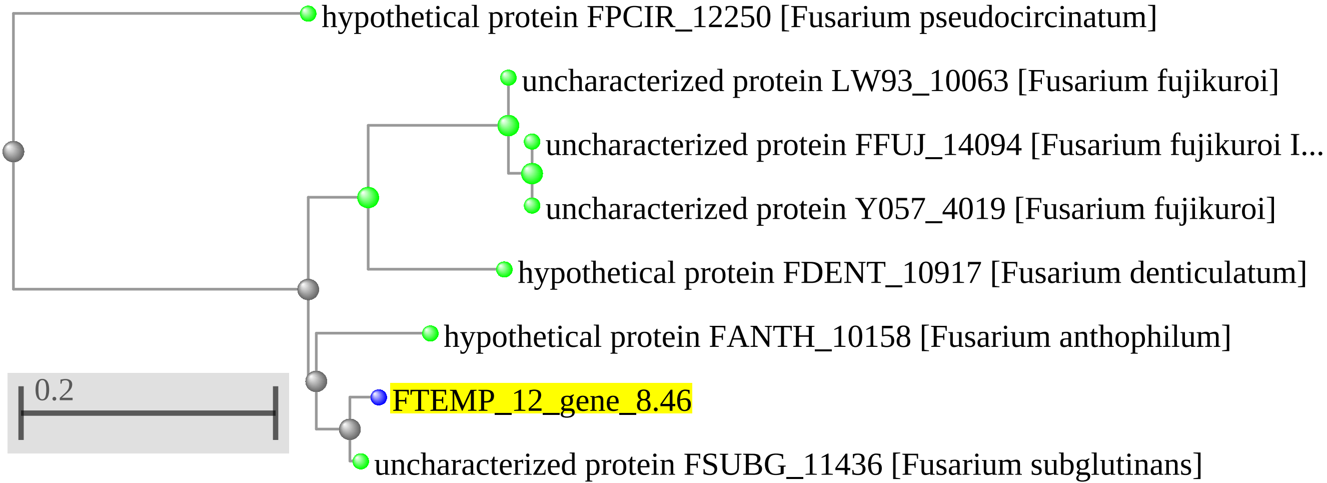
hypothetical protein FPCIR_12250 [Fusarium pseudocircinatum]
uncharacterized protein LW93_10063 [Fusarium fujikuroi]
uncharacterized protein FFUJ_14094 [Fusarium fujikuroi I...
uncharacterized protein Y057_4019 [Fusarium fujikuroi]
hypothetical protein FDENT_10917 [Fusarium denticulatum]
hypothetical protein FANTH_10158 [Fusarium anthophilum]
FTEMP_12_gene_8.46
uncharacterized protein FSUBG_11436 [Fusarium subglutinans]
0.2
