# Supplementary material for: Characterization of Host-Specific Genes from Pine- and Grass-Associated Species of the Fusarium fujikuroi Species Complex
Source: Pathogens. 2022 Jul 29;11(8):858. doi: 10.3390/pathogens11080858 (PMC9415769; doi:10.3390/pathogens11080858)
Supplement: Supplementary file 1 [file pathogens-11-00858-s001.zip › Supplemental Figures/Figure S6.pdf]

Figure S6. Host-range-associated genes with less than 10 ancestral origins hits and mostly not *Fusarium*. Host-range-associated genes investigated are highlighted in yellow; FCIR = *Fusarium circinatum* and FTEMP = *Fusarium temperatum*.

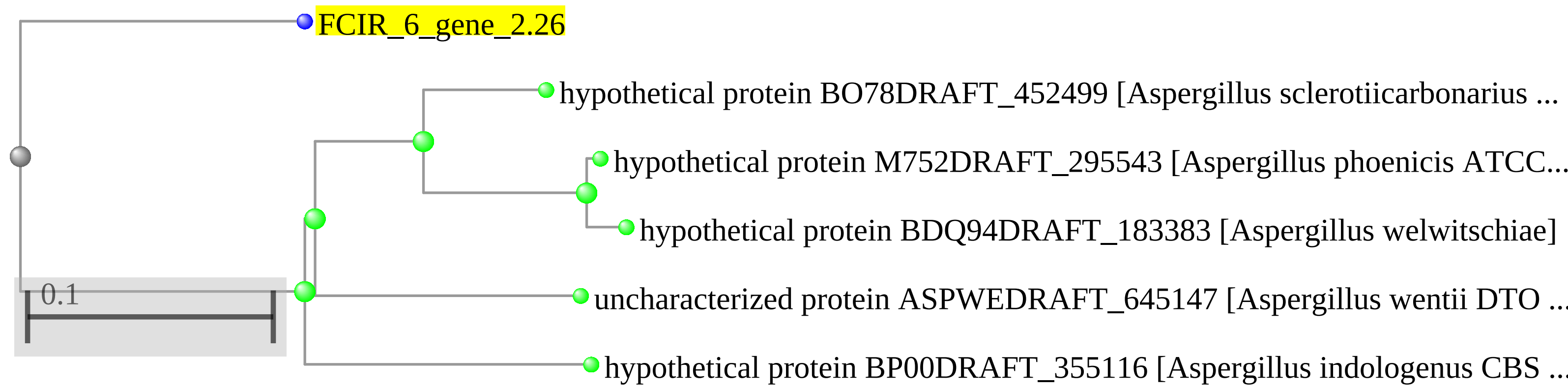
FCIR_6_gene_2.26
hypothetical protein BO78DRAFT_452499 [Aspergillus sclerotiicarbonarius ...
hypothetical protein M752DRAFT_295543 [Aspergillus phoenicis ATCC...
hypothetical protein BDQ94DRAFT_183383 [Aspergillus welwitschiae]
uncharacterized protein ASPWEDRAFT_645147 [Aspergillus wentii DTO ...
hypothetical protein BP00DRAFT_355116 [Aspergillus indologenus CBS ...
0.1

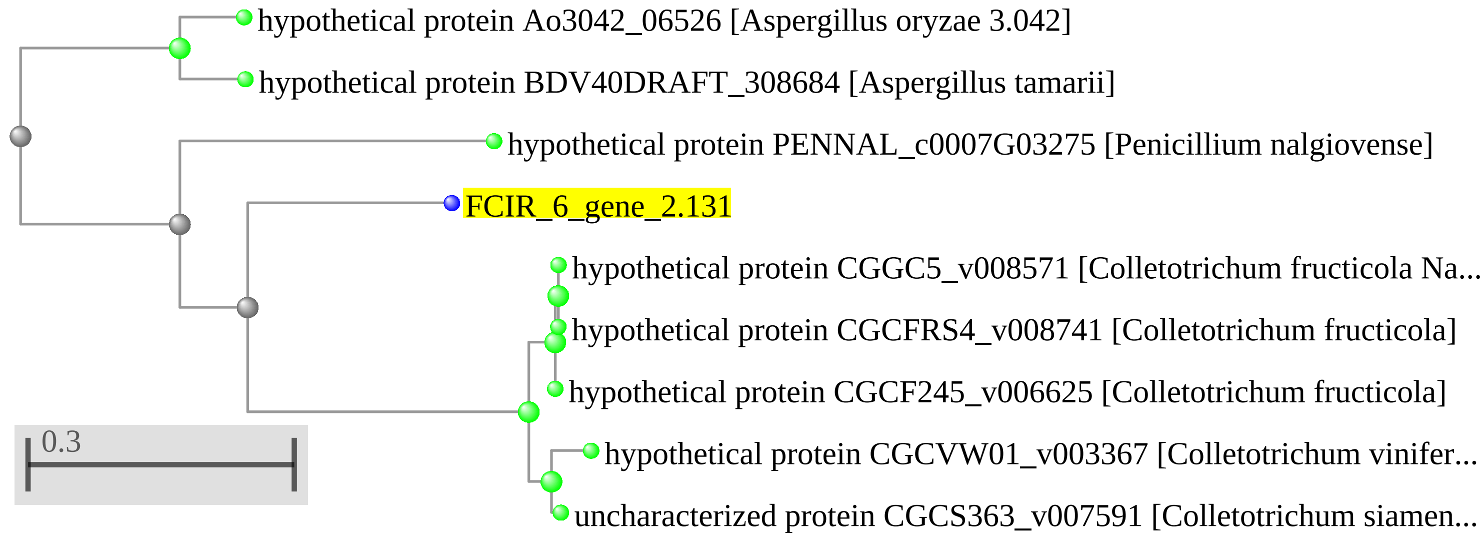

hypothetical protein Ao3042_06526 [Aspergillus oryzae 3.042]
hypothetical protein BDV40DRAFT_308684 [Aspergillus tamarii]
hypothetical protein PENNAL_c0007G03275 [Penicillium nalgiovense]
FCIR_6_gene_2.131
hypothetical protein CGGC5_v008571 [Colletotrichum fructicola Na...
hypothetical protein CGCFRS4_v008741 [Colletotrichum fructicola]
hypothetical protein CGCF245_v006625 [Colletotrichum fructicola]
hypothetical protein CGCVW01_v003367 [Colletotrichum vinifer...
uncharacterized protein CGCS363_v007591 [Colletotrichum siamen...
0.3

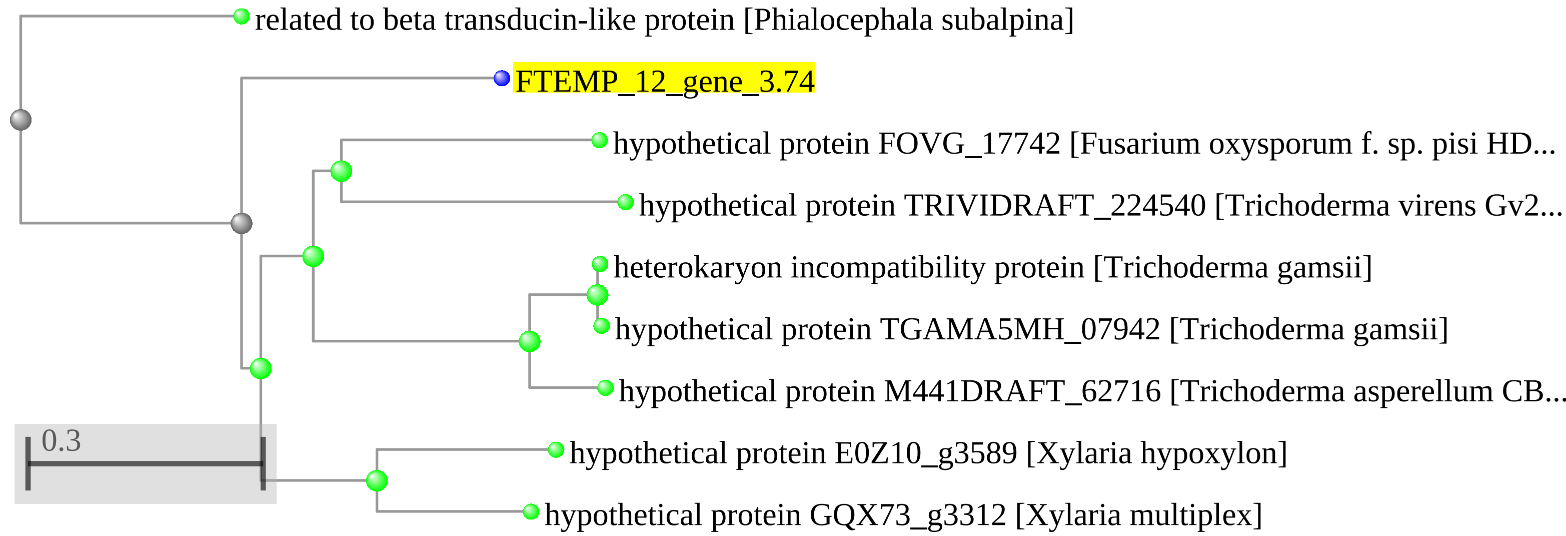
related to beta transducin-like protein [Phialocephala subalpina]
FTEMP_12_gene_3.74
hypothetical protein FOVG_17742 [Fusarium oxysporum f. sp. pisi HD...
hypothetical protein TRIVIDRAFT_224540 [Trichoderma virens Gv2...
heterokaryon incompatibility protein [Trichoderma gamsii]
hypothetical protein TGAMA5MH_07942 [Trichoderma gamsii]
hypothetical protein M441DRAFT_62716 [Trichoderma asperellum CB...
hypothetical protein E0Z10_g3589 [Xylaria hypoxylon]
hypothetical protein GQX73_g3312 [Xylaria multiplex]
0.3
