# Supplementary material for: Characterization of Host-Specific Genes from Pine- and Grass-Associated Species of the Fusarium fujikuroi Species Complex
Source: Pathogens. 2022 Jul 29;11(8):858. doi: 10.3390/pathogens11080858 (PMC9415769; doi:10.3390/pathogens11080858)
Supplement: Supplementary file 1 [file pathogens-11-00858-s001.zip › Supplemental Figures/Figure S7.pdf]

Figure S7. Host-range-associated genes with ancestral origins outside *Fusarium* but in the Ascomycetes. Host-range-associated genes investigated are highlighted in yellow; FCIR = *Fusarium circinatum* and FTEMP = *Fusarium temperatum.*

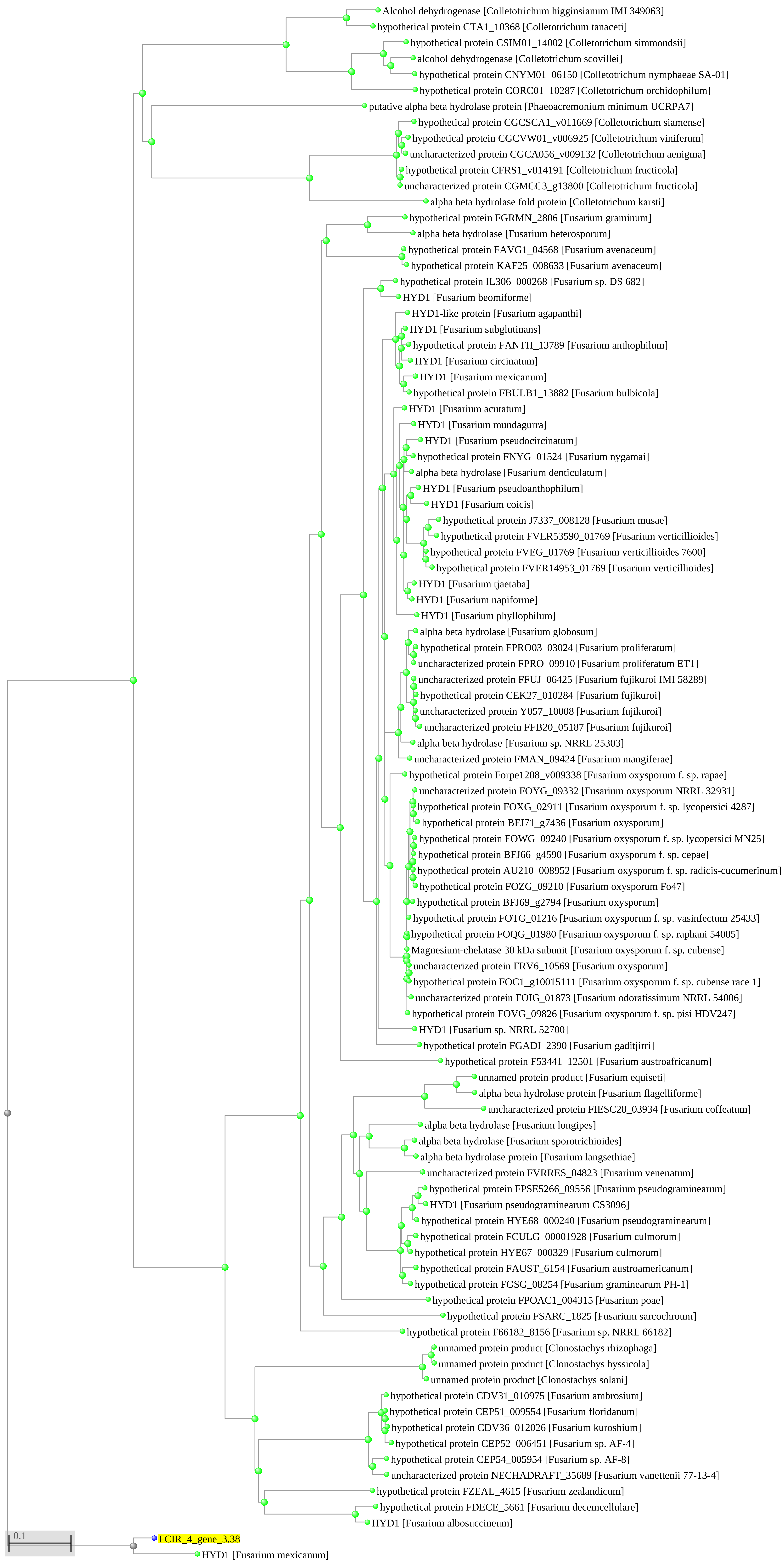
Alcohol dehydrogenase [Colletotrichum higginsianum IMI 349063]
hypothetical protein CTA1_10368 [Colletotrichum tanaceti]
hypothetical protein CSIM01_14002 [Colletotrichum simmondsii]
alcohol dehydrogenase [Colletotrichum scovillei]
hypothetical protein CNYM01_06150 [Colletotrichum nymphaeae SA-01]
hypothetical protein CORC01_10287 [Colletotrichum orchidophilum]
putative alpha beta hydrolase protein [Phaeoacremonium minimum UCRPA7]
hypothetical protein CGCSCA1_v011669 [Colletotrichum siamense]
hypothetical protein CGCVW01_v006925 [Colletotrichum viniferum]
uncharacterized protein CGCA056_v009132 [Colletotrichum aenigma]
hypothetical protein CFRS1_v014191 [Colletotrichum fructicola]
uncharacterized protein CGMCC3_g13800 [Colletotrichum fructicola]
alpha beta hydrolase fold protein [Colletotrichum karsti]
hypothetical protein FGRMN_2806 [Fusarium graminum]
alpha beta hydrolase [Fusarium heterosporum]
hypothetical protein FAVG1_04568 [Fusarium avenaceum]
hypothetical protein KAF25_008633 [Fusarium avenaceum]
hypothetical protein IL306_000268 [Fusarium sp. DS 682]
HYD1 [Fusarium beomiforme]
HYD1-like protein [Fusarium agapanthi]
HYD1 [Fusarium subglutinans]
hypothetical protein FANTH_13789 [Fusarium anthophilum]
HYD1 [Fusarium circinatum]
HYD1 [Fusarium mexicanum]
hypothetical protein FBULB1_13882 [Fusarium bulbicola]
HYD1 [Fusarium acutatum]
HYD1 [Fusarium mundagurra]
HYD1 [Fusarium pseudocircinatum]
hypothetical protein FNYG_01524 [Fusarium nygamai]
alpha beta hydrolase [Fusarium denticulatum]
HYD1 [Fusarium pseudoanthophilum]
HYD1 [Fusarium coicis]
hypothetical protein J7337_008128 [Fusarium musae]
hypothetical protein FVER53590_01769 [Fusarium verticillioides]
hypothetical protein FVEG_01769 [Fusarium verticillioides 7600]
hypothetical protein FVER14953_01769 [Fusarium verticillioides]
HYD1 [Fusarium tjaetaba]
HYD1 [Fusarium napiforme]
HYD1 [Fusarium phyllophilum]
alpha beta hydrolase [Fusarium globosum]
hypothetical protein FPRO03_03024 [Fusarium proliferatum]
uncharacterized protein FPRO_09910 [Fusarium proliferatum ET1]
uncharacterized protein FFUJ_06425 [Fusarium fujikuroi IMI 58289]
hypothetical protein CEK27_010284 [Fusarium fujikuroi]
uncharacterized protein Y057_10008 [Fusarium fujikuroi]
uncharacterized protein FFB20_05187 [Fusarium fujikuroi]
alpha beta hydrolase [Fusarium sp. NRRL 25303]
uncharacterized protein FMAN_09424 [Fusarium mangiferae]
hypothetical protein Forpe1208_v009338 [Fusarium oxysporum f. sp. rapae]
uncharacterized protein FOYG_09332 [Fusarium oxysporum NRRL 32931]
hypothetical protein FOXG_02911 [Fusarium oxysporum f. sp. lycopersici 4287]
hypothetical protein BFJ71_g7436 [Fusarium oxysporum]
hypothetical protein FOWG_09240 [Fusarium oxysporum f. sp. lycopersici MN25]
hypothetical protein BFJ66_g4590 [Fusarium oxysporum f. sp. cepae]
hypothetical protein AU210_008952 [Fusarium oxysporum f. sp. radicis-cucumerinum]
hypothetical protein FOZG_09210 [Fusarium oxysporum Fo47]
hypothetical protein BFJ69_g2794 [Fusarium oxysporum]
hypothetical protein FOTG_01216 [Fusarium oxysporum f. sp. vasinfectum 25433]
hypothetical protein FOQG_01980 [Fusarium oxysporum f. sp. raphani 54005]
Magnesium-chelatase 30 kDa subunit [Fusarium oxysporum f. sp. cubense]
uncharacterized protein FRV6_10569 [Fusarium oxysporum]
hypothetical protein FOC1_g10015111 [Fusarium oxysporum f. sp. cubense race 1]
uncharacterized protein FOIG_01873 [Fusarium odoratissimum NRRL 54006]
hypothetical protein FOVG_09826 [Fusarium oxysporum f. sp. pisi HDV247]
HYD1 [Fusarium sp. NRRL 52700]
hypothetical protein FGADI_2390 [Fusarium gaditjirri]
hypothetical protein F53441_12501 [Fusarium austroafricanum]
unnamed protein product [Fusarium equiseti]
alpha beta hydrolase protein [Fusarium flagelliforme]
uncharacterized protein FIESC28_03934 [Fusarium coffeatum]
alpha beta hydrolase [Fusarium longipes]
alpha beta hydrolase [Fusarium sporotrichioides]
alpha beta hydrolase protein [Fusarium langsethiae]
uncharacterized protein FVRRES_04823 [Fusarium venenatum]
hypothetical protein FPSE5266_09556 [Fusarium pseudograminearum]
HYD1 [Fusarium pseudograminearum CS3096]
hypothetical protein HYE68_000240 [Fusarium pseudograminearum]
hypothetical protein FCULG_00001928 [Fusarium culmorum]
hypothetical protein HYE67_000329 [Fusarium culmorum]
hypothetical protein FAUST_6154 [Fusarium austroamericanum]
hypothetical protein FGSG_08254 [Fusarium graminearum PH-1]
hypothetical protein FPOAC1_004315 [Fusarium poae]
hypothetical protein FSARC_1825 [Fusarium sarcochroum]
hypothetical protein F66182_8156 [Fusarium sp. NRRL 66182]
unnamed protein product [Clonostachys rhizophaga]
unnamed protein product [Clonostachys byssicola]
unnamed protein product [Clonostachys solani]
hypothetical protein CDV31_010975 [Fusarium ambrosium]
hypothetical protein CEP51_009554 [Fusarium floridanum]
hypothetical protein CDV36_012026 [Fusarium kuroshium]
hypothetical protein CEP52_006451 [Fusarium sp. AF-4]
hypothetical protein CEP54_005954 [Fusarium sp. AF-8]
uncharacterized protein NECHADRAFT_35689 [Fusarium vanettenii 77-13-4]
hypothetical protein FZEAL_4615 [Fusarium zealandicum]
hypothetical protein FDECE_5661 [Fusarium decemcellulare]
HYD1 [Fusarium albosuccineum]
0.1
FCIR_4_gene_3.38
HYD1 [Fusarium mexicanum]

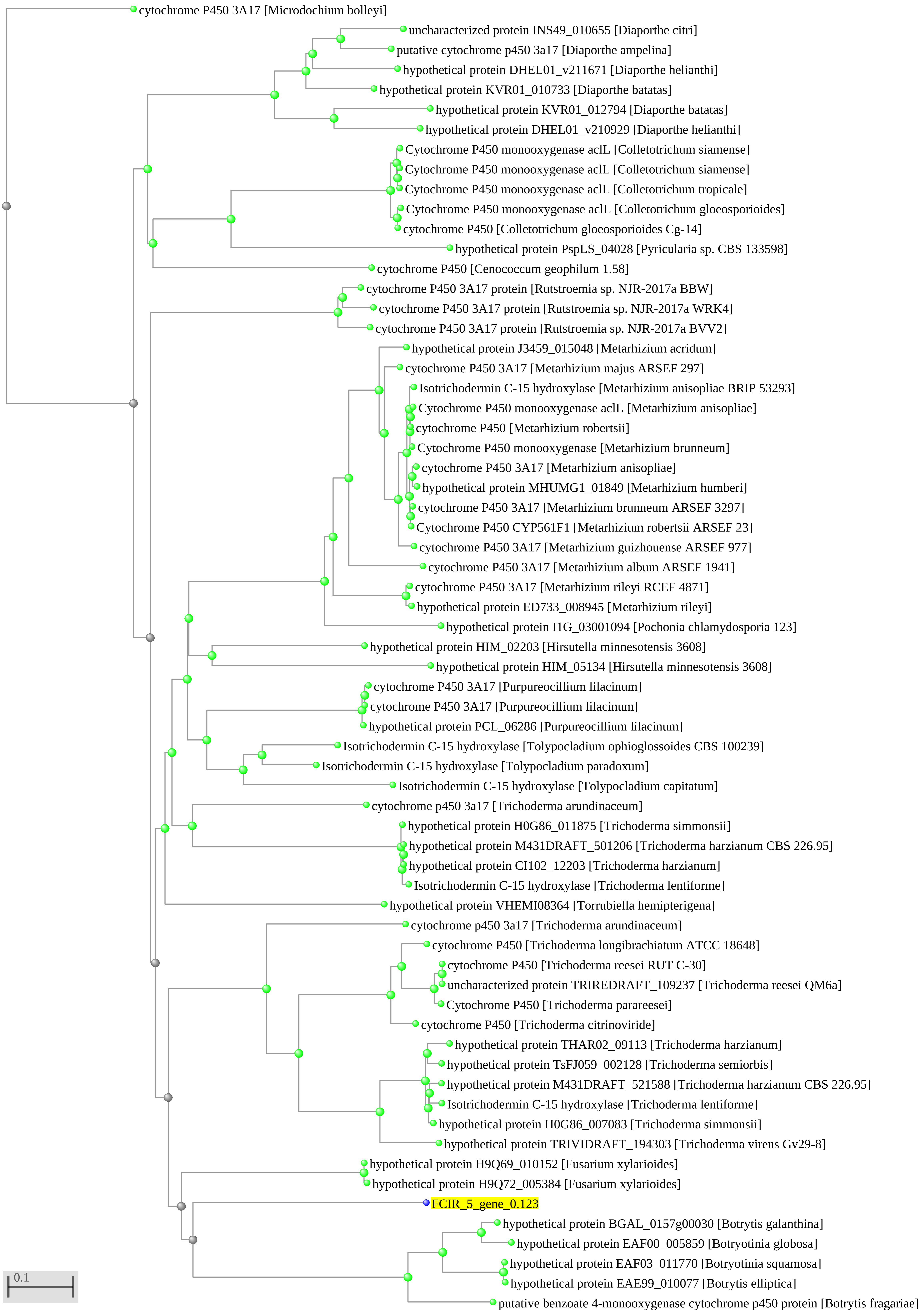

cytochrome P450 3A17 [Microdochium bolleyi]
uncharacterized protein INS49_010655 [Diaporthe citri]
putative cytochrome p450 3a17 [Diaporthe ampelina]
hypothetical protein DHEL01_v211671 [Diaporthe helianthi]
hypothetical protein KVR01_010733 [Diaporthe batatas]
hypothetical protein KVR01_012794 [Diaporthe batatas]
hypothetical protein DHEL01_v210929 [Diaporthe helianthi]
Cytochrome P450 monooxygenase aclL [Colletotrichum siamense]
Cytochrome P450 monooxygenase aclL [Colletotrichum siamense]
Cytochrome P450 monooxygenase aclL [Colletotrichum tropicale]
Cytochrome P450 monooxygenase aclL [Colletotrichum gloeosporioides]
cytochrome P450 [Colletotrichum gloeosporioides Cg-14]
hypothetical protein PspLS_04028 [Pyricularia sp. CBS 133598]
cytochrome P450 [Cenococcum geophilum 1.58]
cytochrome P450 3A17 protein [Rutstroemia sp. NJR-2017a BBW]
cytochrome P450 3A17 protein [Rutstroemia sp. NJR-2017a WRK4]
cytochrome P450 3A17 protein [Rutstroemia sp. NJR-2017a BVV2]
hypothetical protein J3459_015048 [Metarhizium acridum]
cytochrome P450 3A17 [Metarhizium majus ARSEF 297]
Isotrichodermin C-15 hydroxylase [Metarhizium anisopliae BRIP 53293]
Cytochrome P450 monooxygenase aclL [Metarhizium anisopliae]
cytochrome P450 [Metarhizium robertsii]
Cytochrome P450 monooxygenase [Metarhizium brunneum]
cytochrome P450 3A17 [Metarhizium anisopliae]
hypothetical protein MHUMG1_01849 [Metarhizium humberi]
cytochrome P450 3A17 [Metarhizium brunneum ARSEF 3297]
Cytochrome P450 CYP561F1 [Metarhizium robertsii ARSEF 23]
cytochrome P450 3A17 [Metarhizium guizhouense ARSEF 977]
cytochrome P450 3A17 [Metarhizium album ARSEF 1941]
cytochrome P450 3A17 [Metarhizium rileyi RCEF 4871]
hypothetical protein ED733_008945 [Metarhizium rileyi]
hypothetical protein I1G_03001094 [Pochonia chlamydosporia 123]
hypothetical protein HIM_02203 [Hirsutella minnesotensis 3608]
hypothetical protein HIM_05134 [Hirsutella minnesotensis 3608]
cytochrome P450 3A17 [Purpureocillium lilacinum]
cytochrome P450 3A17 [Purpureocillium lilacinum]
hypothetical protein PCL_06286 [Purpureocillium lilacinum]
Isotrichodermin C-15 hydroxylase [Tolypocladium ophioglossoides CBS 100239]
Isotrichodermin C-15 hydroxylase [Tolypocladium paradoxum]
Isotrichodermin C-15 hydroxylase [Tolypocladium capitatum]
cytochrome p450 3a17 [Trichoderma arundinaceum]
hypothetical protein H0G86_011875 [Trichoderma simmonsii]
hypothetical protein M431DRAFT_501206 [Trichoderma harzianum CBS 226.95]
hypothetical protein CI102_12203 [Trichoderma harzianum]
Isotrichodermin C-15 hydroxylase [Trichoderma lentiforme]
hypothetical protein VHEMI08364 [Torrubiella hemipterigena]
cytochrome p450 3a17 [Trichoderma arundinaceum]
cytochrome P450 [Trichoderma longibrachiatum ATCC 18648]
cytochrome P450 [Trichoderma reesei RUT C-30]
uncharacterized protein TRIREDRAFT_109237 [Trichoderma reesei QM6a]
Cytochrome P450 [Trichoderma parareesei]
cytochrome P450 [Trichoderma citrinoviride]
hypothetical protein THAR02_09113 [Trichoderma harzianum]
hypothetical protein TsFJ059_002128 [Trichoderma semiorbis]
hypothetical protein M431DRAFT_521588 [Trichoderma harzianum CBS 226.95]
Isotrichodermin C-15 hydroxylase [Trichoderma lentiforme]
hypothetical protein H0G86_007083 [Trichoderma simmonsii]
hypothetical protein TRIVIDRAFT_194303 [Trichoderma virens Gv29-8]
hypothetical protein H9Q69_010152 [Fusarium xylarioides]
hypothetical protein H9Q72_005384 [Fusarium xylarioides]
FCIR_5_gene_0.123
hypothetical protein BGAL_0157g00030 [Botrytis galanthina]
hypothetical protein EAF00_005859 [Botryotinia globosa]
hypothetical protein EAF03_011770 [Botryotinia squamosa]
hypothetical protein EAE99_010077 [Botrytis elliptica]
putative benzoate 4-monooxygenase cytochrome p450 protein [Botrytis fragariae]
0.1

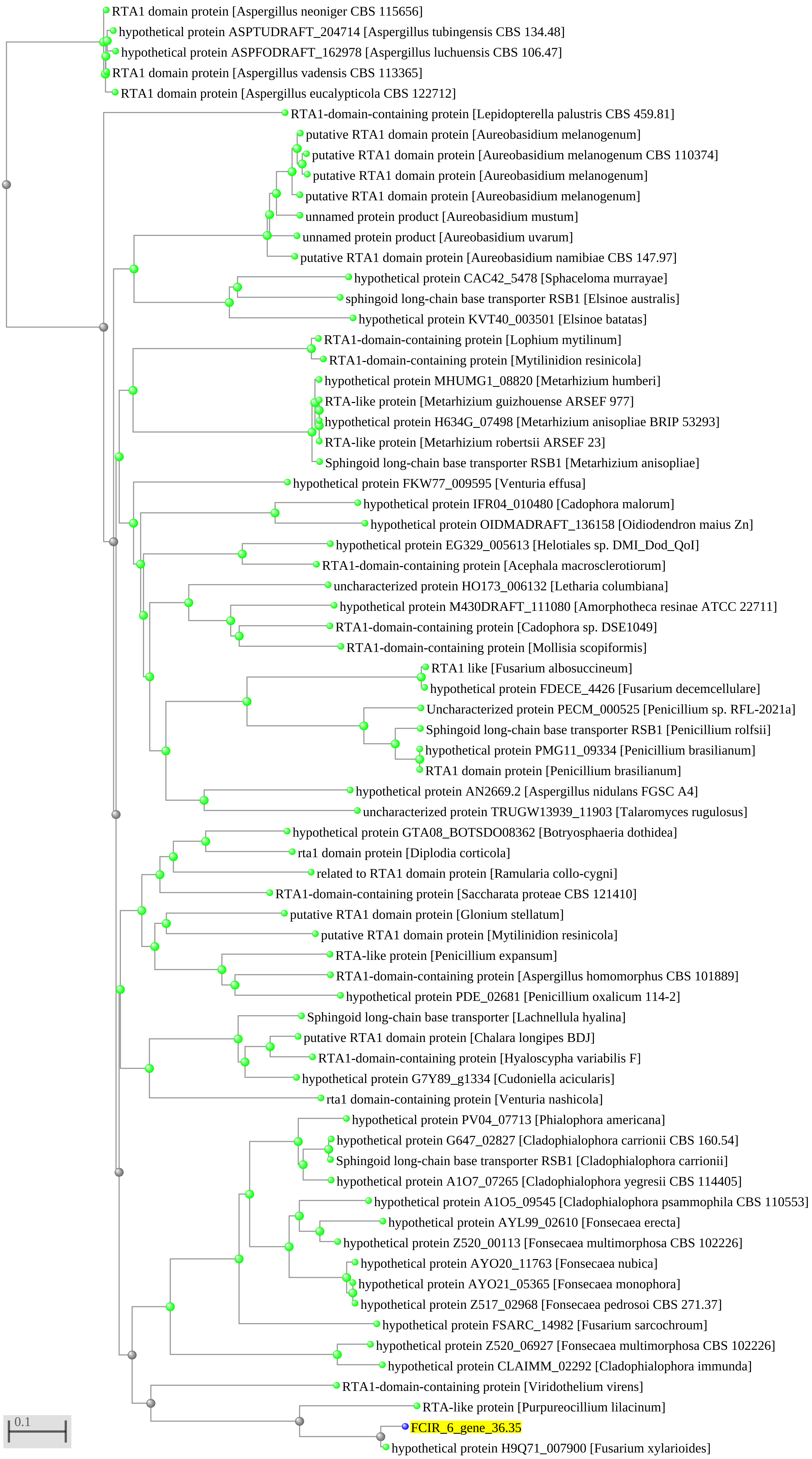
RTA1 domain protein [Aspergillus neoniger CBS 115656]
hypothetical protein ASPTUDRAFT_204714 [Aspergillus tubingensis CBS 134.48]
hypothetical protein ASPFODRAFT_162978 [Aspergillus luchuensis CBS 106.47]
RTA1 domain protein [Aspergillus vadensis CBS 113365]
RTA1 domain protein [Aspergillus eucalypticola CBS 122712]
RTA1-domain-containing protein [Lepidopterella palustris CBS 459.81]
putative RTA1 domain protein [Aureobasidium melanogenum]
putative RTA1 domain protein [Aureobasidium melanogenum CBS 110374]
putative RTA1 domain protein [Aureobasidium melanogenum]
putative RTA1 domain protein [Aureobasidium melanogenum]
unnamed protein product [Aureobasidium mustum]
unnamed protein product [Aureobasidium uvarum]
putative RTA1 domain protein [Aureobasidium namibiae CBS 147.97]
hypothetical protein CAC42_5478 [Sphaceloma murrayae]
sphingoid long-chain base transporter RSB1 [Elsinoe australis]
hypothetical protein KVT40_003501 [Elsinoe batatas]
RTA1-domain-containing protein [Lophium mytilinum]
RTA1-domain-containing protein [Mytilinidion resinicola]
hypothetical protein MHUMG1_08820 [Metarhizium humberi]
RTA-like protein [Metarhizium guizhouense ARSEF 977]
hypothetical protein H634G_07498 [Metarhizium anisopliae BRIP 53293]
RTA-like protein [Metarhizium robertsii ARSEF 23]
Sphingoid long-chain base transporter RSB1 [Metarhizium anisopliae]
hypothetical protein FKW77_009595 [Venturia effusa]
hypothetical protein IFR04_010480 [Cadophora malorum]
hypothetical protein OIDMADRAFT_136158 [Oidiodendron maius Zn]
hypothetical protein EG329_005613 [Helotiales sp. DMI_Dod_QoI]
RTA1-domain-containing protein [Acephala macrosclerotiorum]
uncharacterized protein HO173_006132 [Letharia columbiana]
hypothetical protein M430DRAFT_111080 [Amorphotheca resinae ATCC 22711]
RTA1-domain-containing protein [Cadophora sp. DSE1049]
RTA1-domain-containing protein [Mollisia scopiformis]
RTA1 like [Fusarium albosuccineum]
hypothetical protein FDECE_4426 [Fusarium decemcellulare]
Uncharacterized protein PECM_000525 [Penicillium sp. RFL-2021a]
Sphingoid long-chain base transporter RSB1 [Penicillium rolfsii]
hypothetical protein PMG11_09334 [Penicillium brasilianum]
RTA1 domain protein [Penicillium brasilianum]
hypothetical protein AN2669.2 [Aspergillus nidulans FGSC A4]
uncharacterized protein TRUGW13939_11903 [Talaromyces rugulosus]
hypothetical protein GTA08_BOTSDO08362 [Botryosphaeria dothidea]
rta1 domain protein [Diplodia corticola]
related to RTA1 domain protein [Ramularia collo-cygni]
RTA1-domain-containing protein [Saccharata proteae CBS 121410]
putative RTA1 domain protein [Glonium stellatum]
putative RTA1 domain protein [Mytilinidion resinicola]
RTA-like protein [Penicillium expansum]
RTA1-domain-containing protein [Aspergillus homomorphus CBS 101889]
hypothetical protein PDE_02681 [Penicillium oxalicum 114-2]
Sphingoid long-chain base transporter [Lachnellula hyalina]
putative RTA1 domain protein [Chalara longipes BDJ]
RTA1-domain-containing protein [Hyaloscypha variabilis F]
hypothetical protein G7Y89_g1334 [Cudoniella acicularis]
rta1 domain-containing protein [Venturia nashicola]
hypothetical protein PV04_07713 [Phialophora americana]
hypothetical protein G647_02827 [Cladophialophora carrionii CBS 160.54]
Sphingoid long-chain base transporter RSB1 [Cladophialophora carrionii]
hypothetical protein A1O7_07265 [Cladophialophora yegresii CBS 114405]
hypothetical protein A1O5_09545 [Cladophialophora psammophila CBS 110553]
hypothetical protein AYL99_02610 [Fonsecaea erecta]
hypothetical protein Z520_00113 [Fonsecaea multimorphosa CBS 102226]
hypothetical protein AYO20_11763 [Fonsecaea nubica]
hypothetical protein AYO21_05365 [Fonsecaea monophora]
hypothetical protein Z517_02968 [Fonsecaea pedrosoi CBS 271.37]
hypothetical protein FSARC_14982 [Fusarium sarcochroum]
hypothetical protein Z520_06927 [Fonsecaea multimorphosa CBS 102226]
hypothetical protein CLAIMM_02292 [Cladophialophora immunda]
RTA1-domain-containing protein [Viridothelium virens]
RTA-like protein [Purpureocillium lilacinum]
FCIR_6_gene_36.35
hypothetical protein H9Q71_007900 [Fusarium xylarioides]
0.1

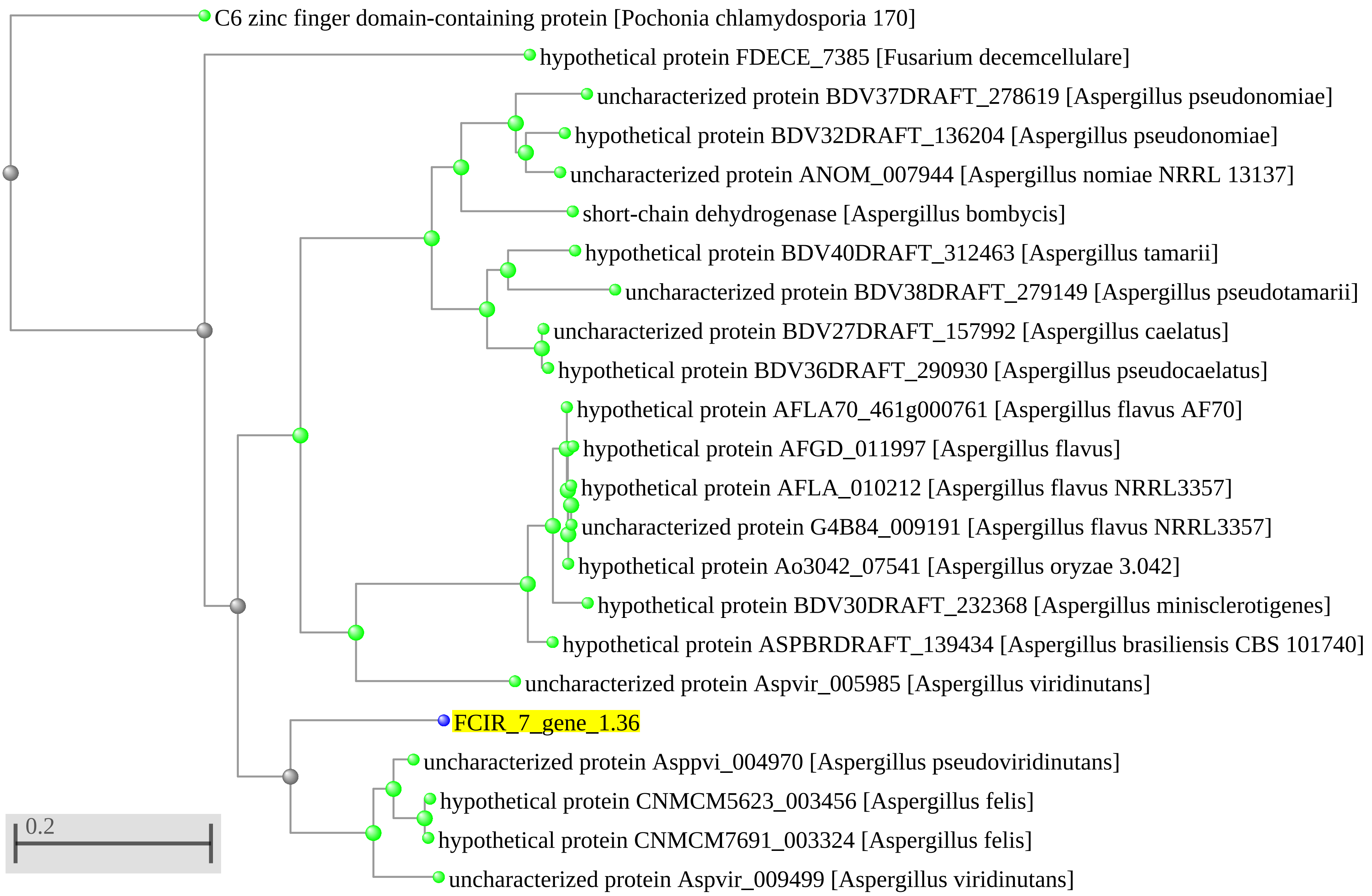
C6 zinc finger domain-containing protein [Pochonia chlamydosporia 170]
hypothetical protein FDECE_7385 [Fusarium decemcellulare]
uncharacterized protein BDV37DRAFT_278619 [Aspergillus pseudonomiae]
hypothetical protein BDV32DRAFT_136204 [Aspergillus pseudonomiae]
uncharacterized protein ANOM_007944 [Aspergillus nomiae NRRL 13137]
short-chain dehydrogenase [Aspergillus bombycis]
hypothetical protein BDV40DRAFT_312463 [Aspergillus tamarii]
uncharacterized protein BDV38DRAFT_279149 [Aspergillus pseudotamarii]
uncharacterized protein BDV27DRAFT_157992 [Aspergillus caelatus]
hypothetical protein BDV36DRAFT_290930 [Aspergillus pseudocaelatus]
hypothetical protein AFLA70_461g000761 [Aspergillus flavus AF70]
hypothetical protein AFGD_011997 [Aspergillus flavus]
hypothetical protein AFLA_010212 [Aspergillus flavus NRRL3357]
uncharacterized protein G4B84_009191 [Aspergillus flavus NRRL3357]
hypothetical protein Ao3042_07541 [Aspergillus oryzae 3.042]
hypothetical protein BDV30DRAFT_232368 [Aspergillus minisclerotigenes]
hypothetical protein ASPBRDRAFT_139434 [Aspergillus brasiliensis CBS 101740]
uncharacterized protein Aspvir_005985 [Aspergillus viridinutans]
FCIR_7_gene_1.36
uncharacterized protein Asppvi_004970 [Aspergillus pseudoviridinutans]
hypothetical protein CNMCM5623_003456 [Aspergillus felis]
hypothetical protein CNMCM7691_003324 [Aspergillus felis]
uncharacterized protein Aspvir_009499 [Aspergillus viridinutans]
0.2

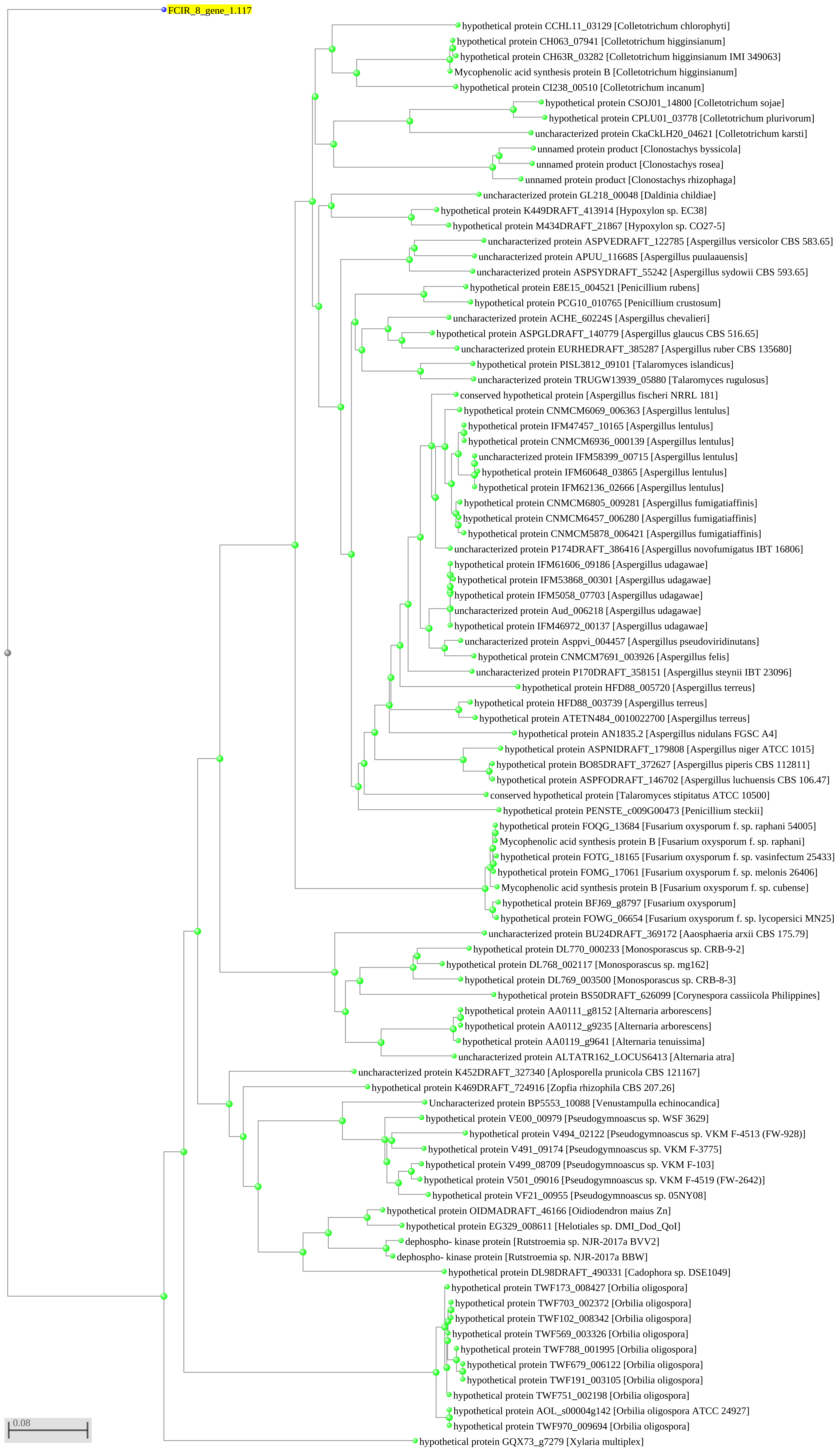
FCIR_8_gene_1.117
hypothetical protein CCHL11_03129 [Colletotrichum chlorophyti]
hypothetical protein CH063_07941 [Colletotrichum higginsianum]
hypothetical protein CH63R_03282 [Colletotrichum higginsianum IMI 349063]
Mycophenolic acid synthesis protein B [Colletotrichum higginsianum]
hypothetical protein CI238_00510 [Colletotrichum incanum]
hypothetical protein CSOJ01_14800 [Colletotrichum sojae]
hypothetical protein CPLU01_03778 [Colletotrichum plurivorum]
uncharacterized protein CkaCkLH20_04621 [Colletotrichum karsti]
unnamed protein product [Clonostachys byssicola]
unnamed protein product [Clonostachys rosea]
unnamed protein product [Clonostachys rhizophaga]
uncharacterized protein GL218_00048 [Daldinia childiae]
hypothetical protein K449DRAFT_413914 [Hypoxylon sp. EC38]
hypothetical protein M434DRAFT_21867 [Hypoxylon sp. CO27-5]
uncharacterized protein ASPVEDRAFT_122785 [Aspergillus versicolor CBS 583.65]
uncharacterized protein APUU_11668S [Aspergillus puulaauensis]
uncharacterized protein ASPSYDRAFT_55242 [Aspergillus sydowii CBS 593.65]
hypothetical protein E8E15_004521 [Penicillium rubens]
hypothetical protein PCG10_010765 [Penicillium crustosum]
uncharacterized protein ACHE_60224S [Aspergillus chevalieri]
hypothetical protein ASPGLDRAFT_140779 [Aspergillus glaucus CBS 516.65]
uncharacterized protein EURHEDRAFT_385287 [Aspergillus ruber CBS 135680]
hypothetical protein PISL3812_09101 [Talaromyces islandicus]
uncharacterized protein TRUGW13939_05880 [Talaromyces rugulosus]
conserved hypothetical protein [Aspergillus fischeri NRRL 181]
hypothetical protein CNMCM6069_006363 [Aspergillus lentulus]
hypothetical protein IFM47457_10165 [Aspergillus lentulus]
hypothetical protein CNMCM6936_000139 [Aspergillus lentulus]
uncharacterized protein IFM58399_00715 [Aspergillus lentulus]
hypothetical protein IFM60648_03865 [Aspergillus lentulus]
hypothetical protein IFM62136_02666 [Aspergillus lentulus]
hypothetical protein CNMCM6805_009281 [Aspergillus fumigatiaffinis]
hypothetical protein CNMCM6457_006280 [Aspergillus fumigatiaffinis]
hypothetical protein CNMCM5878_006421 [Aspergillus fumigatiaffinis]
uncharacterized protein P174DRAFT_386416 [Aspergillus novofumigatus IBT 16806]
hypothetical protein IFM61606_09186 [Aspergillus udagawae]
hypothetical protein IFM53868_00301 [Aspergillus udagawae]
hypothetical protein IFM5058_07703 [Aspergillus udagawae]
uncharacterized protein Aud_006218 [Aspergillus udagawae]
hypothetical protein IFM46972_00137 [Aspergillus udagawae]
uncharacterized protein Asppvi_004457 [Aspergillus pseudoviridinutans]
hypothetical protein CNMCM7691_003926 [Aspergillus felis]
uncharacterized protein P170DRAFT_358151 [Aspergillus steynii IBT 23096]
hypothetical protein HFD88_005720 [Aspergillus terreus]
hypothetical protein HFD88_003739 [Aspergillus terreus]
hypothetical protein ATETN484_0010022700 [Aspergillus terreus]
hypothetical protein AN1835.2 [Aspergillus nidulans FGSC A4]
hypothetical protein ASPNIDRAFT_179808 [Aspergillus niger ATCC 1015]
hypothetical protein BO85DRAFT_372627 [Aspergillus piperis CBS 112811]
hypothetical protein ASPFODRAFT_146702 [Aspergillus luchuensis CBS 106.47]
conserved hypothetical protein [Talaromyces stipitatus ATCC 10500]
hypothetical protein PENSTE_c009G00473 [Penicillium steckii]
hypothetical protein FOQG_13684 [Fusarium oxysporum f. sp. raphani 54005]
Mycophenolic acid synthesis protein B [Fusarium oxysporum f. sp. raphani]
hypothetical protein FOTG_18165 [Fusarium oxysporum f. sp. vasinfectum 25433]
hypothetical protein FOMG_17061 [Fusarium oxysporum f. sp. melonis 26406]
Mycophenolic acid synthesis protein B [Fusarium oxysporum f. sp. cubense]
hypothetical protein BFJ69_g8797 [Fusarium oxysporum]
hypothetical protein FOWG_06654 [Fusarium oxysporum f. sp. lycopersici MN25]
uncharacterized protein BU24DRAFT_369172 [Aaosphaeria arxii CBS 175.79]
hypothetical protein DL770_000233 [Monosporascus sp. CRB-9-2]
hypothetical protein DL768_002117 [Monosporascus sp. mg162]
hypothetical protein DL769_003500 [Monosporascus sp. CRB-8-3]
hypothetical protein BS50DRAFT_626099 [Corynespora cassiicola Philippines]
hypothetical protein AA0111_g8152 [Alternaria arborescens]
hypothetical protein AA0112_g9235 [Alternaria arborescens]
hypothetical protein AA0119_g9641 [Alternaria tenuissima]
uncharacterized protein ALTATR162_LOCUS6413 [Alternaria atra]
uncharacterized protein K452DRAFT_327340 [Aplosporella prunicola CBS 121167]
hypothetical protein K469DRAFT_724916 [Zopfia rhizophila CBS 207.26]
Uncharacterized protein BP5553_10088 [Venustampulla echinocandica]
hypothetical protein VE00_00979 [Pseudogymnoascus sp. WSF 3629]
hypothetical protein V494_02122 [Pseudogymnoascus sp. VKM F-4513 (FW-928)]
hypothetical protein V491_09174 [Pseudogymnoascus sp. VKM F-3775]
hypothetical protein V499_08709 [Pseudogymnoascus sp. VKM F-103]
hypothetical protein V501_09016 [Pseudogymnoascus sp. VKM F-4519 (FW-2642)]
hypothetical protein VF21_00955 [Pseudogymnoascus sp. 05NY08]
hypothetical protein OIDMADRAFT_46166 [Oidiodendron maius Zn]
hypothetical protein EG329_008611 [Helotiales sp. DMI_Dod_QoI]
dephospho- kinase protein [Rutstroemia sp. NJR-2017a BVV2]
dephospho- kinase protein [Rutstroemia sp. NJR-2017a BBW]
hypothetical protein DL98DRAFT_490331 [Cadophora sp. DSE1049]
hypothetical protein TWF173_008427 [Orbilia oligospora]
hypothetical protein TWF703_002372 [Orbilia oligospora]
hypothetical protein TWF102_008342 [Orbilia oligospora]
hypothetical protein TWF569_003326 [Orbilia oligospora]
hypothetical protein TWF788_001995 [Orbilia oligospora]
hypothetical protein TWF679_006122 [Orbilia oligospora]
hypothetical protein TWF191_003105 [Orbilia oligospora]
hypothetical protein TWF751_002198 [Orbilia oligospora]
hypothetical protein AOL_s00004g142 [Orbilia oligospora ATCC 24927]
hypothetical protein TWF970_009694 [Orbilia oligospora]
hypothetical protein GQX73_g7279 [Xylaria multiplex]
0.08

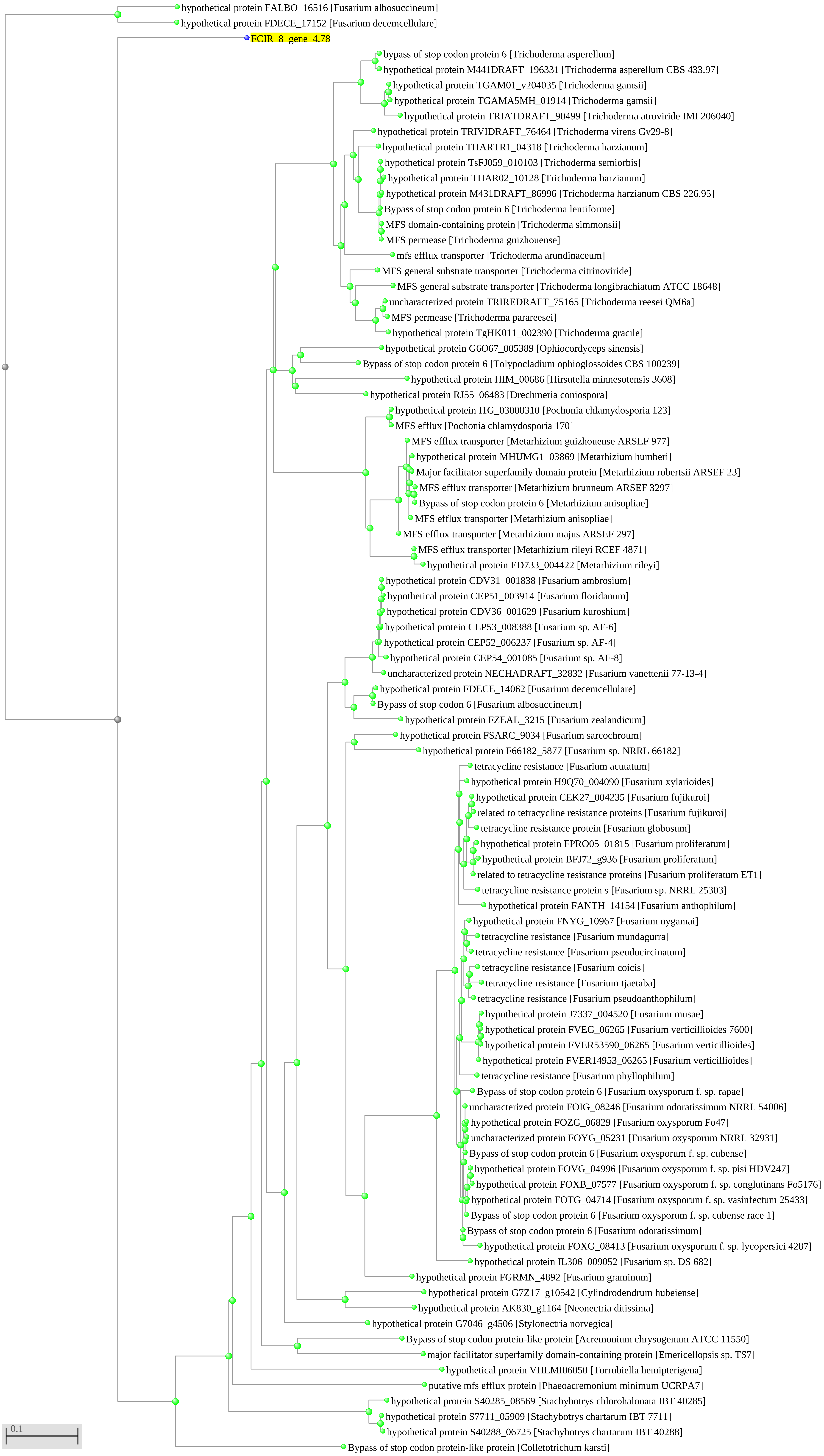

hypothetical protein FALBO_16516 [Fusarium albosuccineum]
hypothetical protein FDECE_17152 [Fusarium decemcellulare]
FCIR_8_gene_4.78
bypass of stop codon protein 6 [Trichoderma asperellum]
hypothetical protein M441DRAFT_196331 [Trichoderma asperellum CBS 433.97]
hypothetical protein TGAM01_v204035 [Trichoderma gamsii]
hypothetical protein TGAMA5MH_01914 [Trichoderma gamsii]
hypothetical protein TRIATDRAFT_90499 [Trichoderma atroviride IMI 206040]
hypothetical protein TRIVIDRAFT_76464 [Trichoderma virens Gv29-8]
hypothetical protein THARTR1_04318 [Trichoderma harzianum]
hypothetical protein TsFJ059_010103 [Trichoderma semiorbis]
hypothetical protein THAR02_10128 [Trichoderma harzianum]
hypothetical protein M431DRAFT_86996 [Trichoderma harzianum CBS 226.95]
Bypass of stop codon protein 6 [Trichoderma lentiforme]
MFS domain-containing protein [Trichoderma simmonsii]
MFS permease [Trichoderma guizhouense]
mfs efflux transporter [Trichoderma arundinaceum]
MFS general substrate transporter [Trichoderma citrinoviride]
MFS general substrate transporter [Trichoderma longibrachiatum ATCC 18648]
uncharacterized protein TRIREDRAFT_75165 [Trichoderma reesei QM6a]
MFS permease [Trichoderma parareesei]
hypothetical protein TgHK011_002390 [Trichoderma gracile]
hypothetical protein G6O67_005389 [Ophiocordyceps sinensis]
Bypass of stop codon protein 6 [Tolypocladium ophioglossoides CBS 100239]
hypothetical protein HIM_00686 [Hirsutella minnesotensis 3608]
hypothetical protein RJ55_06483 [Drechmeria coniospora]
hypothetical protein I1G_03008310 [Pochonia chlamydosporia 123]
MFS efflux [Pochonia chlamydosporia 170]
MFS efflux transporter [Metarhizium guizhouense ARSEF 977]
hypothetical protein MHUMG1_03869 [Metarhizium humberi]
Major facilitator superfamily domain protein [Metarhizium robertsii ARSEF 23]
MFS efflux transporter [Metarhizium brunneum ARSEF 3297]
Bypass of stop codon protein 6 [Metarhizium anisopliae]
MFS efflux transporter [Metarhizium anisopliae]
MFS efflux transporter [Metarhizium majus ARSEF 297]
MFS efflux transporter [Metarhizium rileyi RCEF 4871]
hypothetical protein ED733_004422 [Metarhizium rileyi]
hypothetical protein CDV31_001838 [Fusarium ambrosium]
hypothetical protein CEP51_003914 [Fusarium floridanum]
hypothetical protein CDV36_001629 [Fusarium kuroshium]
hypothetical protein CEP53_008388 [Fusarium sp. AF-6]
hypothetical protein CEP52_006237 [Fusarium sp. AF-4]
hypothetical protein CEP54_001085 [Fusarium sp. AF-8]
uncharacterized protein NECHADRAFT_32832 [Fusarium vanettenii 77-13-4]
hypothetical protein FDECE_14062 [Fusarium decemcellulare]
Bypass of stop codon 6 [Fusarium albosuccineum]
hypothetical protein FZEAL_3215 [Fusarium zealandicum]
hypothetical protein FSARC_9034 [Fusarium sarcochroum]
hypothetical protein F66182_5877 [Fusarium sp. NRRL 66182]
tetracycline resistance [Fusarium acutatum]
hypothetical protein H9Q70_004090 [Fusarium xylarioides]
hypothetical protein CEK27_004235 [Fusarium fujikuroi]
related to tetracycline resistance proteins [Fusarium fujikuroi]
tetracycline resistance protein [Fusarium globosum]
hypothetical protein FPRO05_01815 [Fusarium proliferatum]
hypothetical protein BFJ72_g936 [Fusarium proliferatum]
related to tetracycline resistance proteins [Fusarium proliferatum ET1]
tetracycline resistance protein s [Fusarium sp. NRRL 25303]
hypothetical protein FANTH_14154 [Fusarium anthophilum]
hypothetical protein FNYG_10967 [Fusarium nygamai]
tetracycline resistance [Fusarium mundagurra]
tetracycline resistance [Fusarium pseudocircinatum]
tetracycline resistance [Fusarium coicis]
tetracycline resistance [Fusarium tjaetaba]
tetracycline resistance [Fusarium pseudoanthophilum]
hypothetical protein J7337_004520 [Fusarium musae]
hypothetical protein FVEG_06265 [Fusarium verticillioides 7600]
hypothetical protein FVER53590_06265 [Fusarium verticillioides]
hypothetical protein FVER14953_06265 [Fusarium verticillioides]
tetracycline resistance [Fusarium phyllophilum]
Bypass of stop codon protein 6 [Fusarium oxysporum f. sp. rapae]
uncharacterized protein FOIG_08246 [Fusarium odoratissimum NRRL 54006]
hypothetical protein FOZG_06829 [Fusarium oxysporum Fo47]
uncharacterized protein FOYG_05231 [Fusarium oxysporum NRRL 32931]
Bypass of stop codon protein 6 [Fusarium oxysporum f. sp. cubense]
hypothetical protein FOVG_04996 [Fusarium oxysporum f. sp. pisi HDV247]
hypothetical protein FOXB_07577 [Fusarium oxysporum f. sp. conglutinans Fo5176]
hypothetical protein FOTG_04714 [Fusarium oxysporum f. sp. vasinfectum 25433]
Bypass of stop codon protein 6 [Fusarium oxysporum f. sp. cubense race 1]
Bypass of stop codon protein 6 [Fusarium odoratissimum]
hypothetical protein FOXG_08413 [Fusarium oxysporum f. sp. lycopersici 4287]
hypothetical protein IL306_009052 [Fusarium sp. DS 682]
hypothetical protein FGRMN_4892 [Fusarium graminum]
hypothetical protein G7Z17_g10542 [Cylindrodendrum hubeiense]
hypothetical protein AK830_g1164 [Neonectria ditissima]
hypothetical protein G7046_g4506 [Stylonectria norvegica]
Bypass of stop codon protein-like protein [Acremonium chrysogenum ATCC 11550]
major facilitator superfamily domain-containing protein [Emericellopsis sp. TS7]
hypothetical protein VHEMI06050 [Torrubiella hemipterigena]
putative mfs efflux protein [Phaeoacremonium minimum UCRPA7]
hypothetical protein S40285_08569 [Stachybotrys chlorohalonata IBT 40285]
hypothetical protein S7711_05909 [Stachybotrys chartarum IBT 7711]
hypothetical protein S40288_06725 [Stachybotrys chartarum IBT 40288]
Bypass of stop codon protein-like protein [Colletotrichum karsti]
0.1

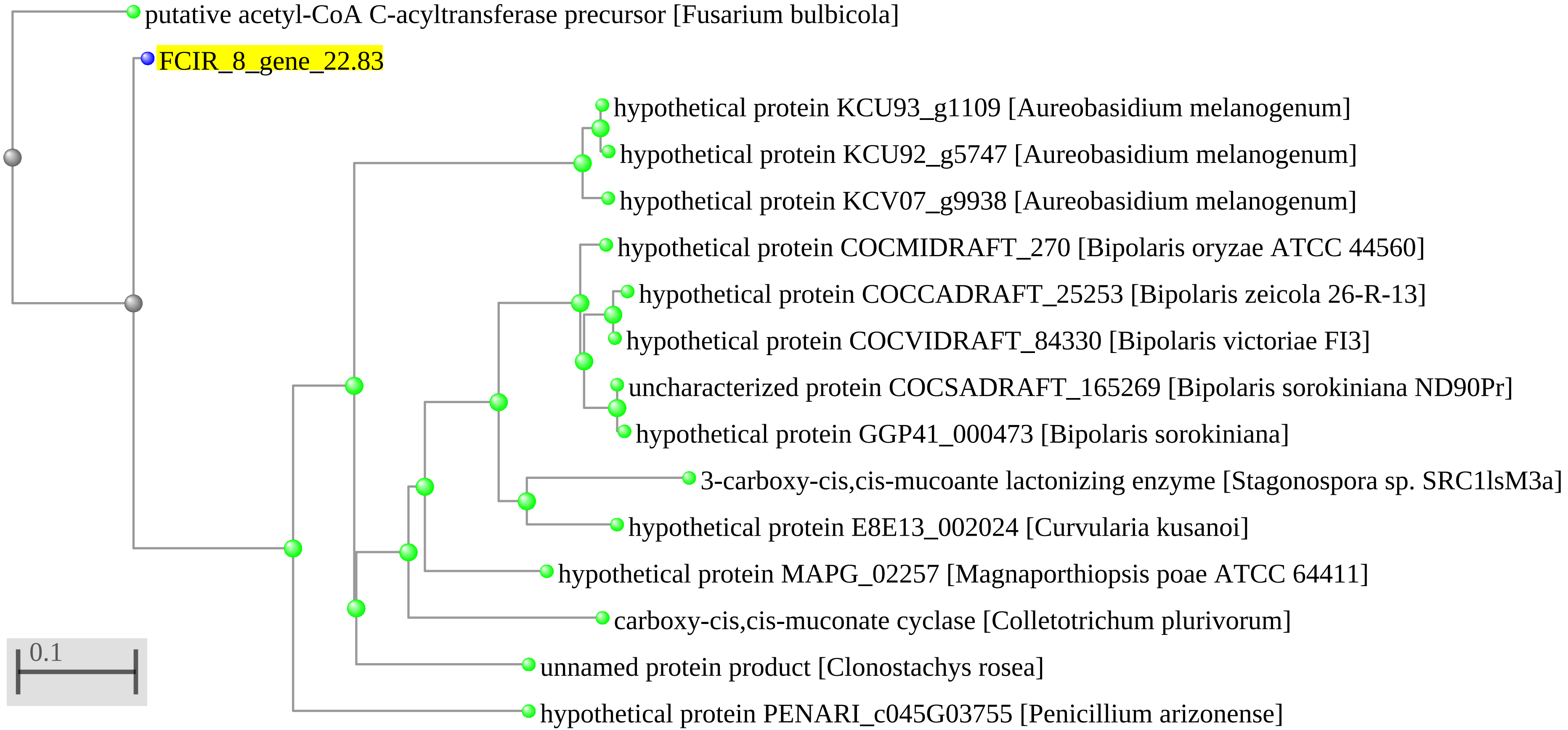
putative acetyl-CoA C-acyltransferase precursor [Fusarium bulbicola]
FCIR_8_gene_22.83
hypothetical protein KCU93_g1109 [Aureobasidium melanogenum]
hypothetical protein KCU92_g5747 [Aureobasidium melanogenum]
hypothetical protein KCV07_g9938 [Aureobasidium melanogenum]
hypothetical protein COCMIDRAFT_270 [Bipolaris oryzae ATCC 44560]
hypothetical protein COCCADRAFT_25253 [Bipolaris zeicola 26-R-13]
hypothetical protein COCVIDRAFT_84330 [Bipolaris victoriae FI3]
uncharacterized protein COCSADRAFT_165269 [Bipolaris sorokiniana ND90Pr]
hypothetical protein GGP41_000473 [Bipolaris sorokiniana]
3-carboxy-cis,cis-mucoante lactonizing enzyme [Stagonospora sp. SRC1lsM3a]
hypothetical protein E8E13_002024 [Curvularia kusanoi]
hypothetical protein MAPG_02257 [Magnaporthiopsis poae ATCC 64411]
carboxy-cis,cis-muconate cyclase [Colletotrichum plurivorum]
unnamed protein product [Clonostachys rosea]
hypothetical protein PENARI_c045G03755 [Penicillium arizonense]
0.1

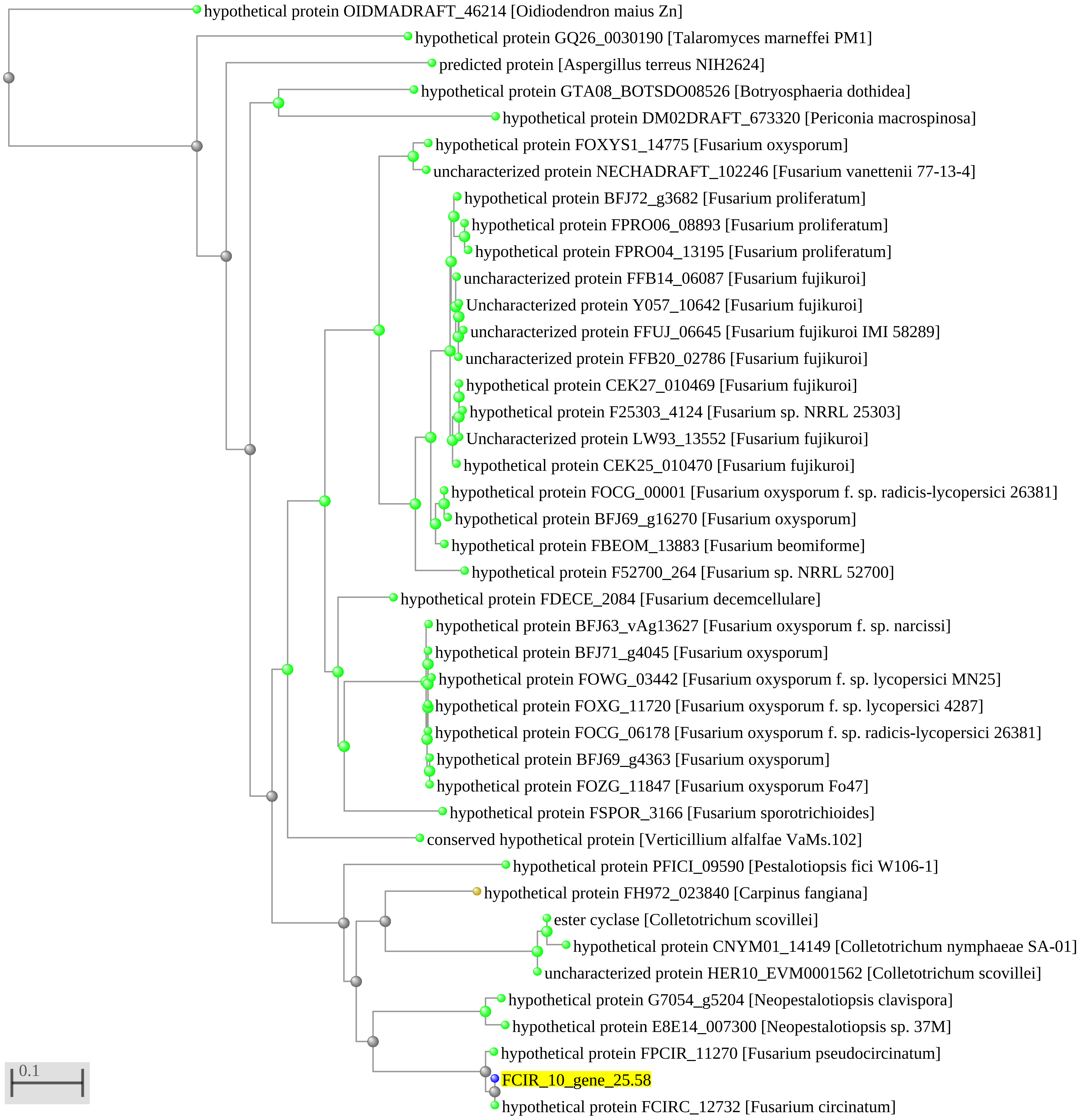
hypothetical protein OIDMADRAFT_46214 [Oidiodendron maius Zn]
hypothetical protein GQ26_0030190 [Talaromyces marneffei PM1]
predicted protein [Aspergillus terreus NIH2624]
hypothetical protein GTA08_BOTSDO08526 [Botryosphaeria dothidea]
hypothetical protein DM02DRAFT_673320 [Periconia macrospinosa]
hypothetical protein FOXYS1_14775 [Fusarium oxysporum]
uncharacterized protein NECHADRAFT_102246 [Fusarium vanettenii 77-13-4]
hypothetical protein BFJ72_g3682 [Fusarium proliferatum]
hypothetical protein FPRO06_08893 [Fusarium proliferatum]
hypothetical protein FPRO04_13195 [Fusarium proliferatum]
uncharacterized protein FFB14_06087 [Fusarium fujikuroi]
Uncharacterized protein Y057_10642 [Fusarium fujikuroi]
uncharacterized protein FFUJ_06645 [Fusarium fujikuroi IMI 58289]
uncharacterized protein FFB20_02786 [Fusarium fujikuroi]
hypothetical protein CEK27_010469 [Fusarium fujikuroi]
hypothetical protein F25303_4124 [Fusarium sp. NRRL 25303]
Uncharacterized protein LW93_13552 [Fusarium fujikuroi]
hypothetical protein CEK25_010470 [Fusarium fujikuroi]
hypothetical protein FOCG_00001 [Fusarium oxysporum f. sp. radicis-lycopersici 26381]
hypothetical protein BFJ69_g16270 [Fusarium oxysporum]
hypothetical protein FBEOM_13883 [Fusarium beomiforme]
hypothetical protein F52700_264 [Fusarium sp. NRRL 52700]
hypothetical protein FDECE_2084 [Fusarium decemcellulare]
hypothetical protein BFJ63_vAg13627 [Fusarium oxysporum f. sp. narcissi]
hypothetical protein BFJ71_g4045 [Fusarium oxysporum]
hypothetical protein FOWG_03442 [Fusarium oxysporum f. sp. lycopersici MN25]
hypothetical protein FOXG_11720 [Fusarium oxysporum f. sp. lycopersici 4287]
hypothetical protein FOCG_06178 [Fusarium oxysporum f. sp. radicis-lycopersici 26381]
hypothetical protein BFJ69_g4363 [Fusarium oxysporum]
hypothetical protein FOZG_11847 [Fusarium oxysporum Fo47]
hypothetical protein FSPOR_3166 [Fusarium sporotrichioides]
conserved hypothetical protein [Verticillium alfalfae VaMs.102]
hypothetical protein PFICI_09590 [Pestalotiopsis fici W106-1]
hypothetical protein FH972_023840 [Carpinus fangiana]
ester cyclase [Colletotrichum scovillei]
hypothetical protein CNYM01_14149 [Colletotrichum nymphaeae SA-01]
uncharacterized protein HER10_EVM0001562 [Colletotrichum scovillei]
hypothetical protein G7054_g5204 [Neopestalotiopsis clavispora]
hypothetical protein E8E14_007300 [Neopestalotiopsis sp. 37M]
hypothetical protein FPCIR_11270 [Fusarium pseudocircinatum]
FCIR_10_gene_25.58
hypothetical protein FCIRC_12732 [Fusarium circinatum]
0.1

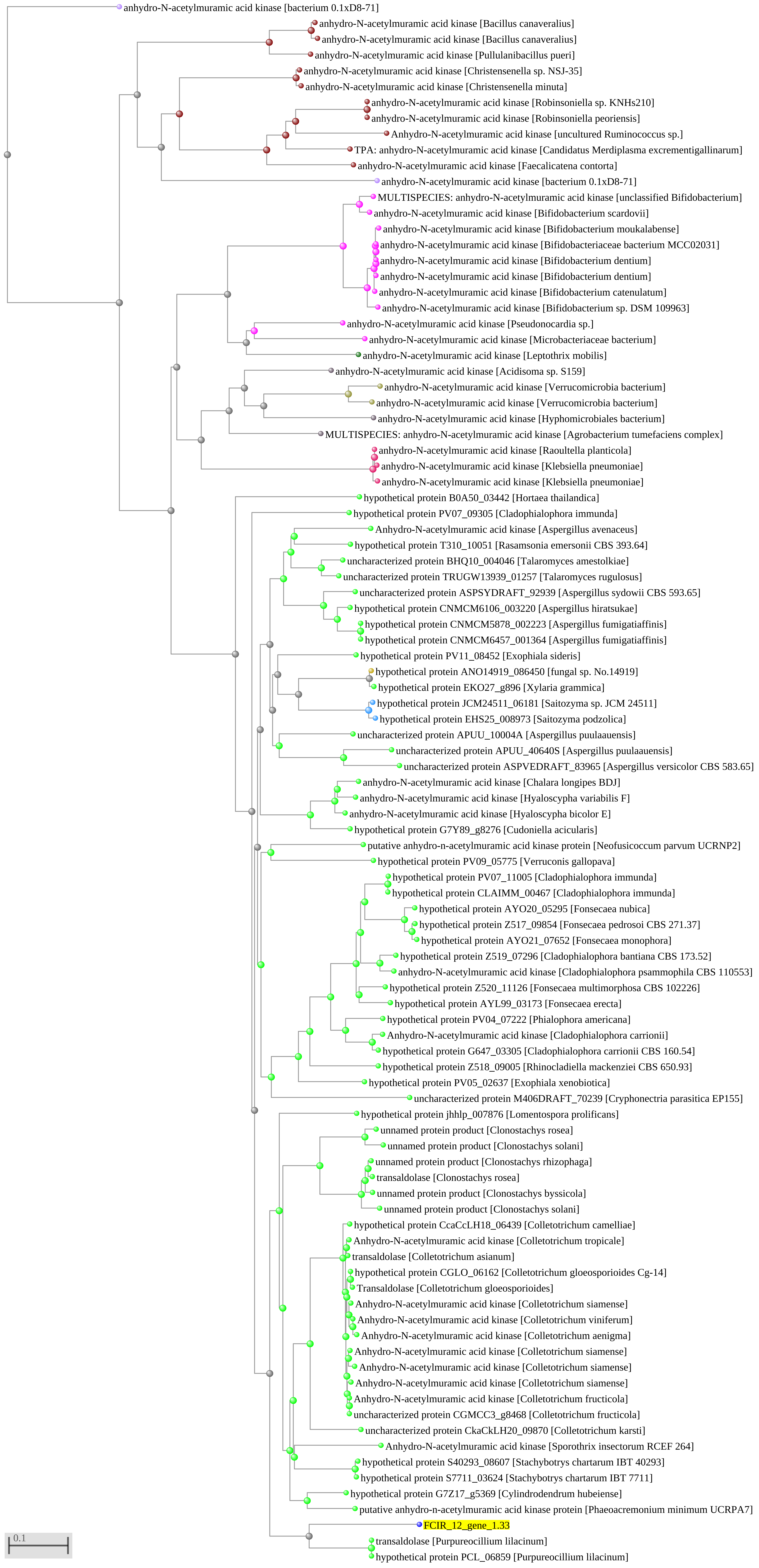
anhydro-N-acetylmuramic acid kinase [bacterium 0.1xD8-71]
anhydro-N-acetylmuramic acid kinase [Bacillus canaveralius]
anhydro-N-acetylmuramic acid kinase [Bacillus canaveralius]
anhydro-N-acetylmuramic acid kinase [Pullulanibacillus pueri]
anhydro-N-acetylmuramic acid kinase [Christensenella sp. NSJ-35]
anhydro-N-acetylmuramic acid kinase [Christensenella minuta]
anhydro-N-acetylmuramic acid kinase [Robinsoniella sp. KNHs210]
anhydro-N-acetylmuramic acid kinase [Robinsoniella peoriensis]
Anhydro-N-acetylmuramic acid kinase [uncultured Ruminococcus sp.]
TPA: anhydro-N-acetylmuramic acid kinase [Candidatus Merdiplasma excrementigallinarum]
anhydro-N-acetylmuramic acid kinase [Faecalicatena contorta]
anhydro-N-acetylmuramic acid kinase [bacterium 0.1xD8-71]
MULTISPECIES: anhydro-N-acetylmuramic acid kinase [unclassified Bifidobacterium]
anhydro-N-acetylmuramic acid kinase [Bifidobacterium scardovii]
anhydro-N-acetylmuramic acid kinase [Bifidobacterium moukalabense]
anhydro-N-acetylmuramic acid kinase [Bifidobacteriaceae bacterium MCC02031]
anhydro-N-acetylmuramic acid kinase [Bifidobacterium dentium]
anhydro-N-acetylmuramic acid kinase [Bifidobacterium dentium]
anhydro-N-acetylmuramic acid kinase [Bifidobacterium catenulatum]
anhydro-N-acetylmuramic acid kinase [Bifidobacterium sp. DSM 109963]
anhydro-N-acetylmuramic acid kinase [Pseudonocardia sp.]
anhydro-N-acetylmuramic acid kinase [Microbacteriaceae bacterium]
anhydro-N-acetylmuramic acid kinase [Leptothrix mobilis]
anhydro-N-acetylmuramic acid kinase [Acidisoma sp. S159]
anhydro-N-acetylmuramic acid kinase [Verrucomicrobia bacterium]
anhydro-N-acetylmuramic acid kinase [Verrucomicrobia bacterium]
anhydro-N-acetylmuramic acid kinase [Hyphomicrobiales bacterium]
MULTISPECIES: anhydro-N-acetylmuramic acid kinase [Agrobacterium tumefaciens complex]
anhydro-N-acetylmuramic acid kinase [Raoultella planticola]
anhydro-N-acetylmuramic acid kinase [Klebsiella pneumoniae]
anhydro-N-acetylmuramic acid kinase [Klebsiella pneumoniae]
hypothetical protein B0A50_03442 [Hortaea thailandica]
hypothetical protein PV07_09305 [Cladophialophora immunda]
Anhydro-N-acetylmuramic acid kinase [Aspergillus avenaceus]
hypothetical protein T310_10051 [Rasamsonia emersonii CBS 393.64]
uncharacterized protein BHQ10_004046 [Talaromyces amestolkiae]
uncharacterized protein TRUGW13939_01257 [Talaromyces rugulosus]
uncharacterized protein ASPSYDRAFT_92939 [Aspergillus sydowii CBS 593.65]
hypothetical protein CNMCM6106_003220 [Aspergillus hiratsukae]
hypothetical protein CNMCM5878_002223 [Aspergillus fumigatiaffinis]
hypothetical protein CNMCM6457_001364 [Aspergillus fumigatiaffinis]
hypothetical protein PV11_08452 [Exophiala sideris]
hypothetical protein ANO14919_086450 [fungal sp. No.14919]
hypothetical protein EKO27_g896 [Xylaria grammica]
hypothetical protein JCM24511_06181 [Saitozyma sp. JCM 24511]
hypothetical protein EHS25_008973 [Saitozyma podzolica]
uncharacterized protein APUU_10004A [Aspergillus puulaauensis]
uncharacterized protein APUU_40640S [Aspergillus puulaauensis]
uncharacterized protein ASPVEDRAFT_83965 [Aspergillus versicolor CBS 583.65]
anhydro-N-acetylmuramic acid kinase [Chalara longipes BDJ]
anhydro-N-acetylmuramic acid kinase [Hyaloscypha variabilis F]
anhydro-N-acetylmuramic acid kinase [Hyaloscypha bicolor E]
hypothetical protein G7Y89_g8276 [Cudoniella acicularis]
putative anhydro-n-acetylmuramic acid kinase protein [Neofusicoccum parvum UCRNP2]
hypothetical protein PV09_05775 [Verruconis gallopava]
hypothetical protein PV07_11005 [Cladophialophora immunda]
hypothetical protein CLAIMM_00467 [Cladophialophora immunda]
hypothetical protein AYO20_05295 [Fonsecaea nubica]
hypothetical protein Z517_09854 [Fonsecaea pedrosoi CBS 271.37]
hypothetical protein AYO21_07652 [Fonsecaea monophora]
hypothetical protein Z519_07296 [Cladophialophora bantiana CBS 173.52]
anhydro-N-acetylmuramic acid kinase [Cladophialophora psammophila CBS 110553]
hypothetical protein Z520_11126 [Fonsecaea multimorphosa CBS 102226]
hypothetical protein AYL99_03173 [Fonsecaea erecta]
hypothetical protein PV04_07222 [Phialophora americana]
Anhydro-N-acetylmuramic acid kinase [Cladophialophora carrionii]
hypothetical protein G647_03305 [Cladophialophora carrionii CBS 160.54]
hypothetical protein Z518_09005 [Rhinocladiella mackenziei CBS 650.93]
hypothetical protein PV05_02637 [Exophiala xenobiotica]
uncharacterized protein M406DRAFT_70239 [Cryphonectria parasitica EP155]
hypothetical protein jhhlp_007876 [Lomentospora prolificans]
unnamed protein product [Clonostachys rosea]
unnamed protein product [Clonostachys solani]
unnamed protein product [Clonostachys rhizophaga]
transaldolase [Clonostachys rosea]
unnamed protein product [Clonostachys byssicola]
unnamed protein product [Clonostachys solani]
hypothetical protein CcaCcLH18_06439 [Colletotrichum camelliae]
Anhydro-N-acetylmuramic acid kinase [Colletotrichum tropicale]
transaldolase [Colletotrichum asianum]
hypothetical protein CGLO_06162 [Colletotrichum gloeosporioides Cg-14]
Transaldolase [Colletotrichum gloeosporioides]
Anhydro-N-acetylmuramic acid kinase [Colletotrichum siamense]
Anhydro-N-acetylmuramic acid kinase [Colletotrichum viniferum]
Anhydro-N-acetylmuramic acid kinase [Colletotrichum aenigma]
Anhydro-N-acetylmuramic acid kinase [Colletotrichum siamense]
Anhydro-N-acetylmuramic acid kinase [Colletotrichum siamense]
Anhydro-N-acetylmuramic acid kinase [Colletotrichum siamense]
Anhydro-N-acetylmuramic acid kinase [Colletotrichum fructicola]
uncharacterized protein CGMCC3_g8468 [Colletotrichum fructicola]
uncharacterized protein CkaCkLH20_09870 [Colletotrichum karsti]
Anhydro-N-acetylmuramic acid kinase [Sporothrix insectorum RCEF 264]
hypothetical protein S40293_08607 [Stachybotrys chartarum IBT 40293]
hypothetical protein S7711_03624 [Stachybotrys chartarum IBT 7711]
hypothetical protein G7Z17_g5369 [Cylindrodendrum hubeiense]
putative anhydro-n-acetylmuramic acid kinase protein [Phaeoacremonium minimum UCRPA7]
FCIR_12_gene_1.33
transaldolase [Purpureocillium lilacinum]
hypothetical protein PCL_06859 [Purpureocillium lilacinum]
0.1

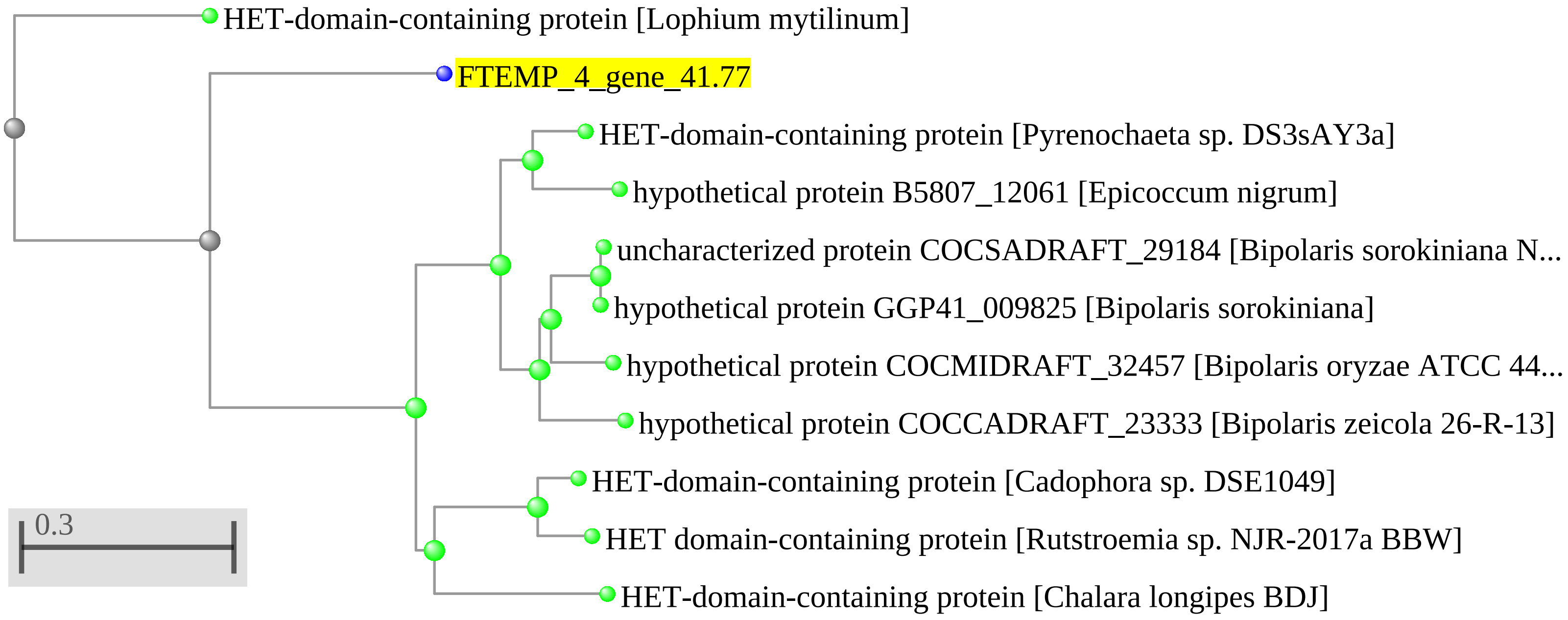

HET-domain-containing protein [Lophium mytilinum]
FTEMP_4_gene_41.77
HET-domain-containing protein [Pyrenochaeta sp. DS3sAY3a]
hypothetical protein B5807_12061 [Epicoccum nigrum]
uncharacterized protein COCSADRAFT_29184 [Bipolaris sorokiniana N...
hypothetical protein GGP41_009825 [Bipolaris sorokiniana]
hypothetical protein COCMIDRAFT_32457 [Bipolaris oryzae ATCC 44...
hypothetical protein COCCADRAFT_23333 [Bipolaris zeicola 26-R-13]
HET-domain-containing protein [Cadophora sp. DSE1049]
HET domain-containing protein [Rutstroemia sp. NJR-2017a BBW]
HET-domain-containing protein [Chalara longipes BDJ]
0.3

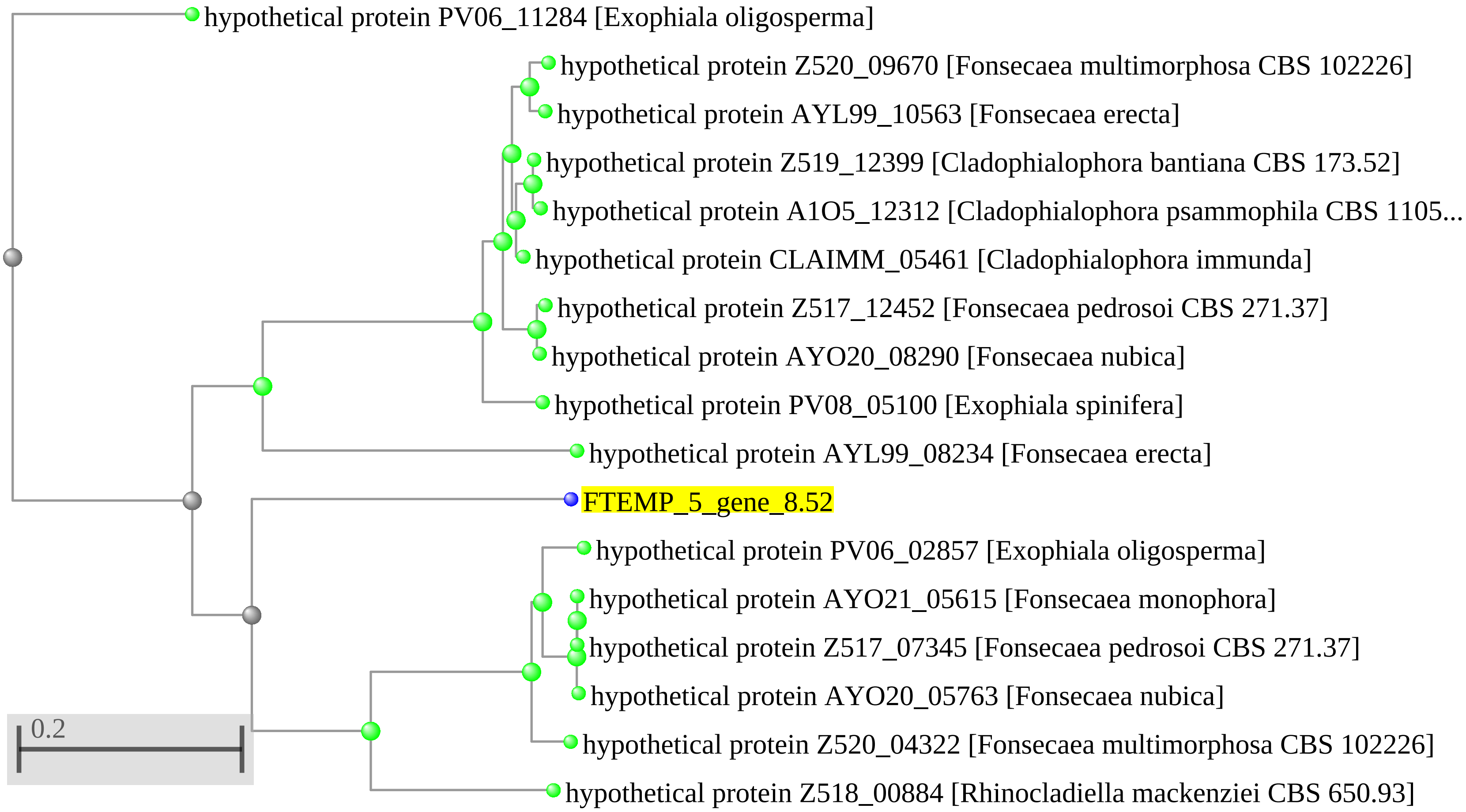
hypothetical protein PV06_11284 [Exophiala oligosperma]
hypothetical protein Z520_09670 [Fonsecaea multimorphosa CBS 102226]
hypothetical protein AYL99_10563 [Fonsecaea erecta]
hypothetical protein Z519_12399 [Cladophialophora bantiana CBS 173.52]
hypothetical protein A1O5_12312 [Cladophialophora psammophila CBS 1105...
hypothetical protein CLAIMM_05461 [Cladophialophora immunda]
hypothetical protein Z517_12452 [Fonsecaea pedrosoi CBS 271.37]
hypothetical protein AYO20_08290 [Fonsecaea nubica]
hypothetical protein PV08_05100 [Exophiala spinifera]
hypothetical protein AYL99_08234 [Fonsecaea erecta]
FTEMP_5_gene_8.52
hypothetical protein PV06_02857 [Exophiala oligosperma]
hypothetical protein AYO21_05615 [Fonsecaea monophora]
hypothetical protein Z517_07345 [Fonsecaea pedrosoi CBS 271.37]
hypothetical protein AYO20_05763 [Fonsecaea nubica]
hypothetical protein Z520_04322 [Fonsecaea multimorphosa CBS 102226]
hypothetical protein Z518_00884 [Rhinocladiella mackenziei CBS 650.93]
0.2

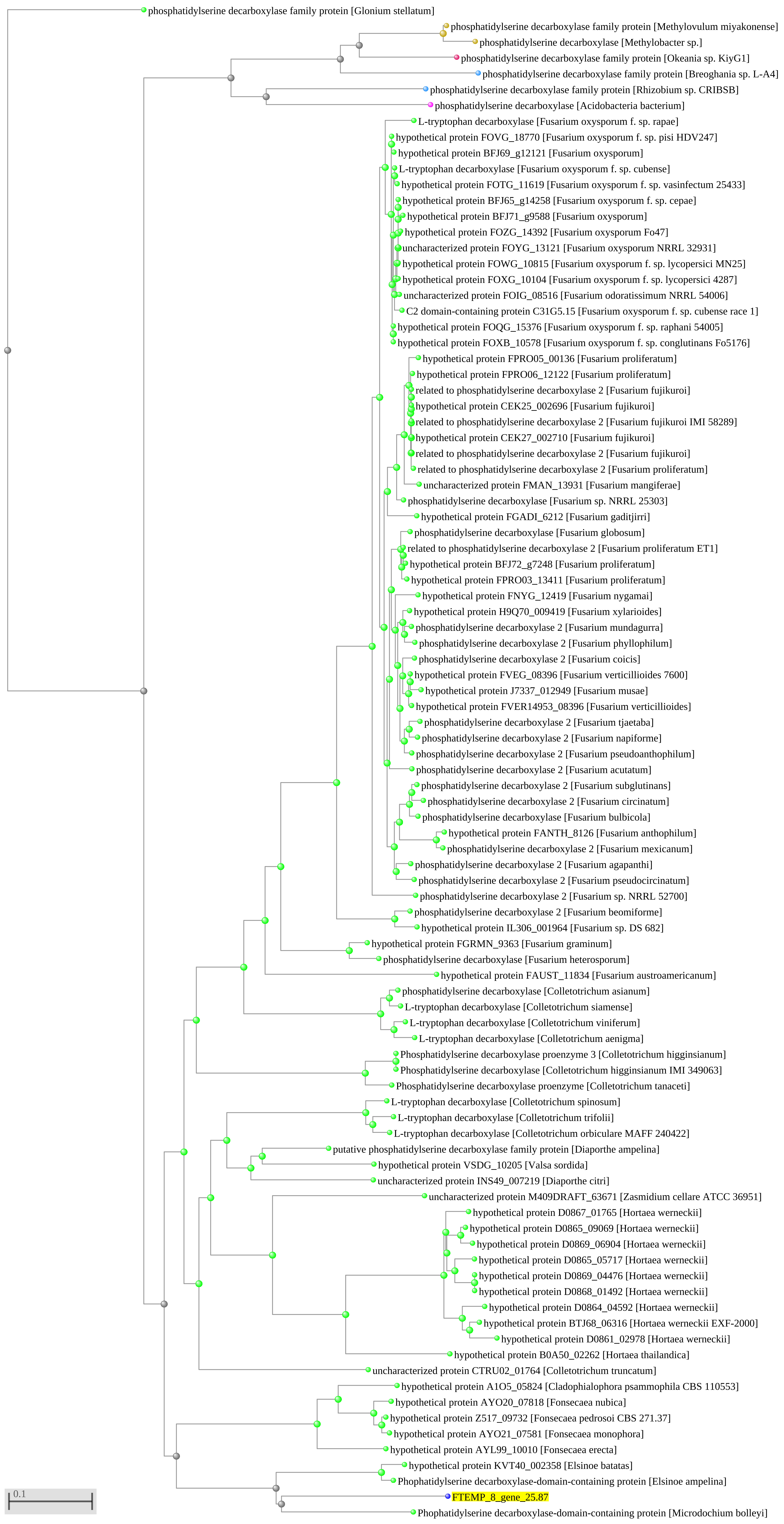
phosphatidylserine decarboxylase family protein [Glonium stellatum]
phosphatidylserine decarboxylase family protein [Methylovulum miyakonense]
phosphatidylserine decarboxylase [Methylobacter sp.]
phosphatidylserine decarboxylase family protein [Okeania sp. KiyG1]
phosphatidylserine decarboxylase family protein [Breoghania sp. L-A4]
phosphatidylserine decarboxylase family protein [Rhizobium sp. CRIBSB]
phosphatidylserine decarboxylase [Acidobacteria bacterium]
L-tryptophan decarboxylase [Fusarium oxysporum f. sp. rapae]
hypothetical protein FOVG_18770 [Fusarium oxysporum f. sp. pisi HDV247]
hypothetical protein BFJ69_g12121 [Fusarium oxysporum]
L-tryptophan decarboxylase [Fusarium oxysporum f. sp. cubense]
hypothetical protein FOTG_11619 [Fusarium oxysporum f. sp. vasinfectum 25433]
hypothetical protein BFJ65_g14258 [Fusarium oxysporum f. sp. cepae]
hypothetical protein BFJ71_g9588 [Fusarium oxysporum]
hypothetical protein FOZG_14392 [Fusarium oxysporum Fo47]
uncharacterized protein FOYG_13121 [Fusarium oxysporum NRRL 32931]
hypothetical protein FOWG_10815 [Fusarium oxysporum f. sp. lycopersici MN25]
hypothetical protein FOXG_10104 [Fusarium oxysporum f. sp. lycopersici 4287]
uncharacterized protein FOIG_08516 [Fusarium odoratissimum NRRL 54006]
C2 domain-containing protein C31G5.15 [Fusarium oxysporum f. sp. cubense race 1]
hypothetical protein FOQG_15376 [Fusarium oxysporum f. sp. raphani 54005]
hypothetical protein FOXB_10578 [Fusarium oxysporum f. sp. conglutinans Fo5176]
hypothetical protein FPRO05_00136 [Fusarium proliferatum]
hypothetical protein FPRO06_12122 [Fusarium proliferatum]
related to phosphatidylserine decarboxylase 2 [Fusarium fujikuroi]
hypothetical protein CEK25_002696 [Fusarium fujikuroi]
related to phosphatidylserine decarboxylase 2 [Fusarium fujikuroi IMI 58289]
hypothetical protein CEK27_002710 [Fusarium fujikuroi]
related to phosphatidylserine decarboxylase 2 [Fusarium fujikuroi]
related to phosphatidylserine decarboxylase 2 [Fusarium proliferatum]
uncharacterized protein FMAN_13931 [Fusarium mangiferae]
phosphatidylserine decarboxylase [Fusarium sp. NRRL 25303]
hypothetical protein FGADI_6212 [Fusarium gaditjirri]
phosphatidylserine decarboxylase [Fusarium globosum]
related to phosphatidylserine decarboxylase 2 [Fusarium proliferatum ET1]
hypothetical protein BFJ72_g7248 [Fusarium proliferatum]
hypothetical protein FPRO03_13411 [Fusarium proliferatum]
hypothetical protein FNYG_12419 [Fusarium nygamai]
hypothetical protein H9Q70_009419 [Fusarium xylarioides]
phosphatidylserine decarboxylase 2 [Fusarium mundagurra]
phosphatidylserine decarboxylase 2 [Fusarium phyllophilum]
phosphatidylserine decarboxylase 2 [Fusarium coicis]
hypothetical protein FVEG_08396 [Fusarium verticillioides 7600]
hypothetical protein J7337_012949 [Fusarium musae]
hypothetical protein FVER14953_08396 [Fusarium verticillioides]
phosphatidylserine decarboxylase 2 [Fusarium tjaetaba]
phosphatidylserine decarboxylase 2 [Fusarium napiforme]
phosphatidylserine decarboxylase 2 [Fusarium pseudoanthophilum]
phosphatidylserine decarboxylase 2 [Fusarium acutatum]
phosphatidylserine decarboxylase 2 [Fusarium subglutinans]
phosphatidylserine decarboxylase 2 [Fusarium circinatum]
phosphatidylserine decarboxylase [Fusarium bulbicola]
hypothetical protein FANTH_8126 [Fusarium anthophilum]
phosphatidylserine decarboxylase 2 [Fusarium mexicanum]
phosphatidylserine decarboxylase 2 [Fusarium agapanthi]
phosphatidylserine decarboxylase 2 [Fusarium pseudocircinatum]
phosphatidylserine decarboxylase 2 [Fusarium sp. NRRL 52700]
phosphatidylserine decarboxylase 2 [Fusarium beomiforme]
hypothetical protein IL306_001964 [Fusarium sp. DS 682]
hypothetical protein FGRMN_9363 [Fusarium graminum]
phosphatidylserine decarboxylase [Fusarium heterosporum]
hypothetical protein FAUST_11834 [Fusarium austroamericanum]
phosphatidylserine decarboxylase [Colletotrichum asianum]
L-tryptophan decarboxylase [Colletotrichum siamense]
L-tryptophan decarboxylase [Colletotrichum viniferum]
L-tryptophan decarboxylase [Colletotrichum aenigma]
Phosphatidylserine decarboxylase proenzyme 3 [Colletotrichum higginsianum]
Phosphatidylserine decarboxylase [Colletotrichum higginsianum IMI 349063]
Phosphatidylserine decarboxylase proenzyme [Colletotrichum tanaceti]
L-tryptophan decarboxylase [Colletotrichum spinosum]
L-tryptophan decarboxylase [Colletotrichum trifolii]
L-tryptophan decarboxylase [Colletotrichum orbiculare MAFF 240422]
putative phosphatidylserine decarboxylase family protein [Diaporthe ampelina]
hypothetical protein VSDG_10205 [Valsa sordida]
uncharacterized protein INS49_007219 [Diaporthe citri]
uncharacterized protein M409DRAFT_63671 [Zasmidium cellare ATCC 36951]
hypothetical protein D0867_01765 [Hortaea werneckii]
hypothetical protein D0865_09069 [Hortaea werneckii]
hypothetical protein D0869_06904 [Hortaea werneckii]
hypothetical protein D0865_05717 [Hortaea werneckii]
hypothetical protein D0869_04476 [Hortaea werneckii]
hypothetical protein D0868_01492 [Hortaea werneckii]
hypothetical protein D0864_04592 [Hortaea werneckii]
hypothetical protein BTJ68_06316 [Hortaea werneckii EXF-2000]
hypothetical protein D0861_02978 [Hortaea werneckii]
hypothetical protein B0A50_02262 [Hortaea thailandica]
uncharacterized protein CTRU02_01764 [Colletotrichum truncatum]
hypothetical protein A1O5_05824 [Cladophialophora psammophila CBS 110553]
hypothetical protein AYO20_07818 [Fonsecaea nubica]
hypothetical protein Z517_09732 [Fonsecaea pedrosoi CBS 271.37]
hypothetical protein AYO21_07581 [Fonsecaea monophora]
hypothetical protein AYL99_10010 [Fonsecaea erecta]
hypothetical protein KVT40_002358 [Elsinoe batatas]
Phophatidylserine decarboxylase-domain-containing protein [Elsinoe ampelina]
FTEMP_8_gene_25.87
Phophatidylserine decarboxylase-domain-containing protein [Microdochium bolleyi]
0.1

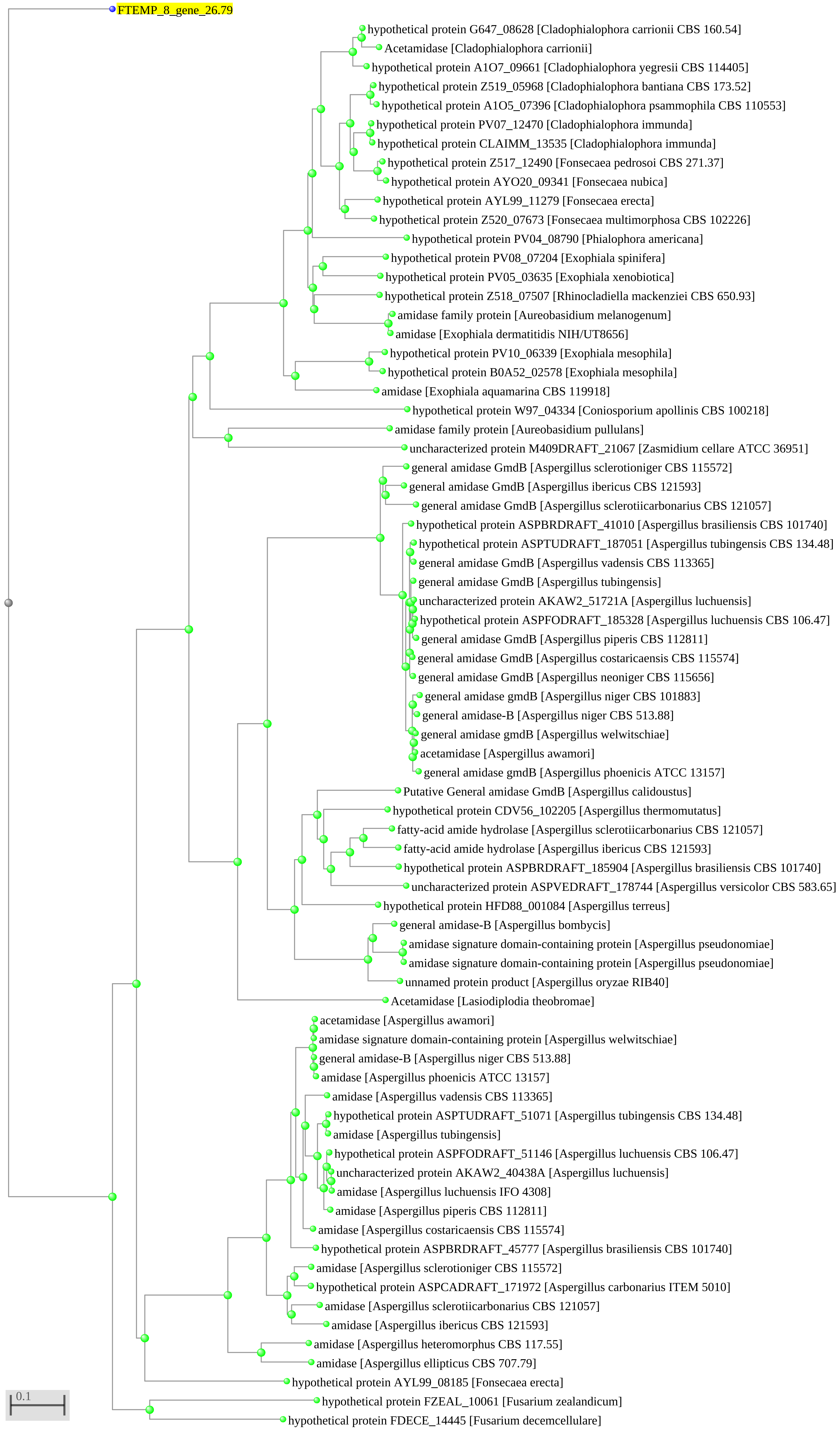
FTEMP_8_gene_26.79
hypothetical protein G647_08628 [Cladophialophora carrionii CBS 160.54]
Acetamidase [Cladophialophora carrionii]
hypothetical protein A1O7_09661 [Cladophialophora yegresii CBS 114405]
hypothetical protein Z519_05968 [Cladophialophora bantiana CBS 173.52]
hypothetical protein A1O5_07396 [Cladophialophora psammophila CBS 110553]
hypothetical protein PV07_12470 [Cladophialophora immunda]
hypothetical protein CLAIMM_13535 [Cladophialophora immunda]
hypothetical protein Z517_12490 [Fonsecaea pedrosoi CBS 271.37]
hypothetical protein AYO20_09341 [Fonsecaea nubica]
hypothetical protein AYL99_11279 [Fonsecaea erecta]
hypothetical protein Z520_07673 [Fonsecaea multimorphosa CBS 102226]
hypothetical protein PV04_08790 [Phialophora americana]
hypothetical protein PV08_07204 [Exophiala spinifera]
hypothetical protein PV05_03635 [Exophiala xenobiotica]
hypothetical protein Z518_07507 [Rhinocladiella mackenziei CBS 650.93]
amidase family protein [Aureobasidium melanogenum]
amidase [Exophiala dermatitidis NIH/UT8656]
hypothetical protein PV10_06339 [Exophiala mesophila]
hypothetical protein B0A52_02578 [Exophiala mesophila]
amidase [Exophiala aquamarina CBS 119918]
hypothetical protein W97_04334 [Coniosporium apollinis CBS 100218]
amidase family protein [Aureobasidium pullulans]
uncharacterized protein M409DRAFT_21067 [Zasmidium cellare ATCC 36951]
general amidase GmdB [Aspergillus sclerotioniger CBS 115572]
general amidase GmdB [Aspergillus ibericus CBS 121593]
general amidase GmdB [Aspergillus sclerotiicarbonarius CBS 121057]
hypothetical protein ASPBRDRAFT_41010 [Aspergillus brasiliensis CBS 101740]
hypothetical protein ASPTUDRAFT_187051 [Aspergillus tubingensis CBS 134.48]
general amidase GmdB [Aspergillus vadensis CBS 113365]
general amidase GmdB [Aspergillus tubingensis]
uncharacterized protein AKAW2_51721A [Aspergillus luchuensis]
hypothetical protein ASPFODRAFT_185328 [Aspergillus luchuensis CBS 106.47]
general amidase GmdB [Aspergillus piperis CBS 112811]
general amidase GmdB [Aspergillus costaricaensis CBS 115574]
general amidase GmdB [Aspergillus neoniger CBS 115656]
general amidase gmdB [Aspergillus niger CBS 101883]
general amidase-B [Aspergillus niger CBS 513.88]
general amidase gmdB [Aspergillus welwitschiae]
acetamidase [Aspergillus awamori]
general amidase gmdB [Aspergillus phoenicis ATCC 13157]
Putative General amidase GmdB [Aspergillus calidoustus]
hypothetical protein CDV56_102205 [Aspergillus thermomutatus]
fatty-acid amide hydrolase [Aspergillus sclerotiicarbonarius CBS 121057]
fatty-acid amide hydrolase [Aspergillus ibericus CBS 121593]
hypothetical protein ASPBRDRAFT_185904 [Aspergillus brasiliensis CBS 101740]
uncharacterized protein ASPVEDRAFT_178744 [Aspergillus versicolor CBS 583.65]
hypothetical protein HFD88_001084 [Aspergillus terreus]
general amidase-B [Aspergillus bombycis]
amidase signature domain-containing protein [Aspergillus pseudonomiae]
amidase signature domain-containing protein [Aspergillus pseudonomiae]
unnamed protein product [Aspergillus oryzae RIB40]
Acetamidase [Lasiodiplodia theobromae]
acetamidase [Aspergillus awamori]
amidase signature domain-containing protein [Aspergillus welwitschiae]
general amidase-B [Aspergillus niger CBS 513.88]
amidase [Aspergillus phoenicis ATCC 13157]
amidase [Aspergillus vadensis CBS 113365]
hypothetical protein ASPTUDRAFT_51071 [Aspergillus tubingensis CBS 134.48]
amidase [Aspergillus tubingensis]
hypothetical protein ASPFODRAFT_51146 [Aspergillus luchuensis CBS 106.47]
uncharacterized protein AKAW2_40438A [Aspergillus luchuensis]
amidase [Aspergillus luchuensis IFO 4308]
amidase [Aspergillus piperis CBS 112811]
amidase [Aspergillus costaricaensis CBS 115574]
hypothetical protein ASPBRDRAFT_45777 [Aspergillus brasiliensis CBS 101740]
amidase [Aspergillus sclerotioniger CBS 115572]
hypothetical protein ASPCADRAFT_171972 [Aspergillus carbonarius ITEM 5010]
amidase [Aspergillus sclerotiicarbonarius CBS 121057]
amidase [Aspergillus ibericus CBS 121593]
amidase [Aspergillus heteromorphus CBS 117.55]
amidase [Aspergillus ellipticus CBS 707.79]
hypothetical protein AYL99_08185 [Fonsecaea erecta]
hypothetical protein FZEAL_10061 [Fusarium zealandicum]
hypothetical protein FDECE_14445 [Fusarium decemcellulare]
0.1

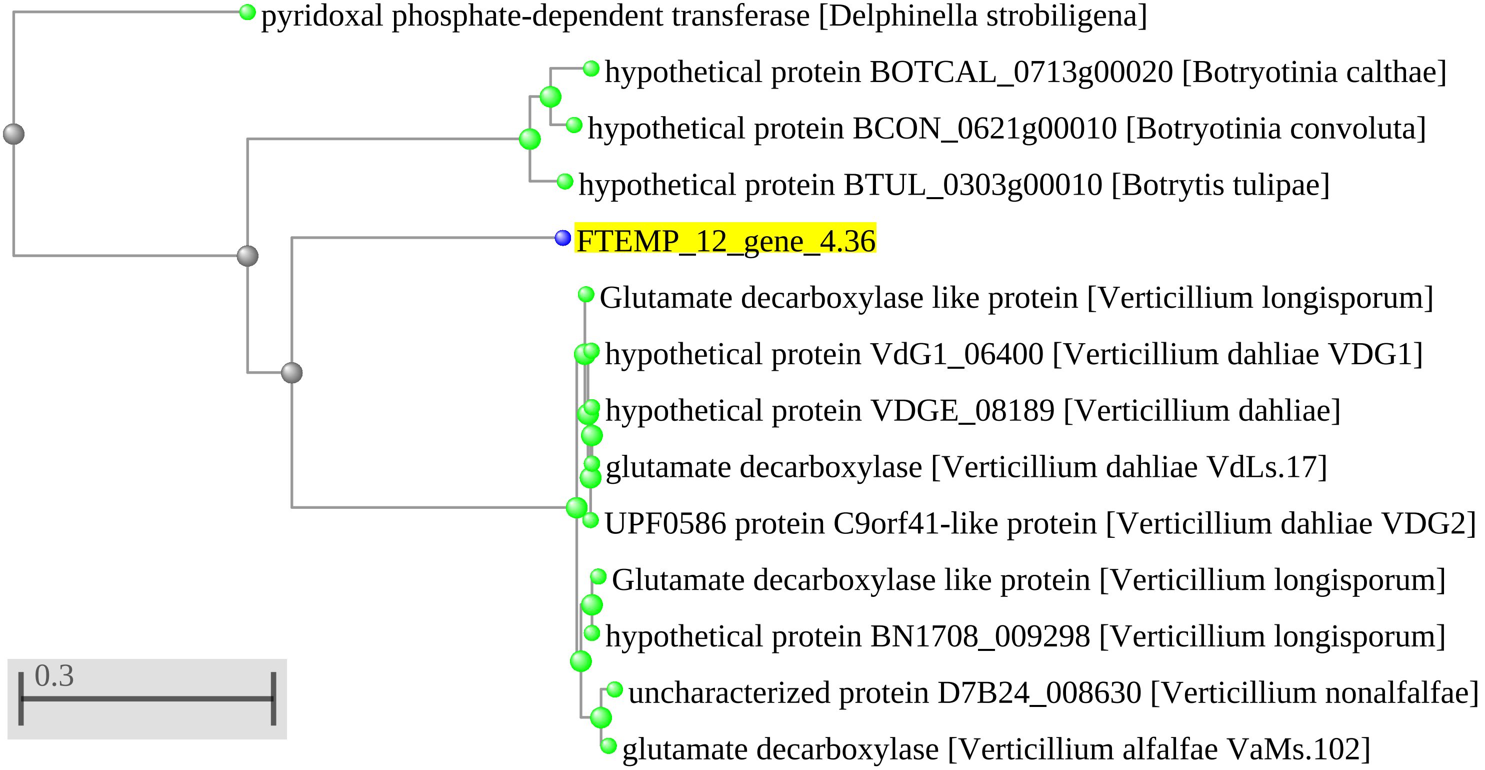
pyridoxal phosphate-dependent transferase [Delphinella strobiligena]
hypothetical protein BOTCAL_0713g00020 [Botryotinia calthae]
hypothetical protein BCON_0621g00010 [Botryotinia convoluta]
hypothetical protein BTUL_0303g00010 [Botrytis tulipae]
FTEMP_12_gene_4.36
Glutamate decarboxylase like protein [Verticillium longisporum]
hypothetical protein VdG1_06400 [Verticillium dahliae VDG1]
hypothetical protein VDGE_08189 [Verticillium dahliae]
glutamate decarboxylase [Verticillium dahliae VdLs.17]
UPF0586 protein C9orf41-like protein [Verticillium dahliae VDG2]
Glutamate decarboxylase like protein [Verticillium longisporum]
hypothetical protein BN1708_009298 [Verticillium longisporum]
uncharacterized protein D7B24_008630 [Verticillium nonalfalfae]
glutamate decarboxylase [Verticillium alfalfae VaMs.102]
0.3
