# Supplementary material for: Characterization of Host-Specific Genes from Pine- and Grass-Associated Species of the Fusarium fujikuroi Species Complex
Source: Pathogens. 2022 Jul 29;11(8):858. doi: 10.3390/pathogens11080858 (PMC9415769; doi:10.3390/pathogens11080858)
Supplement: Supplementary file 1 [file pathogens-11-00858-s001.zip › Supplemental Figures/Figure S8.pdf]

Figure S8. Host-range-associated genes with ancestral origins outside Fungi. Host-range-associated genes investigated are highlighted in yellow; FCIR = *Fusarium circinatum* and FTEMP = *Fusarium temperatum.*

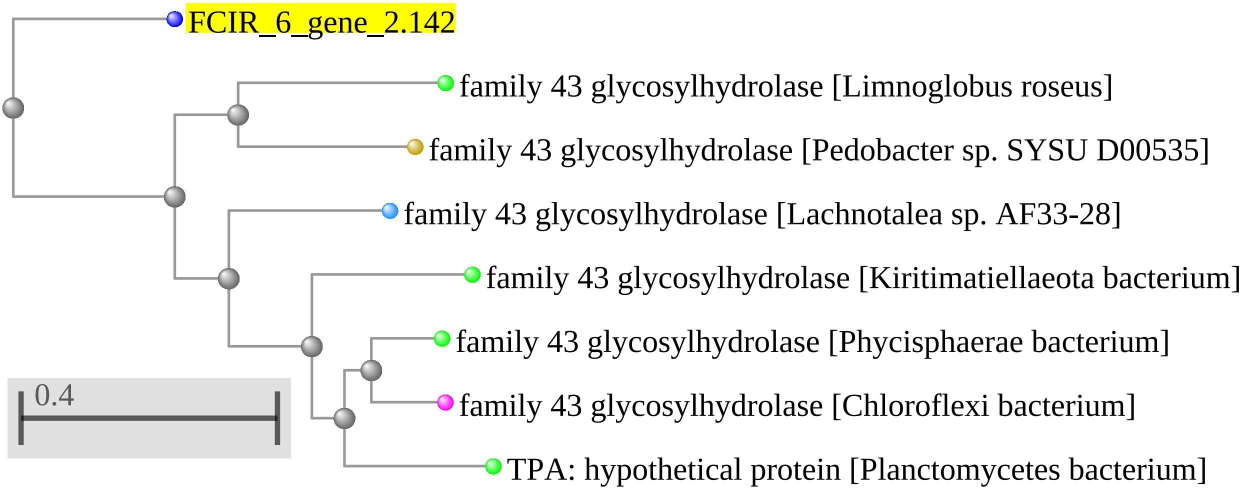
FCIR_6_gene_2.142
family 43 glycosylhydrolase [Limnoglobus roseus]
family 43 glycosylhydrolase [Pedobacter sp. SYSU D00535]
family 43 glycosylhydrolase [Lachnotalea sp. AF33-28]
family 43 glycosylhydrolase [Kiritimatiellaeota bacterium]
family 43 glycosylhydrolase [Phycisphaerae bacterium]
family 43 glycosylhydrolase [Chloroflexi bacterium]
TPA: hypothetical protein [Planctomycetes bacterium]
0.4

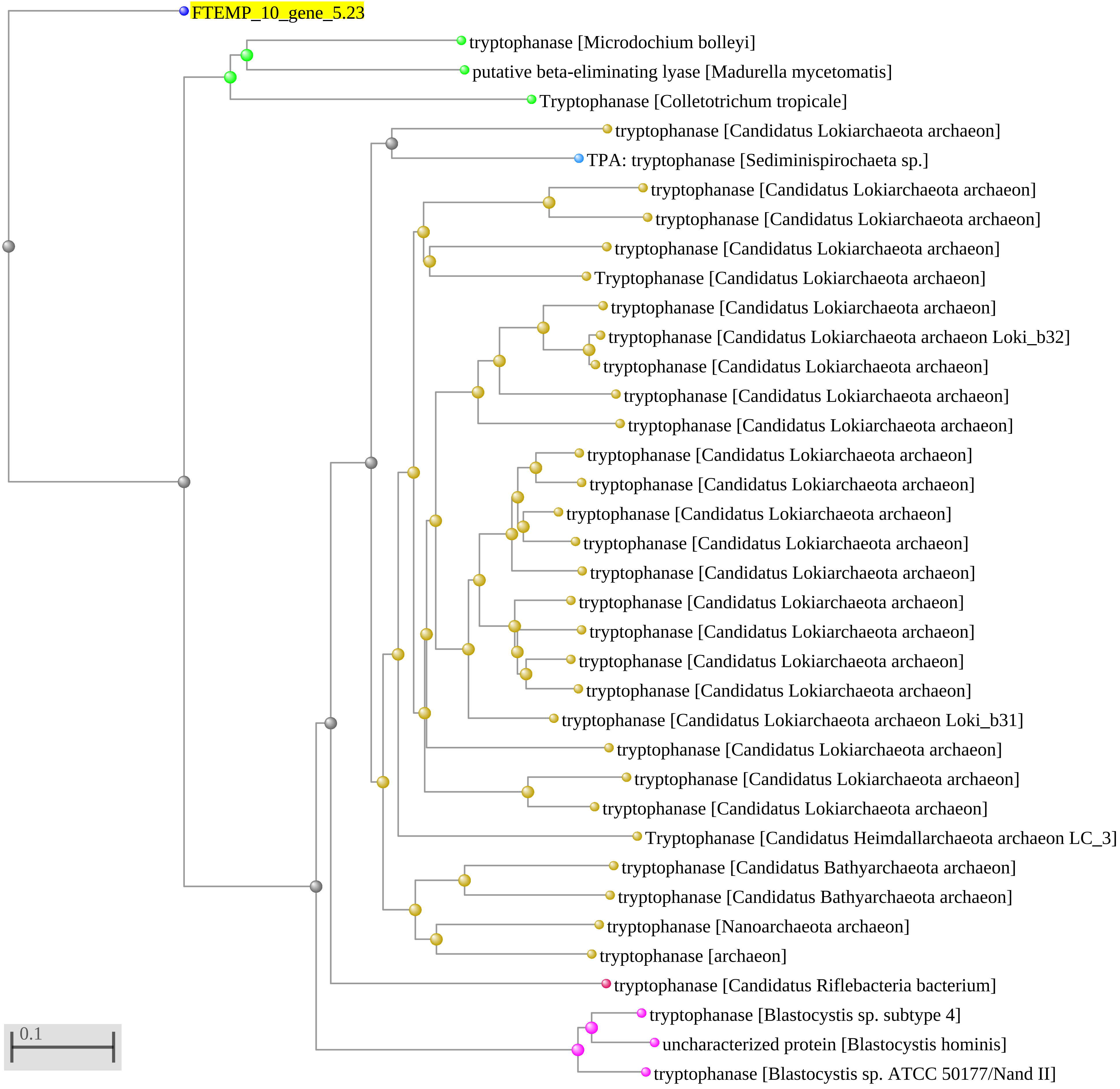
FTEMP_10_gene_5.23
tryptophanase [Microdochium bolleyi]
putative beta-eliminating lyase [Madurella mycetomatis]
Tryptophanase [Colletotrichum tropicale]
tryptophanase [Candidatus Lokiarchaeota archaeon]
TPA: tryptophanase [Sediminispirochaeta sp.]
tryptophanase [Candidatus Lokiarchaeota archaeon]
tryptophanase [Candidatus Lokiarchaeota archaeon]
tryptophanase [Candidatus Lokiarchaeota archaeon]
Tryptophanase [Candidatus Lokiarchaeota archaeon]
tryptophanase [Candidatus Lokiarchaeota archaeon]
tryptophanase [Candidatus Lokiarchaeota archaeon Loki_b32]
tryptophanase [Candidatus Lokiarchaeota archaeon]
tryptophanase [Candidatus Lokiarchaeota archaeon]
tryptophanase [Candidatus Lokiarchaeota archaeon]
tryptophanase [Candidatus Lokiarchaeota archaeon]
tryptophanase [Candidatus Lokiarchaeota archaeon]
tryptophanase [Candidatus Lokiarchaeota archaeon]
tryptophanase [Candidatus Lokiarchaeota archaeon]
tryptophanase [Candidatus Lokiarchaeota archaeon]
tryptophanase [Candidatus Lokiarchaeota archaeon]
tryptophanase [Candidatus Lokiarchaeota archaeon]
tryptophanase [Candidatus Lokiarchaeota archaeon]
tryptophanase [Candidatus Lokiarchaeota archaeon]
tryptophanase [Candidatus Lokiarchaeota archaeon Loki_b31]
tryptophanase [Candidatus Lokiarchaeota archaeon]
tryptophanase [Candidatus Lokiarchaeota archaeon]
tryptophanase [Candidatus Lokiarchaeota archaeon]
Tryptophanase [Candidatus Heimdallarchaeota archaeon LC_3]
tryptophanase [Candidatus Bathyarchaeota archaeon]
tryptophanase [Candidatus Bathyarchaeota archaeon]
tryptophanase [Nanoarchaeota archaeon]
tryptophanase [archaeon]
tryptophanase [Candidatus Riflebacteria bacterium]
tryptophanase [Blastocystis sp. subtype 4]
uncharacterized protein [Blastocystis hominis]
tryptophanase [Blastocystis sp. ATCC 50177/Nand II]
0.1
