# Supplementary material for: Characterization of Host-Specific Genes from Pine- and Grass-Associated Species of the Fusarium fujikuroi Species Complex
Source: Pathogens. 2022 Jul 29;11(8):858. doi: 10.3390/pathogens11080858 (PMC9415769; doi:10.3390/pathogens11080858)
Supplement: Supplementary file 1 [file pathogens-11-00858-s001.zip › Supplemental Figures/Figure S9.pdf]

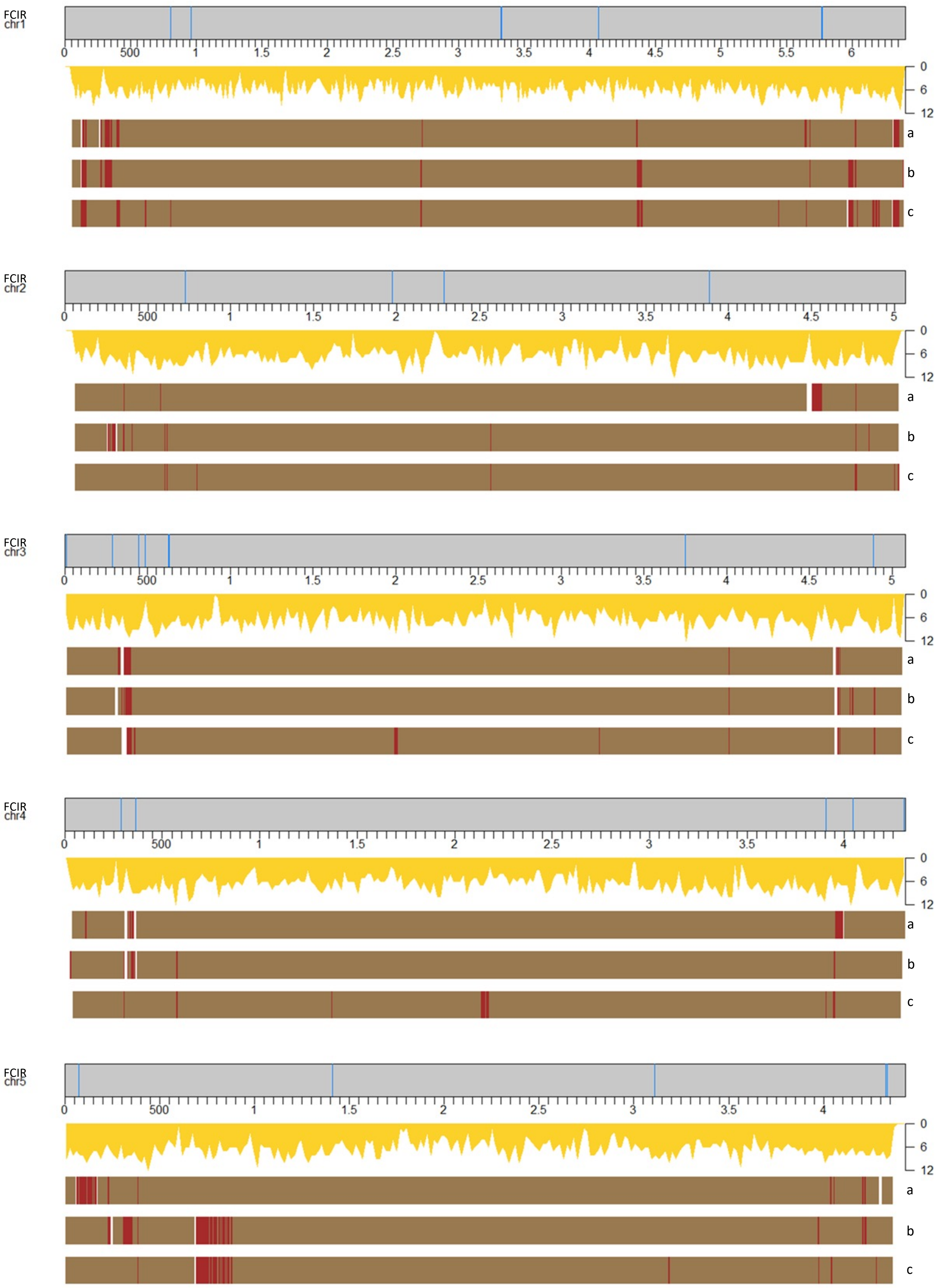
FCIR chr1
0 500 1 1.5 2 2.5 3 3.5 4 4.5 5 5.5 6
0 6 12
a
b
c
FCIR chr2
0 500 1 1.5 2 2.5 3 3.5 4 4.5 5
0 6 12
a
b
c
FCIR chr3
0 500 1 1.5 2 2.5 3 3.5 4 4.5 5
0 6 12
a
b
c
FCIR chr4
0 500 1 1.5 2 2.5 3 3.5 4
0 6 12
a
b
c
FCIR chr5
0 500 1 1.5 2 2.5 3 3.5 4
0 6 12
a
b
c

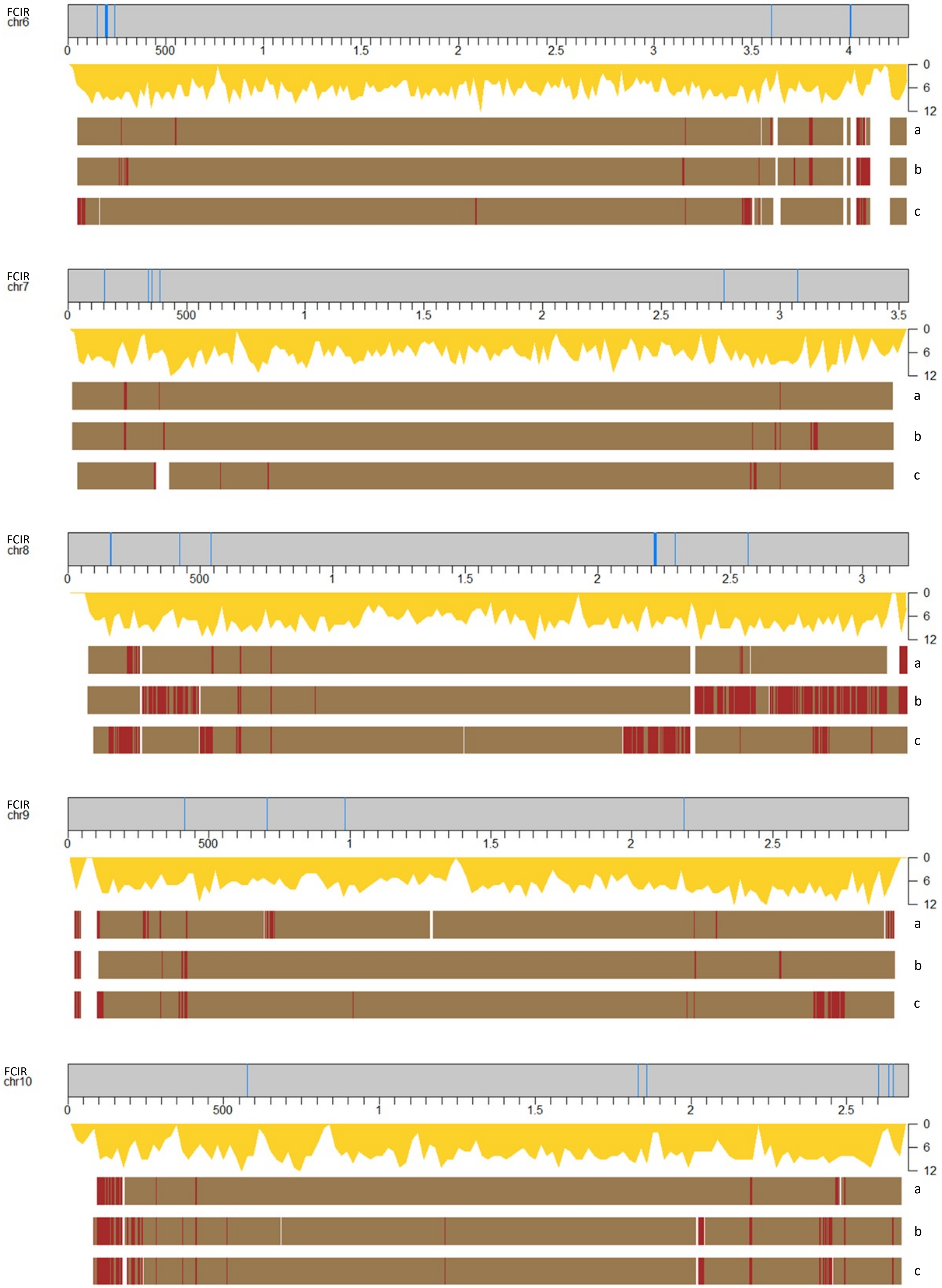
FCIR
chr6
0
500
1
1.5
2
2.5
3
3.5
4
0
6
12
a
b
c
FCIR
chr7
0
500
1
1.5
2
2.5
3
3.5
0
6
12
a
b
c
FCIR
chr8
0
500
1
1.5
2
2.5
3
0
6
12
a
b
c
FCIR
chr9
0
500
1
1.5
2
2.5
0
6
12
a
b
c
FCIR
chr10
0
500
1
1.5
2
2.5
0
6
12
a
b
c

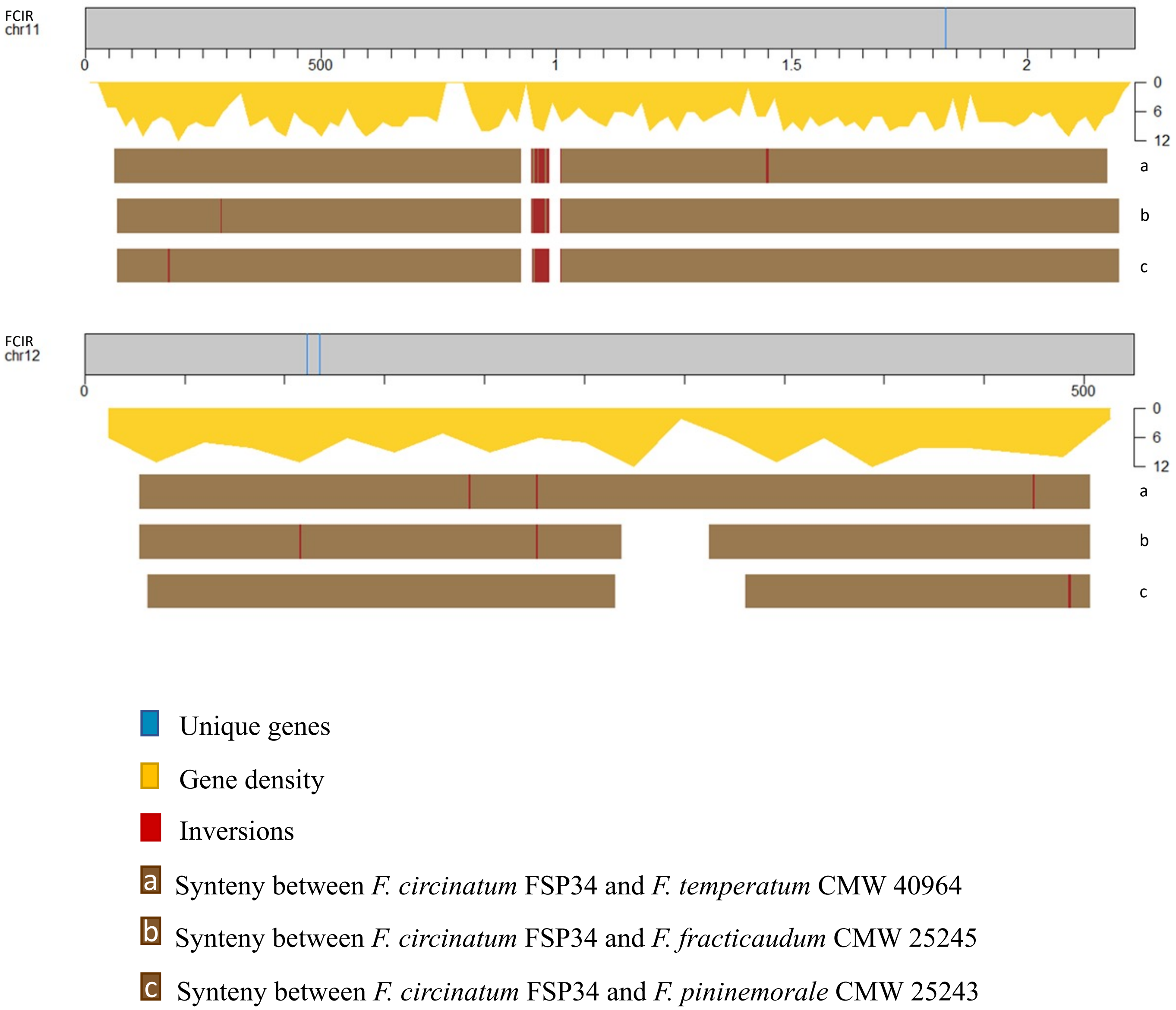

Figure S9. The distribution of pine-host-associated genes and conservation of synteny across and between chromosomes and genomes. Pine-host-associated genes distribution across each of the chromosomes as indicated by the blue lines. The conservation of synteny and inversion between the relevant genomes are indicated in the brown blocks and red lines. FCIR = *F. circinatum*; chromosome size is given in kbp.
