# Supplementary material for: Characterization of Host-Specific Genes from Pine- and Grass-Associated Species of the Fusarium fujikuroi Species Complex
Source: Pathogens. 2022 Jul 29;11(8):858. doi: 10.3390/pathogens11080858 (PMC9415769; doi:10.3390/pathogens11080858)
Supplement: Supplementary file 1 [file pathogens-11-00858-s001.zip › Supplemental Tables/Table S1.pdf]

Table S1. BUSCO results for the relevant *Fusarium* genomes.

Species	Total BUSCOs	Complete BUSCOs	Complete and single-copy BUSCOs	Complete and duplicated BUSCOs	Fragmented BUSCOs	Missing BUSCOs
F. circinatum	3 725	3 624 (97.3%)	3 616 (97.1%)	8 (0.2%)	75 (2.0%)	26 (0.7%)
F. fracticaudum	3 725	3 681 (98.8%)	3 670 (98.5%)	11 (0.3%)	29 (0.8%)	15 (0.4%)
F. pininemorale	3 725	3 688 (99.1%)	3 671 (98.6%)	17 (0.5%)	23 (0.6%)	14 (0.3%)
F. konzum	3 725	3 635 (97.6%)	3 625 (97.3%)	10 (0.3%)	66 (1.8%)	24 (0.6%)
F. subglutinans	3 725	3 677 (98.7%)	3 667 (98.4%)	10 (0.3%)	32 (0.9%)	16 (0.4%)
F. temperatum	3 725	3 681 (98.8%)	3 673 (98.6%)	8 (0.2%)	29 (0.8%)	15 (0.4%)
