# Supplementary material for: Characterization of Host-Specific Genes from Pine- and Grass-Associated Species of the Fusarium fujikuroi Species Complex
Source: Pathogens. 2022 Jul 29;11(8):858. doi: 10.3390/pathogens11080858 (PMC9415769; doi:10.3390/pathogens11080858)
Supplement: Supplementary file 1 [file pathogens-11-00858-s001.zip › Supplemental Tables/Table S2.pdf]

Table S2. The size difference between the chromosomes of four of the six *Fusarium* species.

F. circinatum			*F. fracticaudum*			*F. pininemorale*			*F. temperatum*		
**Chromosome**	**Size (bp)**	**Fold**	**Chromosome**	**Size (bp)**	**Fold**	**Chromosome**	**Size (bp)**	**Fold**	**Chromosome**	**Size (bp)**	**Fold**
**1**	6407589	1.06	1	6653466	1.02	1	6794846		1	6525753	1.04
**2**	5065897		2	4953649	1.02	2	5063039	1.00	2	5052385	1.00
**3**	5081688	1.03	3	4904252	1.06	3	5216703		3	4947561	1.05
**4**	4313068	1.10	4	4491441	1.05	4	4724792		4	4506647	1.05
**5**	4432453	1.09	5	4819172		5	4772958	1.01	5	4540351	1.06
**6**	4301695	1.08	6	4410177	1.05	6	4635304		6	4208847	1.10
**7**	3540654	1.02	7	3414008	1.06	7	3613395		7	3489342	1.04
**8**	3172515	1.05	8	3143385	1.06	8	3329509		8	3182830	1.05
**9**	2981544	1.03	9	2954973	1.04	9	3078815		9	2948229	1.04
**10**	2698620	1.02	10	2584499	1.06	10	2751664		10	2624843	1.05
**11**	2228220	1.07	11	2294545	1.04	11	2391144		11	2247932	1.06
**12**	525065	2.08	12	1094708		12	968722	1.13	12	986231	1.11

*Indicated in red is the chromosome which has the largest size amongst the four investigated *Fusarium* species.
