# Supplementary material for: Characterization of Host-Specific Genes from Pine- and Grass-Associated Species of the Fusarium fujikuroi Species Complex
Source: Pathogens. 2022 Jul 29;11(8):858. doi: 10.3390/pathogens11080858 (PMC9415769; doi:10.3390/pathogens11080858)
Supplement: Supplementary file 1 [file pathogens-11-00858-s001.zip › Supplemental Tables/Table S3.pdf]

Table S3. Presence of telomeres at chromosomal ends for the two representative *Fusarium* species examined.

Chromosome number	*F. circinatum*		*F. temperatum*	
	B[1]	E[2]	B[1]	E[2]
1	✓	✗	✓	✓
2	✗	✓	✗	✗
3	✓	✓	✓	✓
4	✓	✓	✓	✓
5	✗	✓	✓	✗
6	✓	✗	✓	✓
7	✓	✓	✓	✓
8	✓	✗	✓	✗
9	✓	✓	✓	✓
10	✓	✓	✓	✓
11	✓	✓	✓	✓
12	✓	✓	✓	✓

[1]Beginning of chromosome.

[2]End of chromosome.
