# Supplementary material for: Characterization of Host-Specific Genes from Pine- and Grass-Associated Species of the Fusarium fujikuroi Species Complex
Source: Pathogens. 2022 Jul 29;11(8):858. doi: 10.3390/pathogens11080858 (PMC9415769; doi:10.3390/pathogens11080858)
Supplement: Supplementary file 1 [file pathogens-11-00858-s001.zip › Supplemental Tables/Table S6.pdf]

Table S6. The Fischer exact test data for (A) the 72 unique pine-associated genes and (B) the 47 unique Poaceae-associated genes.

A

Representation	GO ID	GO Name	GO Category
[UNDER]	GO:0032991	protein-containing complex	CELLULAR_COMPONENT
[UNDER]	GO:0016021	integral component of membrane	CELLULAR_COMPONENT
[UNDER]	GO:0031224	intrinsic component of membrane	CELLULAR_COMPONENT
[UNDER]	GO:0044425	membrane part	CELLULAR_COMPONENT
[UNDER]	GO:0043170	macromolecule metabolic process	BIOLOGICAL_PROCESS

B

Representation	GO ID	GO Name	GO Category
[OVER]	GO:0004351	glutamate decarboxylase activity	MOLECULAR_FUNCTION
[OVER]	GO:0005871	kinesin complex	CELLULAR_COMPONENT
[OVER]	GO:0006536	glutamate metabolic process	BIOLOGICAL_PROCESS
[OVER]	GO:0016833	oxo-acid-lyase activity	MOLECULAR_FUNCTION
[OVER]	GO:0033764	steroid dehydrogenase activity, acting on the CH-OH group of donors, NAD or NADP as acceptor	MOLECULAR_FUNCTION
[OVER]	GO:0003854	3-beta-hydroxy-delta5-steroid dehydrogenase activity	MOLECULAR_FUNCTION
[OVER]	GO:0004523	RNA-DNA hybrid ribonuclease activity	MOLECULAR_FUNCTION
[OVER]	GO:0016229	steroid dehydrogenase activity	MOLECULAR_FUNCTION
[OVER]	GO:0005875	microtubule associated complex	CELLULAR_COMPONENT
[UNDER]	GO:0016830	carbon-carbon lyase activity	MOLECULAR_FUNCTION
[UNDER]	GO:0016829	lyase activity	MOLECULAR_FUNCTION
[UNDER]	GO:0016740	transferase activity	MOLECULAR_FUNCTION
[UNDER]	GO:0005634	nucleus	CELLULAR_COMPONENT
[UNDER]	GO:0043229	intracellular organelle	CELLULAR_COMPONENT
[UNDER]	GO:0043226	organelle	CELLULAR_COMPONENT
[UNDER]	GO:0044260	cellular macromolecule metabolic process	BIOLOGICAL_PROCESS

	[UNDER]	GO:0044424	intracellular part	CELLULAR_COMPONENT
	[UNDER]	GO:0005622	intracellular	CELLULAR_COMPONENT
	[UNDER]	GO:0044464	cell part	CELLULAR_COMPONENT
	[UNDER]	GO:0005623	cell	CELLULAR_COMPONENT
	[UNDER]	GO:0009987	cellular process	BIOLOGICAL_PROCESS
